# Machine Learning-Based Three-Month Outcome Prediction in Acute Ischemic Stroke: A Single Cerebrovascular-Specialty Hospital Study in South Korea

**DOI:** 10.3390/diagnostics11101909

**Published:** 2021-10-15

**Authors:** Dougho Park, Eunhwan Jeong, Haejong Kim, Hae Wook Pyun, Haemin Kim, Yeon-Ju Choi, Youngsoo Kim, Suntak Jin, Daeyoung Hong, Dong Woo Lee, Su Yun Lee, Mun-Chul Kim

**Affiliations:** 1 Department of Rehabilitation Medicine, Pohang Stroke and Spine Hospital, Pohang 37659, Korea; parkdougho@gmail.com; 2 Department of Neurology, Pohang Stroke and Spine Hospital, Pohang 37659, Korea; jeh132000@hanmail.net (E.J.); openmind-2u@hanmail.net (H.K.); 3 Department of Radiology, Pohang Stroke and Spine Hospital, Pohang 37659, Korea; biganhoo@naver.com; 4 Department of Neurosurgery, Pohang Stroke and Spine Hospital, Pohang 37659, Korea; soosungzzang@hanmail.net (H.K.); ns_yeonju@naver.com (Y.-J.C.); Youngsooooo@gmail.com (Y.K.); zin614@gmail.com (S.J.); hongdy2000@gmail.com (D.H.); nancbr2001@gmail.com (D.W.L.)

**Keywords:** ischemic stroke, functional outcome, algorithm, machine learning, prediction

## Abstract

Background: Functional outcomes after acute ischemic stroke are of great concern to patients and their families, as well as physicians and surgeons who make the clinical decisions. We developed machine learning (ML)-based functional outcome prediction models in acute ischemic stroke. Methods: This retrospective study used a prospective cohort database. A total of 1066 patients with acute ischemic stroke between January 2019 and March 2021 were included. Variables such as demographic factors, stroke-related factors, laboratory findings, and comorbidities were utilized at the time of admission. Five ML algorithms were applied to predict a favorable functional outcome (modified Rankin Scale 0 or 1) at 3 months after stroke onset. Results: Regularized logistic regression showed the best performance with an area under the receiver operating characteristic curve (AUC) of 0.86. Support vector machines represented the second-highest AUC of 0.85 with the highest F1-score of 0.86, and finally, all ML models applied achieved an AUC > 0.8. The National Institute of Health Stroke Scale at admission and age were consistently the top two important variables for generalized logistic regression, random forest, and extreme gradient boosting models. Conclusions: ML-based functional outcome prediction models for acute ischemic stroke were validated and proven to be readily applicable and useful.

## 1. Introduction

Stroke is a representative disease with high mortality and morbidity [1], with 30–70% of stroke survivors reportedly remaining disabled [2]. Functional disability, directly and indirectly, affects the rest of the patient’s life. It not only adversely affects the patient’s return to society and work but also places a burden on family members [3]. Moreover, disability is an important cause of psychiatric complications, such as depression, which significantly reduces the long-term quality of life of patients [4]; therefore, the prognosis related to functional ability after a stroke is understandably one of the most important concerns for patients and their families. In addition, the need for predicting functional recovery is required by physicians and surgeons who need to establish long-term treatment plans [5].

From these requirements, several prediction tools using the risk scoring method have been proposed for acute ischemic stroke, which account for the majority of stroke [6]. The representative tools are the Acute Stroke Registry and Analysis of Lausanne (ASTRAL) score and ischemic stroke predictive risk score (ISCORE) [7,8]. These tools have the advantage of being simple and readily available with few variables; therefore, they can make a quick prediction with only the findings within a short time after hospitalization [9]. In previous studies, external validation results for short-term functional outcome prediction of these tools have shown an area under the receiver operating characteristic curve (AUC) range of approximately 0.8, indicating that these tools were effective [10,11,12].

Meanwhile, research applying machine learning (ML) algorithms in disease diagnosis and prognosis prediction has been growing in recent years [13,14], and more specifically, studies using ML for outcome prediction in acute ischemic stroke have been published [15,16]. Compared with existing risk scoring methods, the ML-based prediction model has the advantage of being able to easily process a large number of samples and variables using electric health recording [17]. In addition, unlike fixed risk scoring tools, there is an evolutionary advantage that advanced ML-based models can be continuously established [18].

Based on this background, this study aimed to develop ML-based models capable of predicting the short-term favorable functional outcomes after acute ischemic stroke through the experience of a single cerebrovascular-specialty hospital in South Korea. Specifically, to share the advantageous features of existing risk scoring tools, the models were specifically developed to be applied within a short time after the stroke occurrence by using variables that can be utilized immediately after admission. Moreover, 37 variables were included by utilizing the advantages of ML modeling based on electric health recording to enable more precise and detailed outcome prediction. The predictive performances of several ML algorithms were evaluated and the best model was identified. Additionally, the variable importance related to the prediction performance was identified.

## 2. Materials and Methods

### 2.1. Data Source and Patients

**Figure 1:**
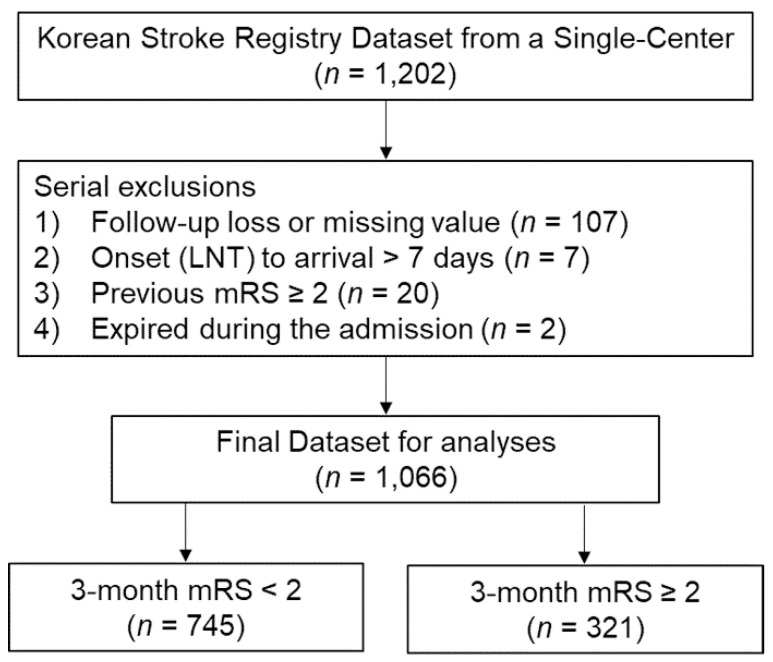
Flow chart of patient inclusion and exclusion. LNT, last normal time; mRS, modified Rankin Scale.

This retrospective study used a prospectively enrolled cohort dataset from a single center and was conducted using the electronic health records of patients admitted to Pohang Stroke and Spine Hospital with acute ischemic stroke between January 2019 and March 2021. This study was reviewed and approved by the Institutional Review Board (Approval number: PSSH0475-2021-08-HR-016-01) and performed in compliance with the Declaration of Helsinki and the International Conference on Harmonization–Good Clinical Practice Guideline. Simultaneously, all patients included in the study were enrolled in the multicenter, prospective, and hospital-based stroke registry (Korean Stroke Registry; www.strokedb.or.kr; accessed on 25 August 2021) in South Korea. Patients or their family members provided written informed consent to agree to the utilization of the patient’s non-identifiable information for research purposes when registered in the Korean Stroke Registry (Research management number: PSSH0475-201901-HR-001). We completed the final dataset for this study by applying the following exclusion criteria in order: (1) follow-up loss at 3 months, (2) having a missing value, (3) taking more than 7 days from the onset (or last normal time [LNT]) to hospitalization, (4) previous neurologic deficit (modified Rankin Scale [mRS] ≥ 2), and (5) patients who died during admission (one died from pneumonia and another from hemorrhagic transformation). A flowchart of patient inclusion for this study is shown in Figure 1.

### 2.2. Variables

Variables that were initially available on admission in patients with acute ischemic stroke were used. The detailed definitions of all the contributing variables are presented in the online Supplementary Materials (Document S1).

Personal factors, such as age, sex, body mass index, and abdominal circumference, were checked. We evaluated the National Institute of Health Stroke Scale (NIHSS) at admission, stroke subtype, onset type, onset (or LNT) to arrival time, circulatory territory, involved side, and the type of acute intravenous/intraarterial treatments as stroke-related factors. We also investigated the initial laboratory findings and blood pressure at the time of admission. Finally, comorbidities including previous stroke or transient ischemic attack, coronary artery disease, peripheral artery diseases, hypertension, diabetes, dyslipidemia, atrial fibrillation, smoking habit, previous administration of antiplatelet/anticoagulant, and potential sources of cardiogenic embolism were checked and confirmed for the patients as well.

The dependent variable was defined as mRS values at 3 months after the onset of the stroke and dichotomized for prediction. Favorable outcomes were defined as mRS scores of 0 and 1, and this group was designated as the target class, with follow-up mRS values measured at an outpatient clinic by a neurologist or neurosurgeon. In the case of not being able to visit the hospital, the mRS measurement was conducted in the form of a telephone interview with patients or their families through a structured questionnaire according to the Korean Stroke Registry guideline [19].

### 2.3. Data Analysis and Machine Learning Processes

**Figure 2:**
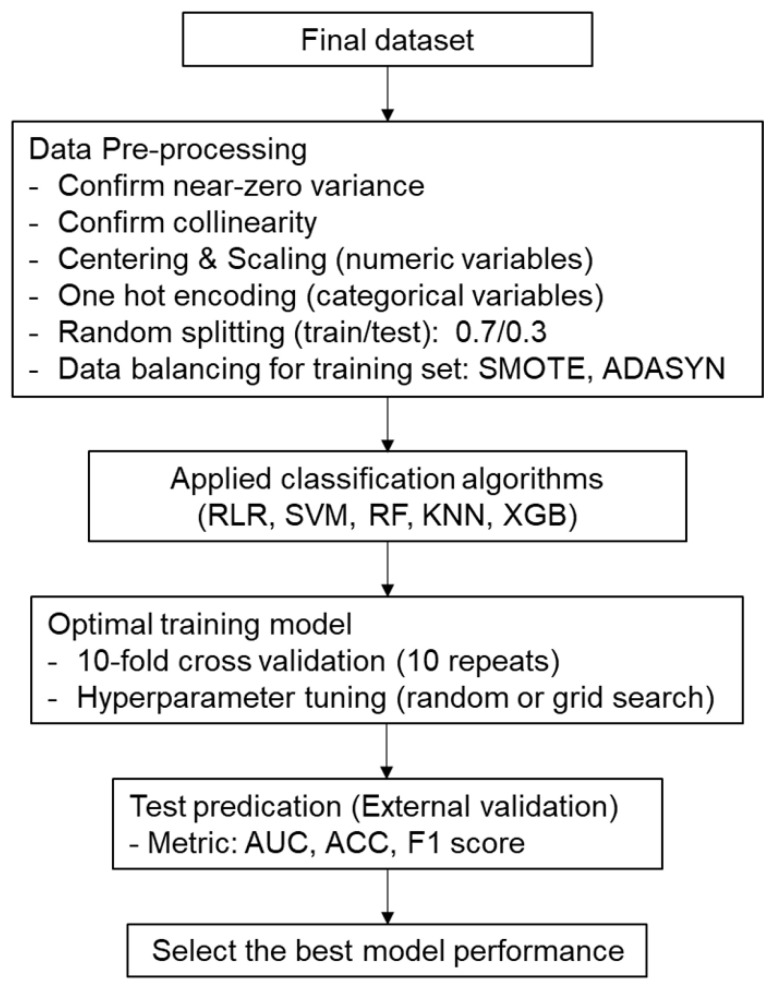
Entire machine learning modeling process for this study. SMOTE, synthetic minority oversampling technique; ADASYN, adaptive synthetic; RLR, regularized logistic regression; SVM, support vector machines; RF, random forest; KNN, k-nearest neighbors; XGB, extreme gradient boosting; AUC, area under the receiver operating characteristic curve; ACC, accuracy.

All statistical analyses and machine learning processes were performed using R software 4.1.0 (R Core Team, R Foundation for Statistical Computing, Vienna, Austria). The entire code used for data analyses and the ML processes in this study, as well as the corresponding results, are available in the online Supplementary Materials (Document S2). The entire ML modeling process is summarized in Figure 2.

Comparative analyses were conducted between outcome groups with mRS < 2 or mRS ≥ 2 at 3 months after stroke onset. Continuous variables were expressed as the mean ± standard deviation, and an independent *t*-test was used to compare the two groups. Categorical variables were presented as frequencies and proportions, and the chi-squared (trend) test was performed for comparative analysis, with the statistical significance defined as *p* < 0.05.

Serial data pre-processing was performed for ML algorithm training and validation. Variables with near-zero variances were identified and removed, with correlation coefficients evaluated to confirm the collinearity between continuous variables. After the variable selection was completed, centering and scaling for continuous variables and one-hot encoding for categorical variables were conducted. The dataset was then randomly split into training and test sets at a ratio of 7:3, and the synthetic minority oversampling technique (SMOTE) and adaptive synthetic (ADASYN) sampling were then applied to the training set to deal with the imbalance in the target class.

Five ML algorithms were evaluated in this study, namely, regularized logistic regression (RLR), support vector machines (SVM), random forest (RF), k-nearest neighbors (KNN), and extreme gradient boosting (XGB). Internal validation was performed by applying these ML algorithms to the training dataset, where pre-processing was completed. To obtain an optimal training model, 10-fold cross-validation with 10 repetitions was performed, with both random and grid search methods applied for hyperparameter tuning. An external validation was then performed on the test dataset using the trained models. This study used AUC as the main metric and investigated the F1-score and overall accuracy to evaluate model performance. The best-performing model was then selected for each ML algorithm based on external validation results. The variable importance from ensemble algorithms (RF and XGB) and a linear model (RLR) was also determined, with the top 10 important variables from each algorithm identified.

## 3. Results

### 3.1. Baseline Characteristics

Amongst the 1066 patients analyzed, 745 (69.9%) and 321 (30.1%) patients had favorable and unfavorable outcomes at the 3-month follow-up, respectively.

Table 1 describes personal and stroke-related features in both outcome groups. The favorable outcome group was 65.8 ± 11.3 years old, which was significantly younger than that of the unfavorable outcome group, 74.4 ± 11.4 years old (*p* < 0.001), and the proportion of men was significantly lower (33.7% vs. 48.6%; *p* < 0.001). In the favorable outcome group, NIHSS at admission was significantly lower (2.3 ± 3.2 vs. 6.3 ± 5.9; *p* < 0.001), and the proportion without acute intravenous/intraarterial treatments was significantly higher (90.3% vs. 79.1%; *p* < 0.001); however, in the favorable outcome group, significantly higher hemoglobin, and triglyceride levels (*p* < 0.001 and *p* = 0.004, respectively), and significantly lower random glucose and blood urea nitrogen levels (*p* = 0.028 and *p* = 0.007, respectively) were found.

Table 2 presents the underlying risk factors for both outcome groups. It was found that the ratio of current smokers was significantly higher in the favorable outcome group (*p* = 0.002), whilst atrial fibrillation was significantly higher in the unfavorable outcome group (*p* = 0.009). There were no significant differences in other risk factors found between the two groups.

### 3.2. Data Pre-Processing

The following variables were removed that showed near-zero variance: previous transient ischemic attack, previous peripheral artery diseases, previous cancer, previous administration of anticoagulant, and high and medium risks of potential sources of cardiogenic embolism. It should also be noted that none of the continuous variables showed any collinearity.

After random splitting, the training and test datasets were divided into 769 and 297 samples, respectively, with the proportions of favorable and unfavorable outcome groups in the training dataset being 537 and 232, respectively. After applying SMOTE, the ratios were 537 and 464, respectively, and after applying ADASYN, the ratios were 537 and 555, respectively. In the test dataset, the two outcome groups had ratios of 208 and 89.

### 3.3. Performances of Machine Learning Algorithms

**Figure 3:**
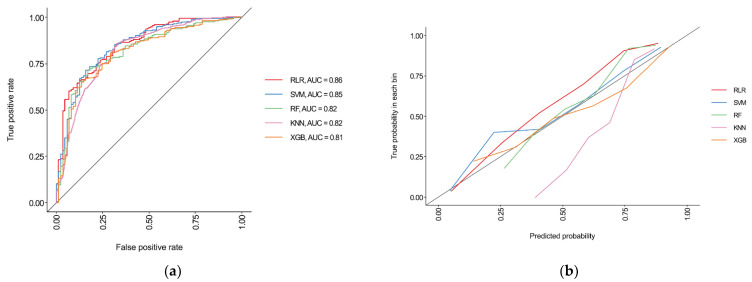
Results of external validation of each machine learning algorithm. (**a**) Receiver operating characteristic curves and (**b**) calibration plots are represented. The regularized logistic regression model showed the best performance with an AUC of 0.86 (red line). Overall, all the ML models showed AUC > 0.8. AUC, area under the receiver operating characteristic curve; RLR, regularized logistic regression; SVM, support vector machines; RF, random forest; KNN, k-nearest neighbors; XGB, extreme gradient boosting.

RLR showed the best performance among all ML algorithms utilized with an AUC of 0.86 (95% confidence interval [CI], 0.82–0.90). Meanwhile, SVM showed the second-highest AUC of 0.85 (95% CI, 0.81–0.89) and recorded the highest overall accuracy and F1-score (0.80 and 0.86, respectively). RF and KNN showed an AUC value of 0.82 (95% CI, 0.77–0.87), and XGB was 0.81 (95% CI, 0.76–0.86) (Table 3 and Figure 3). The confusion matrix for each ML algorithm is presented in Supplementary Table S1.

Among the optimal training models of the best test prediction result for each ML algorithm, SVM, XGB, and KNN showed the best performance when target class balancing was not performed. Contrarily, the best-performed model was generated in RLR and RF when SMOTE and ADASYN were applied, respectively. Supplementary Table S2 shows the balancing method and hyperparameter tuning results for the best model of each ML algorithm.

### 3.4. Variable Importance

**Figure 4:**
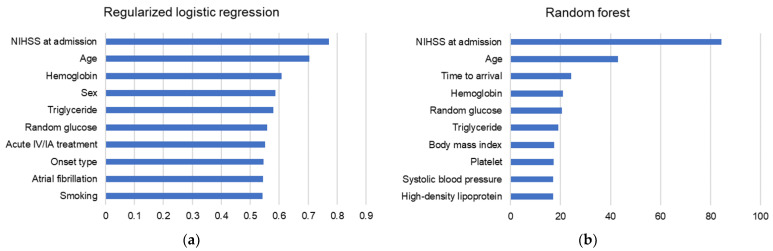

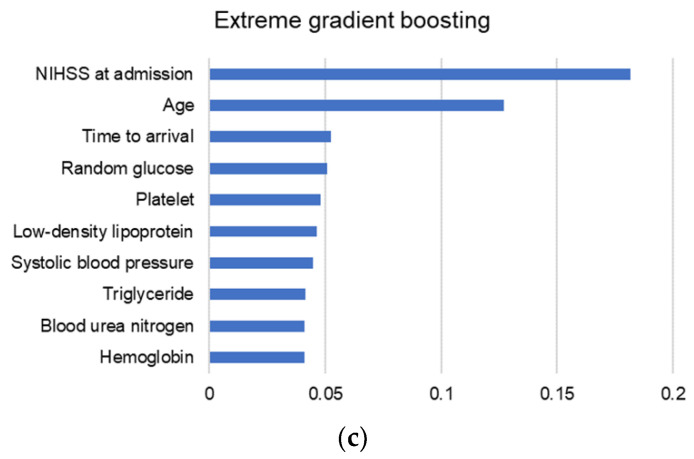
Top ten important variables in (**a**) regularized logistic regression, (**b**) random forest, and (**c**) extreme gradient boosting models. The top two important features were consistent in all three models; NIHSS at admission, followed by age. Additionally, random glucose, hemoglobin, and triglyceride were also included in the top ten important variables in all three models. NIHSS, National Institute of Health Stroke Scale; IV, intravenous; IA, intraarterial.

For RLR, NIHSS was the most important variable for model performance, followed by age and hemoglobin. Both RF and XGB represented the same top three results of variable importance; the most important variable for the prediction performance was the NIHSS score at admission, followed by age and time to arrival. NHISS at admission and age were also the two most important variables in the three ML models. Finally, random glucose, hemoglobin, and triglyceride levels were identified as the top ten important variables in all three models (Figure 4).

## 4. Discussion

This study demonstrated models that predict the short-term functional prognosis of patients with acute ischemic stroke using ML algorithms. All ML algorithms utilized showed validated results with AUC > 0.8. In particular, the proposed models were established based on initial evaluation and examination findings at the time of admission, which has the advantage of being able to predict a favorable outcome within a short time after hospitalization. Moreover, this feature can be useful to physicians and surgeons in making clinical decisions or informing patients and their families.

The proposed ML models’ performance was either similar to or slightly better than existing risk scoring tools such as ASTRAL and ISCORE; nevertheless, it is difficult to conclude that our ML models using more variables are a much-improved prediction tool. The reason for this finding can be attributed to the following. The first is the difference in outcome definition. Unlike both ASTRAL and ISCORE, where mRS 0–2 was defined as a good outcome, only mRS 0 and 1 were defined as favorable outcomes in this study. According to mRS [20], if based on dependency, grade 2 or less can be viewed as a favorable outcome; however, when a narrow favorable outcome criterion was applied, we considered the absence of disability and maintenance of usual daily activities; therefore, it is difficult to compare these two risk scoring tools directly with the proposed ML models because of the different target outcome definitions. The second reason can be inferred from the results of the variable importance. The ASTRAL score is based on six contributing factors: age, NIHSS score, time delay, visual field defect, glucose level, and level of consciousness [7]. ISCORE is calculated based on age, sex, preadmission functionality, cancer, atrial fibrillation, congestive heart failure, renal function, stroke subtype, glucose level, and stroke severity [8]. There is a significant overlap between the contributing factors of these two tools and the variables with high importance in our ML models. Several more variables were utilized compared to the existing risk scoring tools by taking advantage of ML-based data processing; however, it was found that they shared a similar critical variables list.

There have been previous studies on the ML-based predictions of functional outcomes in acute ischemic stroke. Heo et al. [11] predicted a favorable functional outcome at the 3-month follow-up. They designated mRS 0–2 as the target class and directly compared the predictive power of their ML models to the ASTRAL score. Hence, the deep neural network recorded a significantly higher AUC than the ASTRAL score; however, there was no significant difference in the AUC between the ASTRAL score and RF and logistic regression. Alaka et al. [21] also presented ML-based models for predicting the 3-month functional outcome by targeting mRS > 2 in ischemic stroke. The external validation results in their study were inferior to our study, with an AUC range of 0.66 to 0.71. This difference is thought to be due to our model using more sample sizes and variables than theirs. Jang et al. [22] defined mRS > 1 at 3 months in acute ischemic stroke as a bad outcome and performed an analysis based on the same outcome class classification as ours. Among the ML models presented by them, XGB, RF, and SVM showed the highest AUC value of 0.84; our RLR and SVM models slightly outperformed the other models with higher AUC values.

Among the ML algorithms we applied, RLR showed the best performance. Regularization lowers the weight of the parameter to reduce the complexity of the dataset and prevent overfitting [23,24]. Thus, a linear model sometimes outperforms ensemble algorithms, such as RF and XGB [25,26]. We were able to optimally train our best model by applying L1 regularization, which avoids overfitting by increasing the sparsity [27]. Based on this, it is inferred that our prediction model performed better on the dataset with lower complexity in a relatively limited manner. Consequently, our linear model showed better prediction performance than the other ML algorithms in this study.

The target class in the dataset is slightly imbalanced. We applied the SMOTE and ADASYN methods to address this problem. Both SMOTE and ADASYN are KNN-based up-sampling methods [28,29]. We obtained the best results by applying SMOTE to our best model, RLR. RF was able to obtain optimal results by applying ADASYN; however, in SVM and KNN, the training model without balancing showed better results, which is thought to be because the balancing of the training set caused overfitting. XGB can avoid overfitting even with balancing methods; however, the AUC was slightly higher when we did not implement balancing methods. Consequently, to create an ideal ML-based prediction model, it is necessary to confirm the results derived from the original dataset as well as the results with target class balancing. Additionally, it is necessary to consider overfitting caused by up-sampling.

Our ML models showed relatively low specificity, which is thought to be because our target was the majority class. We chose AUC as the main metric because it is not affected by the majority or minority of the target class [30,31]. In contrast, we should interpret the overall accuracy with caution because majority class predictivity may be overestimated in imbalanced data [32]. In fact, we selected the RLR model as the best model because it showed the test prediction result with the highest AUC and the most balanced sensitivity and specificity values among the investigated models.

There were several limitations to this study. First, mRS was used as an indicator of functional outcome, which had the advantage of an intuitive understanding of the functional level; however, it can be challenging to obtain a detailed reflection of the various neurologic symptoms observed after ischemic stroke, such as dysarthria-clumsy hand, ataxic hemiparesis, and pure sensory stroke. In particular, there may be a discrepancy between the measured functional score and the discomfort of the symptoms felt by the patient in these subtypes [33]; therefore, it was believed that a better model could be presented if more specific clinical data were added. Second, it was a single-center study. Therefore, an integrated, multicenter study is required to generalize this study’s result and may present better results. Finally, relatively broad inclusion criteria were also applied to develop a generally applicable model for acute ischemic stroke, which may have contributed to the bias.

## 5. Conclusions

This study demonstrated that ML-based models early and effectively predicted favorable functional outcomes at 3 months after acute ischemic stroke. All ML-based prediction models in this study showed validated results, with an AUC > 0.8. In particular, RLR showed the best performance, with SVM showing promising results as well. Both models exhibited similar or slightly better performance than existing risk scoring tools or previously proposed ML-based prediction models; moreover, they are useful because they are readily applicable, informative to patients and families, and support clinical decision-making.

## Figures and Tables

**Table 1:** Baseline characteristics and stroke-related factors in each group.

Variables	mRS < 2 (*n* = 745)	mRS ≥ 2 (*n* = 321)	*p* Value
Age, years old	65.8 ± 11.3	74.4 ± 11.4	<0.001
Male, *n* (%)	251 (33.7)	156 (48.6)	<0.001
Body mass index, kg/m^2^	24.2 ± 3.1	23.8 ± 3.5	0.060
Abdominal circumference, cm	85.2 ± 9.2	84.6 ± 10.2	0.310
NIHSS at admission	2.3 ± 3.2	6.3 ± 5.9	<0.001
Clear onset, *n* (%)	605 (81.2)	245 (76.3)	0.082
Onset (or LNT) to arrival, day	1.4 ± 1.6	1.2 ± 1.5	0.120
Stroke subtype, *n* (%)			0.066
Small vessel occlusion	289 (38.8)	106 (33.0)	
Large artery atherosclerosis	280 (37.6)	143 (44.5)	
Cardiogenic embolism	79 (10.6)	40 (12.5)	
Others	97 (13.0)	32 (10.0)	
Circulation, *n* (%)			0.097
Anterior	521 (69.9)	244 (76.0)	
Posterior	223 (29.9)	76 (23.7)	
Multiple	1 (0.1)	1 (0.3)	
Side, *n* (%)			0.264
Right	350 (47.0)	153 (47.7)	
Left	341 (45.8)	136 (42.4)	
Bilateral	54 (7.2)	32 (10.0)	
Acute treatment, *n* (%)			<0.001
None	673 (90.3%)	254 (79.1%)	
Intravenous only	13 (1.7%)	10 (3.1%)	
Intraarterial only	38 (5.1%)	42 (13.1%)	
Intravenous and intraarterial	21 (2.8%)	15 (4.7%)	
Systolic blood pressure, mmHg	156.6 ± 27.0	159.0 ± 26.1	0.179
Diastolic blood pressure, mmHg	87.6 ± 15.4	87.1 ± 16.3	0.609
Hemoglobin, g/dl	14.1 ± 1.7	13.4 ± 1.9	<0.001
Platelet, 10^3^/µL	238.3 ± 66.1	231.5 ± 68.6	0.125
Total cholesterol, mg/dl	191.8 ± 46.5	188.6 ± 48.4	0.305
Triglyceride, mg/dl	166.4 ± 131.4	141.2 ± 127.2	0.004
High density lipoprotein, mg/dl	49.3 ± 12.2	48.1 ± 11.6	0.135
Low density lipoprotein, mg/dl	109.4 ± 40.6	113.0 ± 42.6	0.189
Random glucose, mg/dl	140.8 ± 57.1	149.7 ± 62.6	0.028
HbA1c, %	6.2 ± 1.2	6.4 ± 1.4	0.077
Blood urea nitrogen, mg/dl	16.4 ± 5.4	17.7 ± 7.6	0.007
Creatinine, mg/dl	0.9 ± 0.3	0.9 ± 0.4	0.389

mRS, modified Rankin Scale; NIHSS, National Institutes of Health Stroke; LNT, last normal time.

**Table 2:** Underlying risk factors in each group.

Variables	mRS < 2 (*n* = 745)	mRS ≥ 2 (*n* = 321)	*p* Value
Previous stroke, *n* (%)			0.785
None	640 (85.9)	270 (84.1)	
Hemorrhagic	17 (2.3)	9 (2.8)	
Ischemic	85 (11.4)	40 (12.5)	
Mixed	2 (0.3)	2 (0.6)	
Unknown	1 (0.1)	0 (0.0)	
Previous TIA, *n* (%)	14 (1.9)	4 (1.2)	0.633
Previous PAD, *n* (%)	4 (0.5)	3 (0.9)	0.746
Previous CAD, *n* (%)	72 (9.7)	38 (11.8)	0.337
Previous cancer, *n* (%)	9 (1.2)	5 (1.6)	0.868
Previous hypertension, *n* (%)			0.224
None	293 (39.3)	109 (34.0)	
Known	395 (53.0)	188 (58.6)	
Diagnosed at admission	57 (7.7)	24 (7.5)	
Previous diabetes, *n* (%)			0.087
None	556 (74.6)	223 (69.5)	
Known	159 (21.3)	88 (27.4)	
Diagnosed at admission	30 (4.0)	10 (3.1)	
Previous dyslipidemia, *n* (%)			0.650
None	278 (37.3)	122 (38.0)	
Known	222 (29.8)	87 (27.1)	
Diagnosed at admission	245 (32.9)	112 (34.9)	
Smoking, *n* (%)			0.002
None	499 (67.0)	252 (78.5)	
Current	242 (32.5)	69 (21.5)	
Quit ≥ 5 years	2 (0.3)	0 (0.0)	
Quit < 5 years	2 (0.3)	0 (0.0)	
Previous AF, *n* (%)			0.009
None	658 (88.3)	261 (81.3)	
Known	41 (5.5)	30 (9.3)	
Diagnosed at admission	46 (6.2)	30 (9.3)	
Previous antiplatelet, *n* (%)	191 (25.6)	81 (25.2)	0.950
Previous anticoagulation, *n* (%)	19 (2.6)	14 (4.4)	0.170
PSCE-high risk, *n* (%)	20 (2.7)	8 (2.5)	>0.999
PSCE-medium risk, *n* (%)	34 (4.6)	17 (5.3)	0.721

mRS, modified Rankin Scale; TIA, transient ischemic attack; PAD, peripheral artery disease; CAD, coronary artery disease; AF, atrial fibrillation; PSCE, potential sources of cardiogenic embolism.

**Table 3:** Model performances of each machine learning model on the test dataset.

Metric	RLR	SVM	RF	KNN	XGB
AUC (95% CI)	0.86 (0.82–0.90)	0.85 (0.81–0.89)	0.82 (0.77–0.87)	0.82 (0.77–0.87)	0.81 (0.76–0.86)
F1	0.84	0.86	0.85	0.84	0.85
Accuracy (95% CI)	0.78 (0.73–0.83)	0.80 (0.75–0.85)	0.77 (0.72–0.82)	0.73 (0.67–0.78)	0.77 (0.72–0.82)
Sensitivity (95% CI)	0.83 (0.77–0.87)	0.90 (0.86–0.94)	0.89 (0.83–0.92)	0.99 (0.97–1.00)	0.89 (0.84–0.93)
Specificity (95% CI)	0.69 (0.58–0.77)	0.56 (0.46–0.66)	0.53 (0.43–0.63)	0.10 (0.05–0.18)	0.50 (0.39–0.60)
Precision (95% CI)	0.86 (0.81–0.90)	0.83 (0.77–0.87)	0.81 (0.76–0.86)	0.72 (0.67–0.77)	0.80 (0.75–0.85)
NPV (95% CI)	0.63 (0.53–0.72)	0.71 (0.60–0.81)	0.66 (0.55–0.76)	0.90 (0.60–0.98)	0.66 (0.54–0.76)

RLR, regularized logistic regression; SVM, support vector machines; RF, random forest; KNN, k-nearest neighbors; XGB, extreme gradient boosting; AUC, area under the receiver operating characteristic curve; ACC, accuracy; NPV, negative predictive value.

## Data Availability

Data are not publicly available because of privacy and ethical restrictions on the data sharing regulation of the Korean Stroke Registry. Only authorized researchers can access the dataset used in this study (www.stokedb.or.kr; accessed on 25 August 2021). The data analyses and machine learning results for this study are available in the online supplementary content.

## Supplementary Materials

The following are available online at www.mdpi.com/article/10.3390/diagnostics11101909/s1, Table S1: Optimal training model of each machine learning algorithm, Table S2: Confusion matrix, Document S1: Variable definitions and measurements, Document S2: R Markdown.
