# Supplementary material for: Machine Learning-Based Three-Month Outcome Prediction in Acute Ischemic Stroke: A Single Cerebrovascular-Specialty Hospital Study in South Korea
Source: Diagnostics (Basel). 2021 Oct 15;11(10):1909. doi: 10.3390/diagnostics11101909 (PMC8534707; doi:10.3390/diagnostics11101909)
Supplement: Supplementary file 1 [file diagnostics-11-01909-s001.zip › Document S1.pdf]

Supplementary Document S1. Variable definitions and measurements

(According to the Korean Stroke Registry Guideline version 1.0[1])

Personal factors

1. Age (numerical): Automatically calculated after entering birthday.
2. Sex (categorical): simply fill-up
3. Body mass index (numerical)
    - Automatically calculated after entering height and weight.
    - Write down known height and weight, or measure and record if they are not known.
4. Abdominal circumference (numerical)
    - Position: standing with feet apart 25–30 cm apart while exhaling in a comfortable standing position, evenly distribute weight.
    - Measure at the midpoint between the lower ribs and the upper iliac bones.

Personal factors

1. Age (numerical): Automatically calculated after entering birthday.
2. Sex (categorical): simply fill-up
3. Body mass index (numerical)
    - Automatically calculated after entering height and weight.
    - Write down known height and weight, or measure and record if they are not known.
4. Abdominal circumference (numerical)
    - Position: standing with feet 25–30 cm apart while exhaling in a comfortable standing position, evenly distribute weight.
    - Measure at the midpoint between the lower ribs and the upper iliac bones.

Stroke-related factors

1. Initial National Institute of Health Stroke Scale (numerical)
    - Based only on the neurological symptoms caused by index stroke.
2. Stroke subtype (categorical)
    - Following the TOAST classification system.[2]
    - Categories: Large artery atherosclerosis, cardiogenic embolism, small vessel disease, and others.
3. Circulatory territory (categorical) & Involved side (categorical)
    - Records responsible lesion of the index stroke.

- If a lesion has invaded diverse territories, it is indicated in multiple.
- Angiography findings can be applied.
- Categories: anterior circulation, posterior circulation, and both for circulatory territory. Right, left, and bilateral for the involved side.

4. Acute intravenous/intraarterial treatments
   - Describe all treatments performed within 48 hours of admission.
   - Categories: None, intravenous tissue plasminogen activator, intraarterial thrombolysis and intravenous/intraarterial combined.
5. Onset type (categorical) and time to arrival (numerical)
   - Clear onset: When the last known normal time (LNT) and the first known abnormal time (FAT) of neurological symptoms are the same before the onset of stroke (LNT=FAT).
   - Unclear onset: Wake up stroke or Daytime unwitnessed stroke.
   - Time to arrival = LNT to hospital arrival

Laboratory findings (numerical) and blood pressure (numerical)

- Laboratory findings were recorded as the results of the first examination after admission and in the case of in-hospital strokes, as the first examination results after the onset of newly developed symptoms. (Except for serum lipid levels)
- For serum lipid levels, the measurement should be conducted after an 8 hours-empty stomach.
- Initial blood pressure: record the first blood pressure measured in a stable state.

Comorbidities (Risk factors)

1. Stroke (categorical)
   - Based on patient's or families' statements or medical records.
   - Complete stroke is defined as a sudden onset of localized brain dysfunction lasting more than 24 h.
   - Silent “lacunar” infarction is not a target.
   - Categories: None, Ischemic stroke, hemorrhagic stroke, mixed stroke, unknown.
2. Transient ischemic attack (categorical)
   - Based on patient's or families' statements or medical records.
   - It is defined as a sudden onset of localized brain dysfunction lasting less than 24 h and then completely recovers.
3. Cardiac artery disease (categorical)

- Based on patient's or families' statements or medical records.
- History of myocardial infarction.
- Evidence of asymptomatic myocardial infarction and myocardial ischemia on electrocardiogram or echocardiography.
- History of stable or unstable angina.
- History of coronary angioplasty or surgery.

4. Peripheral artery diseases (categorical)
    - Having been diagnosed with peripheral arterial disease in a hospital.
    - History of surgical or interventional treatment for peripheral arterial disease.
5. Hypertension (categorical): If one or more of the following apply.
    - Categories: None, known, and diagnosed at admission.
    - He was diagnosed with hypertension in a hospital and had a history of taking antihypertensive drugs (including cases that stopped taking medication) (known).
    - Having been told that high blood pressure should be treated at the hospital, the patient has never taken any medications (known).
    - Blood pressure higher than 140/90 was measured more than twice in self-measurement or health checkups even without a history of HTN (known).
    - In cases of high blood pressure of 140/90 mmHg or higher twice or more during hospitalization (in case of acute stroke, blood pressure was measured twice after neurological stabilization) (diagnosed at admission).
    - Prescribed antihypertensive medications at the time of discharge (diagnosed at admission).
6. Diabetes (categorical): If one or more of the following apply.
    - Patients were diagnosed with diabetes in a hospital and had a history of taking oral diabetes medication or insulin treatment (including cases that stopped taking medication or insulin treatment) (known).
    - Having been told that high blood sugar should be treated at the hospital, the patient has never taken any medications (known).
    - Fasting plasma blood glucose level of 126 mg/dl or higher for more than 8 hours during hospitalization (diagnosed at admission).
    - HbA1c 6.5 or higher (diagnosed at admission).
    - A random glucose level of 200 mg/dl or higher with the following symptoms: polydipsia, polyuria, and unexplained weight loss during hospitalization (diagnosed at admission).
    - The glucose level was 200 mg/dl or more 2 h after the 75 g oral glucose tolerance test.

- Prescribed oral diabetes medications or insulin treatment at the time of discharge.
- In case of acute stroke, blood pressure measured twice after neurological stabilization.

7. Dyslipidemia (categorical): If one or more of the following apply.
   - The patient was diagnosed with dyslipidemia in a hospital and had a history of taking lipid-lowering agents (known).
   - Having been told that a high lipid level should be treated at the hospital, the patient has never taken any medications (known).
   - LDL C ≥ 100 mg/dl with coronary artery disease or coronary artery-like disease (diabetes, peripheral artery disease, abdominal aortic aneurysm, and ischemic stroke due to atherosclerosis) (diagnosed at admission).
   - LDL C ≥ 130 mg/dl with at least two risk factors (smoking, hypertension, HDL-C < 40 mg/dl, family history of premature CHD [CHD in male first-degree relative < 55 years old; CHD in female < 65 years old], age [≥ 45 years old for men or ≥ 55 years old for women] (diagnosed at admission).
   - LDL C ≥ 160 mg/dl with at least one risk factor (as above) (diagnosed at admission).
   - The test results were determined after fasting for at least 8 h after hospitalization.
8. Atrial fibrillation (categorical)
   - Having been diagnosed with atrial fibrillation in a hospital (known).
   - When atrial fibrillation is observed during hospitalization with electrocardiogram, echocardiography, or Holter monitoring (diagnosed at admission).
9. Smoker (categorical)
   - Current smoker: Have smoked in the past month.
   - Ex-smoker: Smoking cessation for more than 1 month. The period of smoking cessation was divided into fewer or more than five years.
10. Previous administration of antiplatelet/anticoagulant (categorical)
    - It is defined as a case of regular use within the last month.
    - Antiplatelet: All kinds of medications are categorized as antiplatelet agents, including aspirin, clopidogrel, and ticlopidine.
    - Anticoagulant: Warfarin, low-molecular weight heparin, and all kinds of medications were categorized as non-vitamin K antagonist oral anticoagulants including dabigatran, apixaban, rivaroxaban, etc.
11. Potential sources of cardiogenic embolism (categorical)
    - Potential sources of cardiogenic embolism definition from the TOAST classification.[2]

High- and medium-risk sources are listed as follows.

High risk sources	Medium risk sources
Mechanical prosthetic valve	Mitral valve prolapse
Mitral stenosis with atrial fibrillation	Mitral annulus calcification
Atrial fibrillation (other than lone atrial fibrillation)	Mitral stenosis without atrial fibrillation
	Left atrial turbulence (smoke)
Left atrial/atrial appendage thrombus	Atrial septal aneurysm
Sick sinus syndrome	Patent foramen ovale
Recent myocardial infarction (<4 week)	Atrial flutter
Left ventricular thrombus	Lone atrial fibrillation
Dilated cardiomyopathy	Bioprosthetic cardiac valve
Akinetic left ventricular segment	Nonbacterial thrombotic endocarditis
Atrial myxoma	Congestive heart failure
Infective endocarditis	Hypokinetic left ventricular segment
	Myocardial infarction (>4 weeks, <6 months)

Dependent variable (categorical)

- Categories: Favorable (mRS 0 and 1) and unfavorable (mRS > 2).
- The time of investigation is 3-months from the date of hospitalization.
- Outpatient clinics or telephone follow-ups; in some cases, a period of ±1 month can be given.
- Inquire about the current functional level, degree of support for daily activity, spatial extent of daily living, whether the pre-morbid standard of living is being maintained and returning to work.
- Adverse events: Investigate the stroke recurrence, cardiovascular event, or other death.

References for Supplementary Document 1.

1. Korean Stroke Society. Korean stroke registry guideline. Http://www.Strokedb.Or.Kr/bbs/index.Asp?Bbs_code=open_material. Accessed 03 august 2021. 2017
2. Adams HP, Jr., Bendixen BH, Kappelle LJ, et al. Classification of subtype of acute ischemic stroke. Definitions for use in a multicenter clinical trial. Toast. Trial of org 10172 in acute stroke treatment. *Stroke* 1993;24:35-41.

Supplementary Document S2. R code and corresponding results for this study (separate file)
