# Supplementary material for: Machine Learning-Based Three-Month Outcome Prediction in Acute Ischemic Stroke: A Single Cerebrovascular-Specialty Hospital Study in South Korea
Source: Diagnostics (Basel). 2021 Oct 15;11(10):1909. doi: 10.3390/diagnostics11101909 (PMC8534707; doi:10.3390/diagnostics11101909)
Supplement: Supplementary file 1 [file diagnostics-11-01909-s001.zip › Document S2.pdf]

R Markdown

Dougho Park

Last update: 2021 8 14

```
library(caret)
library(data.table)
library(dplyr)
library(LiblineaR)
library(e1071)
library(kernlab)
library(randomForest)
library(tidyverse)
library(xgboost)
library(smotefamily)
library(GGally)
library(MLeval)
library(moonBook)

IS <- read_csv("ischemicadm.csv")

table(is.na(IS))

##
## FALSE
## 40508

IS$Outcome <- as.factor(IS$Outcome)
IS$Sex <- as.factor(IS$Sex)
IS$Onset <- as.factor(IS$Onset)
IS$Subtype <- as.factor(IS$Subtype)
IS$PRE_TIA <- as.factor(IS$PRE_TIA)
IS$PRE_STROKE <- as.factor(IS$PRE_STROKE)
IS$PRE_PAD <- as.factor(IS$PRE_PAD)
IS$PRE_CHD <- as.factor(IS$PRE_CHD)
IS$PRE_CANCER <- as.factor(IS$PRE_CANCER)
IS$PRE_HTN <- as.factor(IS$PRE_HTN)
IS$PRE_DM <- as.factor(IS$PRE_DM)
IS$PRE_HL <- as.factor(IS$PRE_HL)
IS$SMK <- as.factor(IS$SMK)
IS$PRE_AF <- as.factor(IS$PRE_AF)
IS$PRE_ANTIPLT <- as.factor(IS$PRE_ANTIPLT)
IS$PRE_ANTICOA <- as.factor(IS$PRE_ANTICOA)
IS$PSCE_high <- as.factor(IS$PSCE_high)
IS$PSCE_medium <- as.factor(IS$PSCE_medium)
IS$Circulation <- as.factor(IS$Circulation)
```

```r
IS$Side <- as.factor(IS$Side)
IS$Thrombolysis <- as.factor(IS$Thrombolysis)

mytable(Outcome~., data=IS)

##
##          Descriptive Statistics by 'Outcome'
## ____________________________________________________
##                              favor        unfavor       p
##                             (N=745)       (N=321)
## ----------------------------------------------------
##   Sex                                               0.000
##     - Female              251 (33.7%)   156 (48.6%)
##     - Male                494 (66.3%)   165 (51.4%)
##   Onset                                             0.082
##     - Clear               605 (81.2%)   245 (76.3%)
##     - Unclear             140 (18.8%)   76 (23.7%)
##   Subtype                                           0.066
##     - CE                  79 (10.6%)    40 (12.5%)
##     - LAA                 280 (37.6%)   143 (44.5%)
##     - SVO                 289 (38.8%)   106 (33.0%)
##     - Undertermined       97 (13.0%)    32 (10.0%)
##   PRE_TIA                                           0.633
##     - 0                   731 (98.1%)   317 (98.8%)
##     - 1                   14 ( 1.9%)     4 ( 1.2%)
##   PRE_STROKE                                        0.785
##     - 0                   640 (85.9%)   270 (84.1%)
##     - 1                   17 ( 2.3%)     9 ( 2.8%)
##     - 2                   85 (11.4%)    40 (12.5%)
##     - 3                    2 ( 0.3%)     2 ( 0.6%)
##     - 4                    1 ( 0.1%)     0 ( 0.0%)
##   PRE_PAD                                           0.746
##     - 0                   741 (99.5%)   318 (99.1%)
##     - 1                    4 ( 0.5%)     3 ( 0.9%)
##   PRE_CHD                                           0.337
##     - 0                   673 (90.3%)   283 (88.2%)
##     - 1                   72 ( 9.7%)    38 (11.8%)
##   PRE_CANCER                                        0.868
##     - 0                   736 (98.8%)   316 (98.4%)
##     - 1                    9 ( 1.2%)     5 ( 1.6%)
##   PRE_HTN                                           0.224
##     - 0                   293 (39.3%)   109 (34.0%)
##     - 1                   395 (53.0%)   188 (58.6%)
##     - 2                   57 ( 7.7%)    24 ( 7.5%)
##   PRE_DM                                            0.087
##     - 0                   556 (74.6%)   223 (69.5%)
##     - 1                   159 (21.3%)   88 (27.4%)
##     - 2                   30 ( 4.0%)    10 ( 3.1%)
##   PRE_HL                                            0.650
```

```
##     - 0               278 (37.3%)   122 (38.0%)
##     - 1               222 (29.8%)   87 (27.1%)
##     - 2               245 (32.9%)   112 (34.9%)
##   SMK                                             0.002
##     - 0               499 (67.0%)   252 (78.5%)
##     - 1               242 (32.5%)   69 (21.5%)
##     - 2                 2 ( 0.3%)     0 ( 0.0%)
##     - 3                 2 ( 0.3%)     0 ( 0.0%)
##   PRE_AF                                          0.009
##     - 0               658 (88.3%)   261 (81.3%)
##     - 1               41 ( 5.5%)    30 ( 9.3%)
##     - 2               46 ( 6.2%)    30 ( 9.3%)
##   PRE_ANTIPLT                                     0.950
##     - 0               554 (74.4%)   240 (74.8%)
##     - 1               191 (25.6%)   81 (25.2%)
##   PRE_ANTICOA                                     0.170
##     - 0               726 (97.4%)   307 (95.6%)
##     - 1               19 ( 2.6%)    14 ( 4.4%)
##   PSCE_high                                       1.000
##     - 0               725 (97.3%)   313 (97.5%)
##     - 1               20 ( 2.7%)     8 ( 2.5%)
##   PSCE_medium                                     0.721
##     - 0               711 (95.4%)   304 (94.7%)
##     - 1               34 ( 4.6%)    17 ( 5.3%)
##   Circulation                                     0.097
##     - 1               521 (69.9%)   244 (76.0%)
##     - 2               223 (29.9%)   76 (23.7%)
##     - 3                 1 ( 0.1%)     1 ( 0.3%)
##   Side                                            0.264
##     - 1               350 (47.0%)   153 (47.7%)
##     - 2               341 (45.8%)   136 (42.4%)
##     - 3               54 ( 7.2%)    32 (10.0%)
##   Thrombolysis                                    0.000
##     - 0               673 (90.3%)   254 (79.1%)
##     - 1               13 ( 1.7%)    10 ( 3.1%)
##     - 2               38 ( 5.1%)    42 (13.1%)
##     - 3               21 ( 2.8%)    15 ( 4.7%)
##   Age                 65.8 ± 11.3   74.4 ± 11.4  0.000
##   Arrival              1.4 ±  1.6    1.2 ±  1.5  0.120
##   BMI                 24.2 ±  3.1   23.8 ±  3.5  0.060
##   AC                  85.2 ±  9.2   84.6 ± 10.2  0.310
##   NIHSS_adm            2.3 ±  3.2    6.3 ±  5.9  0.000
##   SBP                156.6 ± 27.0  159.0 ± 26.1  0.179
##   DBP                 87.6 ± 15.4   87.1 ± 16.3  0.609
##   Hb                  14.1 ±  1.7   13.4 ±  1.9  0.000
##   PLT                238.3 ± 66.1  231.5 ± 68.6  0.125
##   TC                 191.8 ± 46.5  188.6 ± 48.4  0.305
##   HDL                 49.3 ± 12.2   48.1 ± 11.6  0.135
##   TG                 166.4 ± 131.4 141.2 ± 127.2 0.004
```

```
## LDL                109.4 ± 40.6  113.0 ± 42.6  0.189
## RanGlu             140.8 ± 57.1  149.7 ± 62.6  0.028
## BUN                 16.4 ±  5.4   17.7 ±  7.6  0.007
## Cr                   0.9 ±  0.3    0.9 ±  0.4  0.389
## HbA1C                6.2 ±  1.2    6.4 ±  1.4  0.077
## ---------------------------------------------------
```

```
predata<-IS
nearZeroVar(predata)
```

```
## [1]  5  7  9 16 17 18
```

```
predata$PRE_TIA=NULL
predata$PRE_PAD=NULL
predata$PRE_CANCER=NULL
predata$PRE_ANTICOA=NULL
predata$PSCE_high=NULL
predata$PSCE_medium=NULL

findCorrelation(cor(predata[,16:32]), cutoff = .90)
```

```
## integer(0)
```

```
ggpairs(predata[,16:32], lower=list(continuous="smooth"))
```

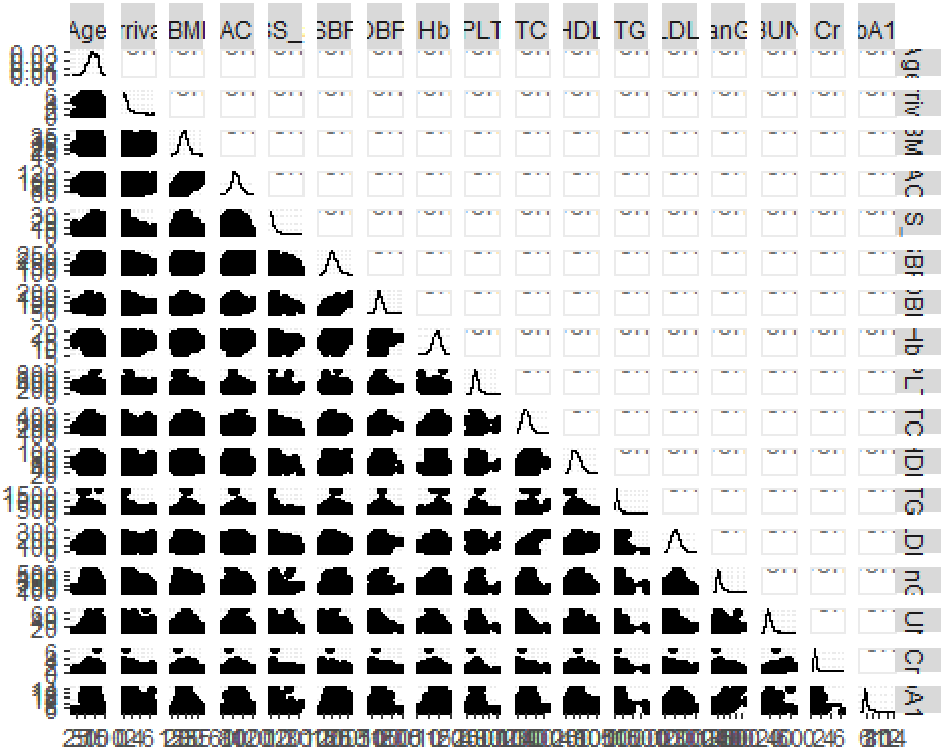

```
st_model<-preProcess(predata[,16:32], method=c("center","scale"))
data<-predict(st_model, predata)
```

```
data=as.data.frame(data)

ohe_feats = c('Sex','Onset','Subtype','PRE_STROKE','PRE_CHD','PRE_HTN','PRE_D
M','PRE_HL','SMK','PRE_AF','PRE_ANTIPLT','Circulation','Side','Thrombolysis')

dummies = dummyVars(~ Sex+Onset+Subtype+PRE_STROKE+PRE_CHD+PRE_HTN+PRE_DM+PRE
_HL+SMK+PRE_AF+PRE_ANTIPLT+Circulation+Side+Thrombolysis, data = data)

df_ohe <- as.data.frame(predict(dummies, newdata = data))
df_combined <- cbind(data[,-c(which(colnames(data) %in% ohe_feats))],df_ohe)
dat = as.data.table(df_combined)

str(dat)

## Classes 'data.table' and 'data.frame':   1066 obs. of  61 variables:
##  $ Outcome               : Factor w/ 2 levels "favor","unfavor": 2 1 1 2 1
1 1 2 2 2 ...
##  $ Age                   : num  2.058 -1.703 0.721 -1.285 -0.199 ...
##  $ Arrival               : num  0.823 0.338 -0.398 0.188 -0.804 ...
##  $ BMI                   : num  -1.628 0.318 -0.546 0.706 -0.811 ...
##  $ AC                    : num  -1.888 -1.574 -0.319 1.251 -1.051 ...
##  $ NIHSS_adm             : num  -0.542 -0.324 -0.76 -0.324 -0.76 ...
##  $ SBP                   : num  -0.349 1.108 -1.134 -1.246 1.257 ...
##  $ DBP                   : num  -1.114 1.2439 -0.0306 -0.7316 0.2243 ...
##  $ Hb                    : num  -1.447 -0.169 0.109 -0.613 0.276 ...
##  $ PLT                   : num  0.0555 2.1472 -2.0362 -1.3191 0.7129 ...
##  $ TC                    : num  -0.634 -0.443 0.62 1.916 0.365 ...
##  $ HDL                   : num  -0.741 1.175 0.759 0.759 -1.157 ...
##  $ TG                    : num  -0.481 -0.251 -0.381 -0.175 -0.152 ...
##  $ LDL                   : num  -0.205 -0.689 0.716 2.073 0.837 ...
##  $ RanGlu                : num  -0.704 -0.263 -0.398 -0.212 0.451 ...
##  $ BUN                   : num  -0.323 -0.112 -0.762 -0.795 -0.486 ...
##  $ Cr                    : num  -0.76 0.123 0.123 -0.466 -0.171 ...
##  $ HbA1C                 : num  -0.709 0.183 -0.466 -0.547 1.805 ...
##  $ Sex.Female            : num  1 1 0 1 0 1 1 1 0 0 ...
##  $ Sex.Male              : num  0 0 1 0 1 0 0 0 1 1 ...
##  $ Onset.Clear           : num  0 1 1 0 1 1 1 1 1 1 ...
##  $ Onset.Unclear         : num  1 0 0 1 0 0 0 0 0 0 ...
##  $ Subtype.CE            : num  1 0 1 1 0 0 0 0 0 1 ...
##  $ Subtype.LAA           : num  0 0 0 0 1 1 0 1 0 0 ...
##  $ Subtype.SVO           : num  0 1 0 0 0 0 1 0 1 0 ...
##  $ Subtype.Undertermined: num  0 0 0 0 0 0 0 0 0 0 ...
##  $ PRE_STROKE.0          : num  0 0 0 1 1 1 1 1 1 1 ...
##  $ PRE_STROKE.1          : num  0 1 1 0 0 0 0 0 0 0 ...
##  $ PRE_STROKE.2          : num  1 0 0 0 0 0 0 0 0 0 ...
##  $ PRE_STROKE.3          : num  0 0 0 0 0 0 0 0 0 0 ...
##  $ PRE_STROKE.4          : num  0 0 0 0 0 0 0 0 0 0 ...
##  $ PRE_CHD.0             : num  0 1 1 1 1 1 1 1 1 1 ...
```

```
##  $ PRE_CHD.1          : num  1 0 0 0 0 0 0 0 0 0 ...
##  $ PRE_HTN.0          : num  1 0 1 1 0 0 1 0 1 1 ...
##  $ PRE_HTN.1          : num  0 1 0 0 1 1 0 1 0 0 ...
##  $ PRE_HTN.2          : num  0 0 0 0 0 0 0 0 0 0 ...
##  $ PRE_DM.0           : num  1 0 1 1 0 1 1 0 1 1 ...
##  $ PRE_DM.1           : num  0 1 0 0 1 0 0 1 0 0 ...
##  $ PRE_DM.2           : num  0 0 0 0 0 0 0 0 0 0 ...
##  $ PRE_HL.0           : num  1 0 1 1 0 1 0 0 1 1 ...
##  $ PRE_HL.1           : num  0 1 0 0 1 0 0 1 0 0 ...
##  $ PRE_HL.2           : num  0 0 0 0 0 0 1 0 0 0 ...
##  $ SMK.0              : num  1 1 1 1 1 1 1 1 0 1 ...
##  $ SMK.1              : num  0 0 0 0 0 0 0 0 1 0 ...
##  $ SMK.2              : num  0 0 0 0 0 0 0 0 0 0 ...
##  $ SMK.3              : num  0 0 0 0 0 0 0 0 0 0 ...
##  $ PRE_AF.0           : num  0 1 1 1 1 1 1 1 1 0 ...
##  $ PRE_AF.1           : num  0 0 0 0 0 0 0 0 0 0 ...
##  $ PRE_AF.2           : num  1 0 0 0 0 0 0 0 0 1 ...
##  $ PRE_ANTIPLT.0      : num  1 1 1 1 1 0 1 1 1 1 ...
##  $ PRE_ANTIPLT.1      : num  0 0 0 0 0 1 0 0 0 0 ...
##  $ Circulation.1      : num  1 1 1 1 0 0 1 1 1 1 ...
##  $ Circulation.2      : num  0 0 0 0 1 1 0 0 0 0 ...
##  $ Circulation.3      : num  0 0 0 0 0 0 0 0 0 0 ...
##  $ Side.1             : num  1 0 0 0 1 1 1 0 0 0 ...
##  $ Side.2             : num  0 1 1 0 0 0 0 1 1 0 ...
##  $ Side.3             : num  0 0 0 1 0 0 0 0 0 1 ...
##  $ Thrombolysis.0     : num  1 1 1 1 1 1 1 1 1 1 ...
##  $ Thrombolysis.1     : num  0 0 0 0 0 0 0 0 0 0 ...
##  $ Thrombolysis.2     : num  0 0 0 0 0 0 0 0 0 0 ...
##  $ Thrombolysis.3     : num  0 0 0 0 0 0 0 0 0 0 ...
##  - attr(*, ".internal.selfref")=<externalptr>
```

```
table(dat$Outcome)
```

```
##
##   favor unfavor
##     745     321
```

```
seed<-111
set.seed(seed)
ind<-sample(2,nrow(dat),replace = T,prob = c(0.7,0.3))
traindata<-dat[ind==1,]
testdata<-dat[ind==2,]


table(traindata$Outcome)
```

```
##
##   favor unfavor
##     537     232
```

```
ctrl <- trainControl(method = "repeatedcv", number = 10, repeats = 10,
                     savePredictions = TRUE, classProbs = TRUE,
                     summaryFunction = twoClassSummary)
metric <- "ROC"



xgbgrid <- expand.grid(nrounds= c(500, 1000, 1500),
                       max_depth=c(6, 8),
                       eta=c(0.1, 0.01), gamma=0,
                       colsample_bytree=0.6,
                       min_child_weight=1, subsample=0.8)

set.seed(seed)
traindata.smote <- SMOTE(traindata[,-1], traindata$Outcome ,K = 5, dup_size=
0)
traindata.smote <- traindata.smote$data
traindata.smote$class <- as.factor(traindata.smote$class)
table(traindata.smote$class)

##
## unfavor   favor
##     464     537

set.seed(seed)
rf_fit_smote1 <- train(class~., data = traindata.smote, method = "rf",
                       trControl=ctrl, metric=metric, tuneLength=10,
                       ntree=500, verbose=FALSE)
rf_fit_smote1

## Random Forest
##
## 1001 samples
##   60 predictor
##    2 classes: 'unfavor', 'favor'
##
## No pre-processing
## Resampling: Cross-Validated (10 fold, repeated 10 times)
## Summary of sample sizes: 901, 902, 901, 901, 901, 900, ...
## Resampling results across tuning parameters:
##
##   mtry  ROC        Sens       Spec
##    2    0.9199657  0.7099029  0.9306115
##    8    0.9291899  0.7987558  0.8739064
##   14    0.9262879  0.8188205  0.8567610
##   21    0.9214709  0.8213922  0.8455556
##   27    0.9185057  0.8213737  0.8412928
##   34    0.9158942  0.8201064  0.8381272
##   40    0.9134774  0.8186031  0.8349686
```

```
##   47    0.9103823  0.8179371  0.8276730
##   53    0.9082809  0.8166374  0.8271069
##   60    0.9068093  0.8179278  0.8261950
##
## ROC was used to select the optimal model using the largest value.
## The final value used for the model was mtry = 8.
```

```
xsmote1 <- evalm(rf_fit_smote1)
```

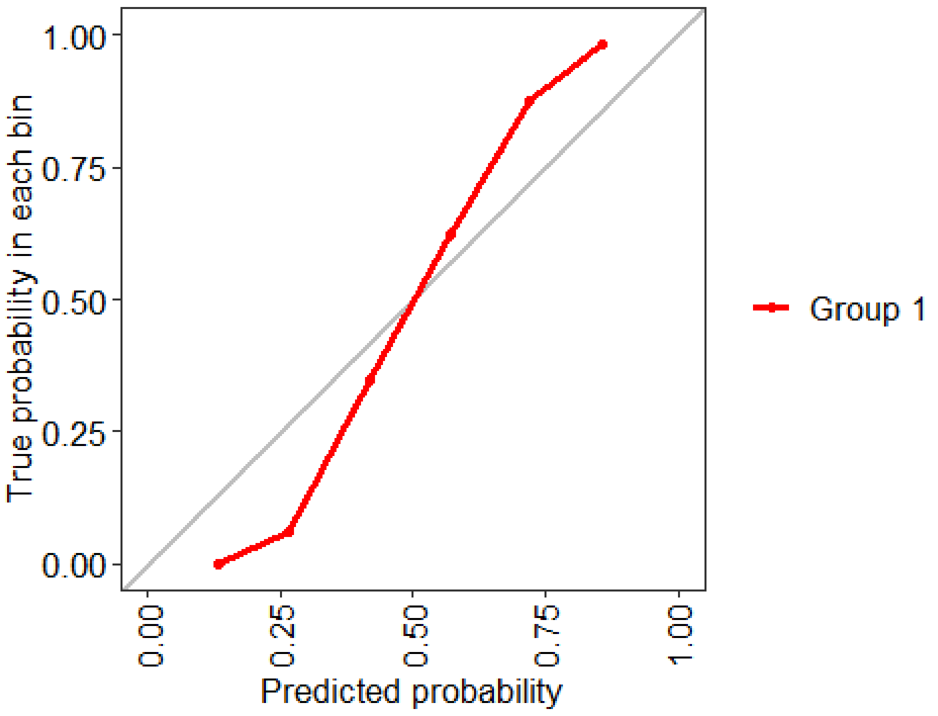
1.00
0.75
0.50
0.25
0.00
True probability in each bin
0.00
0.25
0.50
0.75
1.00
Predicted probability
Group 1

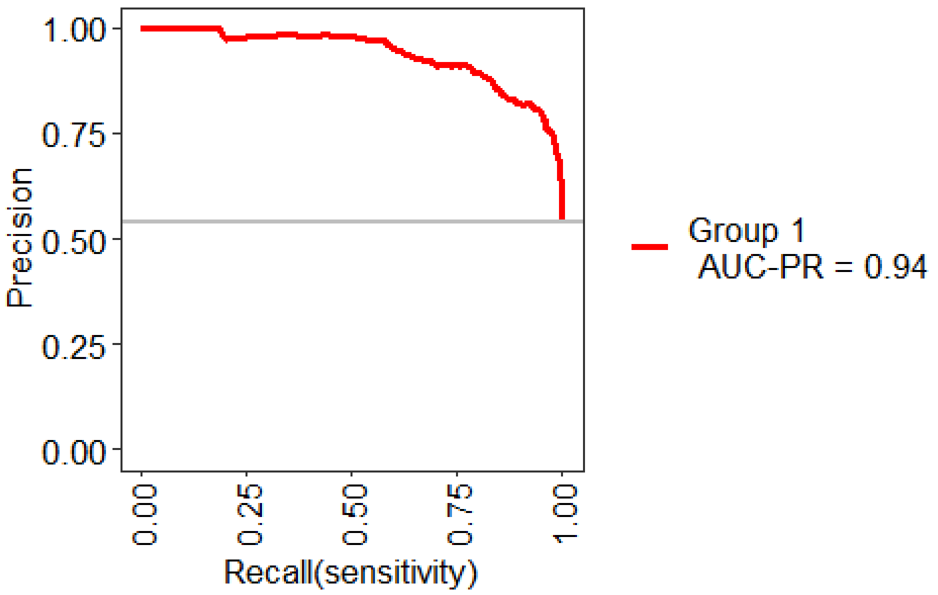
1.00
0.75
0.50
0.25
0.00
Precision
0.00
0.25
0.50
0.75
1.00
Recall(sensitivity)
Group 1
AUC-PR = 0.94

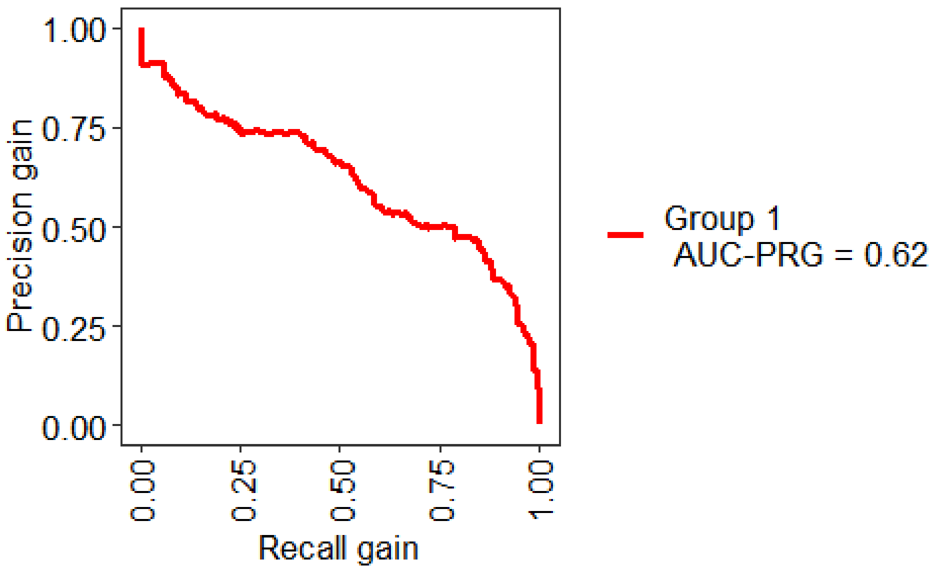

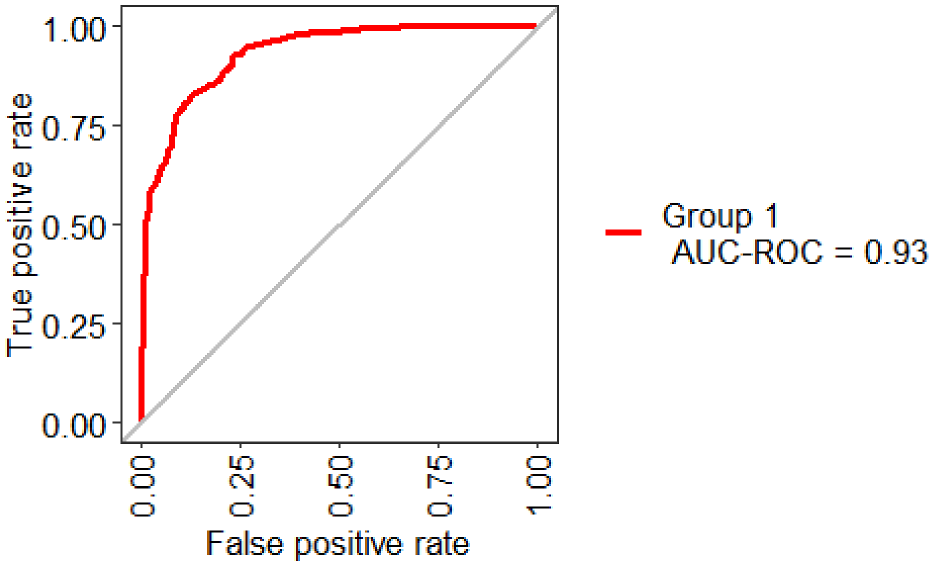


```
xsmote1$stdres
```

```
## $`Group 1`
##                   Score        CI
## SENS              0.872  0.84-0.9
## SPEC              0.797 0.76-0.83
## MCC               0.672      <NA>
## Informedness      0.669      <NA>
## PREC              0.833  0.8-0.86
## NPV               0.843 0.81-0.87
## FPR               0.203      <NA>
## F1                0.852      <NA>
## TP              468.000      <NA>
## FP               94.000      <NA>
## TN              370.000      <NA>
## FN               69.000      <NA>
## AUC-ROC           0.930 0.91-0.95
## AUC-PR            0.940      <NA>
## AUC-PRG           0.620      <NA>
```

```
test_pred_rfsmote1 <- predict(rf_fit_smote1, newdata = testdata)
confusionMatrix(test_pred_rfsmote1, testdata$Outcome)
```

```
## Confusion Matrix and Statistics
## 
##            Reference
## Prediction favor unfavor
##    favor     183      43
##    unfavor    25      46
## 
##                Accuracy : 0.771
##                  95% CI : (0.719, 0.8176)
##     No Information Rate : 0.7003
##     P-Value [Acc > NIR] : 0.003986
## 
##                   Kappa : 0.421
## 
##  Mcnemar's Test P-Value : 0.039250
## 
##             Sensitivity : 0.8798
##             Specificity : 0.5169
##          Pos Pred Value : 0.8097
##          Neg Pred Value : 0.6479
##              Prevalence : 0.7003
##          Detection Rate : 0.6162
##    Detection Prevalence : 0.7609
##       Balanced Accuracy : 0.6983
## 
##        'Positive' Class : favor
## 
```

```
test_prob_smote1 <- predict(rf_fit_smote1, newdata = testdata, type="prob")
msmote1 = data.frame(test_prob_smote1, testdata$Outcome)
ysmote1<-evalm(msmote1)
```

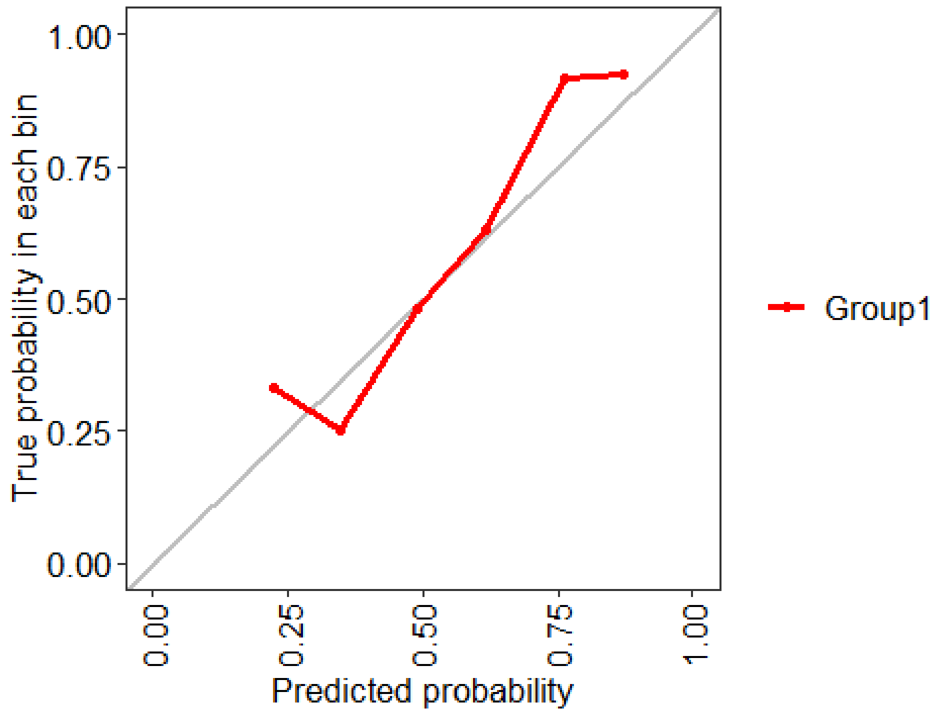

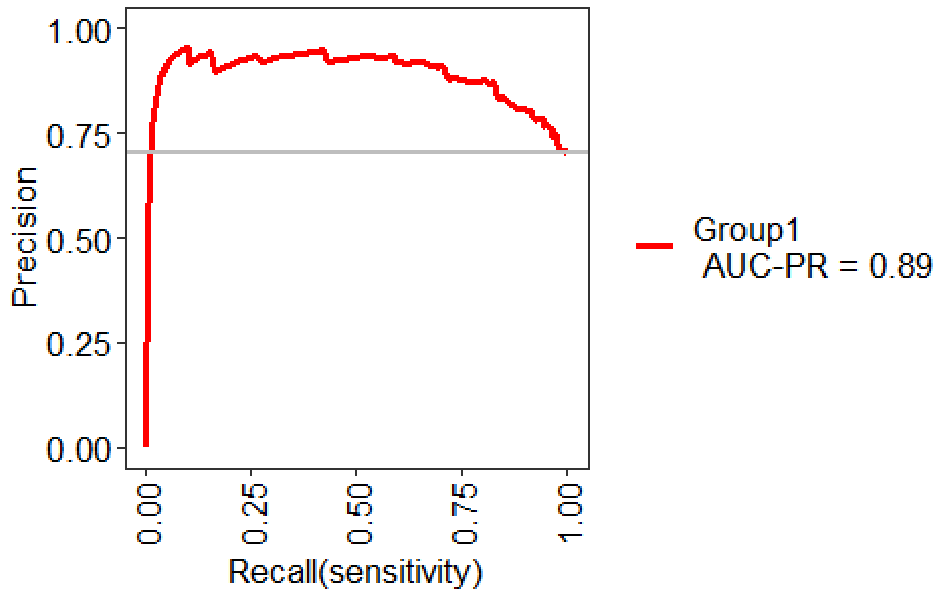

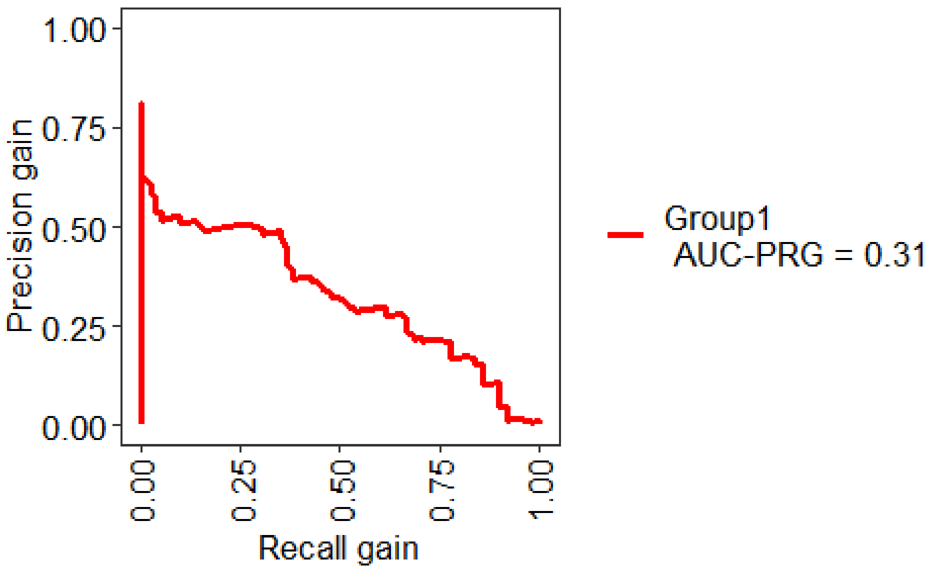

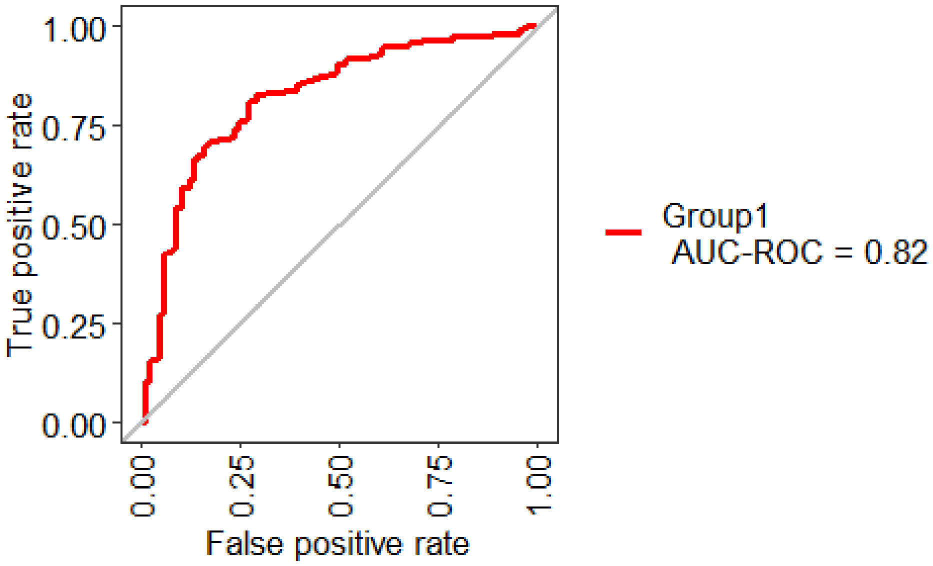


```
ysmote1$stdres
```

```
## $Group1
##                   Score        CI
## SENS              0.880 0.83-0.92
## SPEC              0.517 0.41-0.62
## MCC               0.426      <NA>
## Informedness      0.397      <NA>
## PREC              0.810 0.75-0.86
## NPV               0.648 0.53-0.75
## FPR               0.483      <NA>
## F1                0.843      <NA>
## TP              183.000      <NA>
## FP               43.000      <NA>
## TN               46.000      <NA>
## FN               25.000      <NA>
## AUC-ROC           0.820 0.77-0.87
## AUC-PR            0.890      <NA>
## AUC-PRG           0.310      <NA>
```

```
imp_smote1<-varImp(rf_fit_smote1, scale = FALSE)
imp_smote1
```

```
## rf variable importance
##
##   only 20 most important variables shown (out of 60)
##
##              Overall
## NIHSS_adm     76.830
## Age           43.356
## Hb            20.169
## Arrival       19.376
## RanGlu        18.253
## TG            17.397
## BMI           16.326
## PLT           16.285
## HDL           16.091
## LDL           15.764
## BUN           15.634
## DBP           15.322
## HbA1C         15.283
## TC            15.225
## SBP           14.816
## AC            13.752
## Cr            12.562
## SMK.1          7.084
## Sex.Male       7.063
## Sex.Female     6.413
```

```
plot(imp_smote1)
```

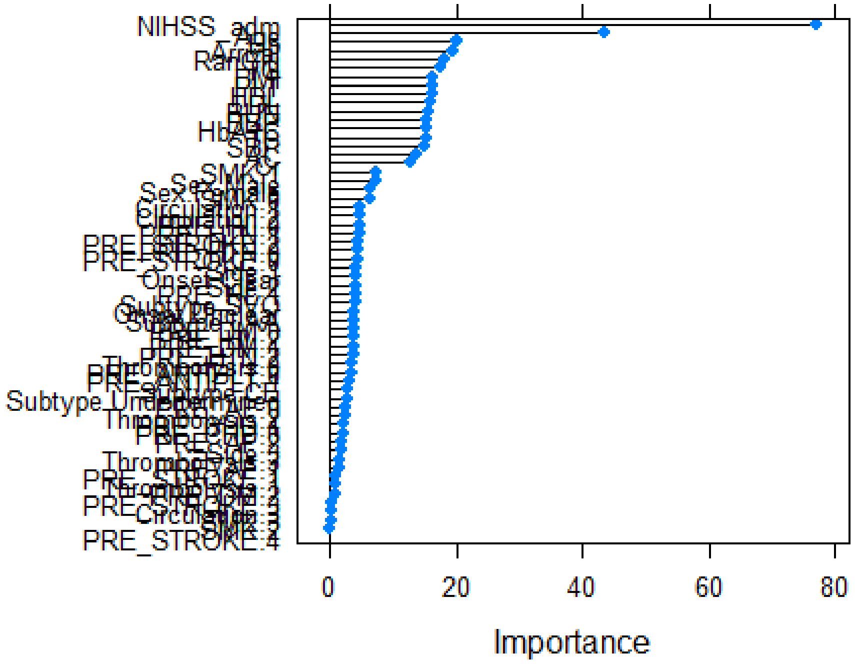


```
set.seed(seed)
rf_fit_smote2 <- train(class~., data = traindata.smote, method = "rf",
                       trControl=ctrl, metric=metric, tuneLength=10,
                       ntree=1000, verbose=FALSE)
rf_fit_smote2

## Random Forest
##
## 1001 samples
##   60 predictor
##    2 classes: 'unfavor', 'favor'
##
## No pre-processing
## Resampling: Cross-Validated (10 fold, repeated 10 times)
## Summary of sample sizes: 901, 902, 901, 901, 901, 900, ...
## Resampling results across tuning parameters:
##
##   mtry  ROC        Sens       Spec
##    2    0.9208632  0.7040981  0.9343326
##    8    0.9303975  0.8006753  0.8727952
##   14    0.9267842  0.8179371  0.8589937
##   21    0.9219385  0.8194496  0.8465059
##   27    0.9189355  0.8205042  0.8425856
##   34    0.9158507  0.8192507  0.8390391
##   40    0.9136014  0.8183673  0.8343920
##   47    0.9108900  0.8190102  0.8304857
```

```
##   53    0.9087927  0.8168501  0.8285989
##   60    0.9071169  0.8179140  0.8274983
##
## ROC was used to select the optimal model using the largest value.
## The final value used for the model was mtry = 8.
```

```
xsmote2 <- evalm(rf_fit_smote2)
```

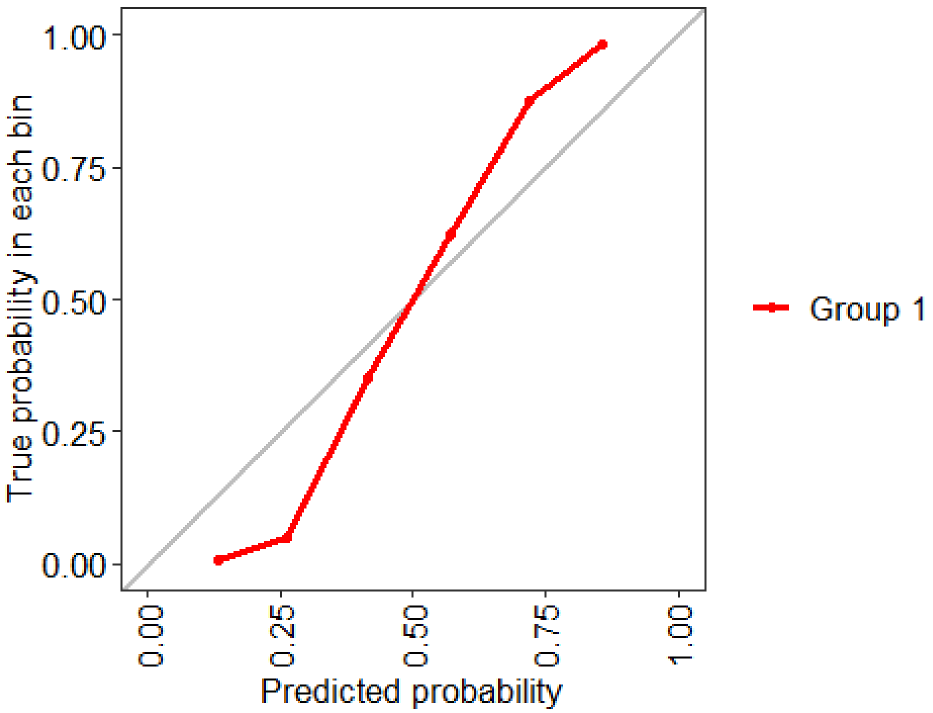
True probability in each bin
1.00
0.75
0.50
0.25
0.00
0.00
0.25
0.50
0.75
1.00
Predicted probability
Group 1

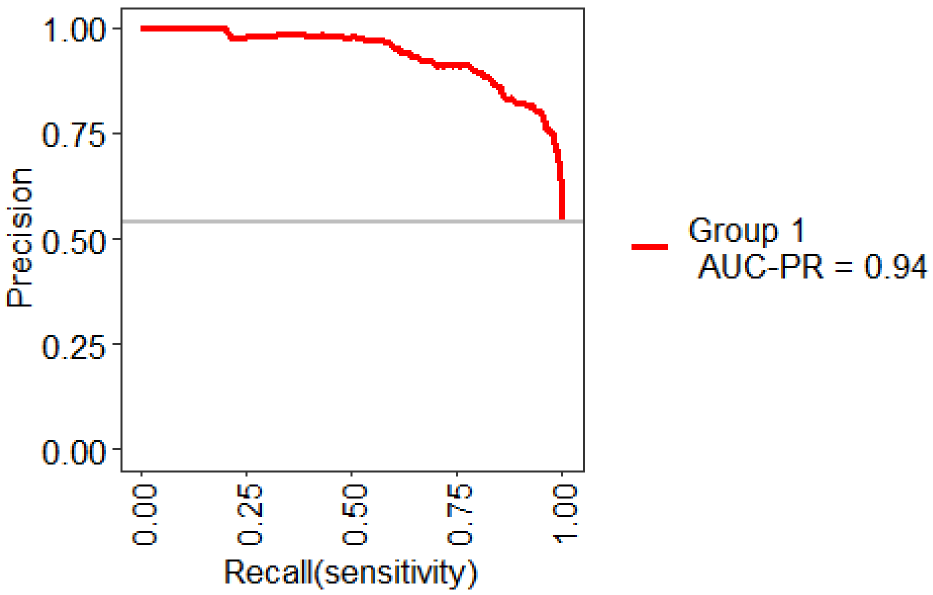
Precision
1.00
0.75
0.50
0.25
0.00
0.00
0.25
0.50
0.75
1.00
Recall(sensitivity)
Group 1
AUC-PR = 0.94

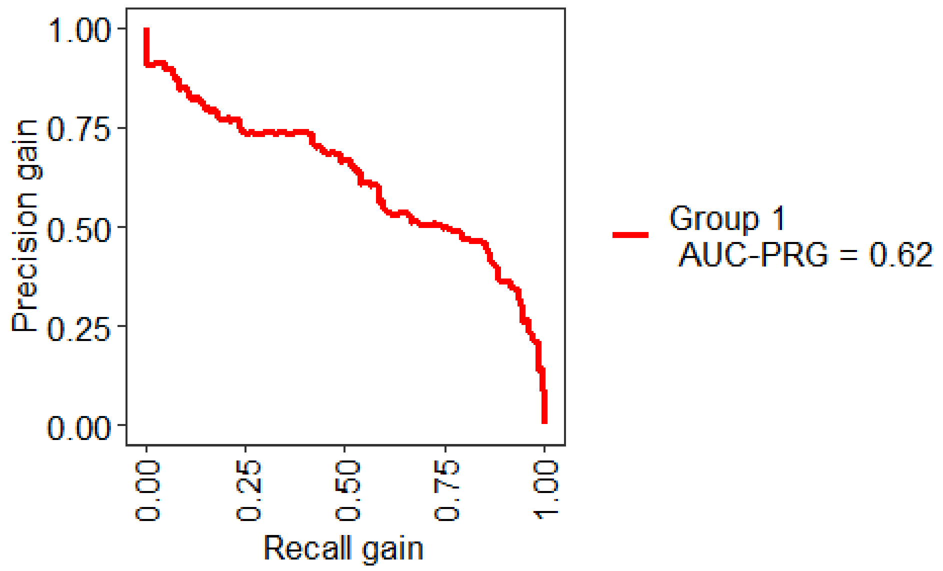

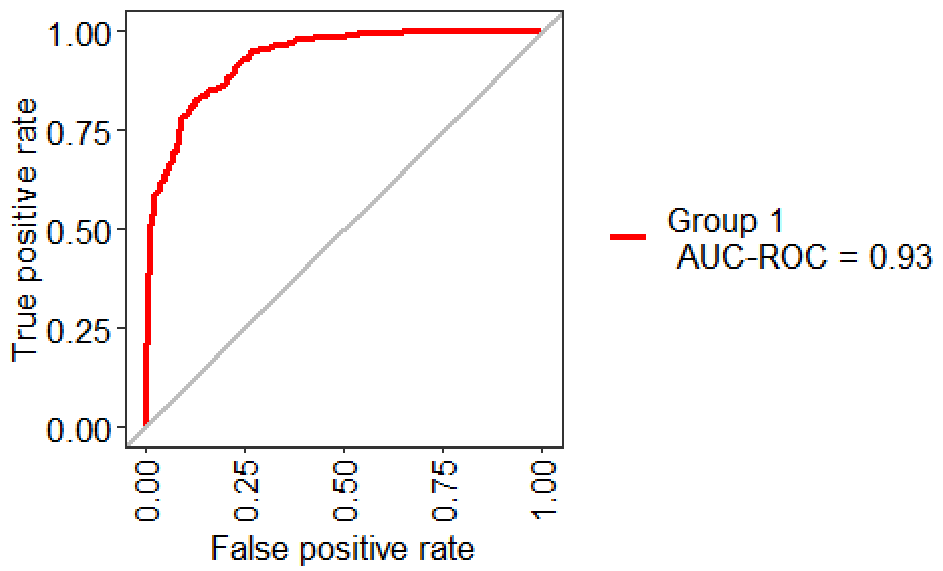


```
xsmote2$stdres
```

```
## $`Group 1`
##                   Score        CI
## SENS              0.873  0.84-0.9
## SPEC              0.797 0.76-0.83
## MCC               0.674      <NA>
## Informedness      0.671      <NA>
## PREC              0.833  0.8-0.86
## NPV               0.845 0.81-0.88
## FPR               0.203      <NA>
## F1                0.853      <NA>
## TP              469.000      <NA>
## FP               94.000      <NA>
## TN              370.000      <NA>
## FN               68.000      <NA>
## AUC-ROC           0.930 0.91-0.95
## AUC-PR            0.940      <NA>
## AUC-PRG           0.620      <NA>
```

```
test_pred_rfsmote2 <- predict(rf_fit_smote1, newdata = testdata)
confusionMatrix(test_pred_rfsmote2, testdata$Outcome)
```

```
## Confusion Matrix and Statistics
##
##           Reference
## Prediction favor unfavor
##    favor     183      43
##    unfavor    25      46
##
##                Accuracy : 0.771
##                  95% CI : (0.719, 0.8176)
##     No Information Rate : 0.7003
##     P-Value [Acc > NIR] : 0.003986
##
##                   Kappa : 0.421
##
##  Mcnemar's Test P-Value : 0.039250
##
##             Sensitivity : 0.8798
##             Specificity : 0.5169
##          Pos Pred Value : 0.8097
##          Neg Pred Value : 0.6479
##              Prevalence : 0.7003
##          Detection Rate : 0.6162
##    Detection Prevalence : 0.7609
##       Balanced Accuracy : 0.6983
##
##        'Positive' Class : favor
##
```

```
test_prob_smote2 <- predict(rf_fit_smote2, newdata = testdata, type="prob")
msmote2 = data.frame(test_prob_smote2, testdata$Outcome)
ysmote2<-evalm(msmote2)
```

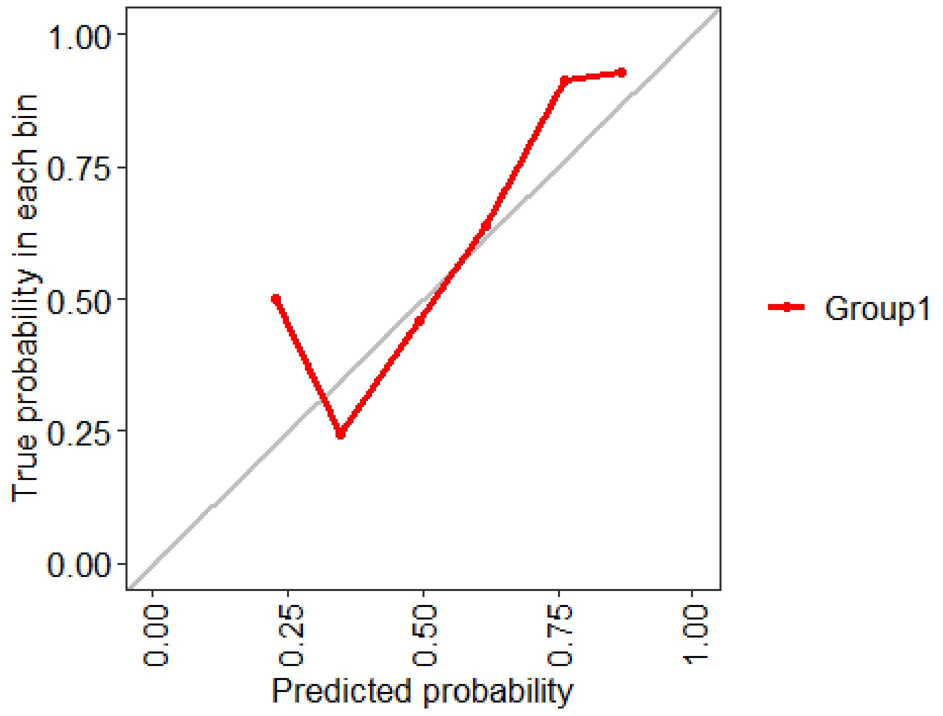

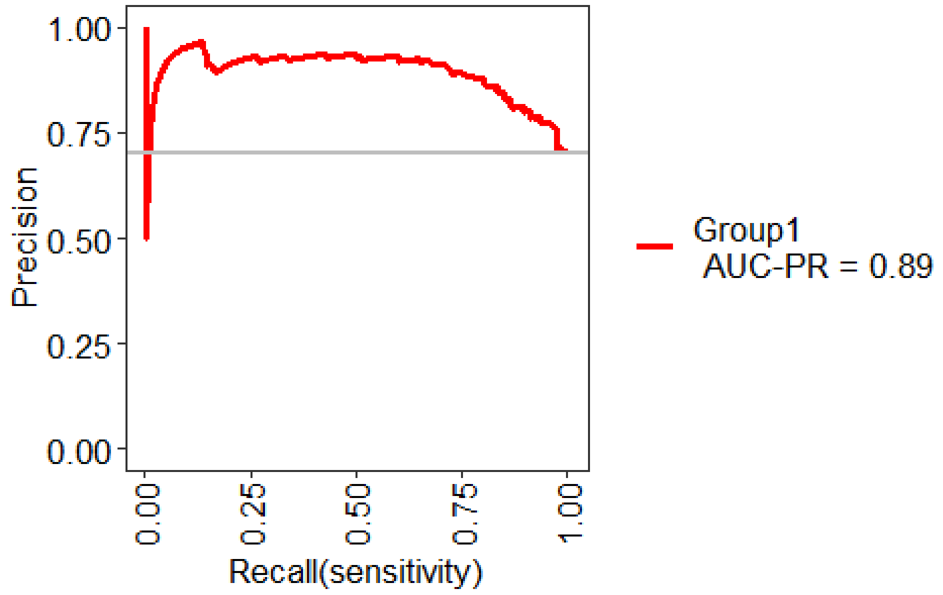

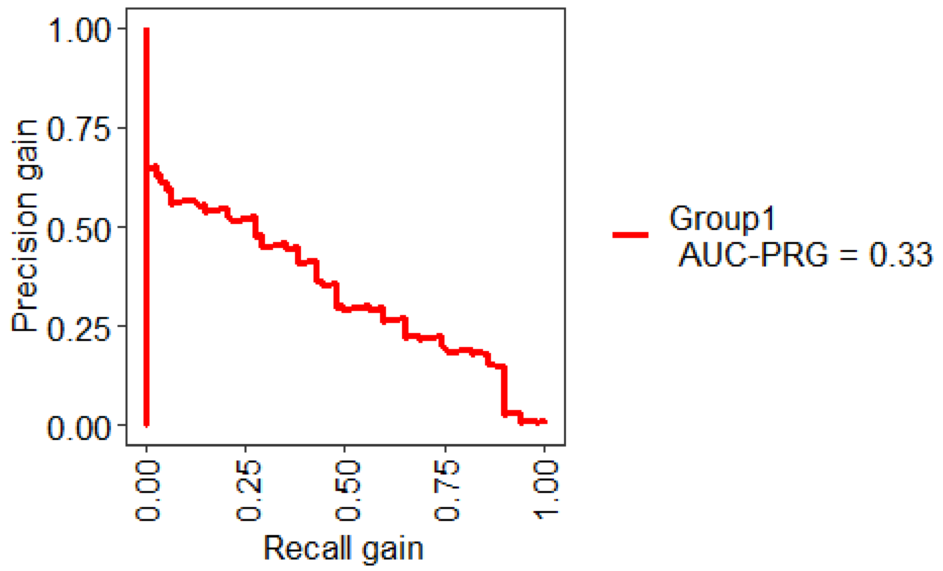

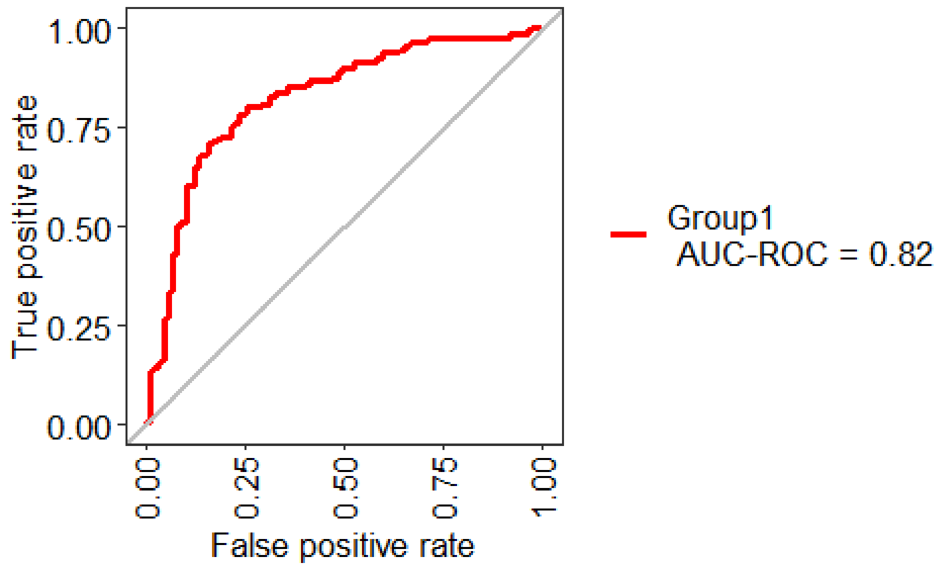


```
ysmote2$stdres
```

```
## $Group1
##                  Score        CI
## SENS             0.889 0.84-0.93
## SPEC             0.517 0.41-0.62
## MCC              0.441      <NA>
## Informedness     0.406      <NA>
## PREC             0.811 0.76-0.86
## NPV              0.667 0.55-0.77
## FPR              0.483      <NA>
## F1               0.849      <NA>
## TP             185.000      <NA>
## FP              43.000      <NA>
## TN              46.000      <NA>
## FN              23.000      <NA>
## AUC-ROC          0.820 0.77-0.87
## AUC-PR           0.890      <NA>
## AUC-PRG          0.330      <NA>
```

```
imp_smote2<-varImp(rf_fit_smote2, scale = FALSE)
imp_smote2
```

```
## rf variable importance
##
##   only 20 most important variables shown (out of 60)
##
##            Overall
## NIHSS_adm  75.783
## Age        43.039
## Hb         20.267
## Arrival    19.418
## RanGlu     18.378
## TG         18.065
## BMI        16.530
## PLT        16.351
## HDL        16.111
## DBP        15.613
## LDL        15.403
## BUN        15.326
## TC         15.267
## HbA1C      15.111
## SBP        14.939
## AC         13.899
## Cr         12.627
## SMK.1       7.401
## Sex.Male    7.320
## SMK.0       6.507
```

```
plot(imp_smote2)
```

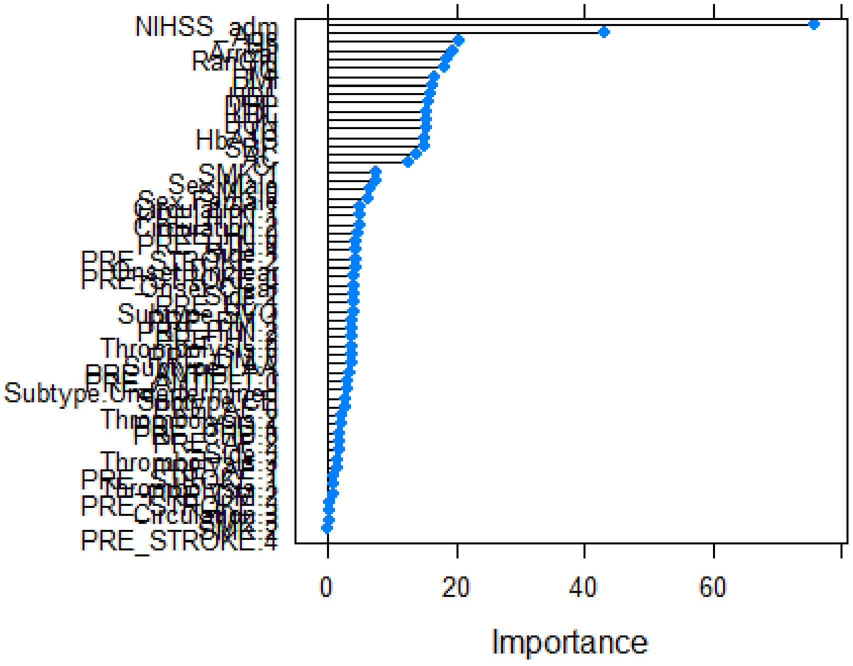


```r
set.seed(seed)
rf_fit_smote3 <- train(class~., data = traindata.smote, method = "rf",
                       trControl=ctrl, metric=metric, tuneLength=10,
                       ntree=1500, verbose=FALSE)
rf_fit_smote3

## Random Forest
##
## 1001 samples
##   60 predictor
##    2 classes: 'unfavor', 'favor'
##
## No pre-processing
## Resampling: Cross-Validated (10 fold, repeated 10 times)
## Summary of sample sizes: 901, 902, 901, 901, 901, 900, ...
## Resampling results across tuning parameters:
##
##   mtry  ROC        Sens       Spec
##    2    0.9210526  0.7062627  0.9335989
##    8    0.9301061  0.7976827  0.8746576
##   14    0.9268312  0.8175208  0.8580713
##   21    0.9222498  0.8213830  0.8478022
##   27    0.9191924  0.8209482  0.8431377
##   34    0.9157090  0.8211795  0.8386723
##   40    0.9139460  0.8183719  0.8347624
##   47    0.9109090  0.8177290  0.8310342
```

```
##   53    0.9088071  0.8155735  0.8289797
##   60    0.9072184  0.8190102  0.8271209
##
## ROC was used to select the optimal model using the largest value.
## The final value used for the model was mtry = 8.
```

```
xsmote3 <- evalm(rf_fit_smote3)
```

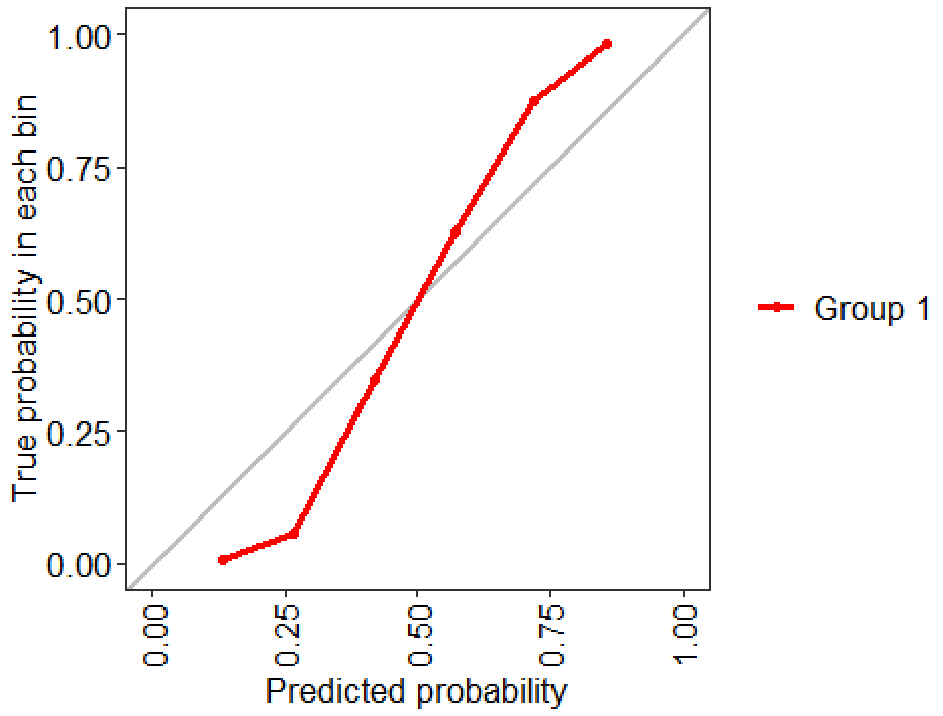
1.00
0.75
0.50
0.25
0.00
True probability in each bin
0.00
0.25
0.50
0.75
1.00
Predicted probability
Group 1

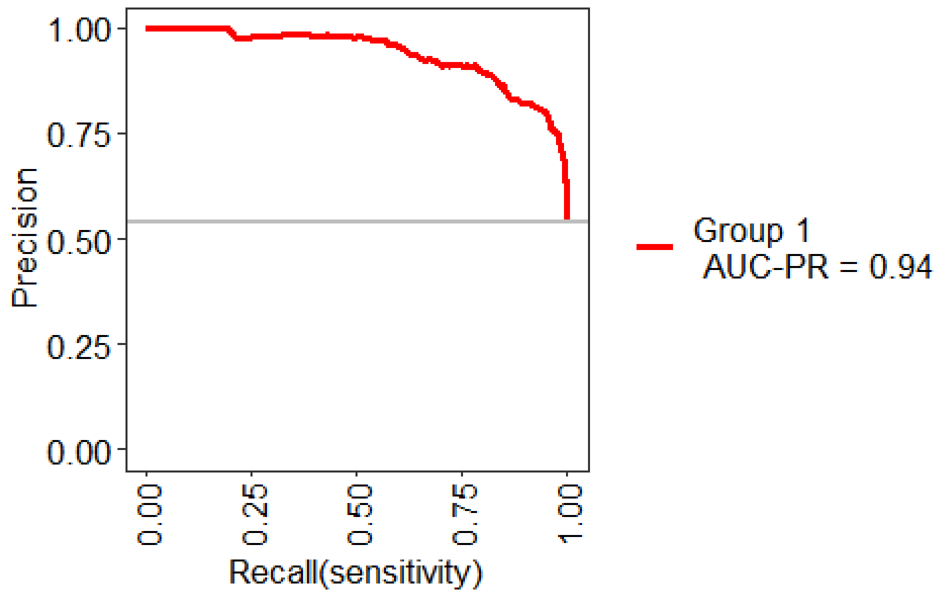
1.00
0.75
0.50
0.25
0.00
Precision
0.00
0.25
0.50
0.75
1.00
Recall(sensitivity)
Group 1
AUC-PR = 0.94

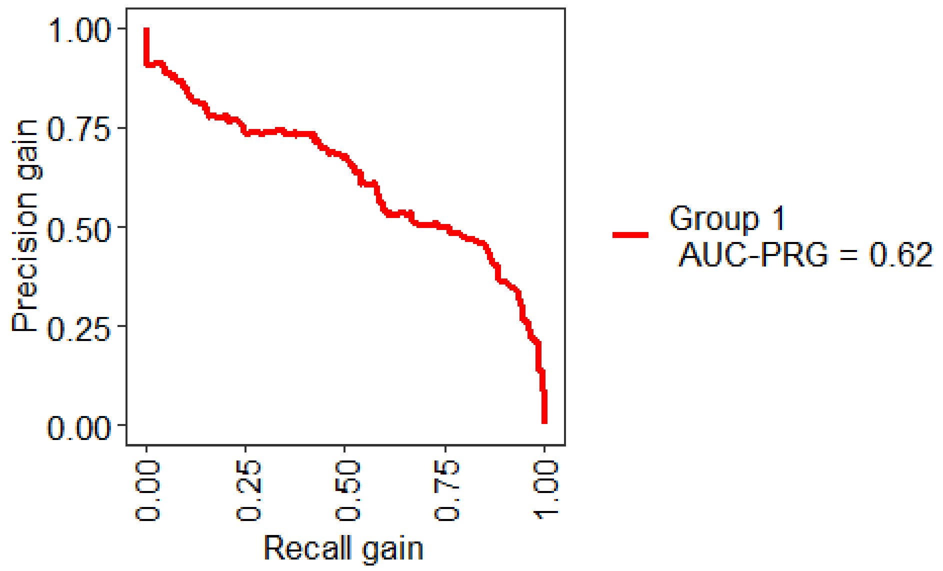

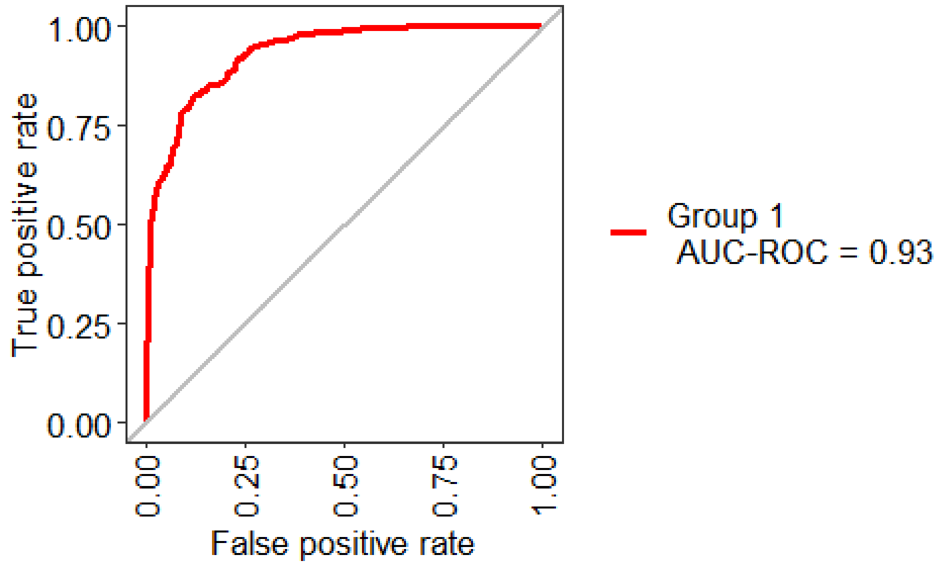


```
xsmote3$stdres
```

```
## $`Group 1`
##                   Score        CI
## SENS              0.873  0.84-0.9
## SPEC              0.797 0.76-0.83
## MCC               0.674      <NA>
## Informedness      0.671      <NA>
## PREC              0.833  0.8-0.86
## NPV               0.845 0.81-0.88
## FPR               0.203      <NA>
## F1                0.853      <NA>
## TP              469.000      <NA>
## FP               94.000      <NA>
## TN              370.000      <NA>
## FN               68.000      <NA>
## AUC-ROC           0.930 0.91-0.95
## AUC-PR            0.940      <NA>
## AUC-PRG           0.620      <NA>
```

```
test_pred_rfsmote3 <- predict(rf_fit_smote3, newdata = testdata)
confusionMatrix(test_pred_rfsmote3, testdata$Outcome)
```

```
## Confusion Matrix and Statistics
## 
##             Reference
## Prediction favor unfavor
##    favor     185      44
##    unfavor    23      45
## 
##                   Accuracy : 0.7744
##                     95% CI : (0.7226, 0.8207)
##        No Information Rate : 0.7003
##        P-Value [Acc > NIR] : 0.00266
## 
##                      Kappa : 0.4236
## 
##   Mcnemar's Test P-Value : 0.01455
## 
##                Sensitivity : 0.8894
##                Specificity : 0.5056
##             Pos Pred Value : 0.8079
##             Neg Pred Value : 0.6618
##                 Prevalence : 0.7003
##             Detection Rate : 0.6229
##       Detection Prevalence : 0.7710
##          Balanced Accuracy : 0.6975
## 
##           'Positive' Class : favor
## 
```

```
test_prob_smote3 <- predict(rf_fit_smote3, newdata = testdata, type="prob")
msmote3 = data.frame(test_prob_smote3, testdata$Outcome)
ysmote3<-evalm(msmote3)
```

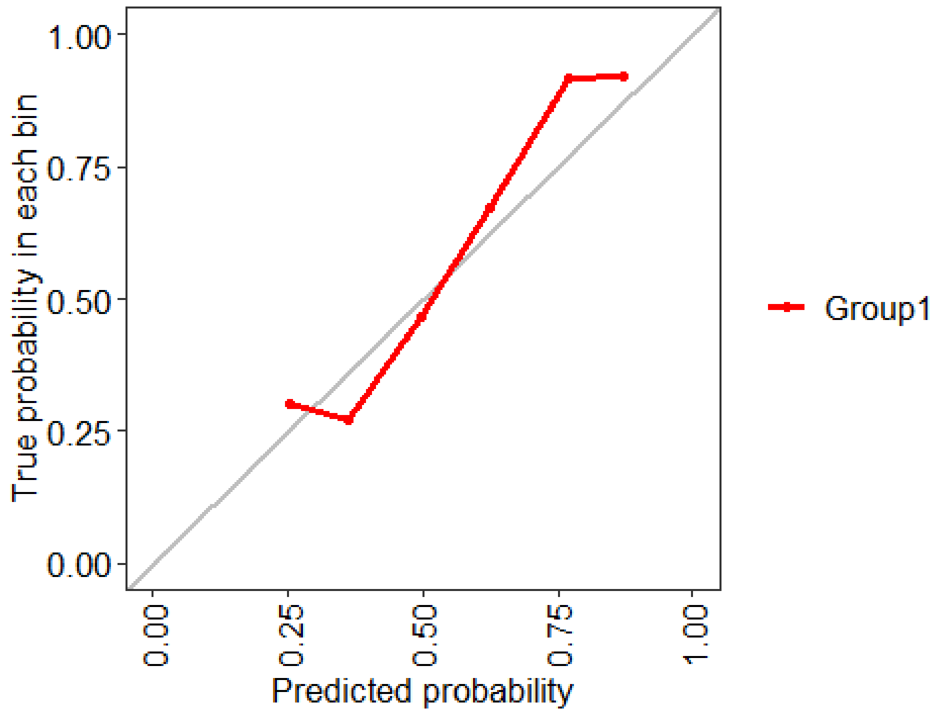

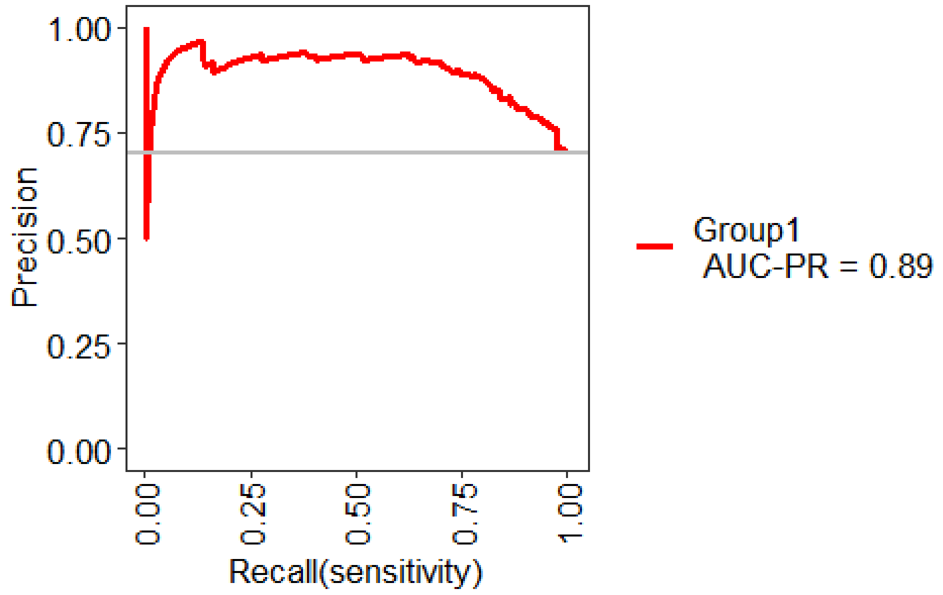

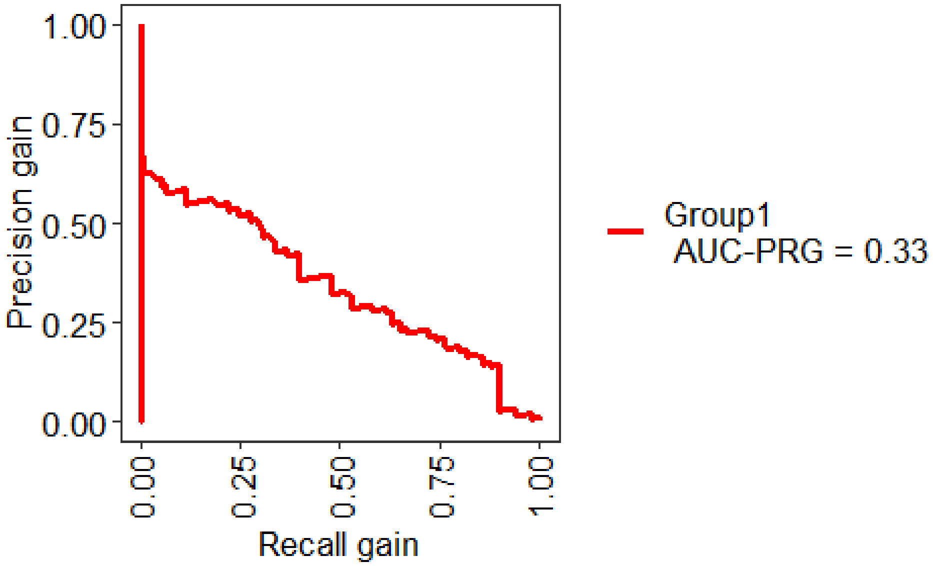

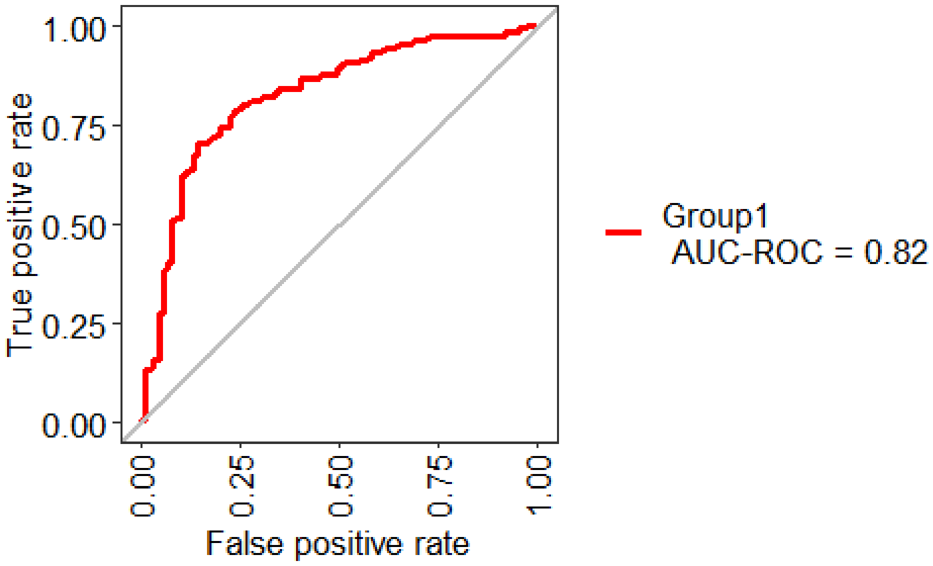


```
ysmote3$stdres
```

```
## $Group1
##                  Score        CI
## SENS             0.889 0.84-0.93
## SPEC             0.506  0.4-0.61
## MCC              0.431      <NA>
## Informedness     0.395      <NA>
## PREC             0.808 0.75-0.85
## NPV              0.662 0.54-0.76
## FPR              0.494      <NA>
## F1               0.847      <NA>
## TP             185.000      <NA>
## FP              44.000      <NA>
## TN              45.000      <NA>
## FN              23.000      <NA>
## AUC-ROC          0.820 0.77-0.87
## AUC-PR           0.890      <NA>
## AUC-PRG          0.330      <NA>
```

```
imp_smote3<-varImp(rf_fit_smote3, scale = FALSE)
imp_smote3
```

```
## rf variable importance
##
##   only 20 most important variables shown (out of 60)
##
##            Overall
## NIHSS_adm  75.137
## Age        42.740
## Hb         20.335
## Arrival    19.585
## RanGlu     18.298
## TG         18.296
## BMI        16.412
## PLT        16.406
## HDL        16.173
## DBP        15.633
## BUN        15.510
## LDL        15.370
## TC         15.251
## HbA1C      15.212
## SBP        14.997
## AC         13.836
## Cr         12.563
## SMK.1       7.375
## Sex.Male    7.140
## SMK.0       6.885
```

```
plot(imp_smote3)
```

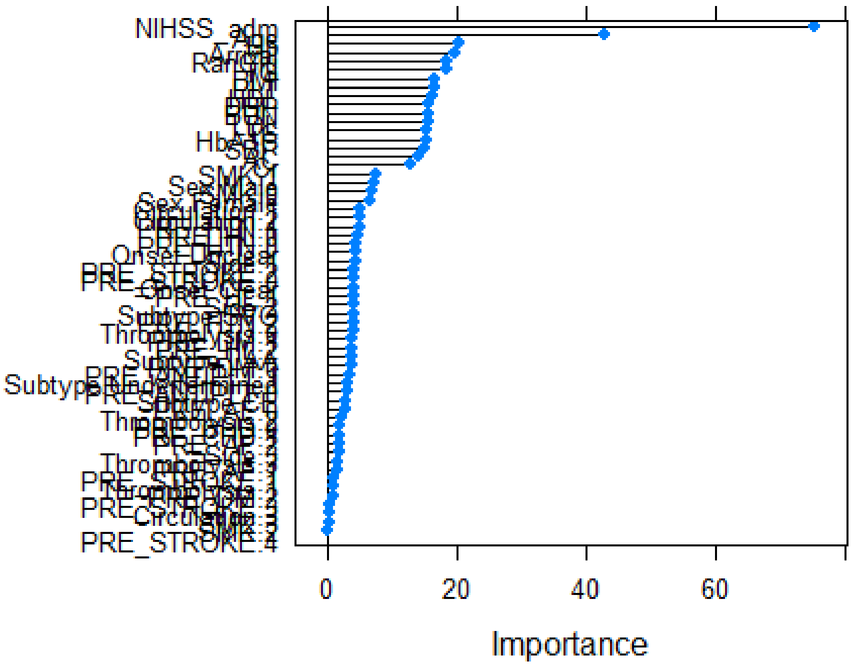


```
set.seed(seed)
xgb_fit_smote <- train(class~., data = traindata.smote, method = "xgbTree",
                       trControl=ctrl, metric=metric, tuneGrid=xgbgrid)
xgb_fit_smote

## eXtreme Gradient Boosting
##
## 1001 samples
##   60 predictor
##    2 classes: 'unfavor', 'favor'
##
## No pre-processing
## Resampling: Cross-Validated (10 fold, repeated 10 times)
## Summary of sample sizes: 901, 902, 901, 901, 901, 900, ...
## Resampling results across tuning parameters:
##
##   eta   max_depth  nrounds  ROC        Sens       Spec
##   0.01  6           500     0.9190590  0.8091119  0.8534556
##   0.01  6          1000     0.9238319  0.8168501  0.8547310
##   0.01  6          1500     0.9247173  0.8224746  0.8530643
##   0.01  8           500     0.9223459  0.8125393  0.8539902
##   0.01  8          1000     0.9257357  0.8190287  0.8573690
##   0.01  8          1500     0.9259316  0.8237835  0.8556848
##   0.10  6           500     0.9202683  0.8213691  0.8485779
##   0.10  6          1000     0.9183886  0.8181730  0.8469287
##   0.10  6          1500     0.9173454  0.8160222  0.8486059
```

```
##   0.10  8           500     0.9220397  0.8235430  0.8566212
##   0.10  8          1000     0.9205760  0.8213784  0.8529071
##   0.10  8          1500     0.9192803  0.8203053  0.8531132
##
## Tuning parameter 'gamma' was held constant at a value of 0
## Tuning
##
## Tuning parameter 'min_child_weight' was held constant at a value of 1
##
## Tuning parameter 'subsample' was held constant at a value of 0.8
## ROC was used to select the optimal model using the largest value.
## The final values used for the model were nrounds = 1500, max_depth = 8, et
a
##  = 0.01, gamma = 0, colsample_bytree = 0.6, min_child_weight = 1 and
##  subsample = 0.8.
```

```
x_xgbsmote <- evalm(xgb_fit_smote)
```

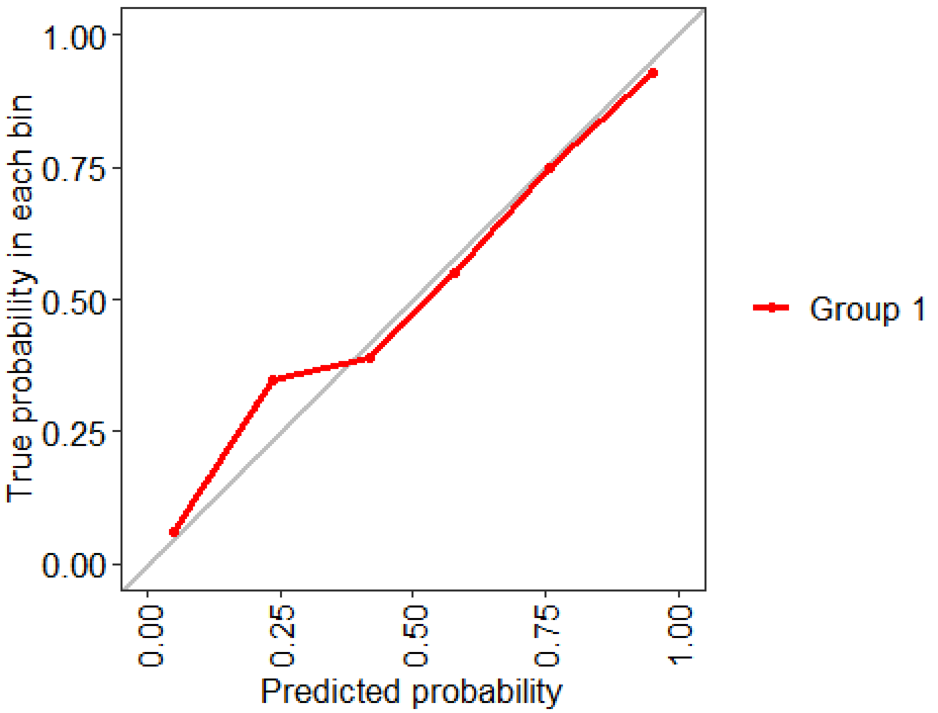

True probability in each bin
1.00
0.75
0.50
0.25
0.00
0.00
0.25
0.50
0.75
1.00
Predicted probability
Group 1

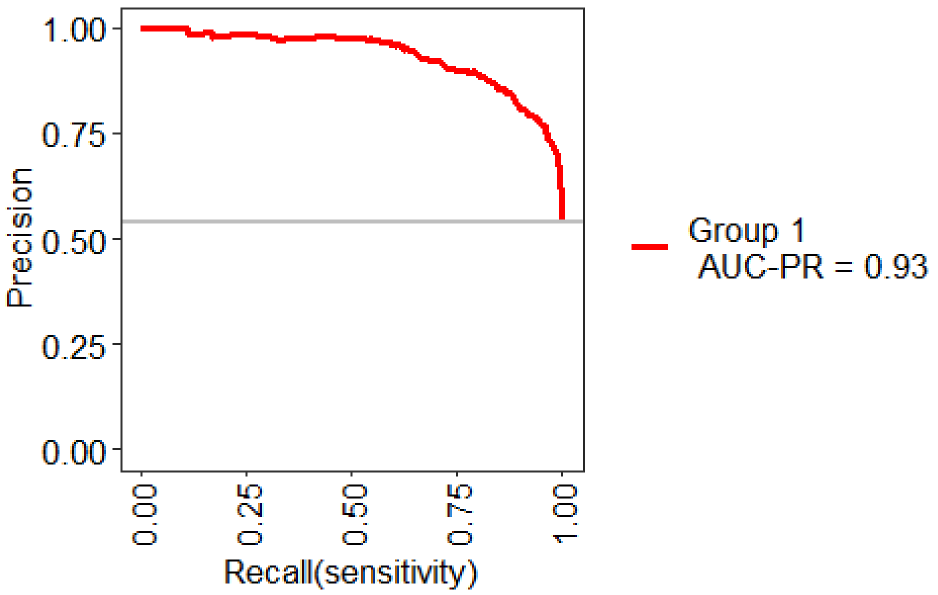

Precision
1.00
0.75
0.50
0.25
0.00
0.00
0.25
0.50
0.75
1.00
Recall(sensitivity)
Group 1
AUC-PR = 0.93

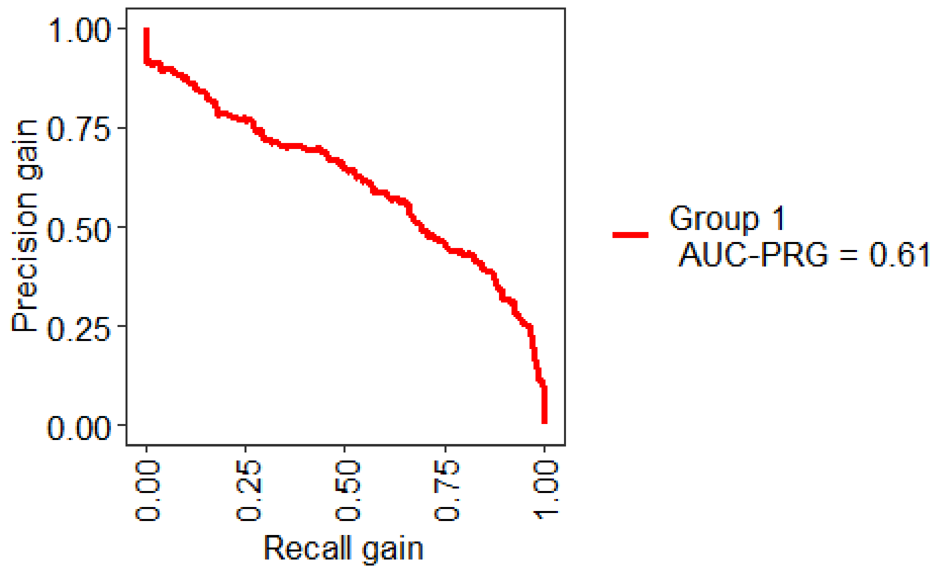

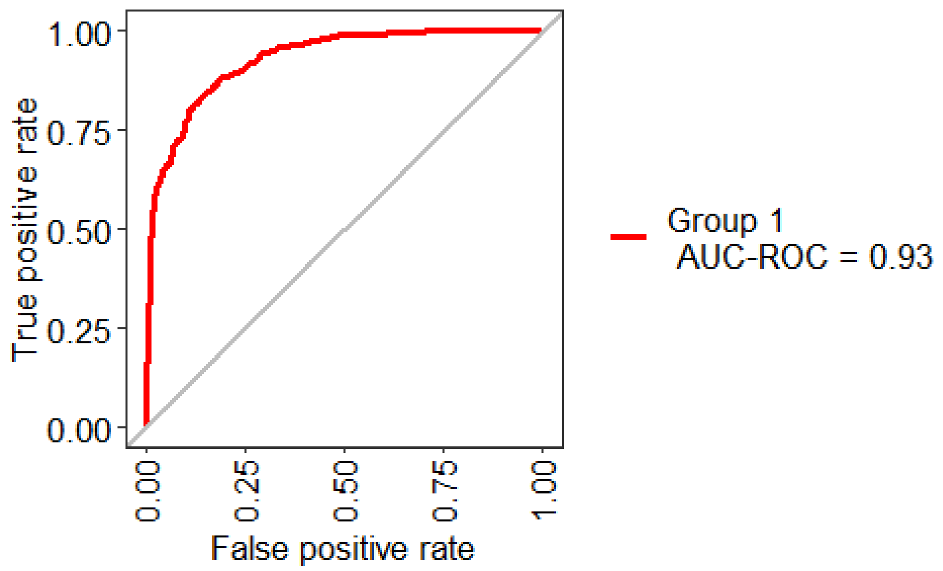


```
x_xgbsmote$stdres
```

```
## $`Group 1`
##                    Score        CI
## SENS               0.862 0.83-0.89
## SPEC               0.830 0.79-0.86
## MCC                0.692      <NA>
## Informedness       0.692      <NA>
## PREC               0.854 0.82-0.88
## NPV                0.839  0.8-0.87
## FPR                0.170      <NA>
## F1                 0.858      <NA>
## TP               463.000      <NA>
## FP                79.000      <NA>
## TN               385.000      <NA>
## FN                74.000      <NA>
## AUC-ROC            0.930 0.91-0.95
## AUC-PR             0.930      <NA>
## AUC-PRG            0.610      <NA>
```

```
test_pred_xgbsmote <- predict(xgb_fit_smote, newdata = testdata)
confusionMatrix(test_pred_xgbsmote, testdata$Outcome)
```

```
## Confusion Matrix and Statistics
##
##             Reference
## Prediction favor unfavor
##    favor     180      36
##    unfavor    28      53
##
##                Accuracy : 0.7845
##                  95% CI : (0.7333, 0.8299)
##     No Information Rate : 0.7003
##     P-Value [Acc > NIR] : 0.0007089
##
##                   Kappa : 0.4731
##
##  Mcnemar's Test P-Value : 0.3815739
##
##             Sensitivity : 0.8654
##             Specificity : 0.5955
##          Pos Pred Value : 0.8333
##          Neg Pred Value : 0.6543
##              Prevalence : 0.7003
##          Detection Rate : 0.6061
##    Detection Prevalence : 0.7273
##       Balanced Accuracy : 0.7304
##
##        'Positive' Class : favor
##
```

```
test_prob_xgbsmote <- predict(xgb_fit_smote, newdata = testdata, type="prob")
mxgbsmote = data.frame(test_prob_xgbsmote, testdata$Outcome)
yxgbsmote<-evalm(mxgbsmote)
```

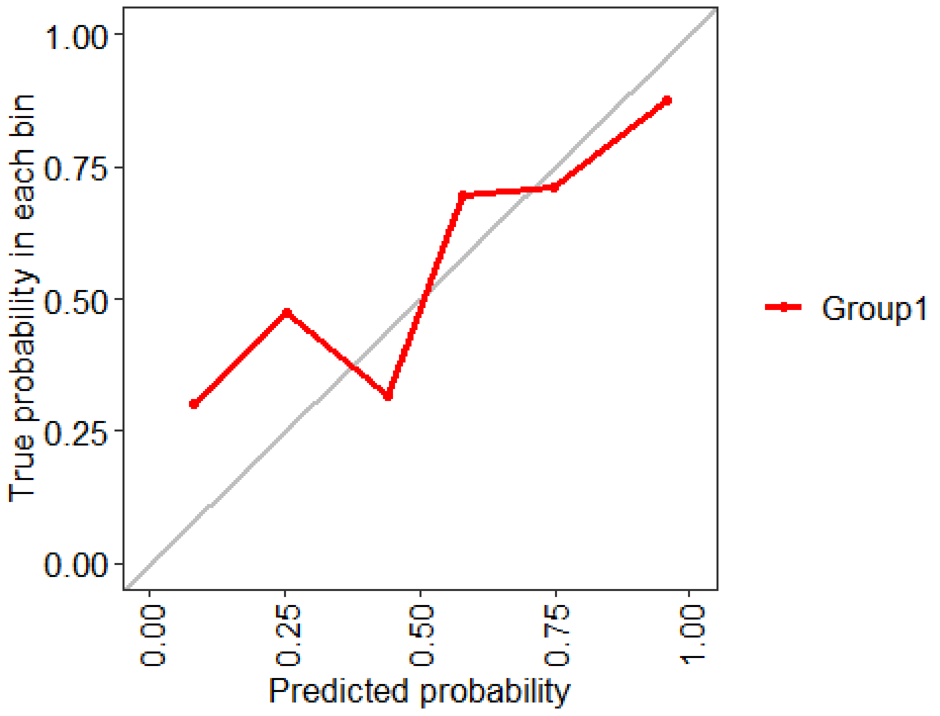

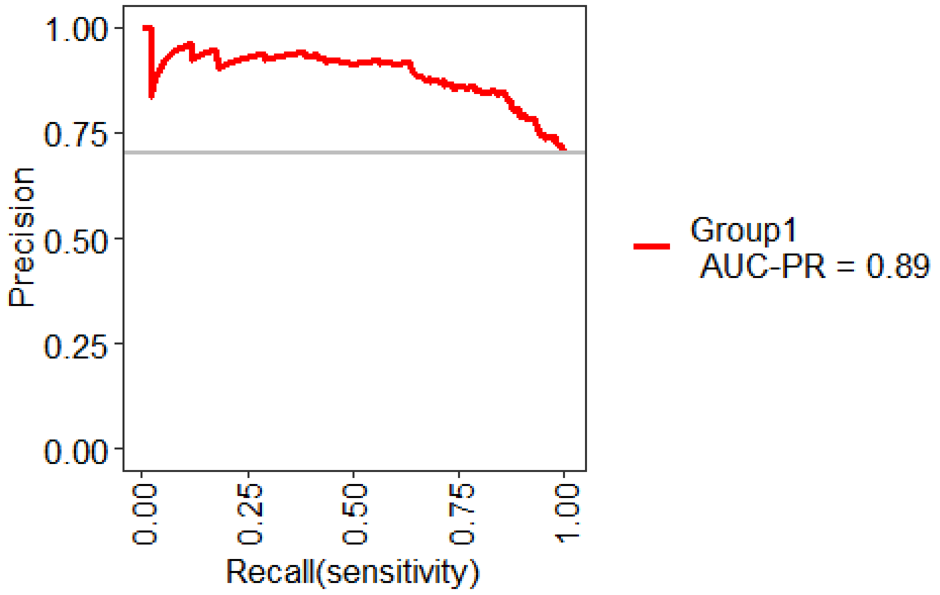

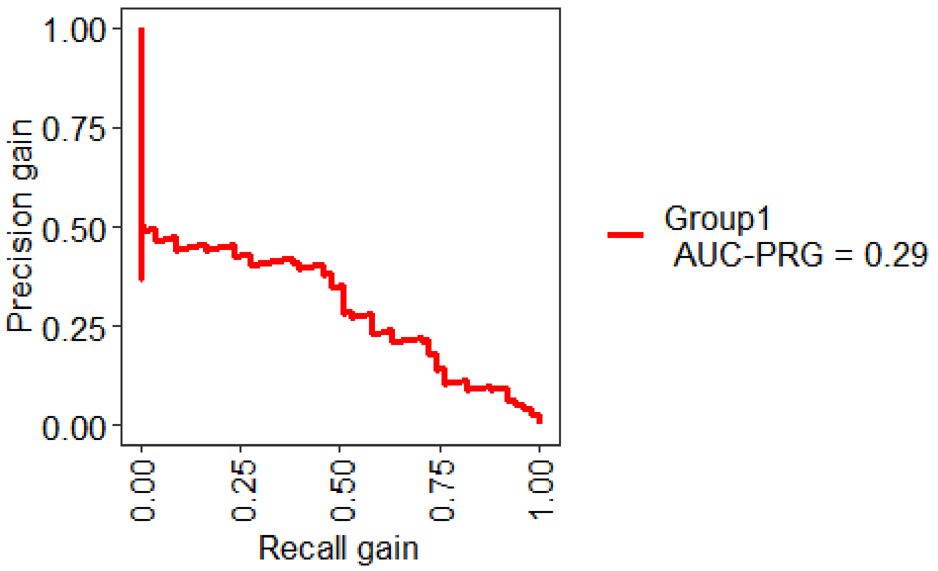

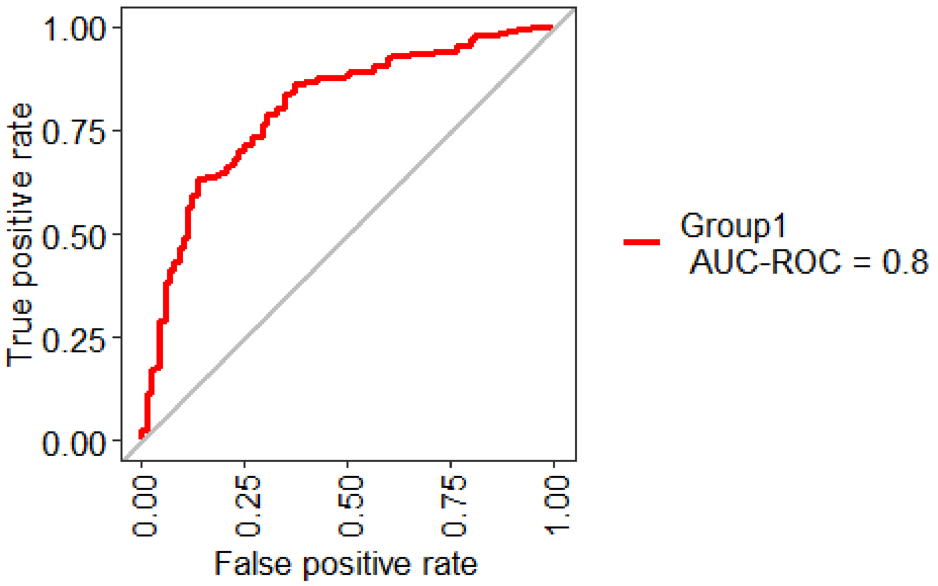


```
yxgbsmote$stdres
```

```
## $Group1
##                    Score        CI
## SENS               0.865 0.81-0.91
## SPEC               0.596 0.49-0.69
## MCC                0.474      <NA>
## Informedness       0.461      <NA>
## PREC               0.833 0.78-0.88
## NPV                0.654 0.55-0.75
## FPR                0.404      <NA>
## F1                 0.849      <NA>
## TP               180.000      <NA>
## FP                36.000      <NA>
## TN                53.000      <NA>
## FN                28.000      <NA>
## AUC-ROC            0.800 0.75-0.85
## AUC-PR             0.890      <NA>
## AUC-PRG            0.290      <NA>
```

```
imp_smote<-varImp(xgb_fit_smote, scale = FALSE)
imp_smote
```

```
## xgbTree variable importance
##
##   only 20 most important variables shown (out of 60)
##
##               Overall
## NIHSS_adm     0.17418
## Age           0.10367
## RanGlu        0.04473
## Arrival       0.04389
## PLT           0.04131
## Hb            0.03973
## DBP           0.03770
## BMI           0.03705
## LDL           0.03407
## HDL           0.03375
## TG            0.03353
## SBP           0.03286
## BUN           0.03195
## HbA1C         0.02922
## TC            0.02833
## Cr            0.02589
## AC            0.02185
## Sex.Female    0.01341
## SMK.0         0.01333
## PRE_STROKE.2 0.01109
```

```
plot(imp_smote)
```

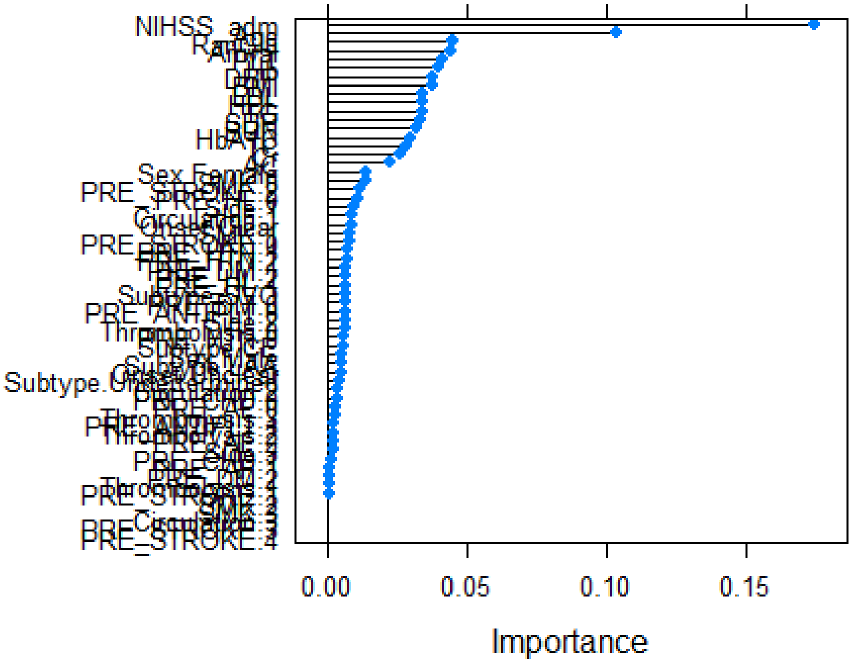


```r
set.seed(seed)
svm_fit_smote <- train(class~., data = traindata.smote, method = "svmRadial",

                       trControl=ctrl, metric=metric, tuneLength = 10)
svm_fit_smote

## Support Vector Machines with Radial Basis Function Kernel
##
## 1001 samples
##   60 predictor
##    2 classes: 'unfavor', 'favor'
##
## No pre-processing
## Resampling: Cross-Validated (10 fold, repeated 10 times)
## Summary of sample sizes: 901, 902, 901, 901, 901, 900, ...
## Resampling results across tuning parameters:
##
##   C      ROC        Sens       Spec
##     0.25  0.8185238  0.6787188  0.7965269
##     0.50  0.8424136  0.7073913  0.8099126
##     1.00  0.8660391  0.7595236  0.8175227
##     2.00  0.8865991  0.7967761  0.8236827
##     4.00  0.9026900  0.8243987  0.8307372
##     8.00  0.9177071  0.8543617  0.8457862
##    16.00  0.9242558  0.8782424  0.8485639
##    32.00  0.9237056  0.8803932  0.8450175
```

```
##    64.00  0.9235749  0.8793293  0.8442732
##   128.00  0.9235749  0.8788853  0.8453913
##
## Tuning parameter 'sigma' was held constant at a value of 0.03214894
## ROC was used to select the optimal model using the largest value.
## The final values used for the model were sigma = 0.03214894 and C = 16.
```

```
x_svmsmote <- evalm(svm_fit_smote)
```

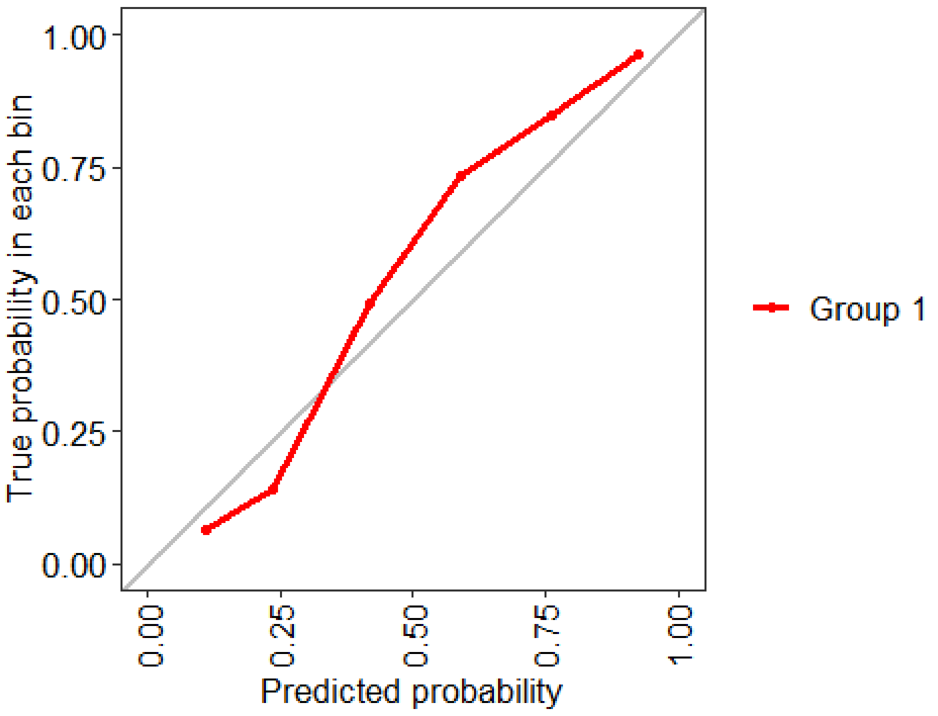

1.00
0.75
0.50
0.25
0.00
True probability in each bin
0.00
0.25
0.50
0.75
1.00
Predicted probability
Group 1

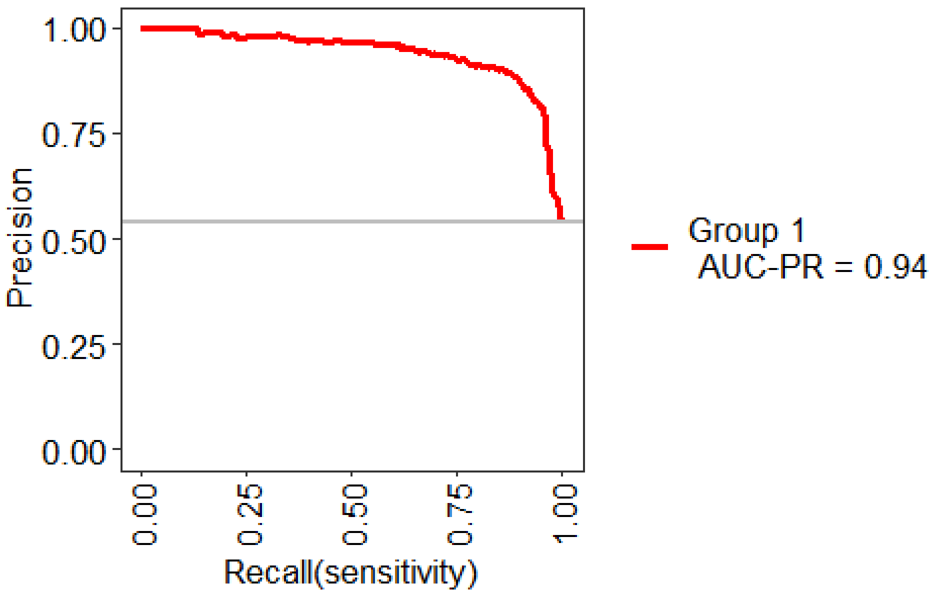

1.00
0.75
0.50
0.25
0.00
Precision
0.00
0.25
0.50
0.75
1.00
Recall(sensitivity)
Group 1
AUC-PR = 0.94

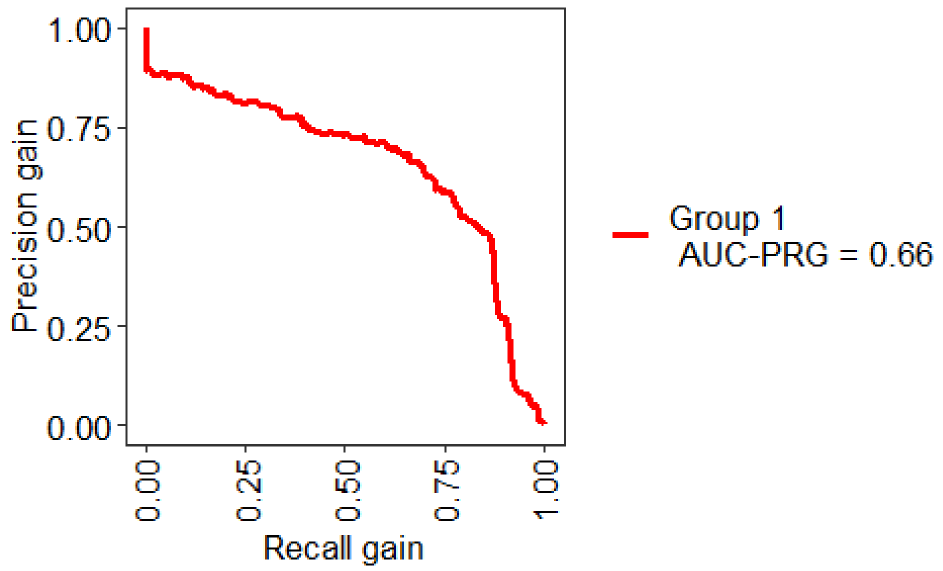

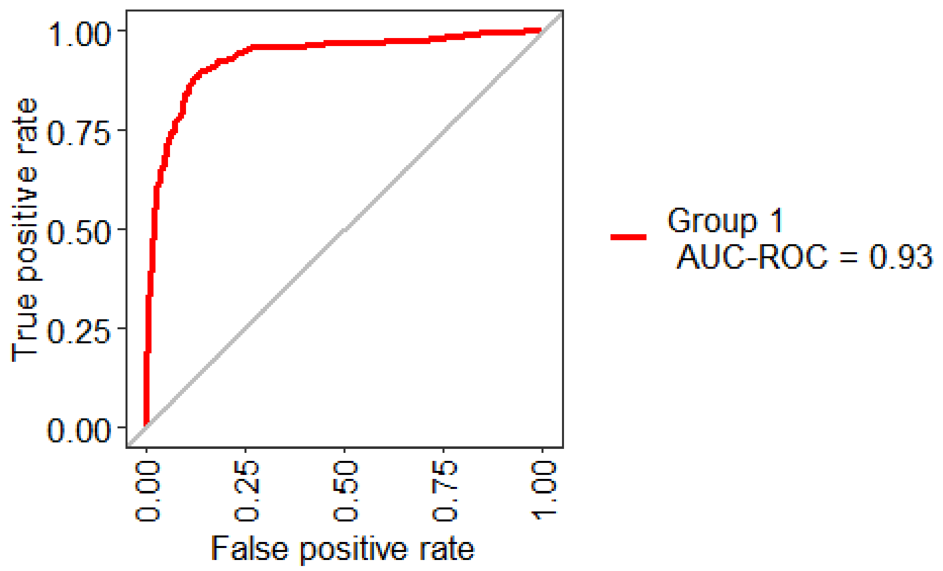


```
x_svmsmote$stdres
```

```
## $`Group 1`
##                  Score        CI
## SENS             0.851 0.82-0.88
## SPEC             0.892 0.86-0.92
## MCC              0.741      <NA>
## Informedness     0.743      <NA>
## PREC             0.901 0.87-0.92
## NPV              0.838  0.8-0.87
## FPR              0.108      <NA>
## F1               0.875      <NA>
## TP             457.000      <NA>
## FP              50.000      <NA>
## TN             414.000      <NA>
## FN              80.000      <NA>
## AUC-ROC          0.930 0.91-0.95
## AUC-PR           0.940      <NA>
## AUC-PRG          0.660      <NA>
```

```
test_pred_svmsmote <- predict(svm_fit_smote, newdata = testdata)
confusionMatrix (test_pred_svmsmote, testdata$Outcome)
```

```
## Confusion Matrix and Statistics
##
##             Reference
## Prediction favor unfavor
##    favor     176      55
##    unfavor    32      34
##
##                Accuracy : 0.7071
##                  95% CI : (0.6517, 0.7582)
##     No Information Rate : 0.7003
##     P-Value [Acc > NIR] : 0.42788
##
##                   Kappa : 0.2464
##
##  Mcnemar's Test P-Value : 0.01834
##
##             Sensitivity : 0.8462
##             Specificity : 0.3820
##          Pos Pred Value : 0.7619
##          Neg Pred Value : 0.5152
##              Prevalence : 0.7003
##          Detection Rate : 0.5926
##    Detection Prevalence : 0.7778
##       Balanced Accuracy : 0.6141
##
##        'Positive' Class : favor
##
```

```
test_prob_svmsmote <- predict(svm_fit_smote, newdata = testdata, type="prob")
msvmsmote = data.frame(test_prob_svmsmote, testdata$Outcome)
ysvmsmote<-evalm(msvmsmote)
```

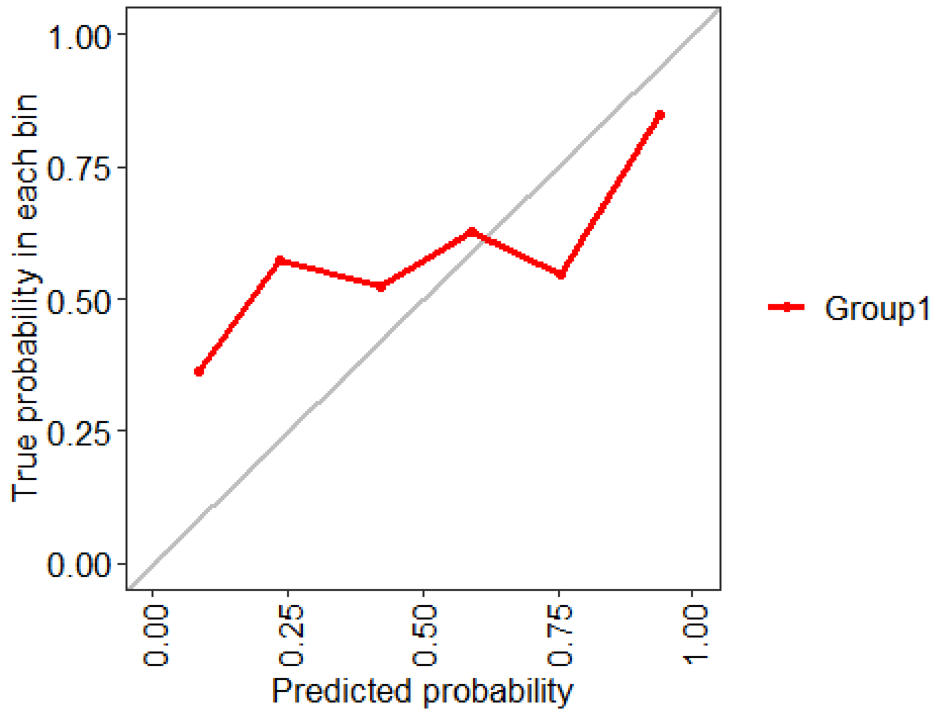

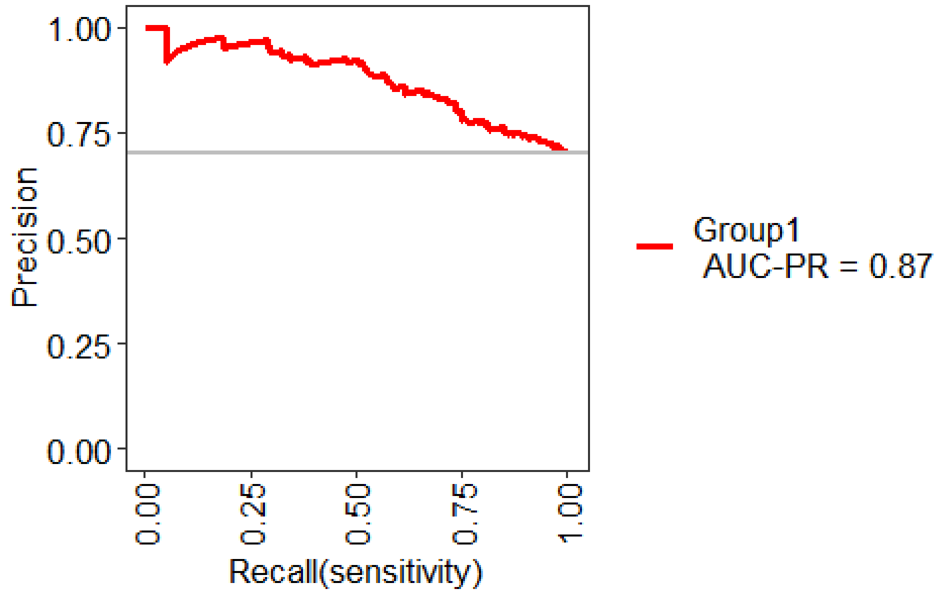

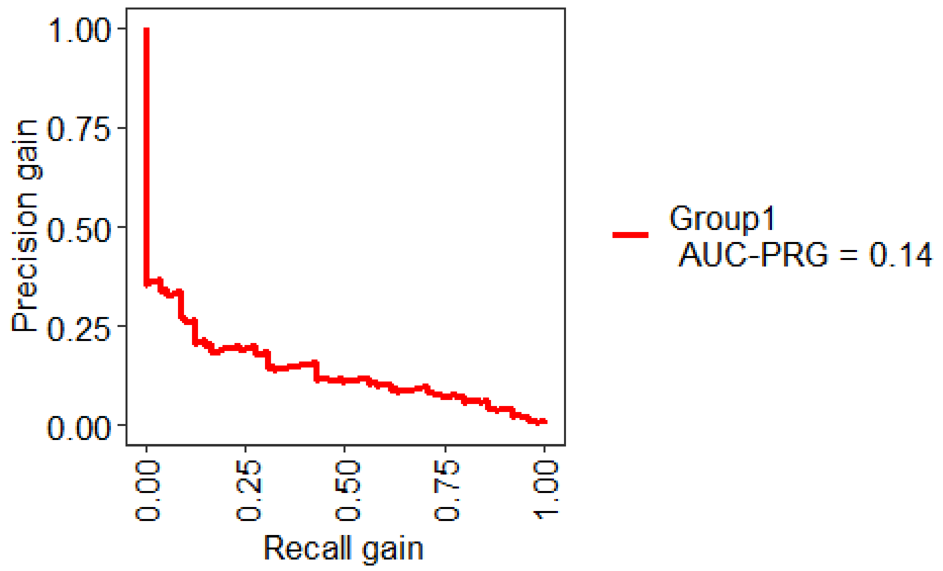

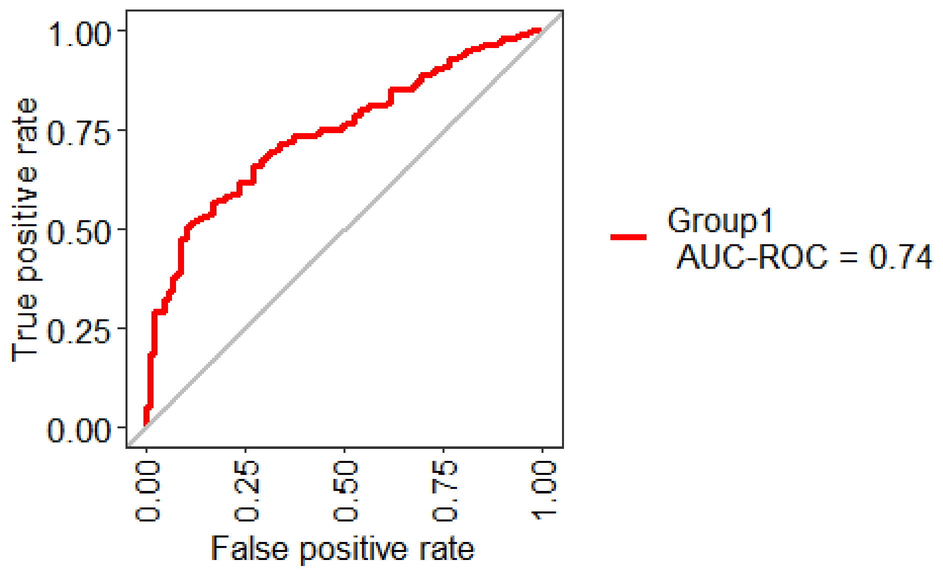


```
ysvmsmote$stdres
```

```
## $Group1
##                  Score        CI
## SENS             0.846 0.79-0.89
## SPEC             0.382 0.29-0.49
## MCC              0.251      <NA>
## Informedness     0.228      <NA>
## PREC             0.762  0.7-0.81
## NPV              0.515  0.4-0.63
## FPR              0.618      <NA>
## F1               0.802      <NA>
## TP             176.000      <NA>
## FP              55.000      <NA>
## TN              34.000      <NA>
## FN              32.000      <NA>
## AUC-ROC          0.740  0.68-0.8
## AUC-PR           0.870      <NA>
## AUC-PRG          0.140      <NA>
```

```
set.seed(seed)
knn_fit_smote <- train(class~., data = traindata.smote, method = "knn",
                       trControl=ctrl, metric=metric, tuneLength = 50)
knn_fit_smote
```

```
## k-Nearest Neighbors
##
## 1001 samples
##   60 predictor
##    2 classes: 'unfavor', 'favor'
##
## No pre-processing
## Resampling: Cross-Validated (10 fold, repeated 10 times)
## Summary of sample sizes: 901, 902, 901, 901, 901, 900, ...
## Resampling results across tuning parameters:
##
##   k    ROC        Sens       Spec
##     5  0.7891124  0.8774607  0.5530713
##     7  0.7718016  0.8621832  0.5428546
##     9  0.7724889  0.8442183  0.5392488
##    11  0.7670486  0.8276087  0.5433508
##    13  0.7621251  0.8276226  0.5355486
##    15  0.7605204  0.8295282  0.5295982
##    17  0.7574139  0.8157308  0.5320056
##    19  0.7579700  0.8081684  0.5351468
##    21  0.7592525  0.8077521  0.5370266
##    23  0.7582354  0.8042923  0.5396191
##    25  0.7597226  0.8086309  0.5312823
##    27  0.7586580  0.8058187  0.5303529
##    29  0.7566641  0.7995606  0.5295528
##    31  0.7549773  0.7956707  0.5351677
```

```
##    33  0.7551995  0.7989269  0.5330992
##    35  0.7548698  0.7972248  0.5355486
##    37  0.7536818  0.7991397  0.5355241
##    39  0.7517316  0.7987373  0.5332879
##    41  0.7505306  0.7946438  0.5301118
##    43  0.7491852  0.7892553  0.5342453
##    45  0.7502842  0.7890333  0.5357372
##    47  0.7501962  0.7912165  0.5349965
##    49  0.7501505  0.7922664  0.5355416
##    51  0.7495641  0.7952868  0.5336827
##    53  0.7495018  0.7937558  0.5331202
##    55  0.7480027  0.7924699  0.5366562
##    57  0.7472201  0.7888205  0.5349860
##    59  0.7463208  0.7871045  0.5374319
##    61  0.7458379  0.7845051  0.5374528
##    63  0.7466711  0.7814847  0.5383857
##    65  0.7465251  0.7780250  0.5391335
##    67  0.7463538  0.7799722  0.5376625
##    69  0.7453873  0.7765541  0.5423061
##    71  0.7446172  0.7745930  0.5437945
##    73  0.7449039  0.7741906  0.5454507
##    75  0.7433888  0.7731082  0.5359888
##    77  0.7428019  0.7705227  0.5415828
##    79  0.7419135  0.7683765  0.5408386
##    81  0.7404245  0.7655550  0.5456709
##    83  0.7390456  0.7649075  0.5477149
##    85  0.7388668  0.7627567  0.5475542
##    87  0.7387461  0.7620907  0.5501398
##    89  0.7396144  0.7631776  0.5531516
##    91  0.7399839  0.7607956  0.5557512
##    93  0.7404950  0.7569149  0.5585465
##    95  0.7401592  0.7532331  0.5596576
##    97  0.7403218  0.7545375  0.5628302
##    99  0.7406606  0.7549722  0.5642837
##   101  0.7409364  0.7543293  0.5646471
##   103  0.7411981  0.7543386  0.5683892
## 
## ROC was used to select the optimal model using the largest value.
## The final value used for the model was k = 5.
```

```
x_knnsmote <- evalm(knn_fit_smote)
```

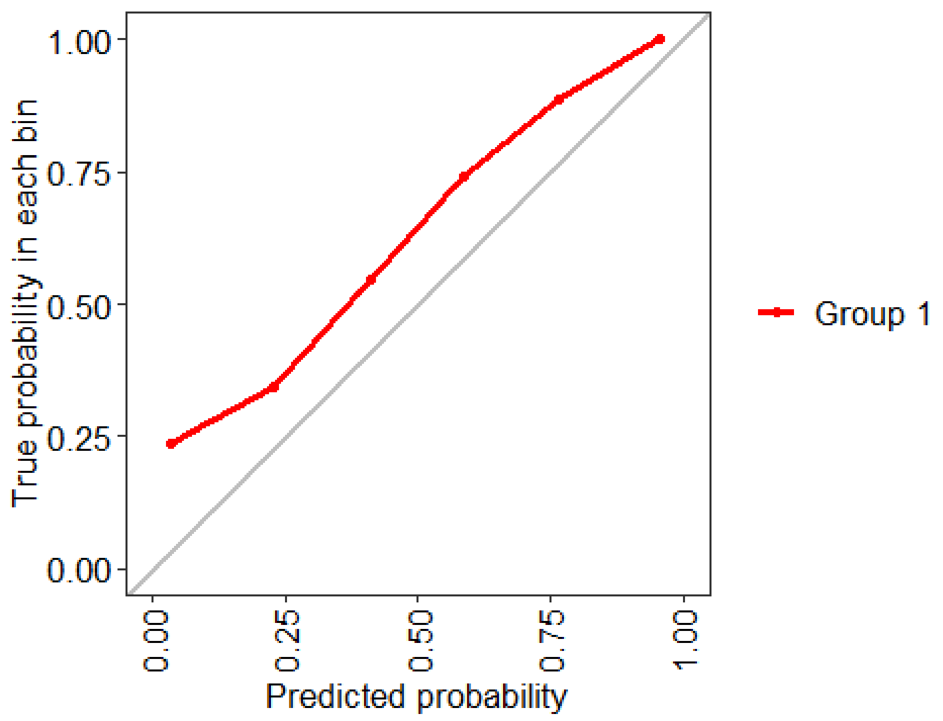

1.00
0.75
0.50
0.25
0.00
True probability in each bin
0.00
0.25
0.50
0.75
1.00
Predicted probability
Group 1

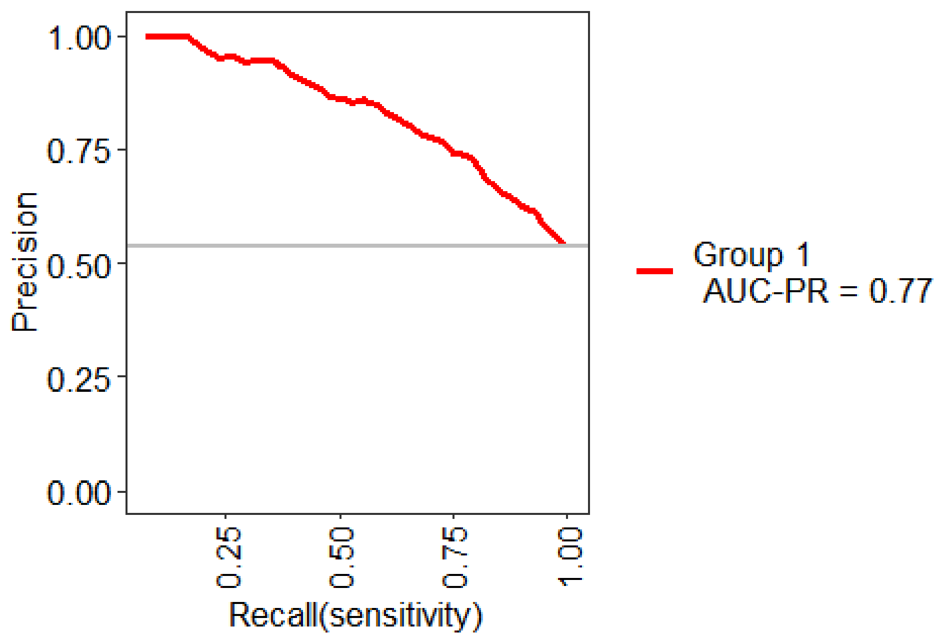

1.00
0.75
0.50
0.25
0.00
Precision
0.25
0.50
0.75
1.00
Recall(sensitivity)
Group 1
AUC-PR = 0.77

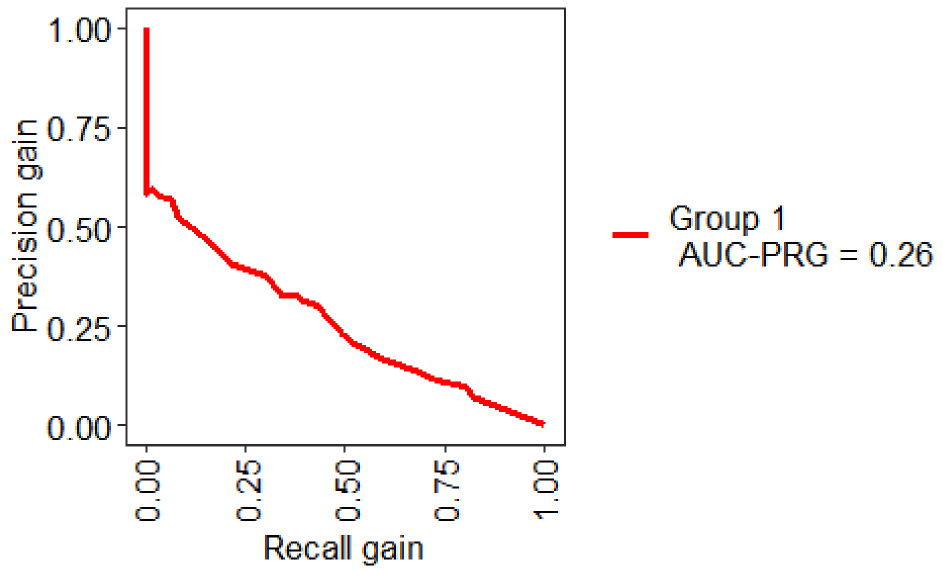

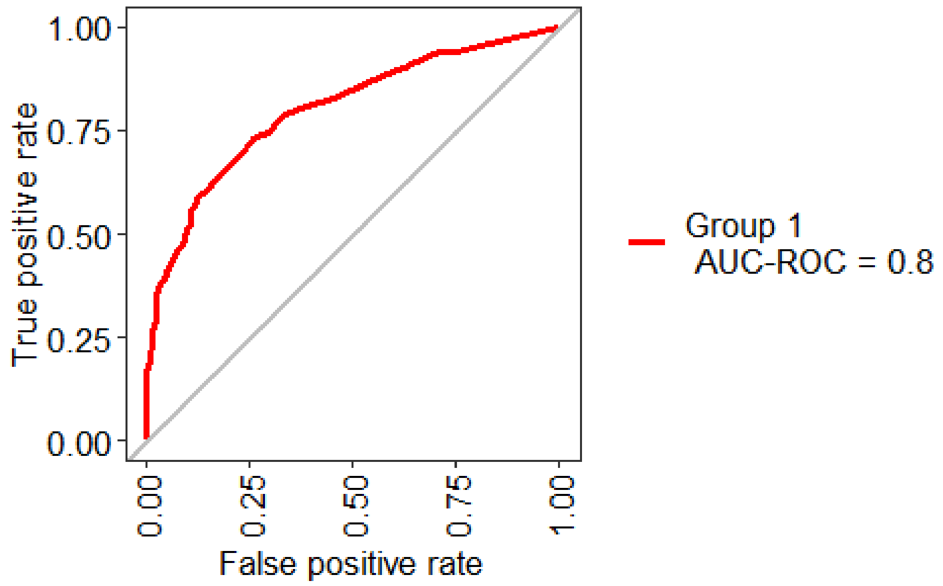


```
x_knnsmote$stdres
```

```
## $`Group 1`
##                   Score        CI
## SENS              0.562  0.52-0.6
## SPEC              0.888 0.86-0.91
## MCC               0.470      <NA>
## Informedness      0.450      <NA>
## PREC              0.853 0.81-0.89
## NPV               0.637  0.6-0.67
## FPR               0.112      <NA>
## F1                0.678      <NA>
## TP              302.000      <NA>
## FP               52.000      <NA>
## TN              412.000      <NA>
## FN              235.000      <NA>
## AUC-ROC           0.800 0.77-0.83
## AUC-PR            0.770      <NA>
## AUC-PRG           0.260      <NA>
```

```
test_pred_knnsmote <- predict(knn_fit_smote, newdata = testdata)
confusionMatrix(test_pred_knnsmote, testdata$Outcome)
```

```
## Confusion Matrix and Statistics
##
##            Reference
## Prediction favor unfavor
##    favor     108      39
##    unfavor   100      50
##
##                Accuracy : 0.532
##                  95% CI : (0.4735, 0.5898)
##     No Information Rate : 0.7003
##     P-Value [Acc > NIR] : 1
##
##                   Kappa : 0.0677
##
##  Mcnemar's Test P-Value : 3.597e-07
##
##             Sensitivity : 0.5192
##             Specificity : 0.5618
##          Pos Pred Value : 0.7347
##          Neg Pred Value : 0.3333
##              Prevalence : 0.7003
##          Detection Rate : 0.3636
##    Detection Prevalence : 0.4949
##       Balanced Accuracy : 0.5405
##
##        'Positive' Class : favor
##
```

```
test_prob_knnsmote <- predict(knn_fit_smote, newdata = testdata, type="prob")
mknnsmote = data.frame(test_prob_knnsmote, testdata$Outcome)
yknnsmote<-evalm(mknnsmote)
```

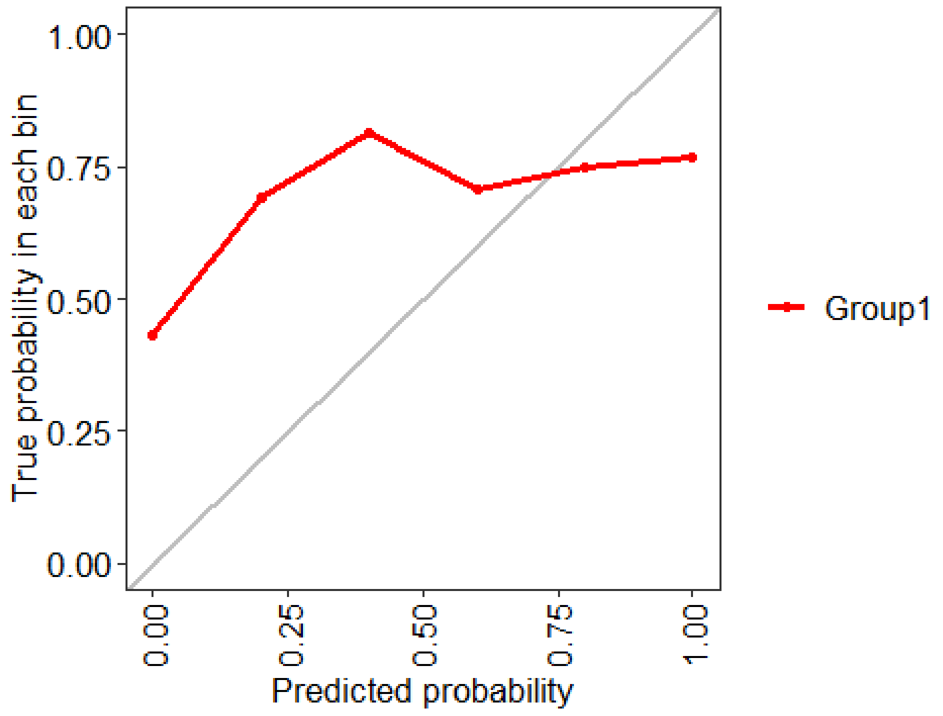

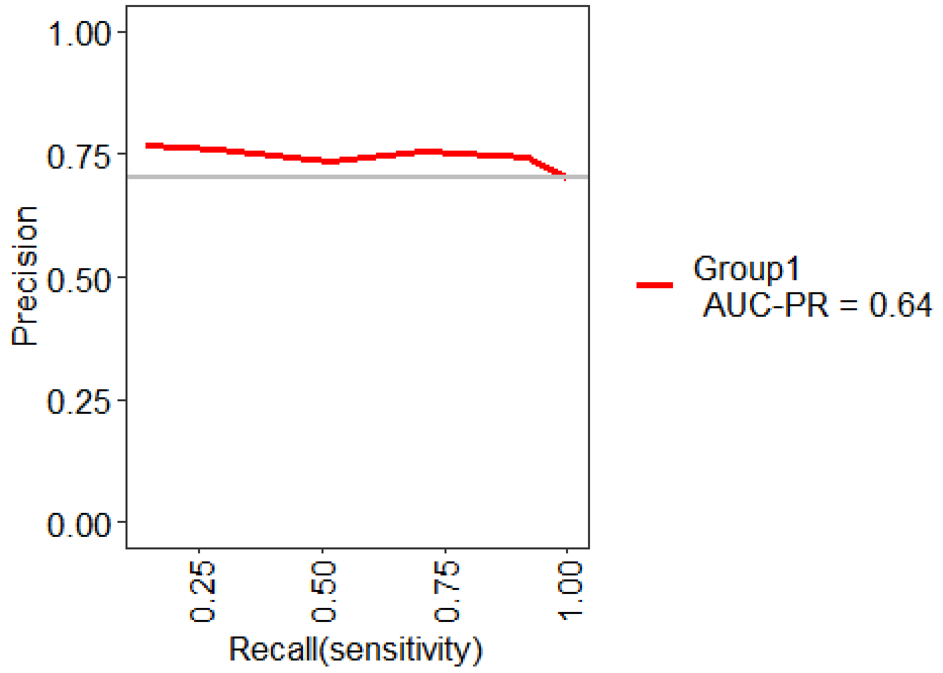

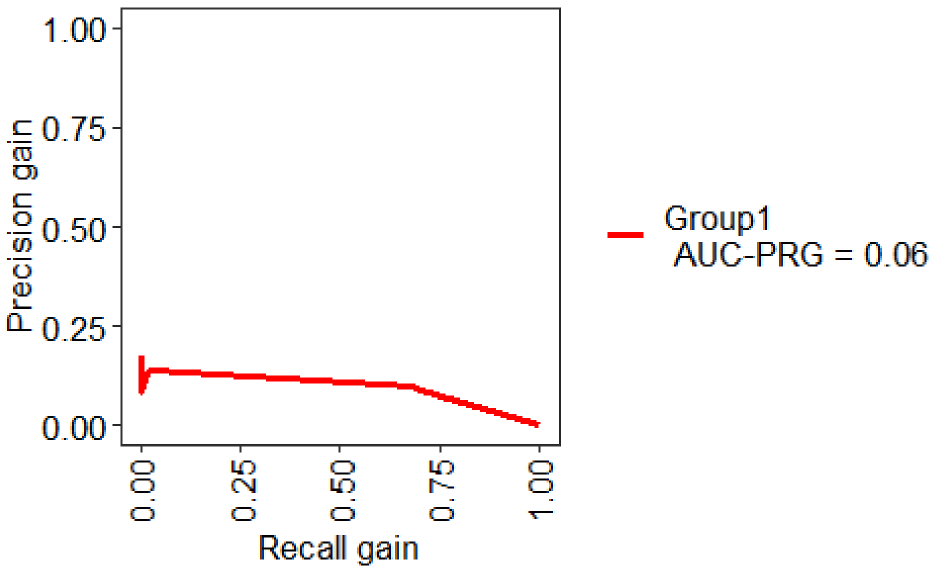

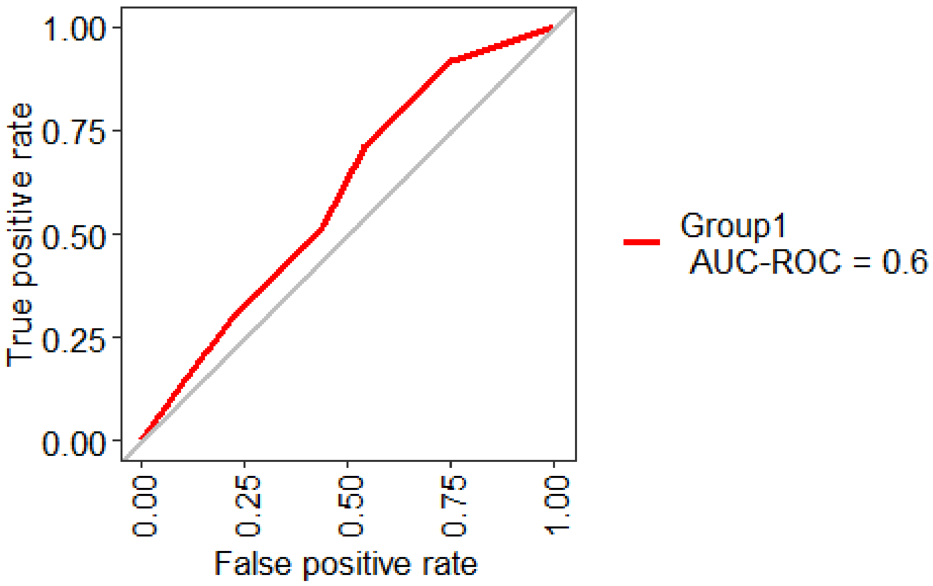


```
yknnsmote$stdres
```

```
## $Group1
##                   Score        CI
## SENS              0.519 0.45-0.59
## SPEC              0.562 0.46-0.66
## MCC               0.074      <NA>
## Informedness      0.081      <NA>
## PREC              0.735  0.66-0.8
## NPV               0.333 0.26-0.41
## FPR               0.438      <NA>
## F1                0.608      <NA>
## TP              108.000      <NA>
## FP               39.000      <NA>
## TN               50.000      <NA>
## FN              100.000      <NA>
## AUC-ROC           0.600 0.53-0.67
## AUC-PR            0.640      <NA>
## AUC-PRG           0.060      <NA>
```

```
set.seed(seed)
lr_fit_smote<-train(class~., data = traindata.smote, method = "regLogistic",
                    trControl=ctrl, metric=metric, tuneLength=5, verbose=FALS
E)
lr_fit_smote
```

```
## Regularized Logistic Regression
##
## 1001 samples
##   60 predictor
##    2 classes: 'unfavor', 'favor'
##
## No pre-processing
## Resampling: Cross-Validated (10 fold, repeated 10 times)
## Summary of sample sizes: 901, 902, 901, 901, 901, 900, ...
## Resampling results across tuning parameters:
##
##   cost  loss       epsilon  ROC        Sens       Spec
##   0.25  L1         1e-04    0.7969245  0.6323959  0.7944514
##   0.25  L1         1e-03    0.7969850  0.6321832  0.7942662
##   0.25  L1         1e-02    0.7971942  0.6308973  0.7948218
##   0.25  L1         1e-01    0.7960505  0.6270259  0.7948253
##   0.25  L1         1e+00    0.7689637  0.6030065  0.7881237
##   0.25  L2_dual    1e-04    0.7929293  0.6347225  0.7925751
##   0.25  L2_dual    1e-03    0.7929332  0.6347225  0.7925751
##   0.25  L2_dual    1e-02    0.7929452  0.6347225  0.7927603
##   0.25  L2_dual    1e-01    0.7929293  0.6347225  0.7935115
##   0.25  L2_dual    1e+00    0.7929583  0.6332285  0.7937072
##   0.25  L2_primal  1e-04    0.7929412  0.6347225  0.7925751
##   0.25  L2_primal  1e-03    0.7929898  0.6349399  0.7927603
##   0.25  L2_primal  1e-02    0.7927406  0.6327798  0.7923934
```

```
##   0.25  L2_primal  1e-01    0.7901499  0.6295930  0.7940776
##   0.25  L2_primal  1e+00    0.7765499  0.6058788  0.7973934
##   0.50  L1         1e-04    0.7956599  0.6302313  0.7916667
##   0.50  L1         1e-03    0.7956641  0.6298011  0.7914745
##   0.50  L1         1e-02    0.7959197  0.6304579  0.7905451
##   0.50  L1         1e-01    0.7960497  0.6269796  0.7929630
##   0.50  L1         1e+00    0.7625651  0.6041119  0.7796716
##   0.50  L2_dual    1e-04    0.7932344  0.6403191  0.7918274
##   0.50  L2_dual    1e-03    0.7932344  0.6403191  0.7918274
##   0.50  L2_dual    1e-02    0.7932427  0.6403191  0.7916422
##   0.50  L2_dual    1e-01    0.7932417  0.6401110  0.7920126
##   0.50  L2_dual    1e+00    0.7928623  0.6390333  0.7914535
##   0.50  L2_primal  1e-04    0.7932303  0.6403191  0.7918274
##   0.50  L2_primal  1e-03    0.7932786  0.6398890  0.7912718
##   0.50  L2_primal  1e-02    0.7931668  0.6396762  0.7918309
##   0.50  L2_primal  1e-01    0.7906780  0.6345097  0.7927778
##   0.50  L2_primal  1e+00    0.7778085  0.6093293  0.7958980
##   1.00  L1         1e-04    0.7942840  0.6358326  0.7918414
##   1.00  L1         1e-03    0.7941958  0.6356198  0.7918414
##   1.00  L1         1e-02    0.7936938  0.6315264  0.7909154
##   1.00  L1         1e-01    0.7943349  0.6302220  0.7922257
##   1.00  L1         1e+00    0.7585264  0.6062211  0.7685639
##   1.00  L2_dual    1e-04    0.7936385  0.6435338  0.7918274
##   1.00  L2_dual    1e-03    0.7936426  0.6435338  0.7918274
##   1.00  L2_dual    1e-02    0.7936588  0.6435338  0.7920161
##   1.00  L2_dual    1e-01    0.7936349  0.6439685  0.7920161
##   1.00  L2_dual    1e+00    0.7932739  0.6413969  0.7901642
##   1.00  L2_primal  1e-04    0.7936385  0.6435338  0.7918274
##   1.00  L2_primal  1e-03    0.7936465  0.6437465  0.7914570
##   1.00  L2_primal  1e-02    0.7935280  0.6435291  0.7920126
##   1.00  L2_primal  1e-01    0.7914801  0.6385846  0.7931551
##   1.00  L2_primal  1e+00    0.7782699  0.6128076  0.7953215
##   2.00  L1         1e-04    0.7935205  0.6418409  0.7927743
##   2.00  L1         1e-03    0.7934173  0.6420537  0.7925856
##   2.00  L1         1e-02    0.7926995  0.6401156  0.7899755
##   2.00  L1         1e-01    0.7911561  0.6308557  0.7914885
##   2.00  L1         1e+00    0.7606968  0.6127428  0.7687282
##   2.00  L2_dual    1e-04    0.7936976  0.6480620  0.7916422
##   2.00  L2_dual    1e-03    0.7937015  0.6480620  0.7916422
##   2.00  L2_dual    1e-02    0.7937178  0.6480620  0.7918274
##   2.00  L2_dual    1e-01    0.7938023  0.6480574  0.7916422
##   2.00  L2_dual    1e+00    0.7933012  0.6471878  0.7907128
##   2.00  L2_primal  1e-04    0.7937057  0.6480620  0.7916422
##   2.00  L2_primal  1e-03    0.7937221  0.6480620  0.7916422
##   2.00  L2_primal  1e-02    0.7936337  0.6480574  0.7918274
##   2.00  L2_primal  1e-01    0.7919345  0.6418363  0.7920300
##   2.00  L2_primal  1e+00    0.7784615  0.6149537  0.7934696
##   4.00  L1         1e-04    0.7934949  0.6448520  0.7923899
##   4.00  L1         1e-03    0.7933116  0.6448381  0.7923899
```

```
##   4.00  L1          1e-02    0.7918242  0.6426735  0.7905346
##   4.00  L1          1e-01    0.7900653  0.6355550  0.7907268
##   4.00  L1          1e+00    0.7558755  0.5988945  0.7629804
##   4.00  L2_dual     1e-04    0.7935897  0.6480666  0.7916387
##   4.00  L2_dual     1e-03    0.7935897  0.6480666  0.7916387
##   4.00  L2_dual     1e-02    0.7935815  0.6480666  0.7916387
##   4.00  L2_dual     1e-01    0.7936214  0.6484921  0.7918239
##   4.00  L2_dual     1e+00    0.7932773  0.6485338  0.7901677
##   4.00  L2_primal   1e-04    0.7935976  0.6480666  0.7916387
##   4.00  L2_primal   1e-03    0.7935613  0.6480666  0.7916387
##   4.00  L2_primal   1e-02    0.7935213  0.6487095  0.7910797
##   4.00  L2_primal   1e-01    0.7919995  0.6446531  0.7907268
##   4.00  L2_primal   1e+00    0.7783605  0.6149537  0.7932949
##
## ROC was used to select the optimal model using the largest value.
## The final values used for the model were cost = 0.25, loss = L1 and epsilo
n
##  = 0.01.
```

```
xlrsmote <- evalm(lr_fit_smote)
```

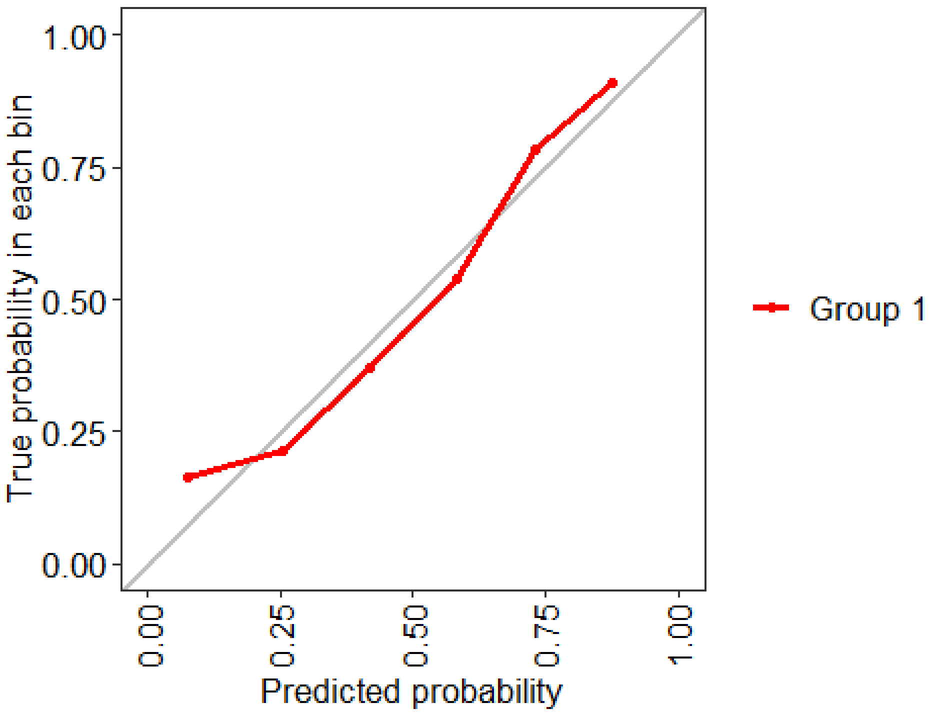

True probability in each bin
1.00
0.75
0.50
0.25
0.00
0.00
0.25
0.50
0.75
1.00
Predicted probability
Group 1

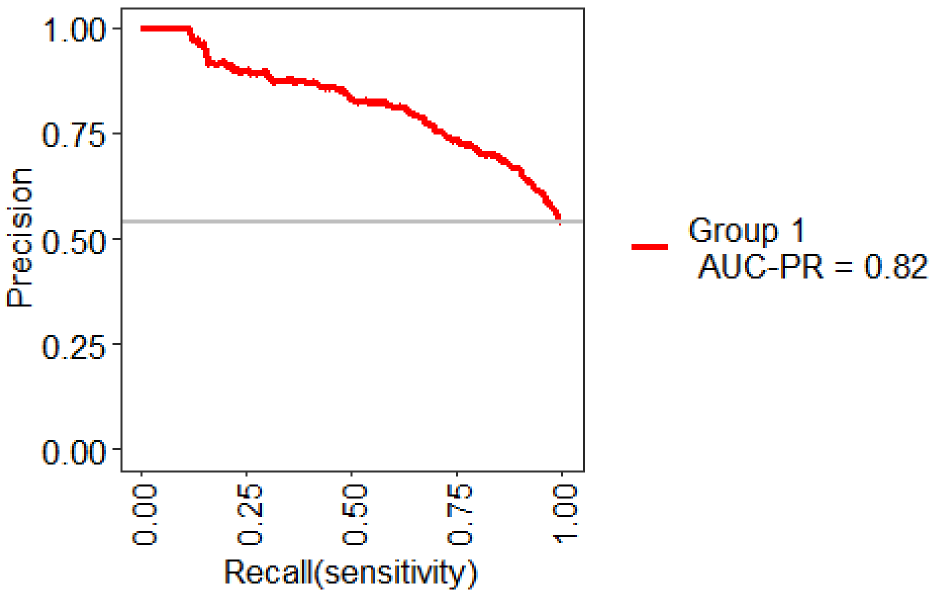

Precision
1.00
0.75
0.50
0.25
0.00
0.00
0.25
0.50
0.75
1.00
Recall(sensitivity)
Group 1
AUC-PR = 0.82

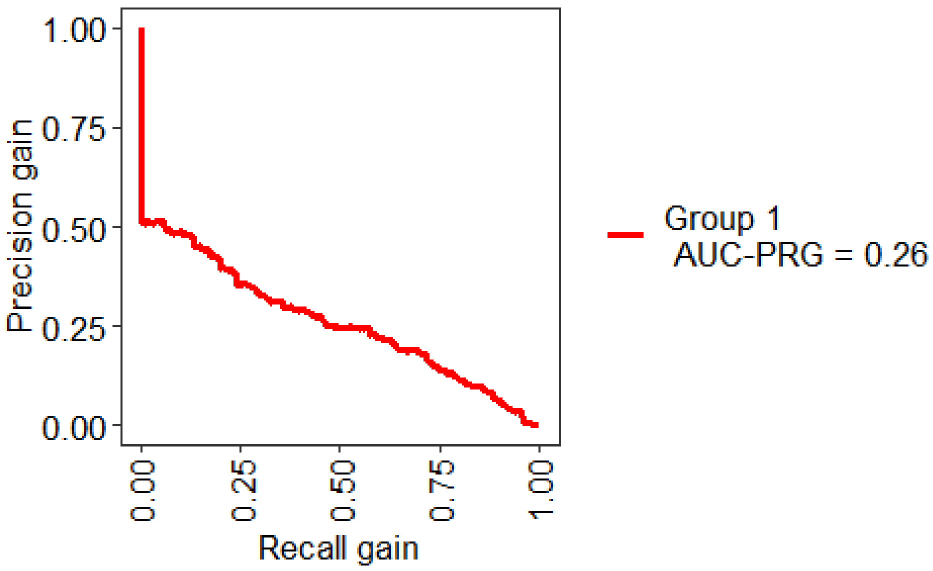

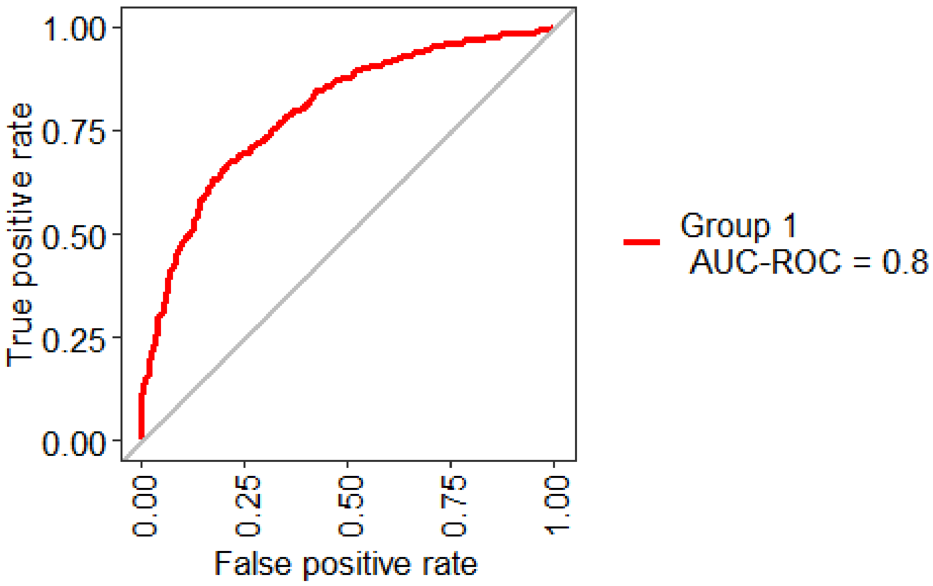


```
xlrsmote$stdres
```

```
## $`Group 1`
##                    Score        CI
## SENS               0.791 0.76-0.82
## SPEC               0.636 0.59-0.68
## MCC                0.434      <NA>
## Informedness       0.427      <NA>
## PREC               0.715 0.68-0.75
## NPV                0.725 0.68-0.77
## FPR                0.364      <NA>
## F1                 0.752      <NA>
## TP               425.000      <NA>
## FP               169.000      <NA>
## TN               295.000      <NA>
## FN               112.000      <NA>
## AUC-ROC            0.800 0.77-0.83
## AUC-PR             0.820      <NA>
## AUC-PRG            0.260      <NA>
```

```
test_pred_lrsmote <- predict(lr_fit_smote, newdata = testdata)
confusionMatrix(test_pred_lrsmote, testdata$Outcome)
```

```
## Confusion Matrix and Statistics
##
##           Reference
## Prediction favor unfavor
##    favor     172      28
##    unfavor    36      61
##
##                Accuracy : 0.7845
##                  95% CI : (0.7333, 0.8299)
##     No Information Rate : 0.7003
##     P-Value [Acc > NIR] : 0.0007089
##
##                   Kappa : 0.4995
##
##  Mcnemar's Test P-Value : 0.3815739
##
##             Sensitivity : 0.8269
##             Specificity : 0.6854
##          Pos Pred Value : 0.8600
##          Neg Pred Value : 0.6289
##              Prevalence : 0.7003
##          Detection Rate : 0.5791
##    Detection Prevalence : 0.6734
##       Balanced Accuracy : 0.7562
##
##        'Positive' Class : favor
##
```

```r
test_prob_lrsmote <- predict(lr_fit_smote, newdata = testdata, type="prob")
mlrsmote = data.frame(test_prob_lrsmote, testdata$Outcome)
ylrsmote<-evalm(mlrsmote)
```

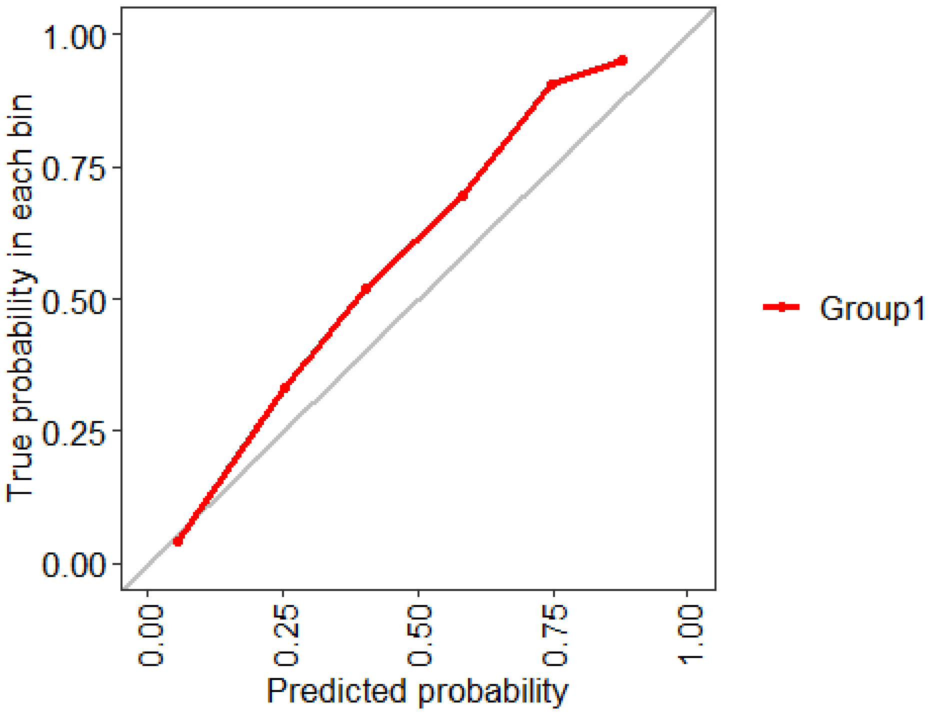

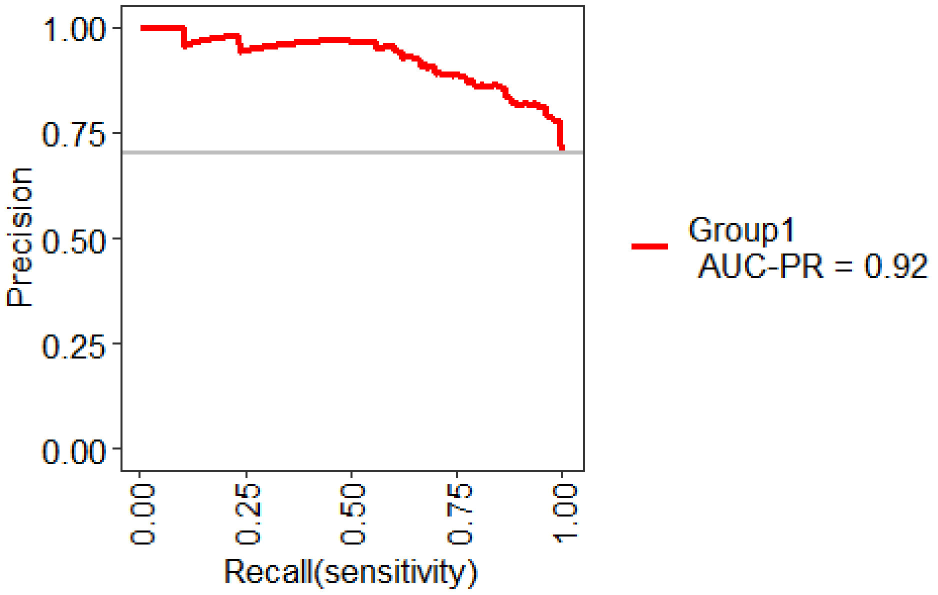

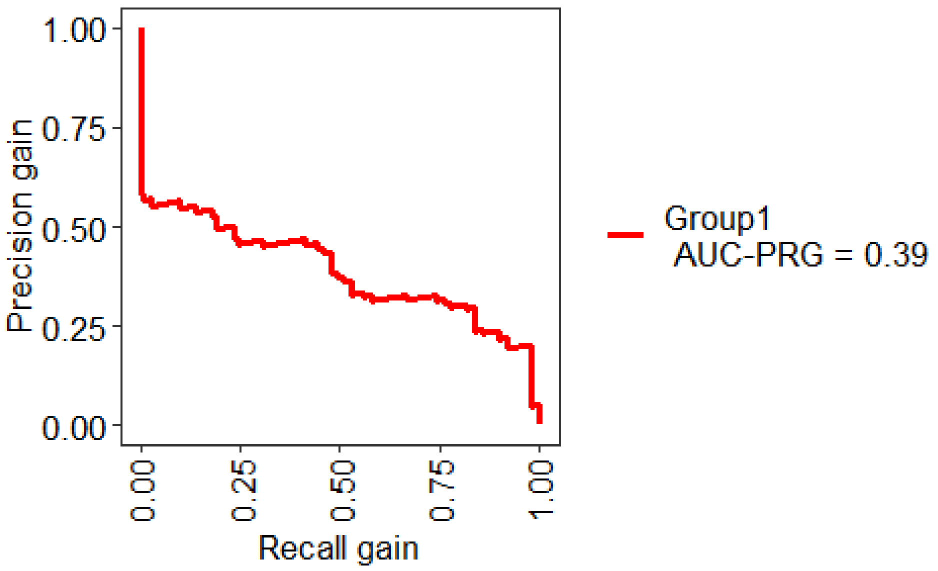

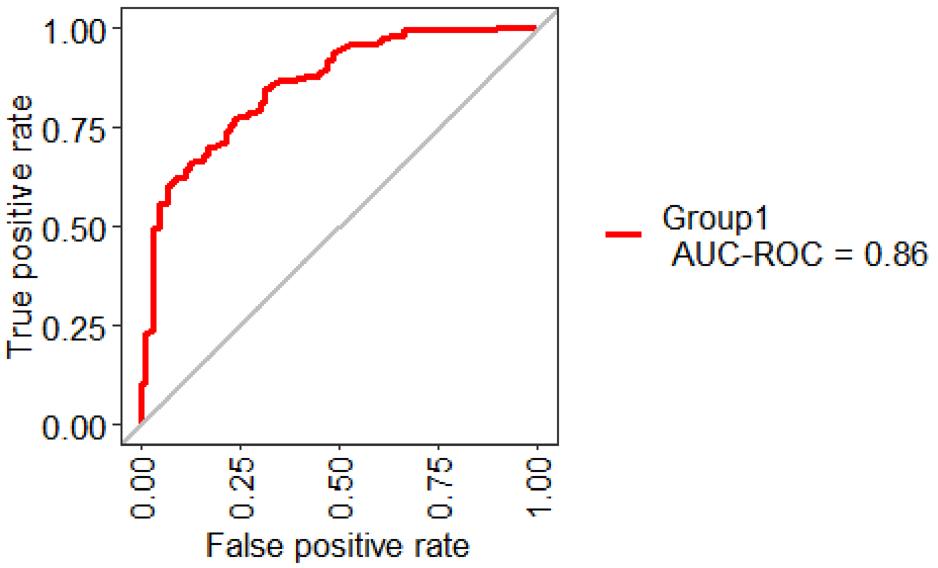


```
ylrsmote$stdres
```

```
## $Group1
##                   Score        CI
## SENS              0.827 0.77-0.87
## SPEC              0.685 0.58-0.77
## MCC               0.500      <NA>
## Informedness      0.512      <NA>
## PREC              0.860  0.81-0.9
## NPV               0.629 0.53-0.72
## FPR               0.315      <NA>
## F1                0.843      <NA>
## TP              172.000      <NA>
## FP               28.000      <NA>
## TN               61.000      <NA>
## FN               36.000      <NA>
## AUC-ROC           0.860  0.82-0.9
## AUC-PR            0.920      <NA>
## AUC-PRG           0.390      <NA>

set.seed(seed)
traindata.adas <- ADAS(traindata[,-1], traindata$Outcome)
traindata.adas <- traindata.adas$data
traindata.adas$class<-as.factor(traindata.adas$class)
table(traindata.adas$class)

##
## unfavor   favor
##     555     537

set.seed(seed)
rf_fit_adas1 <- train(class~., data = traindata.adas, method = "rf",
                      trControl=ctrl, metric=metric, tuneLength=10,
                      ntree=500, verbose=FALSE)
rf_fit_adas1

## Random Forest
##
## 1092 samples
##   60 predictor
##    2 classes: 'unfavor', 'favor'
##
## No pre-processing
## Resampling: Cross-Validated (10 fold, repeated 10 times)
## Summary of sample sizes: 982, 984, 983, 983, 983, 982, ...
## Resampling results across tuning parameters:
##
##   mtry  ROC        Sens       Spec
##    2    0.9416938  0.8126656  0.8921698
##    8    0.9476428  0.8747727  0.8549022
##   14    0.9433361  0.8848864  0.8290496
##   21    0.9382339  0.8866721  0.8156464
```

```
##   27    0.9337678  0.8888344  0.8100559
##   34    0.9306829  0.8876039  0.8096716
##   40    0.9282187  0.8886623  0.8070929
##   47    0.9256722  0.8885097  0.8027848
##   53    0.9247812  0.8867045  0.8043047
##   60    0.9230139  0.8870714  0.8007338
##
## ROC was used to select the optimal model using the largest value.
## The final value used for the model was mtry = 8.
```

```
xadas1 <- evalm(rf_fit_adas1)
```

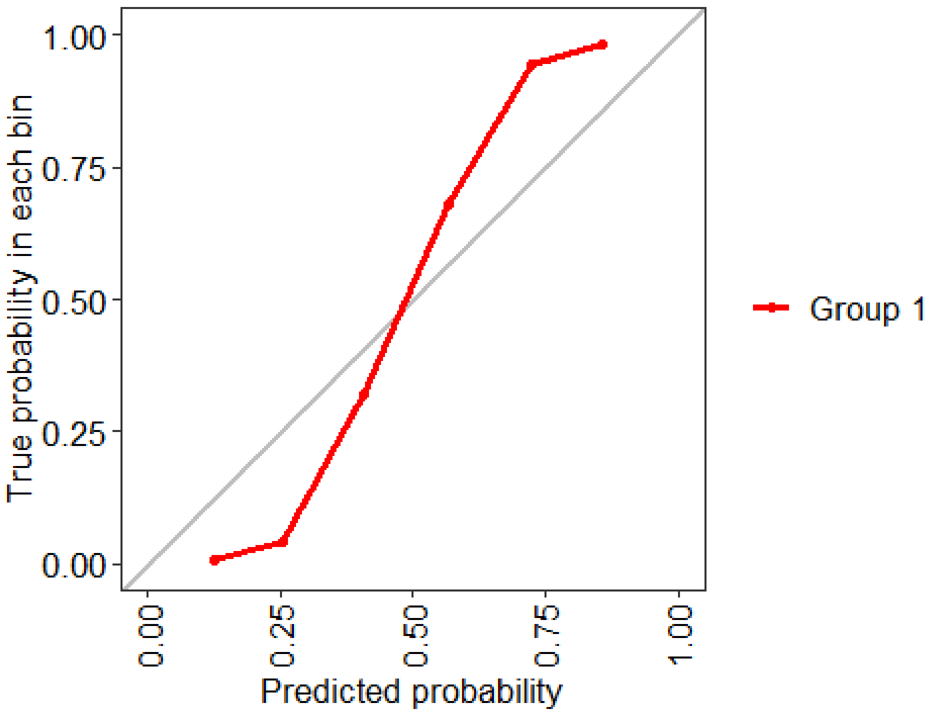
True probability in each bin
1.00
0.75
0.50
0.25
0.00
0.00
0.25
0.50
0.75
1.00
Predicted probability
Group 1

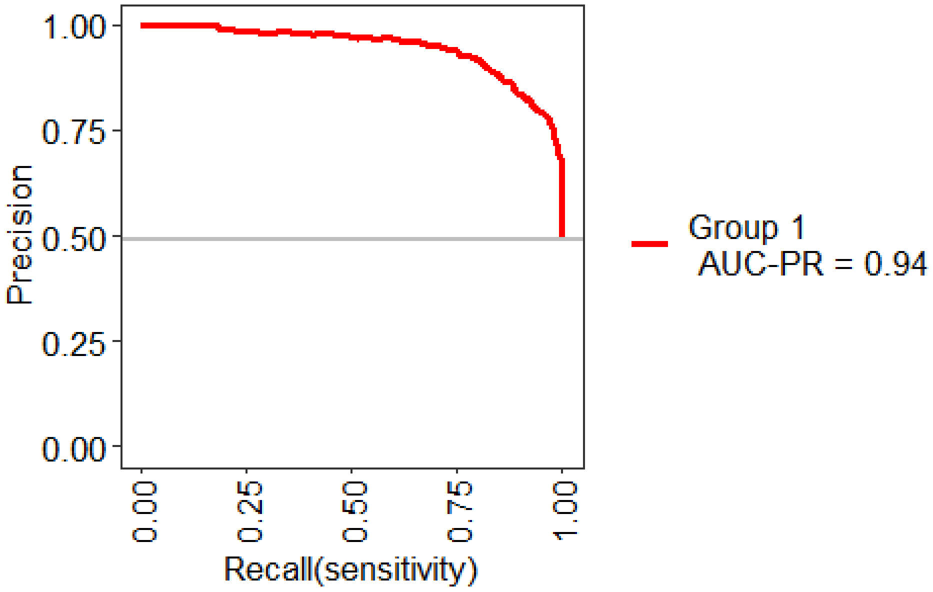
Precision
1.00
0.75
0.50
0.25
0.00
0.00
0.25
0.50
0.75
1.00
Recall(sensitivity)
Group 1
AUC-PR = 0.94

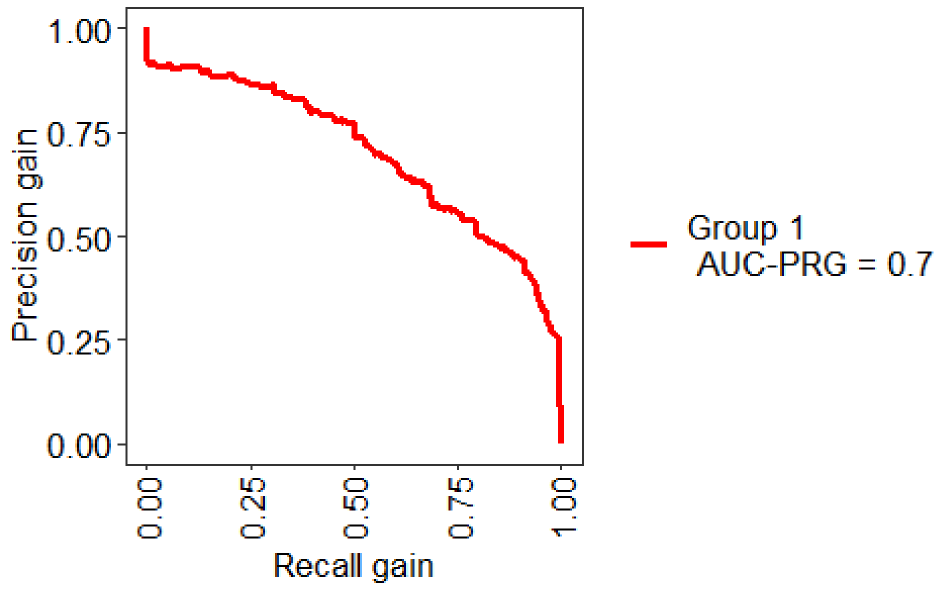

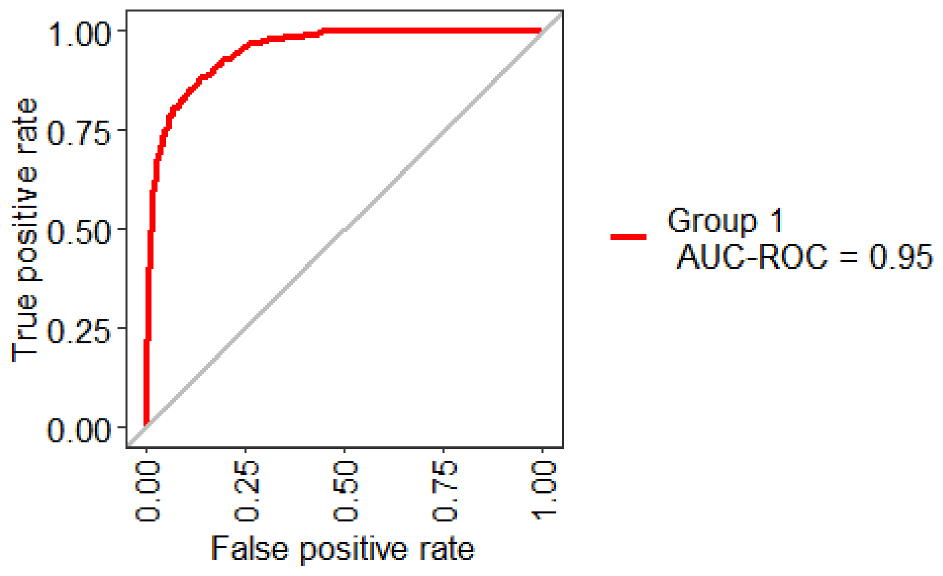


```
xadas1$stdres
```

```
## $`Group 1`
##                  Score        CI
## SENS             0.857 0.82-0.88
## SPEC             0.876  0.85-0.9
## MCC              0.733      <NA>
## Informedness     0.732      <NA>
## PREC             0.870  0.84-0.9
## NPV              0.863 0.83-0.89
## FPR              0.124      <NA>
## F1               0.863      <NA>
## TP             460.000      <NA>
## FP              69.000      <NA>
## TN             486.000      <NA>
## FN              77.000      <NA>
## AUC-ROC          0.950 0.94-0.96
## AUC-PR           0.940      <NA>
## AUC-PRG          0.700      <NA>
```

```
test_pred_rfadas1 <- predict(rf_fit_adas1, newdata = testdata)
confusionMatrix(test_pred_rfadas1, testdata$Outcome)
```

```
## Confusion Matrix and Statistics
## 
##           Reference
## Prediction favor unfavor
##    favor     183      40
##    unfavor    25      49
## 
##                Accuracy : 0.7811
##                  95% CI : (0.7297, 0.8268)
##     No Information Rate : 0.7003
##     P-Value [Acc > NIR] : 0.001122
## 
##                   Kappa : 0.4522
## 
##  Mcnemar's Test P-Value : 0.082478
## 
##             Sensitivity : 0.8798
##             Specificity : 0.5506
##          Pos Pred Value : 0.8206
##          Neg Pred Value : 0.6622
##              Prevalence : 0.7003
##          Detection Rate : 0.6162
##    Detection Prevalence : 0.7508
##       Balanced Accuracy : 0.7152
## 
##        'Positive' Class : favor
## 
```

```
test_prob_adas1 <- predict(rf_fit_adas1, newdata = testdata, type="prob")
madas1 = data.frame(test_prob_adas1, testdata$Outcome)
yadas1<-evalm(madas1)
```

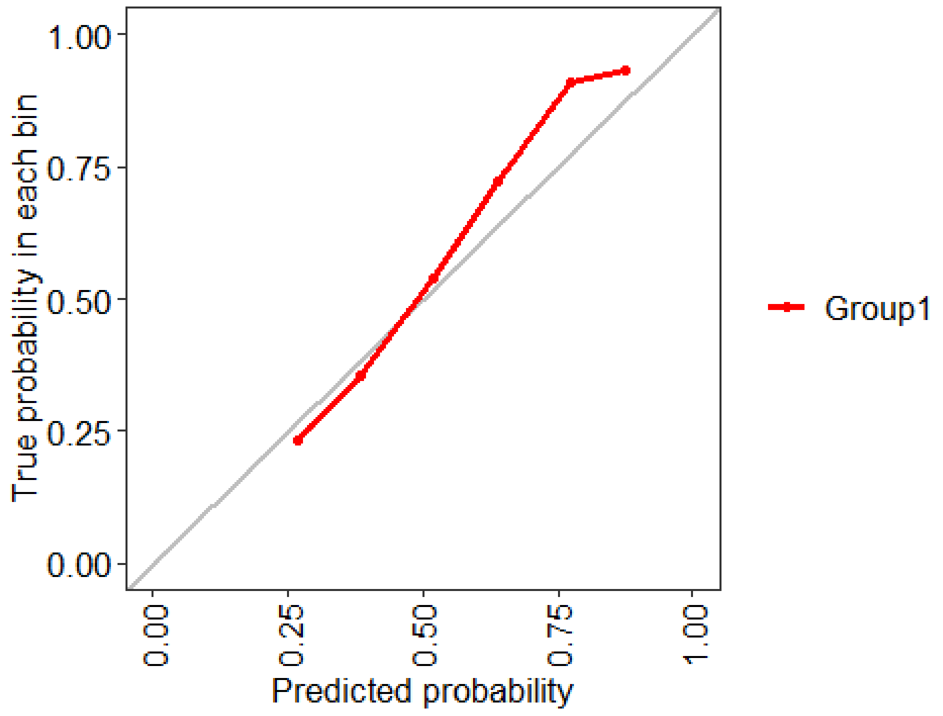

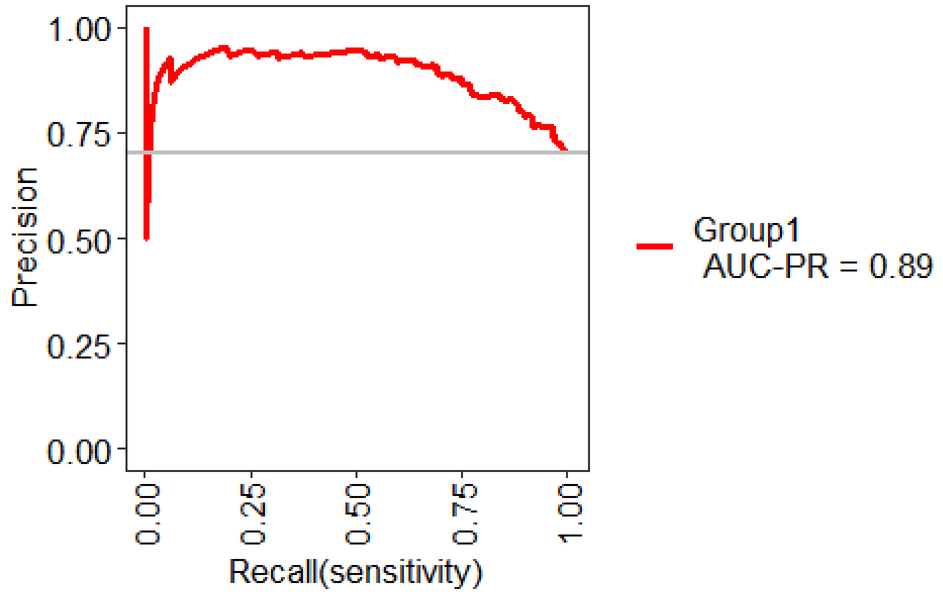

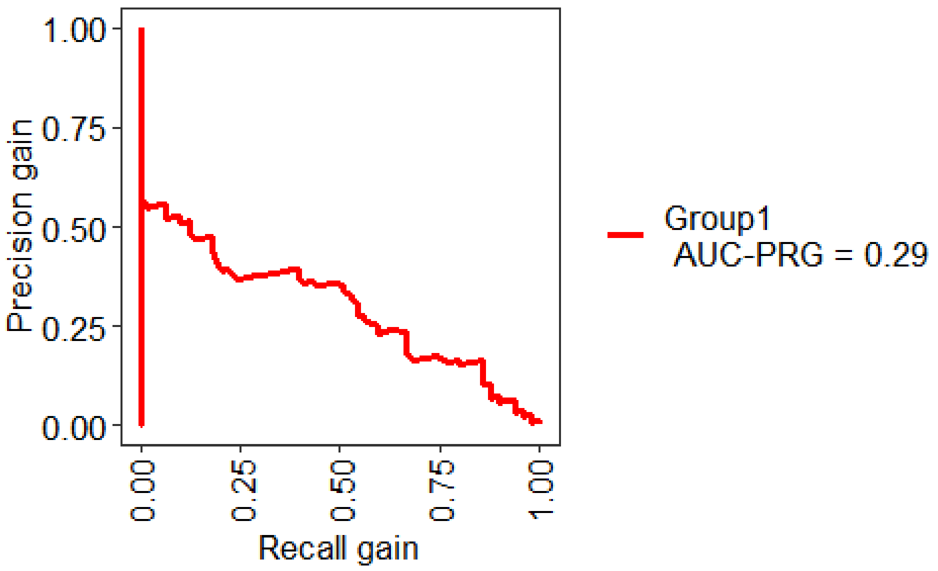

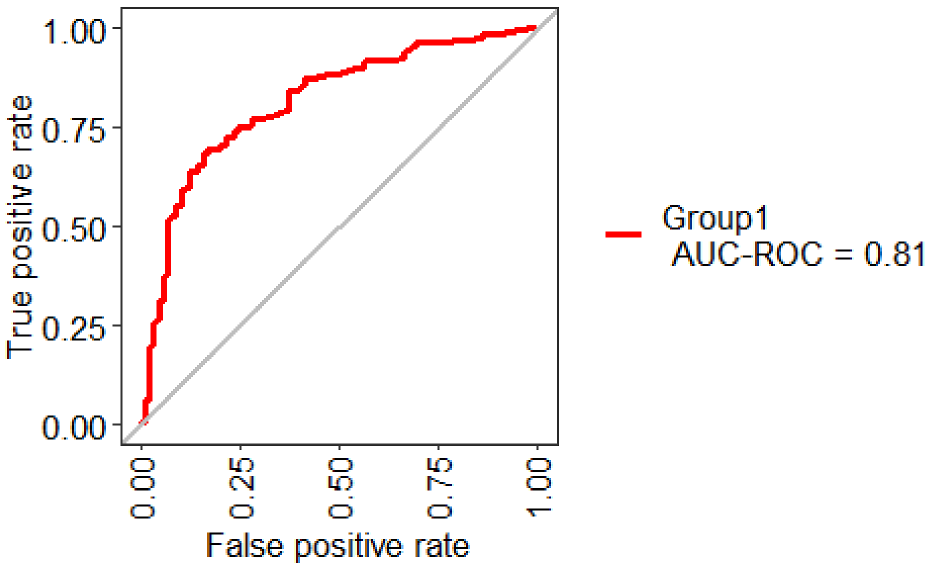


```
yadas1$stdres
```

```
## $Group1
##                  Score        CI
## SENS             0.880 0.83-0.92
## SPEC             0.539 0.44-0.64
## MCC              0.446      <NA>
## Informedness     0.419      <NA>
## PREC             0.817 0.76-0.86
## NPV              0.658 0.54-0.76
## FPR              0.461      <NA>
## F1               0.847      <NA>
## TP             183.000      <NA>
## FP              41.000      <NA>
## TN              48.000      <NA>
## FN              25.000      <NA>
## AUC-ROC          0.810 0.76-0.86
## AUC-PR           0.890      <NA>
## AUC-PRG          0.290      <NA>
```

```
imp_adas1<-varImp(rf_fit_adas1, scale = FALSE)
imp_adas1
```

```
## rf variable importance
##
##   only 20 most important variables shown (out of 60)
##
##                 Overall
## NIHSS_adm        83.812
## Age              44.127
## Arrival          24.509
## Hb               20.915
## RanGlu           20.481
## TG               18.801
## SBP              17.547
## PLT              17.306
## BMI              17.145
## HDL              16.936
## TC               16.690
## DBP              16.294
## BUN              16.026
## LDL              15.836
## HbA1C            15.335
## AC               14.873
## Cr               13.689
## Circulation.2     9.191
## Circulation.1     7.601
## Sex.Male          6.692
```

```
plot(imp_adas1)
```

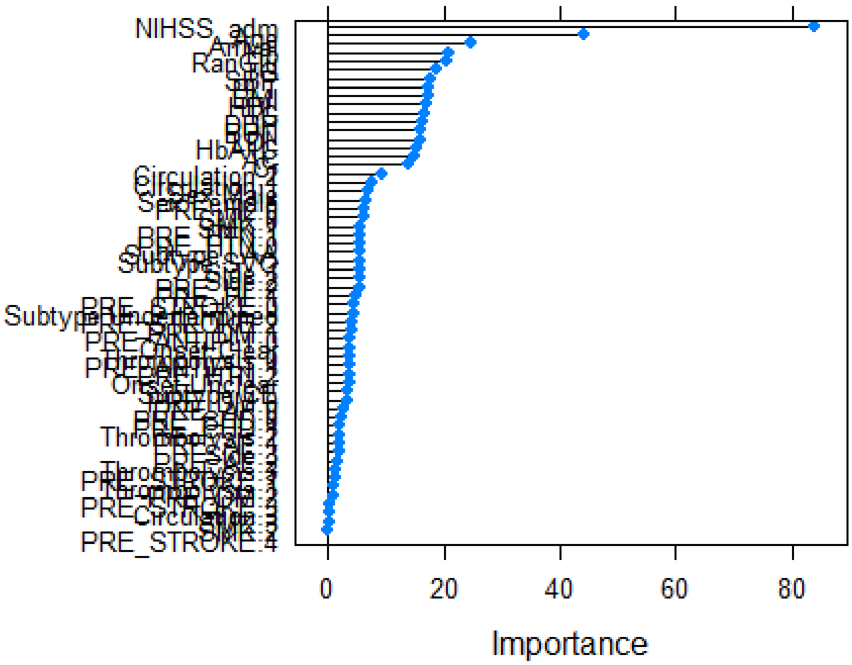


```
set.seed(seed)
rf_fit_adas2 <- train(class~., data = traindata.adas, method = "rf",
                      trControl=ctrl, metric=metric, tuneLength=10,
                      ntree=1000, verbose=FALSE)
rf_fit_adas2

## Random Forest
##
## 1092 samples
##   60 predictor
##    2 classes: 'unfavor', 'favor'
##
## No pre-processing
## Resampling: Cross-Validated (10 fold, repeated 10 times)
## Summary of sample sizes: 982, 984, 983, 983, 983, 982, ...
## Resampling results across tuning parameters:
##
##   mtry  ROC        Sens       Spec
##    2    0.9435779  0.8155325  0.8979280
##    8    0.9480666  0.8729935  0.8549196
##   14    0.9437288  0.8857825  0.8307128
##   21    0.9384581  0.8893896  0.8163802
##   27    0.9348037  0.8893734  0.8124738
##   34    0.9312007  0.8890390  0.8096751
##   40    0.9288731  0.8888279  0.8074668
##   47    0.9260299  0.8885000  0.8038889
```

```
##   53    0.9248544  0.8854448  0.8046506
##   60    0.9232808  0.8872468  0.8014710
##
## ROC was used to select the optimal model using the largest value.
## The final value used for the model was mtry = 8.

xadas2 <- evalm(rf_fit_adas2)
```

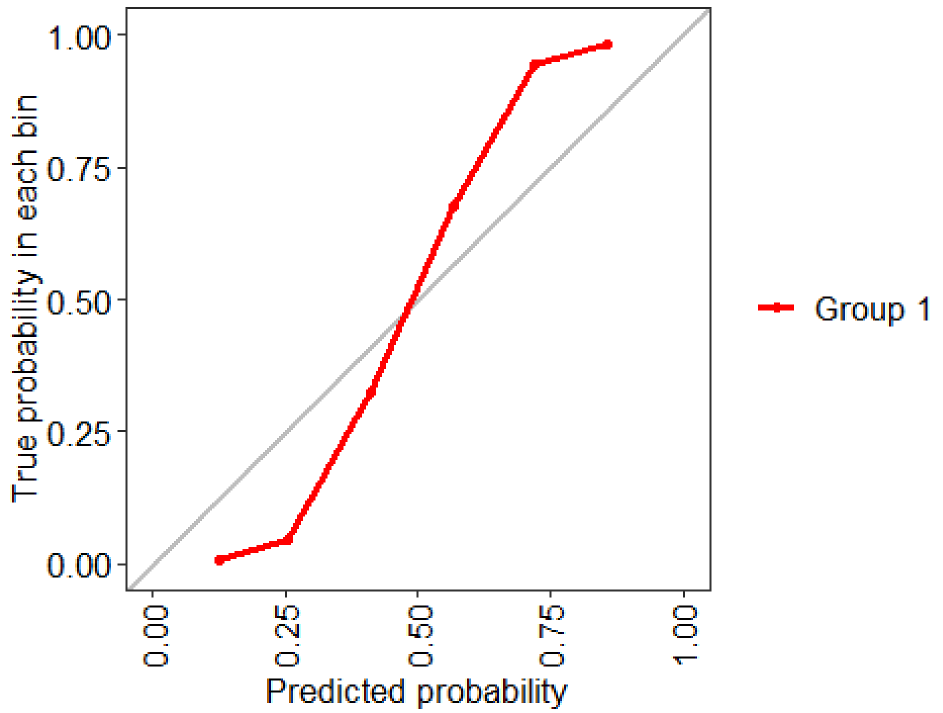
True probability in each bin
1.00
0.75
0.50
0.25
0.00
0.00
0.25
0.50
0.75
1.00
Predicted probability
Group 1

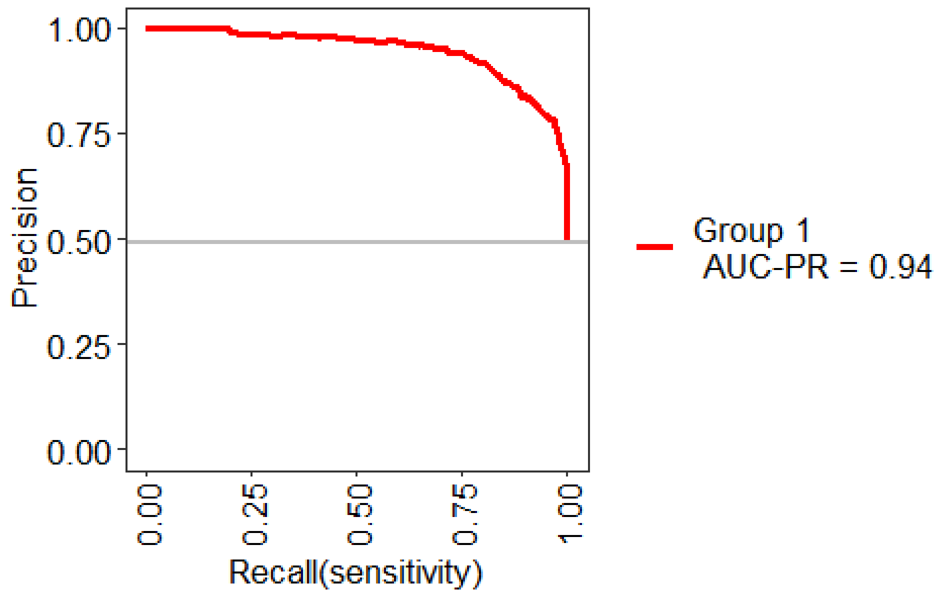
Precision
1.00
0.75
0.50
0.25
0.00
0.00
0.25
0.50
0.75
1.00
Recall(sensitivity)
Group 1
AUC-PR = 0.94

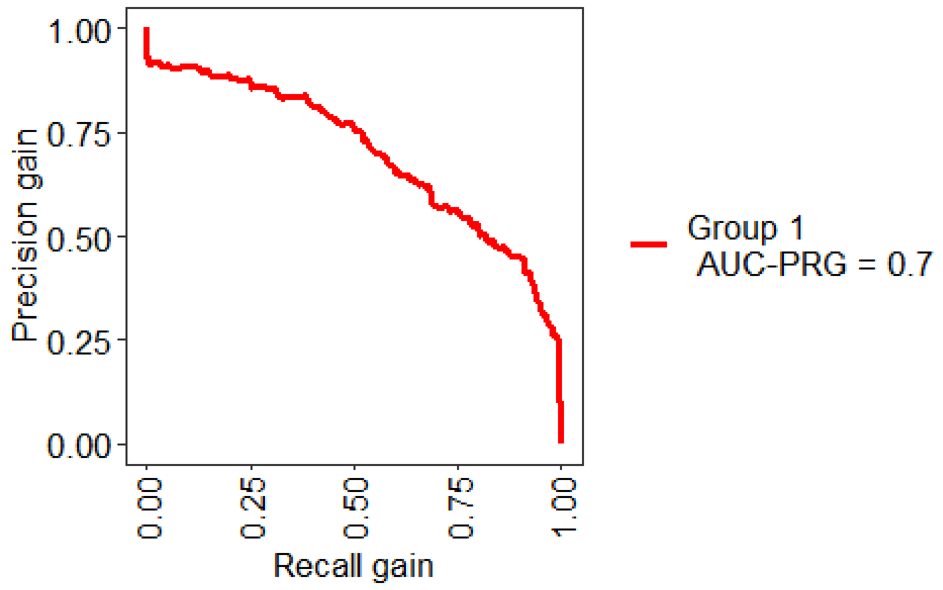

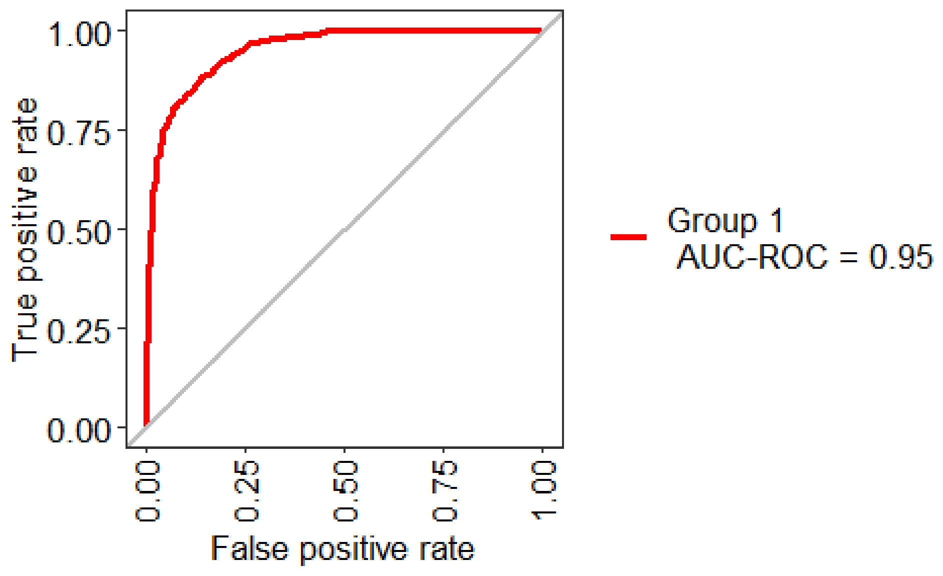


```
xadas2$stdres
```

```
## $`Group 1`
##                  Score        CI
## SENS             0.857 0.82-0.88
## SPEC             0.876  0.85-0.9
## MCC              0.733      <NA>
## Informedness     0.732      <NA>
## PREC             0.870  0.84-0.9
## NPV              0.863 0.83-0.89
## FPR              0.124      <NA>
## F1               0.863      <NA>
## TP             460.000      <NA>
## FP              69.000      <NA>
## TN             486.000      <NA>
## FN              77.000      <NA>
## AUC-ROC          0.950 0.94-0.96
## AUC-PR           0.940      <NA>
## AUC-PRG          0.700      <NA>
```

```
test_pred_rfadas2 <- predict(rf_fit_adas2, newdata = testdata)
confusionMatrix(test_pred_rfadas2, testdata$Outcome)
```

```
## Confusion Matrix and Statistics
## 
##           Reference
## Prediction favor unfavor
##    favor     183      41
##    unfavor    25      48
## 
##                Accuracy : 0.7778
##                  95% CI : (0.7262, 0.8238)
##     No Information Rate : 0.7003
##     P-Value [Acc > NIR] : 0.001743
## 
##                   Kappa : 0.4419
## 
##  Mcnemar's Test P-Value : 0.064838
## 
##             Sensitivity : 0.8798
##             Specificity : 0.5393
##          Pos Pred Value : 0.8170
##          Neg Pred Value : 0.6575
##              Prevalence : 0.7003
##          Detection Rate : 0.6162
##    Detection Prevalence : 0.7542
##       Balanced Accuracy : 0.7096
## 
##        'Positive' Class : favor
## 
```

```
test_prob_adas2 <- predict(rf_fit_adas2, newdata = testdata, type="prob")
madas2 = data.frame(test_prob_adas2, testdata$Outcome)
yadas2<-evalm(madas2)
```

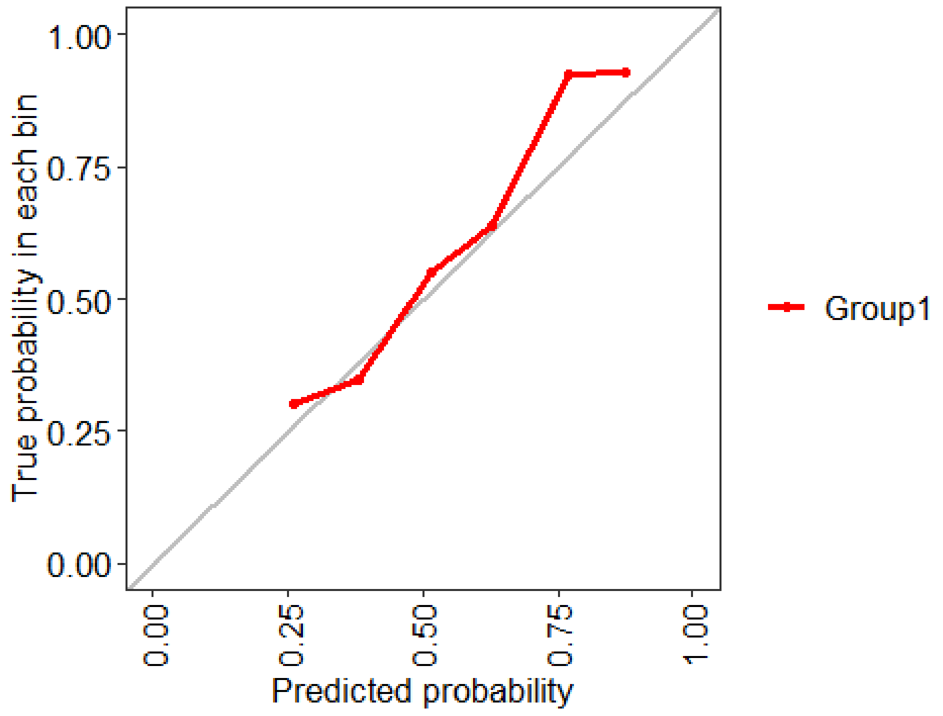

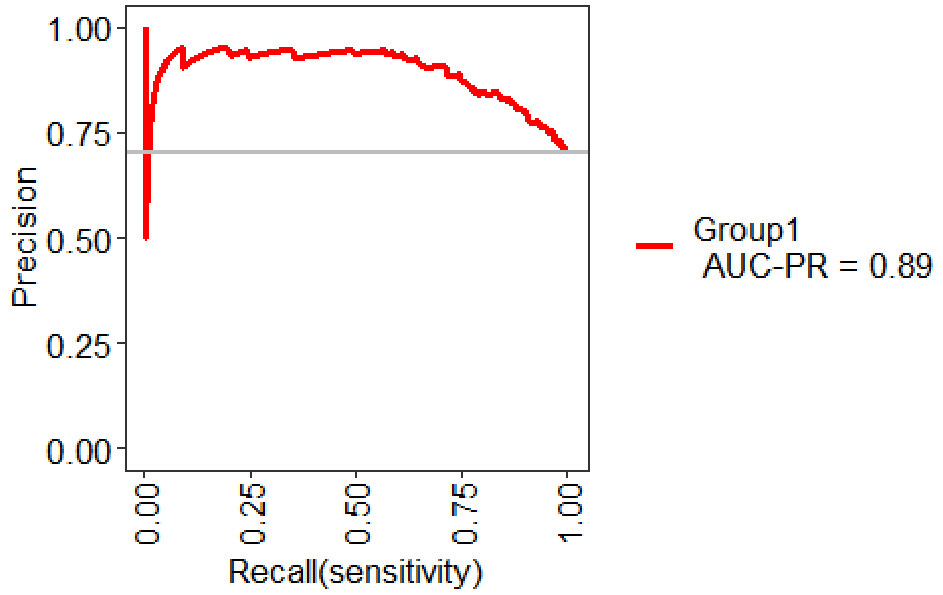

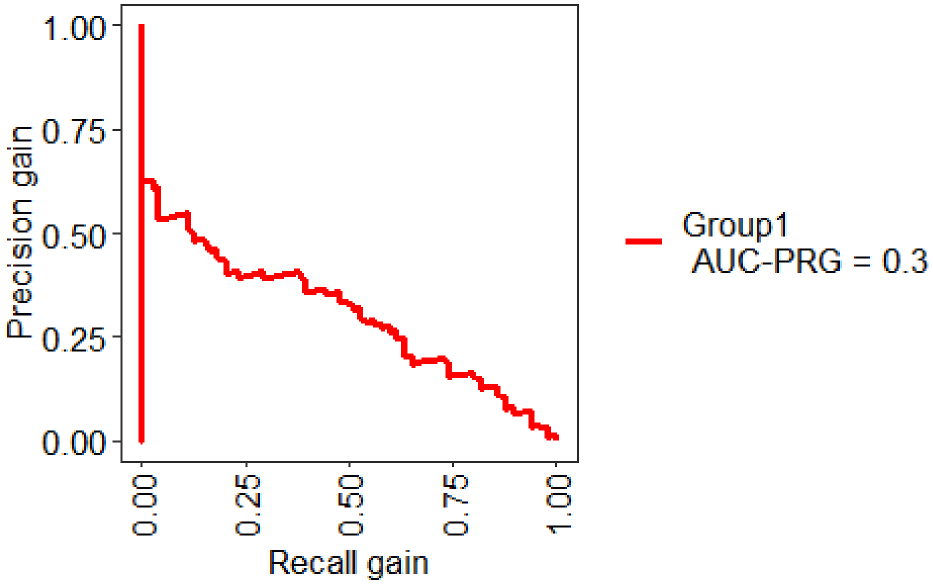

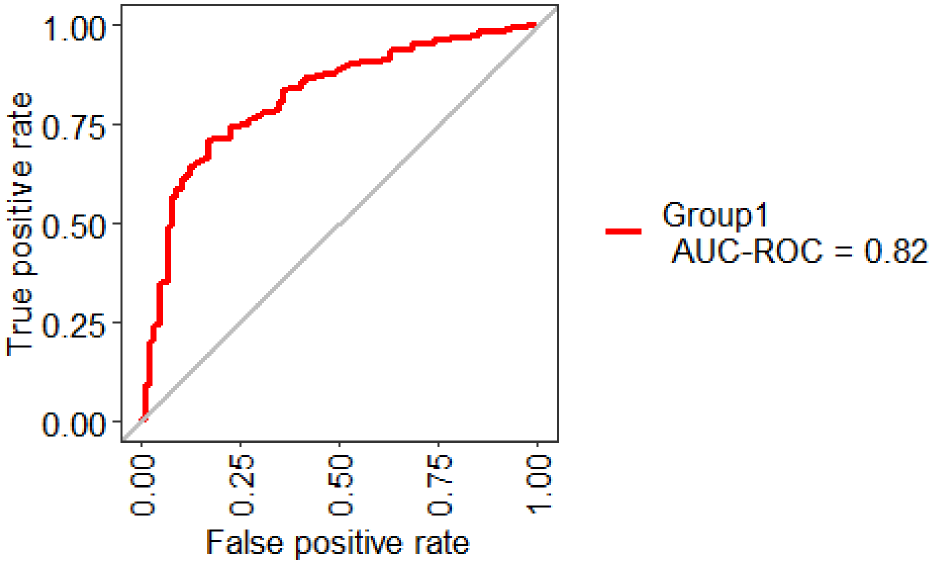


```
yadas2$stdres
```

```
## $Group1
##                   Score        CI
## SENS              0.880 0.83-0.92
## SPEC              0.528 0.43-0.63
## MCC               0.436      <NA>
## Informedness      0.408      <NA>
## PREC              0.813 0.76-0.86
## NPV               0.653 0.54-0.75
## FPR               0.472      <NA>
## F1                0.845      <NA>
## TP              183.000      <NA>
## FP               42.000      <NA>
## TN               47.000      <NA>
## FN               25.000      <NA>
## AUC-ROC           0.820 0.77-0.87
## AUC-PR            0.890      <NA>
## AUC-PRG           0.300      <NA>
```

```
imp_adas2<-varImp(rf_fit_adas2, scale = FALSE)
imp_adas2
```

```
## rf variable importance
##
##   only 20 most important variables shown (out of 60)
##
##                  Overall
## NIHSS_adm         84.779
## Age               43.061
## Arrival           24.087
## Hb                21.055
## RanGlu            20.460
## TG                19.011
## SBP               17.472
## BMI               17.302
## PLT               17.117
## HDL               17.080
## DBP               16.460
## TC                16.120
## BUN               15.892
## LDL               15.734
## HbA1C             15.447
## AC                15.115
## Cr                13.773
## Circulation.2      8.372
## Circulation.1      7.630
## Sex.Male           6.862
```

```
plot(imp_adas2)
```

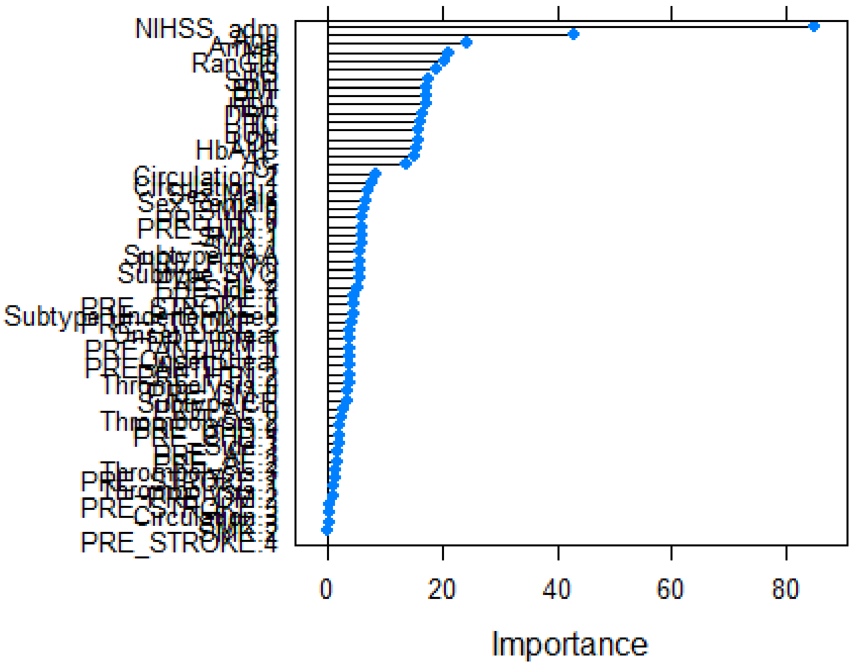


```
set.seed(seed)
rf_fit_adas3 <- train(class~., data = traindata.adas, method = "rf",
                      trControl=ctrl, metric=metric, tuneLength=10,
                      ntree=1500, verbose=FALSE)
rf_fit_adas3

## Random Forest
##
## 1092 samples
##   60 predictor
##    2 classes: 'unfavor', 'favor'
##
## No pre-processing
## Resampling: Cross-Validated (10 fold, repeated 10 times)
## Summary of sample sizes: 982, 984, 983, 983, 983, 982, ...
## Resampling results across tuning parameters:
##
##   mtry  ROC        Sens       Spec
##    2    0.9437309  0.8146201  0.8973550
##    8    0.9483870  0.8737110  0.8545388
##   14    0.9438076  0.8854383  0.8310901
##   21    0.9387098  0.8884935  0.8167680
##   27    0.9352906  0.8886494  0.8108106
##   34    0.9313092  0.8899448  0.8091160
##   40    0.9288040  0.8881299  0.8070894
##   47    0.9260637  0.8892175  0.8070755
```

```
##   53    0.9247415  0.8868961  0.8052131
##   60    0.9235761  0.8867240  0.8031586
##
## ROC was used to select the optimal model using the largest value.
## The final value used for the model was mtry = 8.

xadas3 <- evalm(rf_fit_adas3)
```

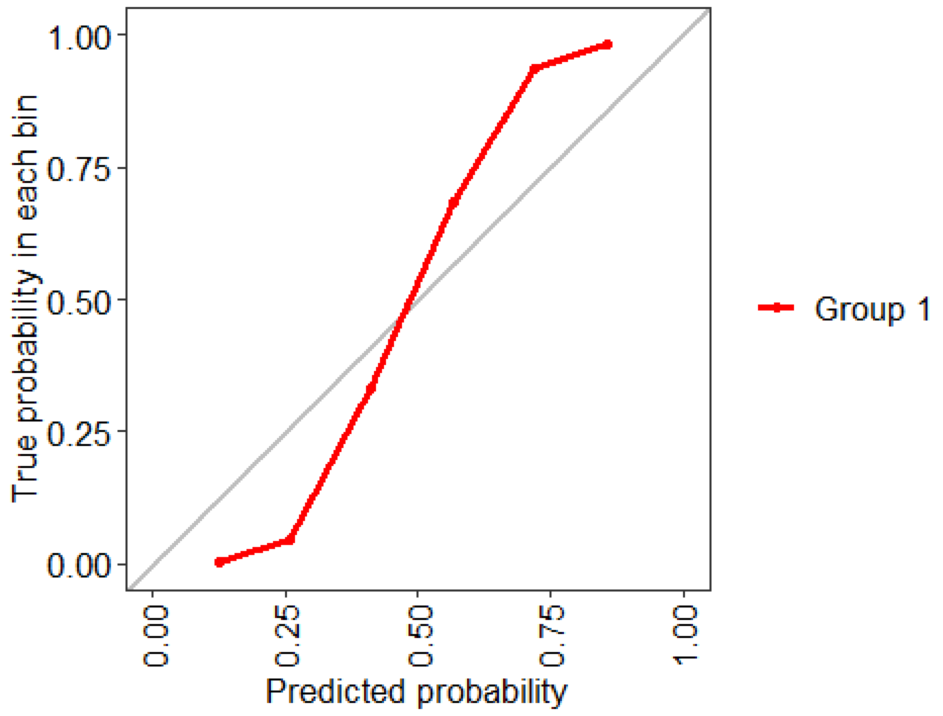

1.00
0.75
0.50
0.25
0.00
True probability in each bin
0.00
0.25
0.50
0.75
1.00
Predicted probability
Group 1

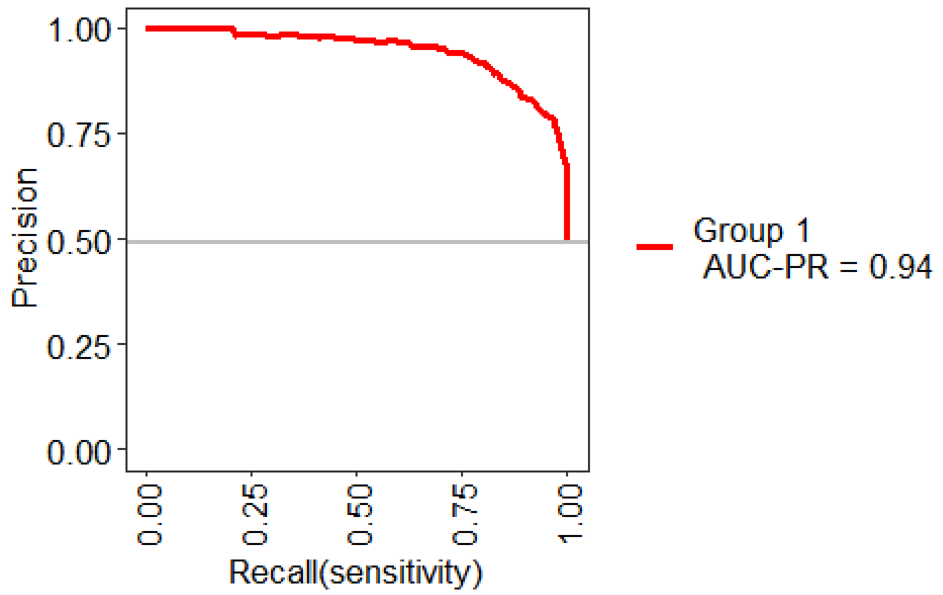

1.00
0.75
0.50
0.25
0.00
Precision
0.00
0.25
0.50
0.75
1.00
Recall(sensitivity)
Group 1
AUC-PR = 0.94

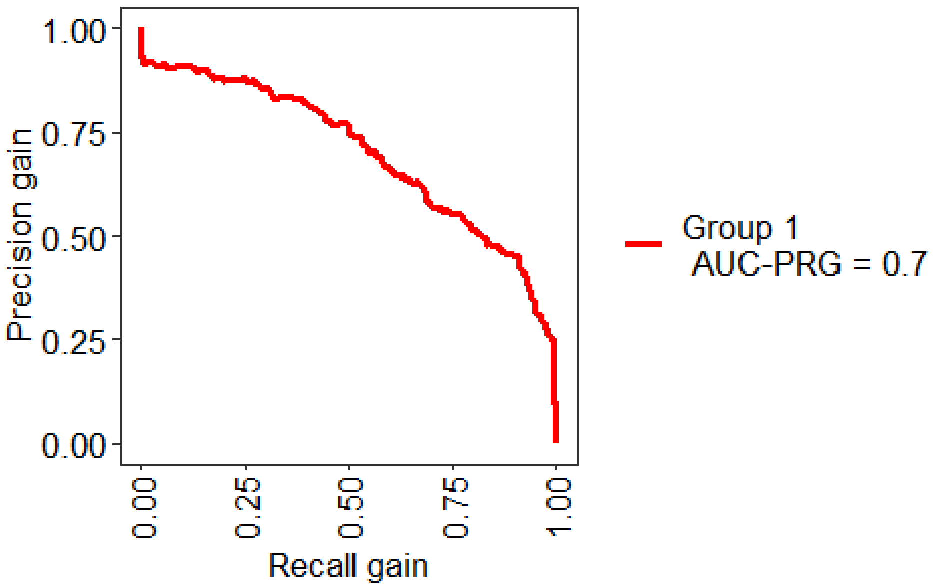
Precision gain
1.00
0.75
0.50
0.25
0.00
0.00
0.25
0.50
0.75
1.00
Recall gain
Group 1
AUC-PRG = 0.7

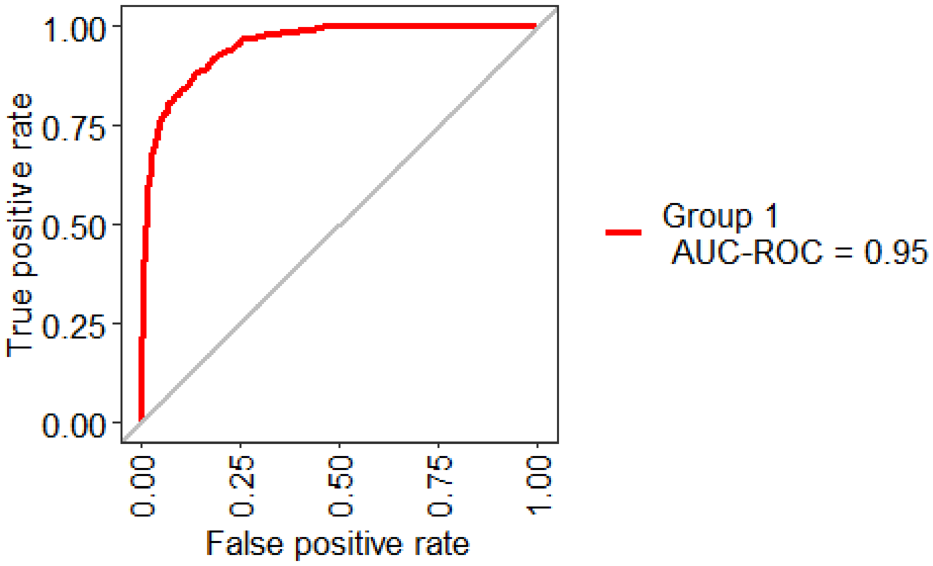
True positive rate
1.00
0.75
0.50
0.25
0.00
0.00
0.25
0.50
0.75
1.00
False positive rate
Group 1
AUC-ROC = 0.95

```
xadas3$roc
```

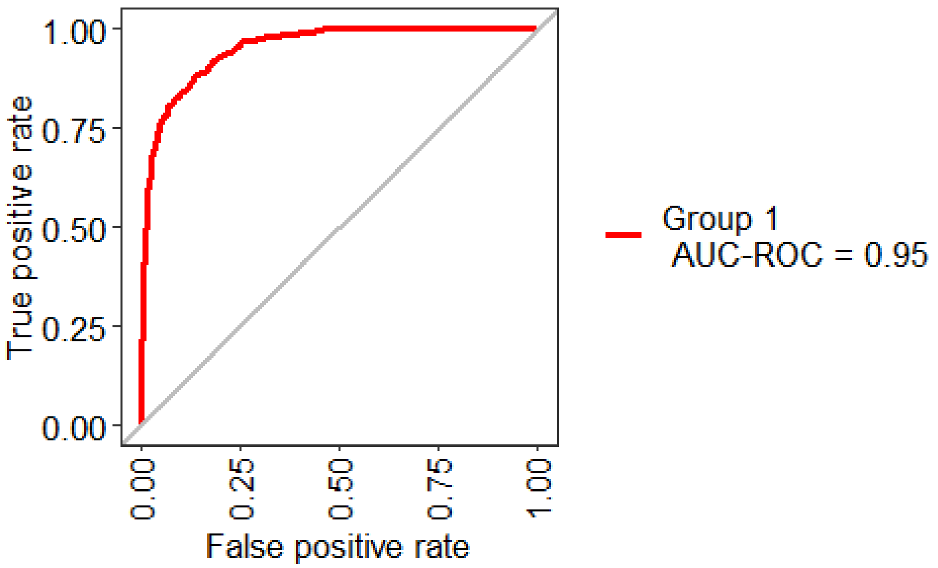


```
xadas3$stdres

## $`Group 1`
##                  Score        CI
## SENS             0.860 0.83-0.89
## SPEC             0.874  0.84-0.9
## MCC              0.734      <NA>
## Informedness     0.734      <NA>
## PREC             0.868 0.84-0.89
## NPV              0.866 0.84-0.89
## FPR              0.126      <NA>
## F1               0.864      <NA>
## TP             462.000      <NA>
## FP              70.000      <NA>
## TN             485.000      <NA>
## FN              75.000      <NA>
## AUC-ROC          0.950 0.94-0.96
## AUC-PR           0.940      <NA>
## AUC-PRG          0.700      <NA>

test_pred_rfadas3 <- predict(rf_fit_adas3, newdata = testdata)
confusionMatrix(test_pred_rfadas3, testdata$Outcome)

## Confusion Matrix and Statistics
##
##             Reference
```

```
## Prediction favor unfavor
##    favor     183      42
##    unfavor    25      47
##
##                Accuracy : 0.7744
##                  95% CI : (0.7226, 0.8207)
##     No Information Rate : 0.7003
##     P-Value [Acc > NIR] : 0.00266
##
##                   Kappa : 0.4315
##
##  Mcnemar's Test P-Value : 0.05062
##
##             Sensitivity : 0.8798
##             Specificity : 0.5281
##          Pos Pred Value : 0.8133
##          Neg Pred Value : 0.6528
##              Prevalence : 0.7003
##          Detection Rate : 0.6162
##    Detection Prevalence : 0.7576
##       Balanced Accuracy : 0.7039
##
##        'Positive' Class : favor
##

test_prob_adas3 <- predict(rf_fit_adas3, newdata = testdata, type="prob")
madas3 = data.frame(test_prob_adas3, testdata$Outcome)
yadas3<-evalm(madas3)
```

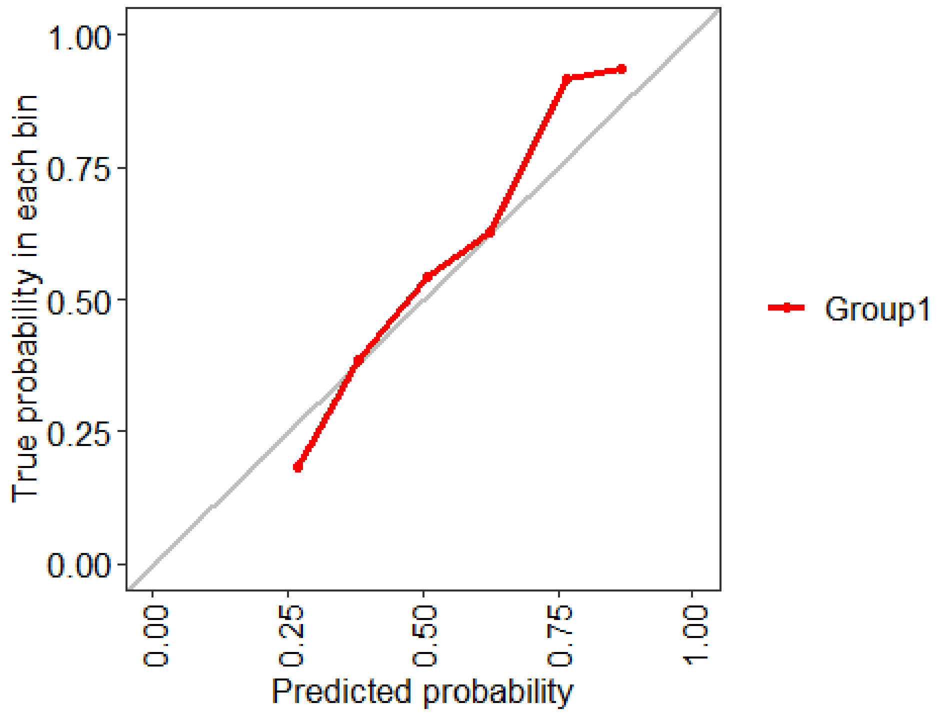
1.00
0.75
0.50
0.25
0.00
True probability in each bin
0.00
0.25
0.50
0.75
1.00
Predicted probability
Group1

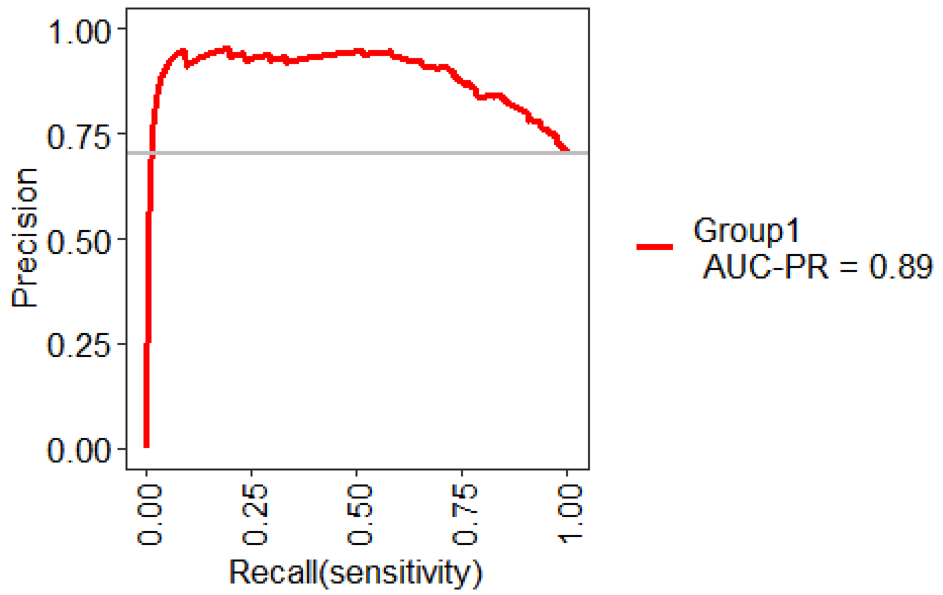
1.00
0.75
0.50
0.25
0.00
Precision
0.00
0.25
0.50
0.75
1.00
Recall(sensitivity)
Group1
AUC-PR = 0.89

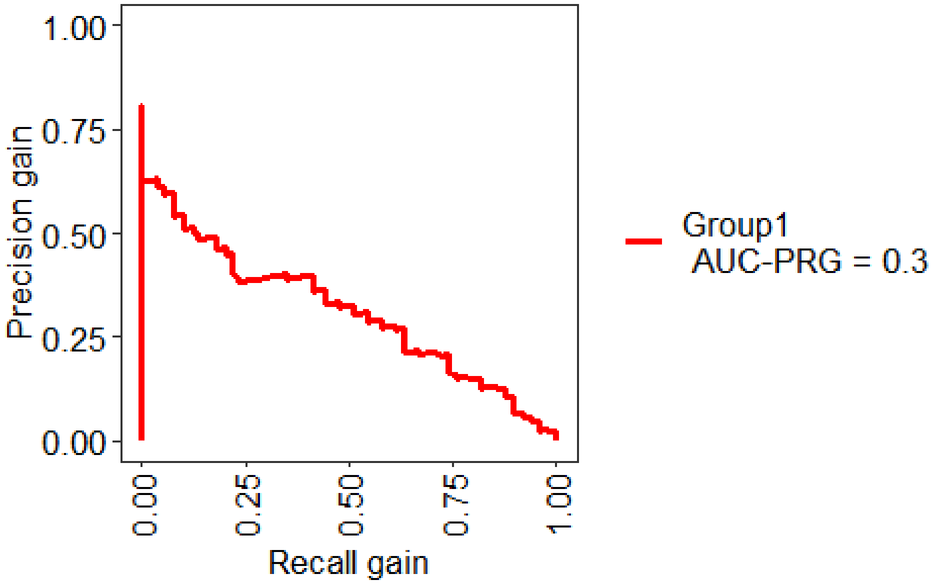

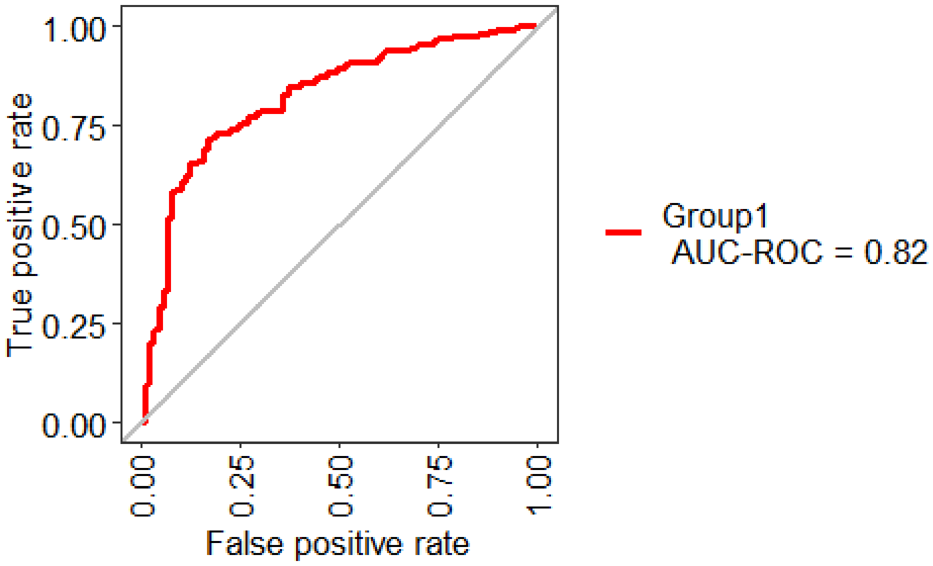


```
yadas3$stdres
```

```
## $Group1
##                   Score        CI
## SENS              0.885 0.83-0.92
## SPEC              0.528 0.43-0.63
## MCC               0.443      <NA>
## Informedness      0.413      <NA>
## PREC              0.814 0.76-0.86
## NPV               0.662 0.55-0.76
## FPR               0.472      <NA>
## F1                0.848      <NA>
## TP              184.000      <NA>
## FP               42.000      <NA>
## TN               47.000      <NA>
## FN               24.000      <NA>
## AUC-ROC           0.820 0.77-0.87
## AUC-PR            0.890      <NA>
## AUC-PRG           0.300      <NA>
```

```
imp_adas3<-varImp(rf_fit_adas3, scale = FALSE)
imp_adas3
```

```
## rf variable importance
##
##   only 20 most important variables shown (out of 60)
##
##                  Overall
## NIHSS_adm         84.293
## Age               43.021
## Arrival           24.255
## Hb                21.021
## RanGlu            20.518
## TG                19.182
## BMI               17.344
## PLT               17.298
## SBP               17.096
## HDL               17.020
## DBP               16.209
## TC                16.083
## LDL               16.077
## BUN               16.012
## AC                15.205
## HbA1C             15.159
## Cr                14.000
## Circulation.2      8.376
## Circulation.1      7.673
## Sex.Male           6.848
```

```
plot(imp_adas3)
```

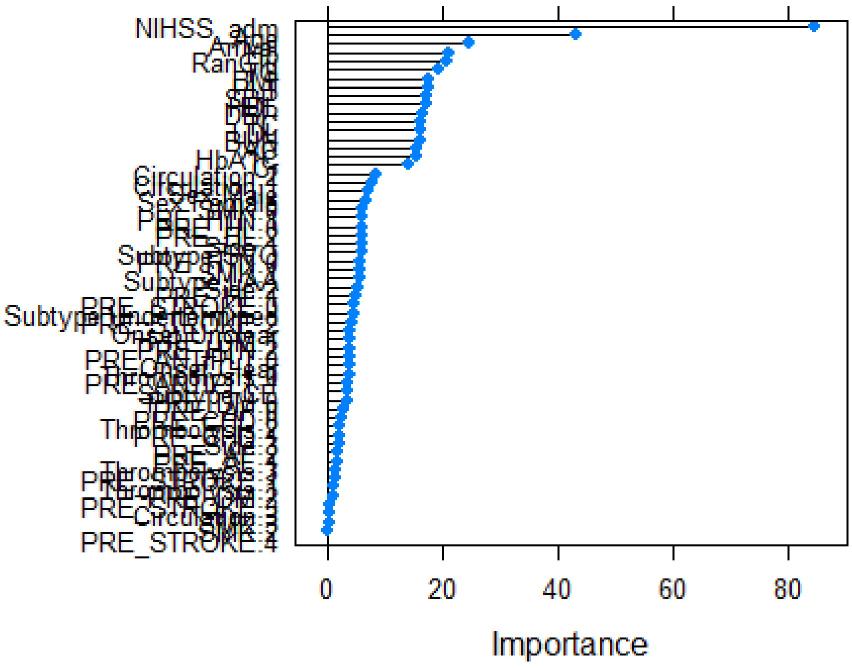


```
set.seed(seed)
xgb_fit_adas <- train(class~., data = traindata.adas, method = "xgbTree",
                      trControl=ctrl, metric=metric, tuneGrid=xgbgrid)
xgb_fit_adas

## eXtreme Gradient Boosting
##
## 1092 samples
##   60 predictor
##    2 classes: 'unfavor', 'favor'
##
## No pre-processing
## Resampling: Cross-Validated (10 fold, repeated 10 times)
## Summary of sample sizes: 982, 984, 983, 983, 983, 982, ...
## Resampling results across tuning parameters:
##
##   eta   max_depth  nrounds  ROC        Sens       Spec
##   0.01  6           500     0.9362709  0.8666753  0.8312474
##   0.01  6          1000     0.9398508  0.8659740  0.8405835
##   0.01  6          1500     0.9404205  0.8672370  0.8413347
##   0.01  8           500     0.9401724  0.8728214  0.8359050
##   0.01  8          1000     0.9424091  0.8692110  0.8413242
##   0.01  8          1500     0.9424251  0.8695649  0.8417016
##   0.10  6           500     0.9359121  0.8598442  0.8357932
##   0.10  6          1000     0.9344892  0.8571526  0.8359539
##   0.10  6          1500     0.9333332  0.8553571  0.8367156
```

```
##   0.10  8           500     0.9375362  0.8627240  0.8388784
##   0.10  8          1000     0.9357828  0.8582305  0.8392662
##   0.10  8          1500     0.9348424  0.8546234  0.8366667
##
## Tuning parameter 'gamma' was held constant at a value of 0
## Tuning
##
## Tuning parameter 'min_child_weight' was held constant at a value of 1
##
## Tuning parameter 'subsample' was held constant at a value of 0.8
## ROC was used to select the optimal model using the largest value.
## The final values used for the model were nrounds = 1500, max_depth = 8, et
a
##  = 0.01, gamma = 0, colsample_bytree = 0.6, min_child_weight = 1 and
##  subsample = 0.8.
```

```
x_xgbadas <- evalm(xgb_fit_adas)
```

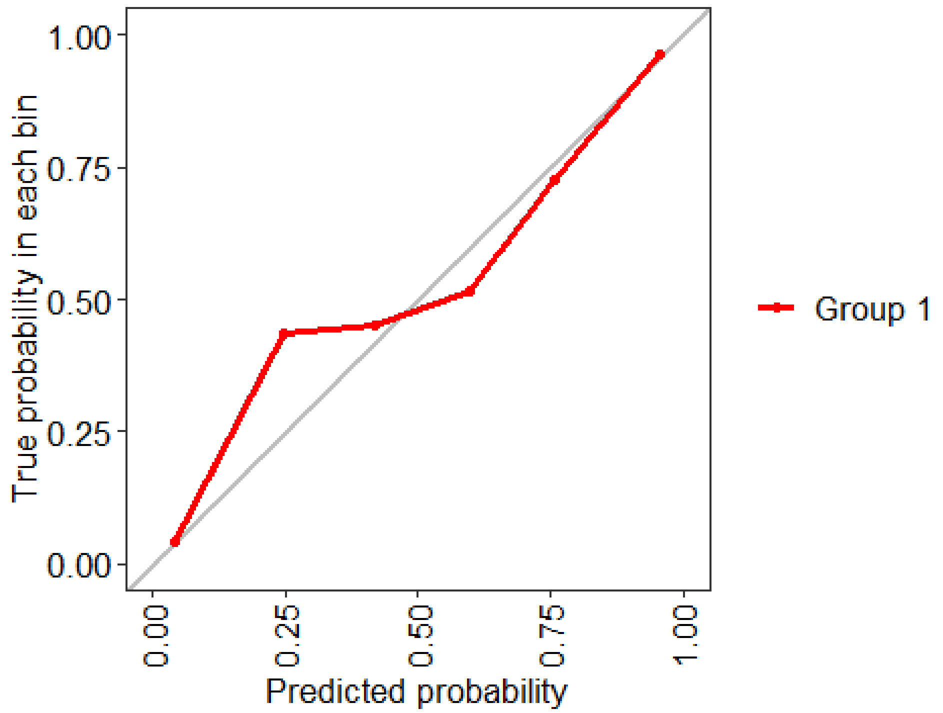
1.00
0.75
0.50
0.25
0.00
True probability in each bin
0.00
0.25
0.50
0.75
1.00
Predicted probability
Group 1

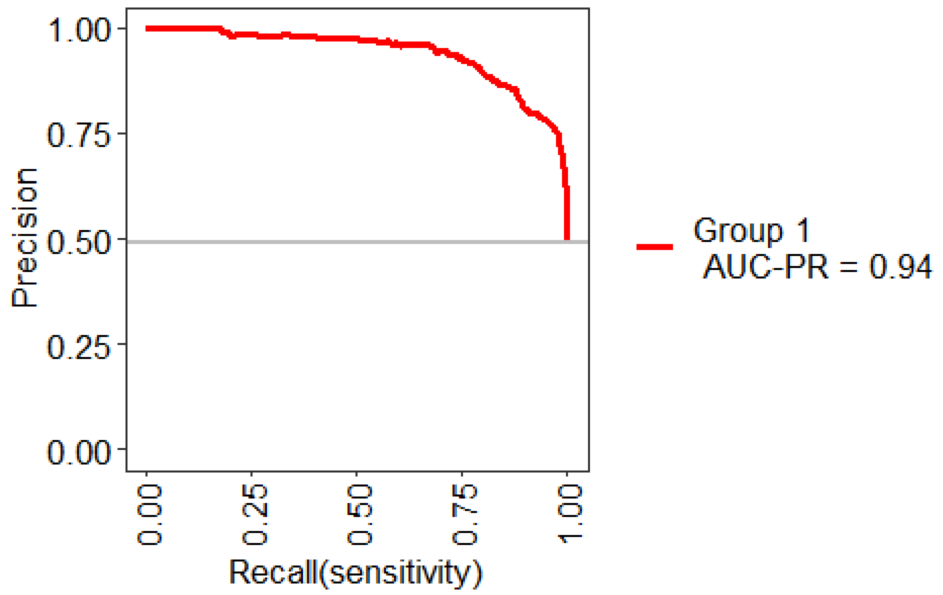
1.00
0.75
0.50
0.25
0.00
Precision
0.00
0.25
0.50
0.75
1.00
Recall(sensitivity)
Group 1
AUC-PR = 0.94

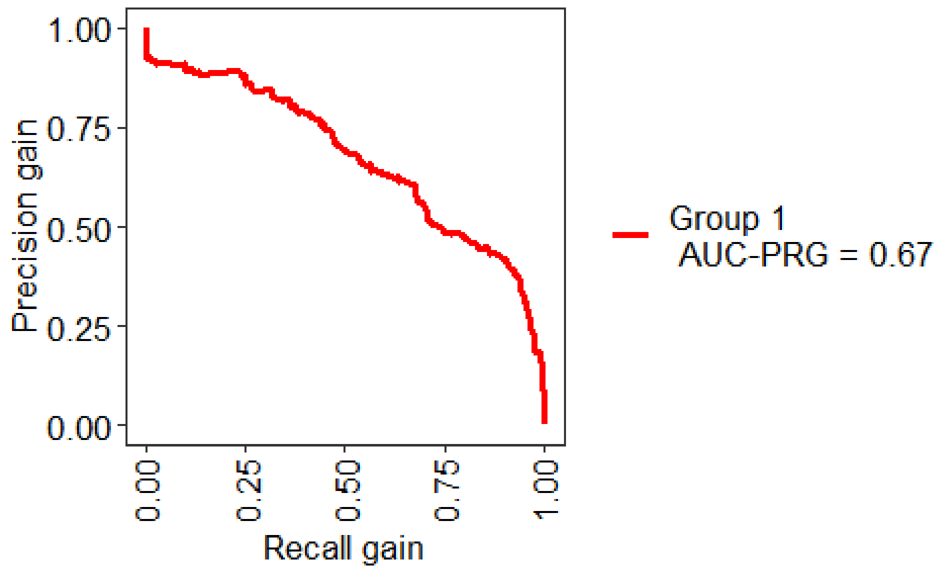

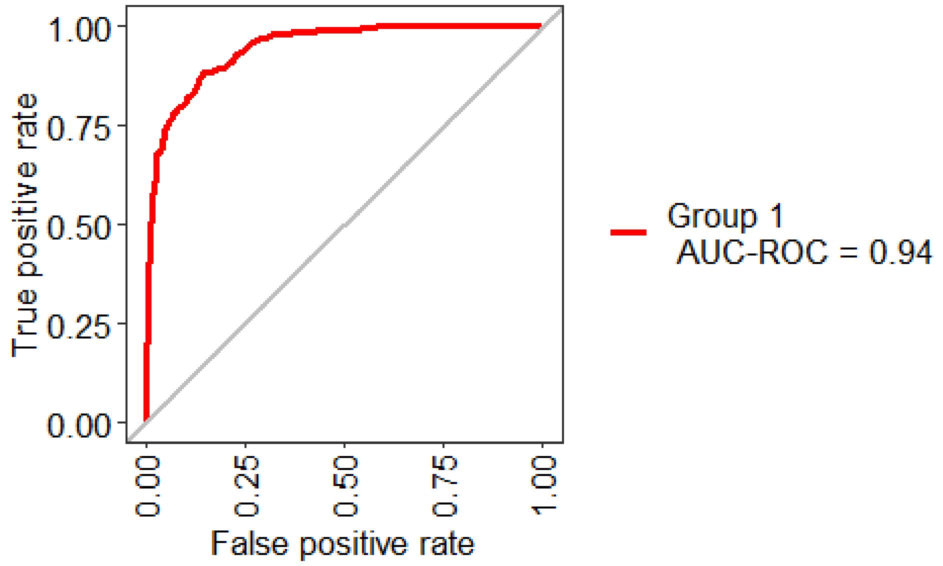


```
x_xgbadas$stdres
```

```
## $`Group 1`
##                    Score        CI
## SENS               0.836  0.8-0.87
## SPEC               0.876  0.85-0.9
## MCC                0.713      <NA>
## Informedness       0.712      <NA>
## PREC               0.867 0.83-0.89
## NPV                0.847 0.81-0.87
## FPR                0.124      <NA>
## F1                 0.851      <NA>
## TP               449.000      <NA>
## FP                69.000      <NA>
## TN               486.000      <NA>
## FN                88.000      <NA>
## AUC-ROC            0.940 0.93-0.95
## AUC-PR             0.940      <NA>
## AUC-PRG            0.670      <NA>
```

```
test_pred_xgbadas <- predict(xgb_fit_adas, newdata = testdata)
confusionMatrix(test_pred_xgbadas, testdata$Outcome)
```

```
## Confusion Matrix and Statistics
## 
##           Reference
## Prediction favor unfavor
##    favor     173      34
##    unfavor    35      55
## 
##                Accuracy : 0.7677
##                  95% CI : (0.7154, 0.8145)
##     No Information Rate : 0.7003
##     P-Value [Acc > NIR] : 0.005869
## 
##                   Kappa : 0.4483
## 
##  Mcnemar's Test P-Value : 1.000000
## 
##             Sensitivity : 0.8317
##             Specificity : 0.6180
##          Pos Pred Value : 0.8357
##          Neg Pred Value : 0.6111
##              Prevalence : 0.7003
##          Detection Rate : 0.5825
##    Detection Prevalence : 0.6970
##       Balanced Accuracy : 0.7249
## 
##        'Positive' Class : favor
## 
```

```
test_prob_xgbadas <- predict(xgb_fit_adas, newdata = testdata, type="prob")
mxgbadas = data.frame(test_prob_xgbadas, testdata$Outcome)
yxgbadas<-evalm(mxgbadas)
```

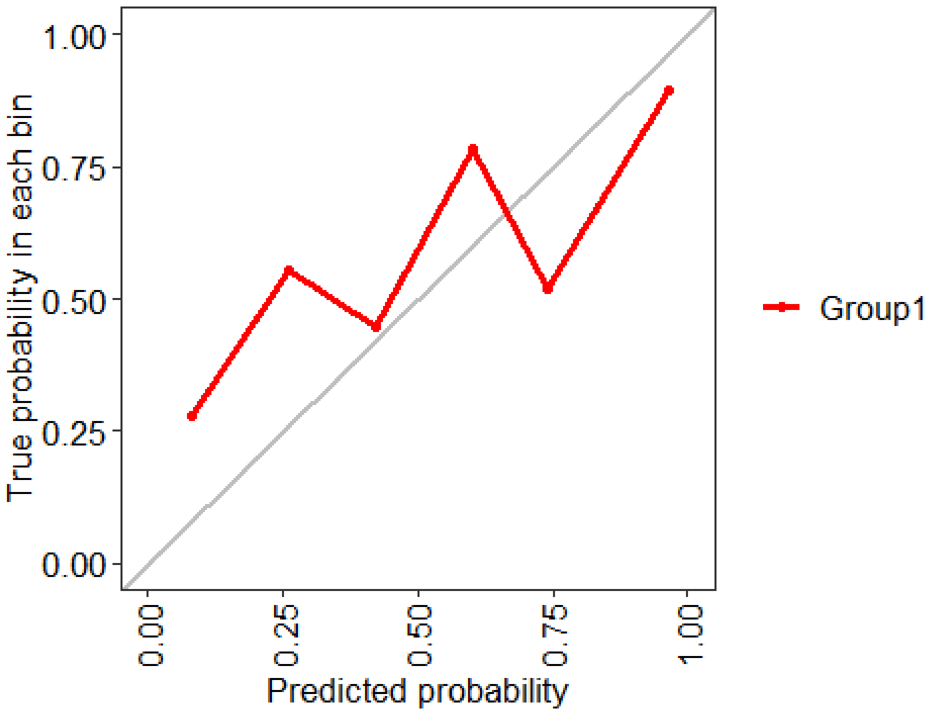

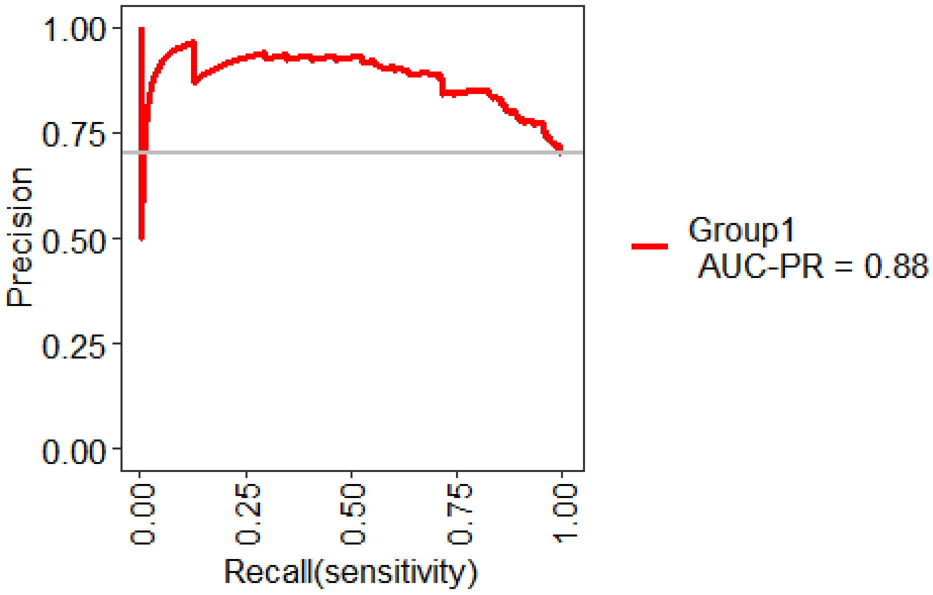

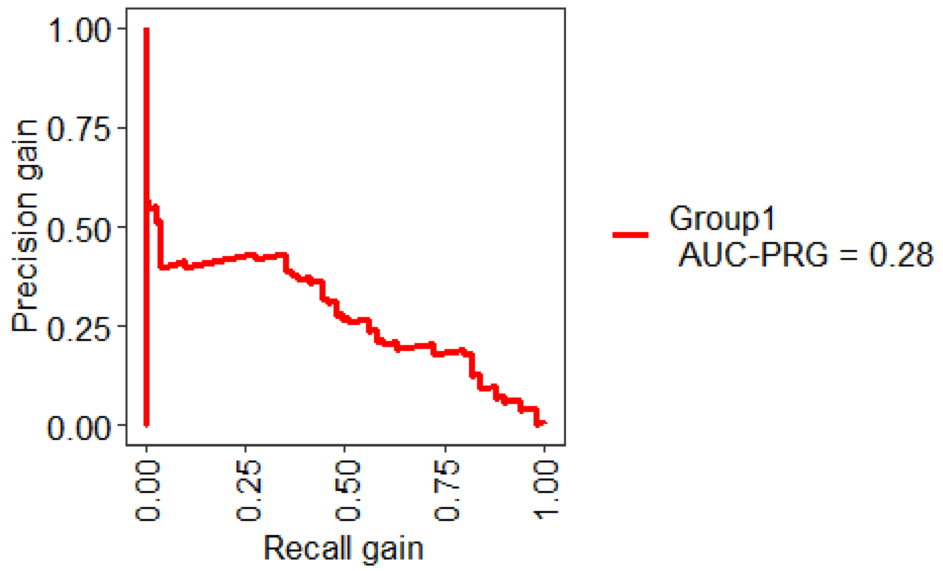

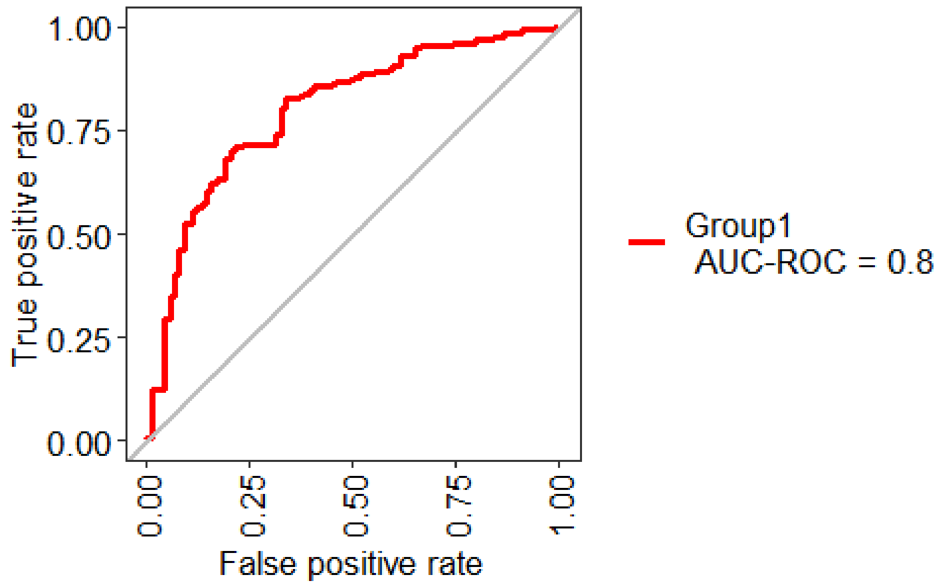


```
yxgbadas$stdres
```

```
## $Group1
##                   Score        CI
## SENS              0.832 0.77-0.88
## SPEC              0.618 0.51-0.71
## MCC               0.448      <NA>
## Informedness      0.450      <NA>
## PREC              0.836 0.78-0.88
## NPV               0.611 0.51-0.71
## FPR               0.382      <NA>
## F1                0.834      <NA>
## TP              173.000      <NA>
## FP               34.000      <NA>
## TN               55.000      <NA>
## FN               35.000      <NA>
## AUC-ROC           0.800 0.75-0.85
## AUC-PR            0.880      <NA>
## AUC-PRG           0.280      <NA>
```

```
imp_adas<-varImp(xgb_fit_adas, scale = FALSE)
imp_adas
```

```
## xgbTree variable importance
##
##   only 20 most important variables shown (out of 60)
##
##                  Overall
## NIHSS_adm        0.19861
## Age              0.09764
## Arrival          0.05139
## RanGlu           0.04151
## PLT              0.03591
## SBP              0.03586
## Hb               0.03506
## DBP              0.03330
## TG               0.03266
## BMI              0.03204
## LDL              0.02924
## HDL              0.02866
## TC               0.02770
## BUN              0.02665
## Cr               0.02543
## HbA1C            0.02504
## AC               0.02124
## Side.1           0.01551
## Circulation.1    0.01490
## Sex.Female       0.01450
```

```
plot(imp_adas)
```

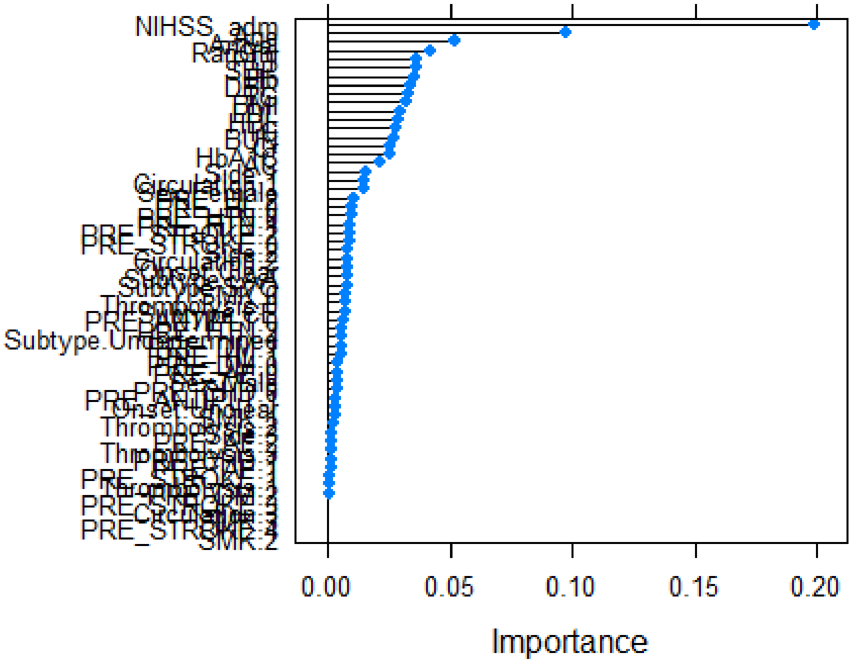


```
set.seed(seed)
svm_fit_adas <- train(class~., data = traindata.adas, method = "svmRadial",
                      trControl=ctrl, metric=metric, tuneLength = 10)
svm_fit_adas
```

```
## Support Vector Machines with Radial Basis Function Kernel
##
## 1092 samples
##   60 predictor
##    2 classes: 'unfavor', 'favor'
##
## No pre-processing
## Resampling: Cross-Validated (10 fold, repeated 10 times)
## Summary of sample sizes: 982, 984, 983, 983, 983, 982, ...
## Resampling results across tuning parameters:
##
##   C      ROC        Sens       Spec
##    0.25  0.8200145  0.7463799  0.7335604
##    0.50  0.8498604  0.7922922  0.7594340
##    1.00  0.8801814  0.8225519  0.7914605
##    2.00  0.9075508  0.8612987  0.8149266
##    4.00  0.9300968  0.8883182  0.8368728
##    8.00  0.9461839  0.9147857  0.8523096
##   16.00  0.9491871  0.9247078  0.8551572
##   32.00  0.9490927  0.9229091  0.8515968
##   64.00  0.9491760  0.9243539  0.8513941
```

```
##   128.00  0.9491760  0.9243474  0.8493501
##
## Tuning parameter 'sigma' was held constant at a value of 0.03255705
## ROC was used to select the optimal model using the largest value.
## The final values used for the model were sigma = 0.03255705 and C = 16.
```

```
x_svmadas <- evalm(svm_fit_adas)
```

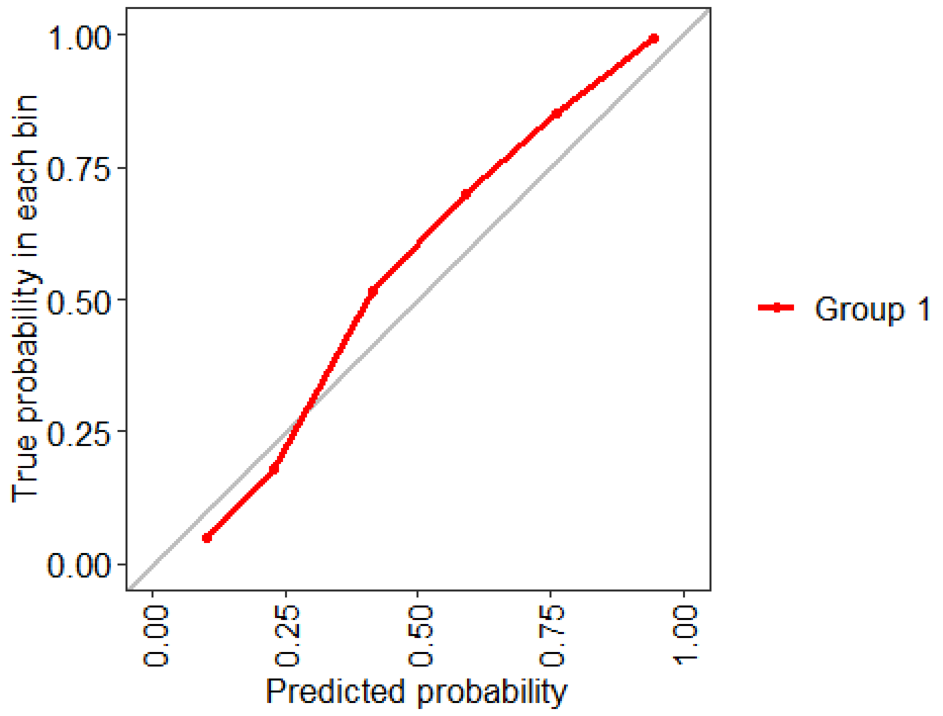
1.00
0.75
0.50
0.25
0.00
True probability in each bin
0.00
0.25
0.50
0.75
1.00
Predicted probability
Group 1

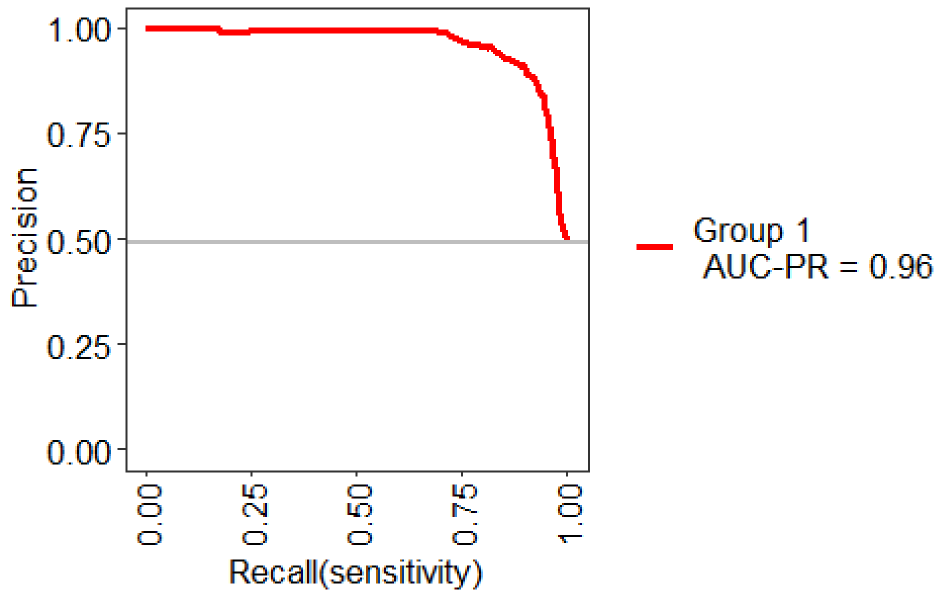
1.00
0.75
0.50
0.25
0.00
Precision
0.00
0.25
0.50
0.75
1.00
Recall(sensitivity)
Group 1
AUC-PR = 0.96

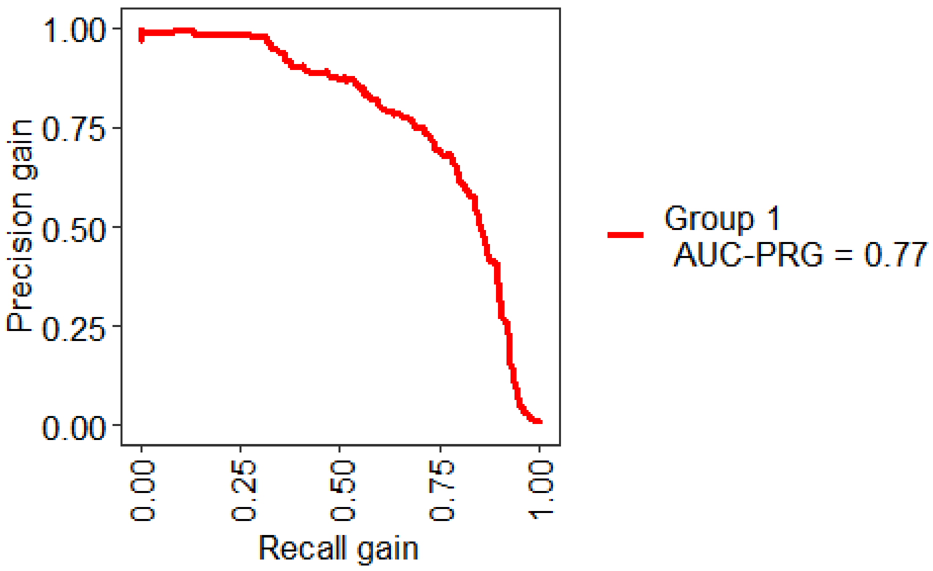

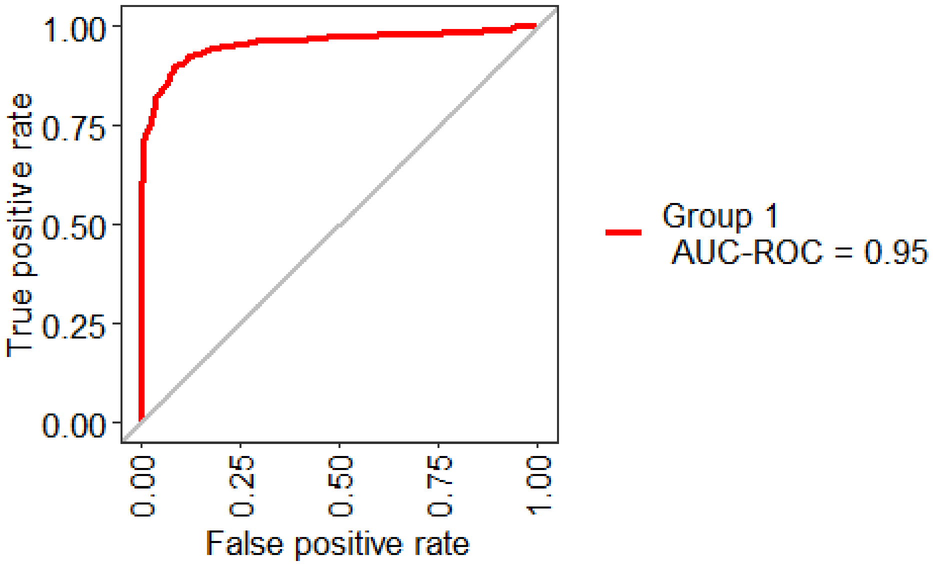


```
x_svmadas$stdres
```

```
## $`Group 1`
##                  Score        CI
## SENS             0.860 0.83-0.89
## SPEC             0.933 0.91-0.95
## MCC              0.797      <NA>
## Informedness     0.794      <NA>
## PREC             0.926  0.9-0.95
## NPV              0.874  0.84-0.9
## FPR              0.067      <NA>
## F1               0.892      <NA>
## TP             462.000      <NA>
## FP              37.000      <NA>
## TN             518.000      <NA>
## FN              75.000      <NA>
## AUC-ROC          0.950 0.94-0.96
## AUC-PR           0.960      <NA>
## AUC-PRG          0.770      <NA>
```

```
test_pred_svmadas <- predict(svm_fit_adas, newdata = testdata)
confusionMatrix (test_pred_svmadas, testdata$Outcome)
```

```
## Confusion Matrix and Statistics
##
##           Reference
## Prediction favor unfavor
##    favor     183      56
##    unfavor    25      33
##
##                Accuracy : 0.7273
##                  95% CI : (0.6728, 0.7771)
##     No Information Rate : 0.7003
##     P-Value [Acc > NIR] : 0.1712592
##
##                   Kappa : 0.2783
##
##  Mcnemar's Test P-Value : 0.0008581
##
##             Sensitivity : 0.8798
##             Specificity : 0.3708
##          Pos Pred Value : 0.7657
##          Neg Pred Value : 0.5690
##              Prevalence : 0.7003
##          Detection Rate : 0.6162
##    Detection Prevalence : 0.8047
##       Balanced Accuracy : 0.6253
##
##        'Positive' Class : favor
##
```

```
test_prob_svmadas <- predict(svm_fit_adas, newdata = testdata, type="prob")
msvmadas = data.frame(test_prob_svmadas, testdata$Outcome)
ysvmadas<-evalm(msvmadas)
```

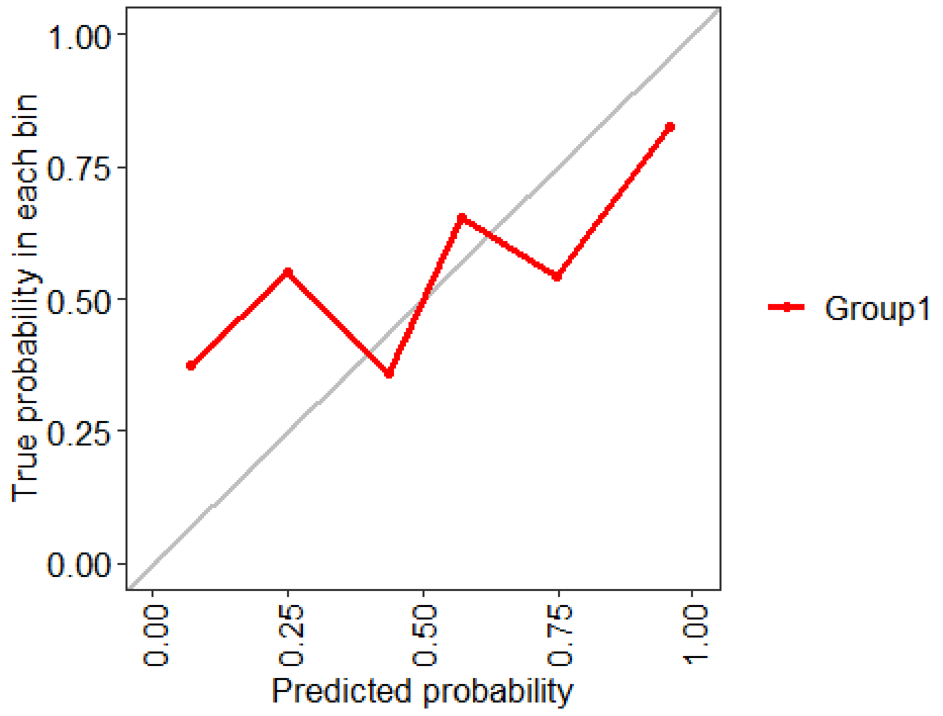

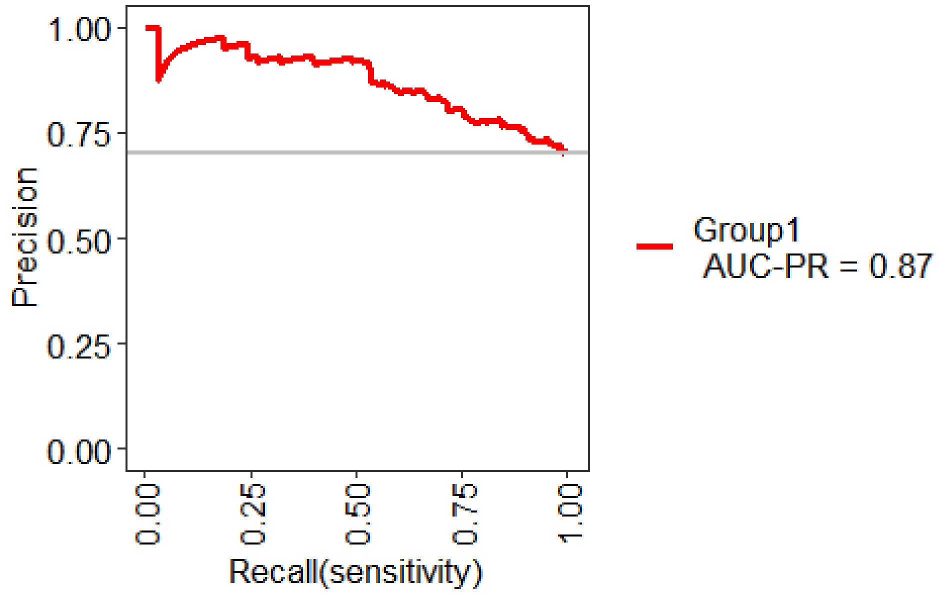

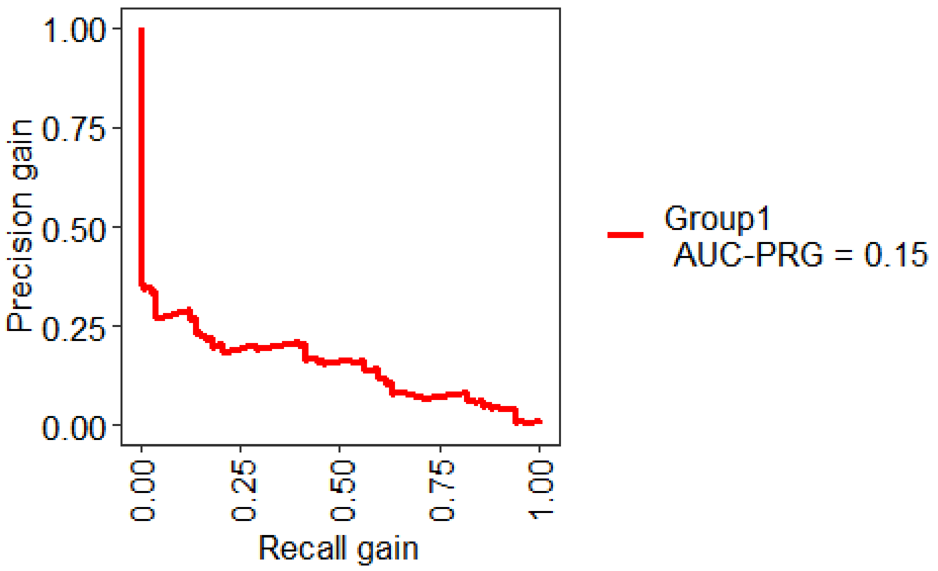

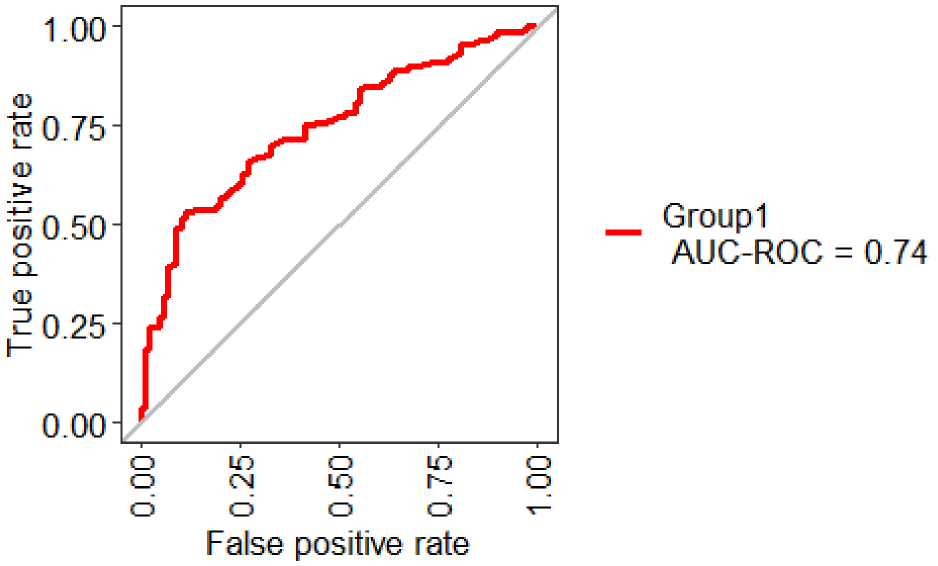


```
ysvmadas$stdres
```

```
## $Group1
##                    Score        CI
## SENS               0.880 0.83-0.92
## SPEC               0.371 0.28-0.47
## MCC                0.290      <NA>
## Informedness       0.251      <NA>
## PREC               0.766 0.71-0.81
## NPV                0.569 0.44-0.69
## FPR                0.629      <NA>
## F1                 0.819      <NA>
## TP               183.000      <NA>
## FP                56.000      <NA>
## TN                33.000      <NA>
## FN                25.000      <NA>
## AUC-ROC            0.740  0.68-0.8
## AUC-PR             0.870      <NA>
## AUC-PRG            0.150      <NA>
```

```
set.seed(seed)
knn_fit_adas <- train(class~., data = traindata.adas, method = "knn",
                      trControl=ctrl, metric=metric, tuneLength = 50)
knn_fit_adas
```

```
## k-Nearest Neighbors
##
## 1092 samples
##   60 predictor
##    2 classes: 'unfavor', 'favor'
##
## No pre-processing
## Resampling: Cross-Validated (10 fold, repeated 10 times)
## Summary of sample sizes: 982, 984, 983, 983, 983, 982, ...
## Resampling results across tuning parameters:
##
##   k    ROC        Sens       Spec
##     5  0.7960040  0.9495325  0.4555765
##     7  0.7764053  0.9408669  0.4270231
##     9  0.7724186  0.9370812  0.4044829
##    11  0.7631439  0.9387078  0.3838190
##    13  0.7602684  0.9338701  0.3733857
##    15  0.7547646  0.9265032  0.3660971
##    17  0.7458261  0.9171396  0.3611076
##    19  0.7435190  0.9140942  0.3551328
##    21  0.7439380  0.9151721  0.3532739
##    23  0.7421574  0.9155325  0.3462089
##    25  0.7405973  0.9207500  0.3400559
##    27  0.7406208  0.9227338  0.3313208
##    29  0.7425156  0.9210974  0.3275821
##    31  0.7410642  0.9228896  0.3255311
```

```
##    33  0.7425580  0.9234351  0.3234976
##    35  0.7413471  0.9196494  0.3190252
##    37  0.7408390  0.9198214  0.3151188
##    39  0.7385125  0.9179968  0.3136198
##    41  0.7383945  0.9192695  0.3048532
##    43  0.7379461  0.9185487  0.3013627
##    45  0.7361045  0.9185390  0.2931586
##    47  0.7352218  0.9207143  0.2894584
##    49  0.7354411  0.9239610  0.2874004
##    51  0.7346885  0.9259383  0.2883019
##    53  0.7341101  0.9248442  0.2858980
##    55  0.7340553  0.9241364  0.2844130
##    57  0.7340744  0.9268312  0.2795388
##    59  0.7353202  0.9281136  0.2801083
##    61  0.7347136  0.9277370  0.2787806
##    63  0.7332101  0.9277208  0.2754263
##    65  0.7310903  0.9257500  0.2743117
##    67  0.7298167  0.9282597  0.2733718
##    69  0.7298059  0.9280844  0.2741125
##    71  0.7295168  0.9275227  0.2770894
##    73  0.7282963  0.9259188  0.2758001
##    75  0.7274917  0.9206883  0.2744934
##    77  0.7261828  0.9197857  0.2739308
##    79  0.7254983  0.9161753  0.2748637
##    81  0.7248028  0.9125877  0.2744864
##    83  0.7242829  0.9098831  0.2745038
##    85  0.7233342  0.9068117  0.2776415
##    87  0.7217892  0.9046558  0.2811845
##    89  0.7200454  0.9024805  0.2819182
##    91  0.7183580  0.9019513  0.2845353
##    93  0.7168714  0.9026526  0.2884416
##    95  0.7159436  0.9031981  0.2901188
##    97  0.7156314  0.9021169  0.2923550
##    99  0.7152049  0.9030325  0.2930887
##   101  0.7144363  0.9028571  0.2960727
##   103  0.7157263  0.9017825  0.2960622
##
## ROC was used to select the optimal model using the largest value.
## The final value used for the model was k = 5.
```

```
x_knnadas <- evalm(knn_fit_adas)
```

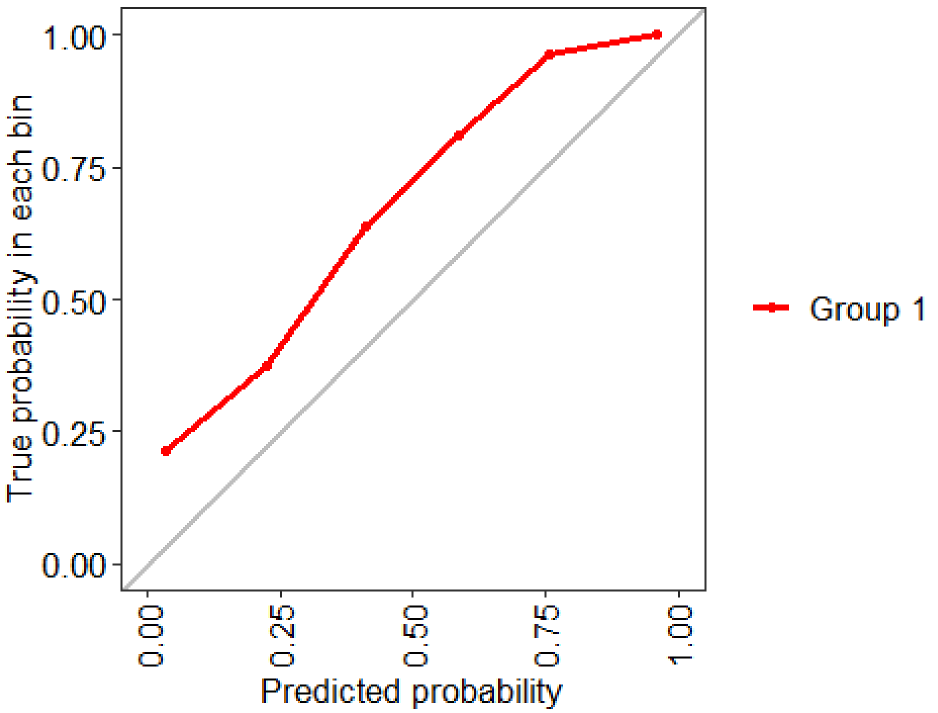

1.00
0.75
0.50
0.25
0.00
True probability in each bin
0.00
0.25
0.50
0.75
1.00
Predicted probability
Group 1

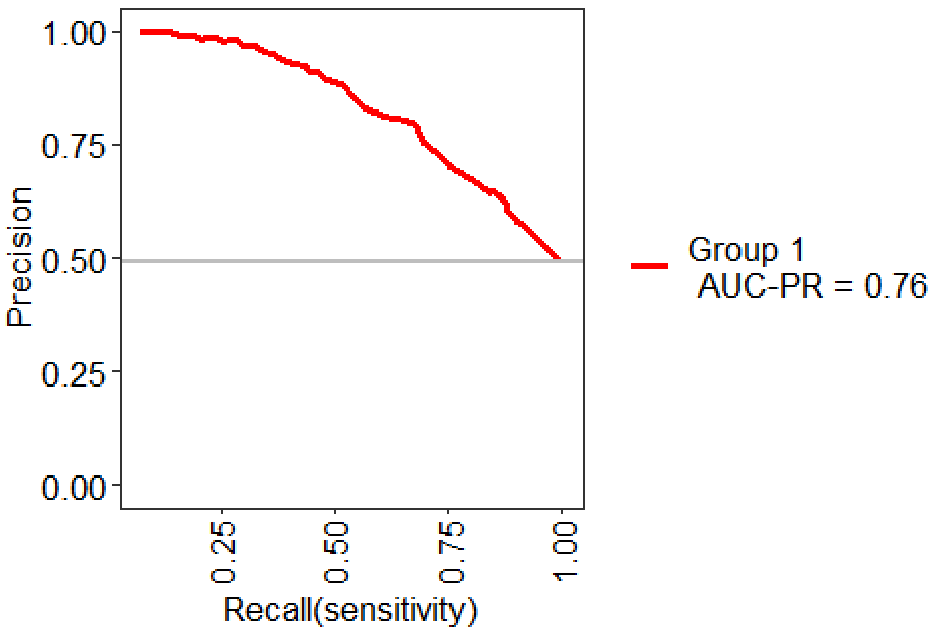

1.00
0.75
0.50
0.25
0.00
Precision
0.25
0.50
0.75
1.00
Recall(sensitivity)
Group 1
AUC-PR = 0.76

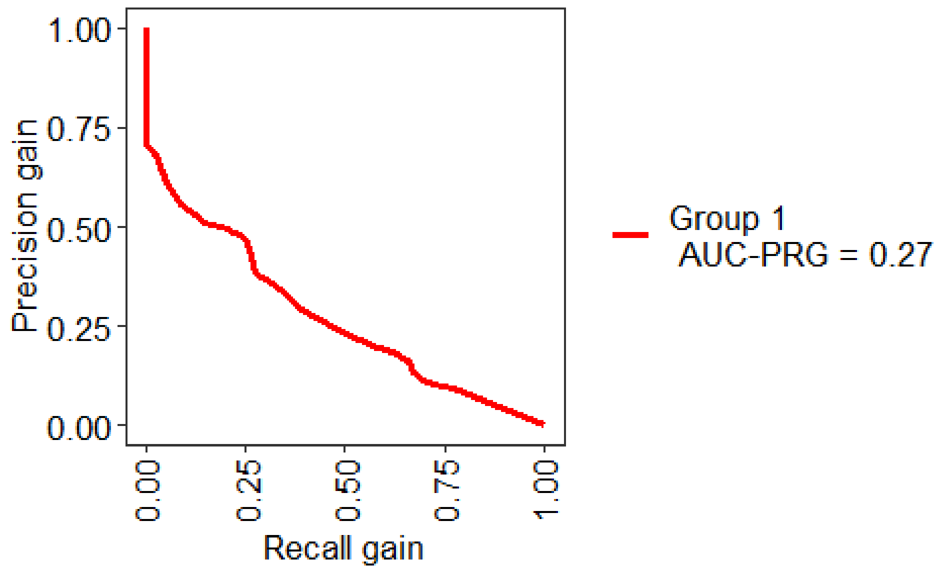

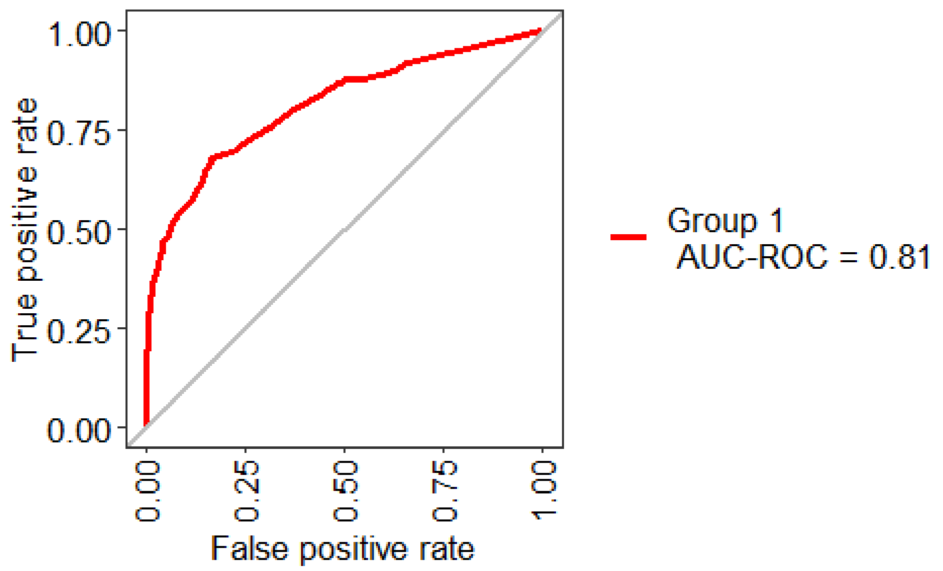


```
x_knnadas$stdres
```

```
## $`Group 1`
##                   Score       CI
## SENS              0.462  0.42-0.5
## SPEC              0.957 0.94-0.97
## MCC               0.484      <NA>
## Informedness      0.419      <NA>
## PREC              0.912 0.87-0.94
## NPV               0.648 0.61-0.68
## FPR               0.043      <NA>
## F1                0.613      <NA>
## TP              248.000      <NA>
## FP               24.000      <NA>
## TN              531.000      <NA>
## FN              289.000      <NA>
## AUC-ROC           0.810 0.78-0.84
## AUC-PR            0.760      <NA>
## AUC-PRG           0.270      <NA>
```

```
test_pred_knnadas <- predict(knn_fit_adas, newdata = testdata)
confusionMatrix(test_pred_knnadas, testdata$Outcome)
```

```
## Confusion Matrix and Statistics
##
##           Reference
## Prediction favor unfavor
##    favor     100      36
##    unfavor   108      53
##
##                Accuracy : 0.5152
##                  95% CI : (0.4567, 0.5733)
##     No Information Rate : 0.7003
##     P-Value [Acc > NIR] : 1
##
##                   Kappa : 0.0619
##
##  Mcnemar's Test P-Value : 3.285e-09
##
##             Sensitivity : 0.4808
##             Specificity : 0.5955
##          Pos Pred Value : 0.7353
##          Neg Pred Value : 0.3292
##              Prevalence : 0.7003
##          Detection Rate : 0.3367
##    Detection Prevalence : 0.4579
##       Balanced Accuracy : 0.5381
##
##        'Positive' Class : favor
##
```

```
test_prob_knnadas <- predict(knn_fit_adas, newdata = testdata, type="prob")
mknnadas = data.frame(test_prob_knnadas, testdata$Outcome)
yknnadas<-evalm(mknnadas)
```

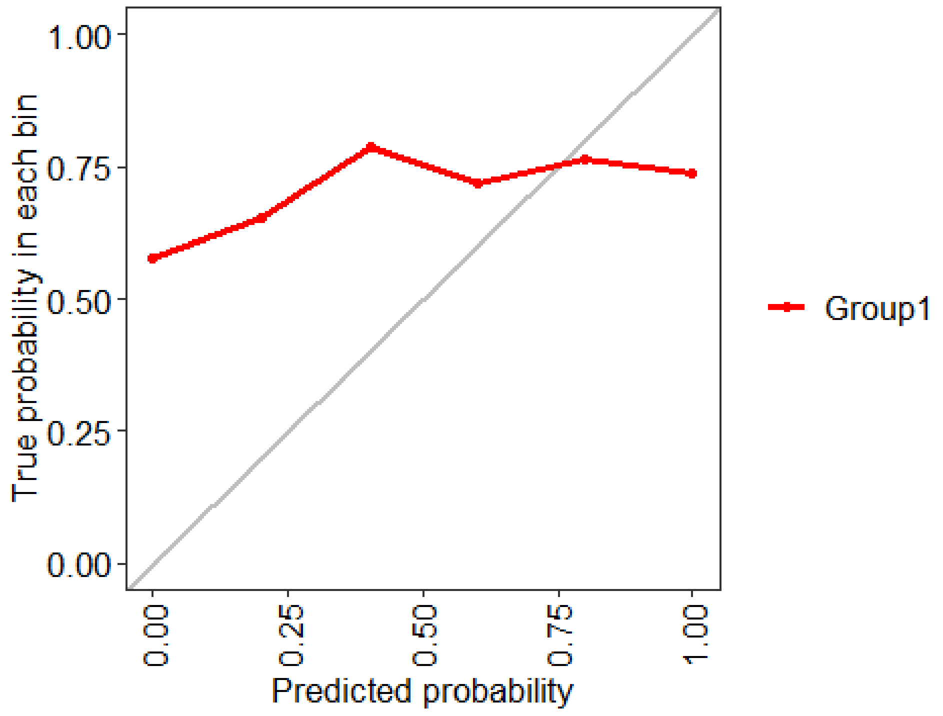

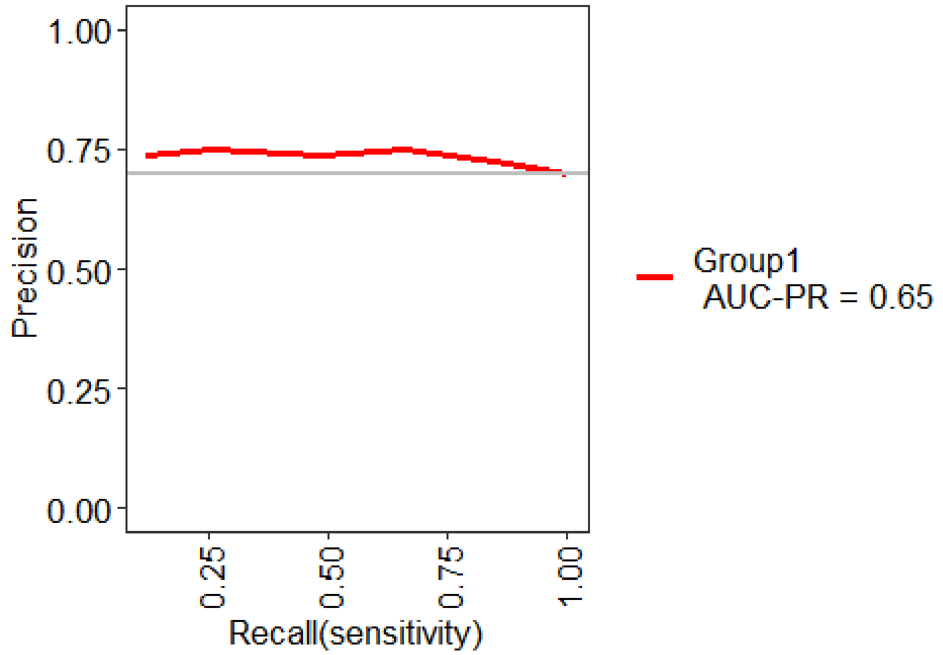

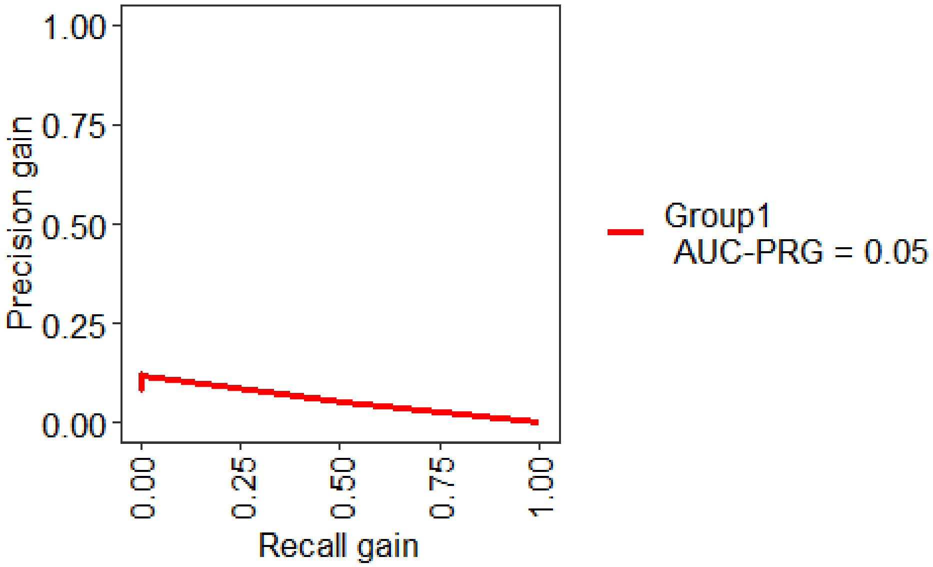

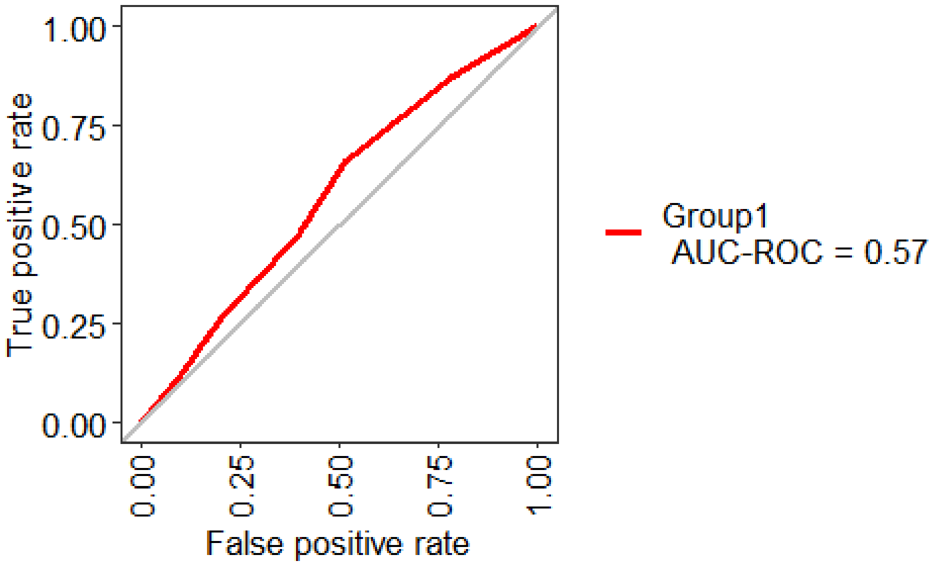


```
yknnadas$stdres
```

```
## $Group1
##                   Score        CI
## SENS              0.486 0.42-0.55
## SPEC              0.596 0.49-0.69
## MCC               0.075      <NA>
## Informedness      0.081      <NA>
## PREC              0.737  0.66-0.8
## NPV               0.331 0.26-0.41
## FPR               0.404      <NA>
## F1                0.586      <NA>
## TP              101.000      <NA>
## FP               36.000      <NA>
## TN               53.000      <NA>
## FN              107.000      <NA>
## AUC-ROC           0.570  0.5-0.64
## AUC-PR            0.650      <NA>
## AUC-PRG           0.050      <NA>
```

```
set.seed(seed)
lr_fit_adas<-train(class~., data = traindata.adas, method = "regLogistic",
                   trControl=ctrl, metric=metric, tuneLength=5, verbose=FALS
E)
lr_fit_adas
```

```
## Regularized Logistic Regression
##
## 1092 samples
##   60 predictor
##    2 classes: 'unfavor', 'favor'
##
## No pre-processing
## Resampling: Cross-Validated (10 fold, repeated 10 times)
## Summary of sample sizes: 982, 984, 983, 983, 983, 982, ...
## Resampling results across tuning parameters:
##
##   cost  loss       epsilon  ROC        Sens       Spec
##   0.25  L1         1e-04    0.7854758  0.7098182  0.7272082
##   0.25  L1         1e-03    0.7855296  0.7101786  0.7272082
##   0.25  L1         1e-02    0.7854295  0.7091006  0.7270231
##   0.25  L1         1e-01    0.7848793  0.7040390  0.7242243
##   0.25  L1         1e+00    0.7575081  0.6716299  0.7051188
##   0.25  L2_dual    1e-04    0.7851466  0.7335747  0.7247973
##   0.25  L2_dual    1e-03    0.7851500  0.7335747  0.7247973
##   0.25  L2_dual    1e-02    0.7851402  0.7335747  0.7247973
##   0.25  L2_dual    1e-01    0.7851266  0.7337532  0.7247939
##   0.25  L2_dual    1e+00    0.7849793  0.7326656  0.7238539
##   0.25  L2_primal  1e-04    0.7851400  0.7335747  0.7247973
##   0.25  L2_primal  1e-03    0.7851735  0.7333929  0.7247973
##   0.25  L2_primal  1e-02    0.7848383  0.7339351  0.7247939
```

```
##   0.25  L2_primal  1e-01    0.7812245  0.7304935  0.7232984
##   0.25  L2_primal  1e+00    0.7658040  0.7023961  0.7150978
##   0.50  L1         1e-04    0.7844653  0.7205649  0.7272257
##   0.50  L1         1e-03    0.7843842  0.7205649  0.7272257
##   0.50  L1         1e-02    0.7844096  0.7203961  0.7264885
##   0.50  L1         1e-01    0.7844620  0.7162760  0.7257023
##   0.50  L1         1e+00    0.7468333  0.6652338  0.6870999
##   0.50  L2_dual    1e-04    0.7856116  0.7322955  0.7236758
##   0.50  L2_dual    1e-03    0.7856116  0.7322955  0.7236758
##   0.50  L2_dual    1e-02    0.7856116  0.7322955  0.7236758
##   0.50  L2_dual    1e-01    0.7856358  0.7321136  0.7234871
##   0.50  L2_dual    1e+00    0.7851581  0.7319156  0.7240566
##   0.50  L2_primal  1e-04    0.7856183  0.7322955  0.7236758
##   0.50  L2_primal  1e-03    0.7856083  0.7324740  0.7236758
##   0.50  L2_primal  1e-02    0.7854701  0.7310357  0.7229315
##   0.50  L2_primal  1e-01    0.7838607  0.7292078  0.7232879
##   0.50  L2_primal  1e+00    0.7654325  0.7032792  0.7160412
##   1.00  L1         1e-04    0.7858442  0.7270552  0.7255521
##   1.00  L1         1e-03    0.7857668  0.7268766  0.7248078
##   1.00  L1         1e-02    0.7846400  0.7268831  0.7235115
##   1.00  L1         1e-01    0.7832544  0.7205779  0.7234941
##   1.00  L1         1e+00    0.7447113  0.6697825  0.6869497
##   1.00  L2_dual    1e-04    0.7856508  0.7274091  0.7223725
##   1.00  L2_dual    1e-03    0.7856541  0.7274091  0.7223725
##   1.00  L2_dual    1e-02    0.7856609  0.7272305  0.7223725
##   1.00  L2_dual    1e-01    0.7856443  0.7272305  0.7218169
##   1.00  L2_dual    1e+00    0.7854121  0.7272468  0.7223585
##   1.00  L2_primal  1e-04    0.7856409  0.7274091  0.7223725
##   1.00  L2_primal  1e-03    0.7856208  0.7275909  0.7227463
##   1.00  L2_primal  1e-02    0.7856042  0.7272338  0.7223760
##   1.00  L2_primal  1e-01    0.7850584  0.7263377  0.7212509
##   1.00  L2_primal  1e+00    0.7652542  0.7032760  0.7177184
##   2.00  L1         1e-04    0.7855253  0.7281299  0.7233019
##   2.00  L1         1e-03    0.7854351  0.7283117  0.7234906
##   2.00  L1         1e-02    0.7847448  0.7274286  0.7231377
##   2.00  L1         1e-01    0.7818248  0.7212955  0.7234801
##   2.00  L1         1e+00    0.7465577  0.6741169  0.6871523
##   2.00  L2_dual    1e-04    0.7856152  0.7286786  0.7223690
##   2.00  L2_dual    1e-03    0.7856152  0.7286786  0.7223690
##   2.00  L2_dual    1e-02    0.7856117  0.7286786  0.7223690
##   2.00  L2_dual    1e-01    0.7856584  0.7283149  0.7225542
##   2.00  L2_dual    1e+00    0.7853764  0.7286753  0.7201223
##   2.00  L2_primal  1e-04    0.7856051  0.7284968  0.7223690
##   2.00  L2_primal  1e-03    0.7856019  0.7284968  0.7223690
##   2.00  L2_primal  1e-02    0.7855939  0.7281364  0.7221803
##   2.00  L2_primal  1e-01    0.7850280  0.7281364  0.7212474
##   2.00  L2_primal  1e+00    0.7651364  0.7041688  0.7173480
##   4.00  L1         1e-04    0.7853962  0.7276006  0.7242278
##   4.00  L1         1e-03    0.7853122  0.7272435  0.7240461
```

```
##   4.00  L1          1e-02    0.7843728  0.7274253  0.7233089
##   4.00  L1          1e-01    0.7818287  0.7241786  0.7253389
##   4.00  L1          1e+00    0.7431065  0.6800812  0.6836233
##   4.00  L2_dual     1e-04    0.7855904  0.7293929  0.7229350
##   4.00  L2_dual     1e-03    0.7855938  0.7293929  0.7229350
##   4.00  L2_dual     1e-02    0.7855738  0.7297532  0.7229350
##   4.00  L2_dual     1e-01    0.7855868  0.7295747  0.7227498
##   4.00  L2_dual     1e+00    0.7853885  0.7288506  0.7195528
##   4.00  L2_primal   1e-04    0.7855871  0.7293929  0.7229350
##   4.00  L2_primal   1e-03    0.7855702  0.7295714  0.7227498
##   4.00  L2_primal   1e-02    0.7855932  0.7293896  0.7229315
##   4.00  L2_primal   1e-01    0.7849154  0.7279416  0.7212474
##   4.00  L2_primal   1e+00    0.7650957  0.7038084  0.7179071
##
## ROC was used to select the optimal model using the largest value.
## The final values used for the model were cost = 1, loss = L1 and epsilon
##  = 1e-04.
```

```
xlradas <- evalm(lr_fit_adas)
```

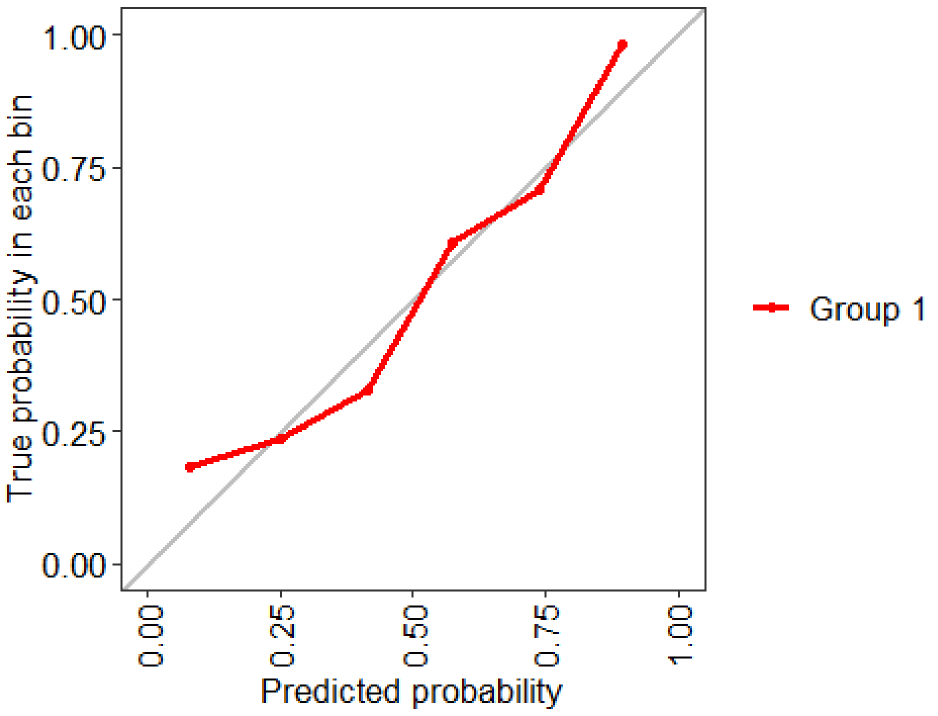
1.00
0.75
0.50
0.25
0.00
True probability in each bin
0.00
0.25
0.50
0.75
1.00
Predicted probability
Group 1

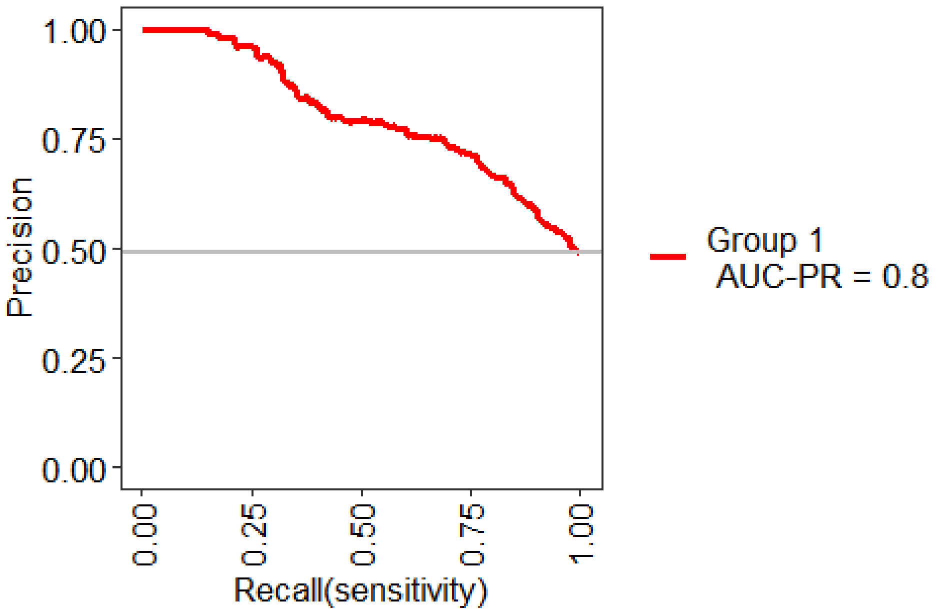
1.00
0.75
0.50
0.25
0.00
Precision
0.00
0.25
0.50
0.75
1.00
Recall(sensitivity)
Group 1
AUC-PR = 0.8

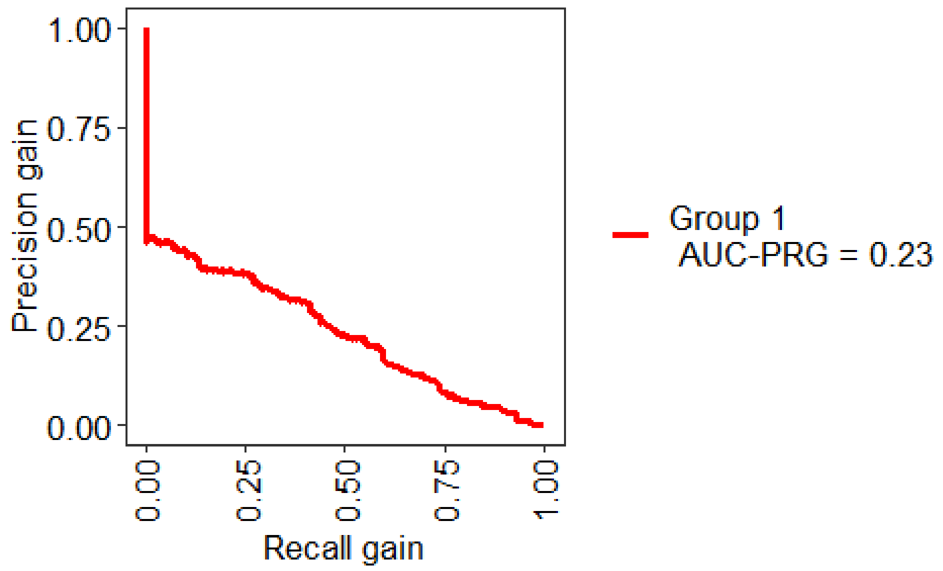

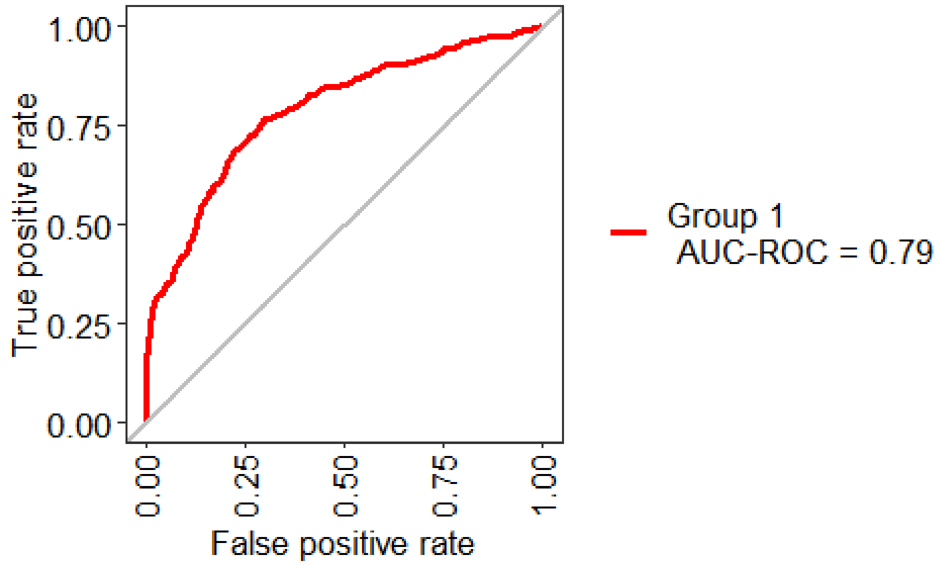


```
xlradas$stdres
```

```
## $`Group 1`
##                   Score        CI
## SENS              0.730 0.69-0.77
## SPEC              0.726 0.69-0.76
## MCC               0.456      <NA>
## Informedness      0.456      <NA>
## PREC              0.721 0.68-0.76
## NPV               0.735  0.7-0.77
## FPR               0.274      <NA>
## F1                0.725      <NA>
## TP              392.000      <NA>
## FP              152.000      <NA>
## TN              403.000      <NA>
## FN              145.000      <NA>
## AUC-ROC           0.790 0.76-0.82
## AUC-PR            0.800      <NA>
## AUC-PRG           0.230      <NA>
```

```
test_pred_lradas <- predict(lr_fit_adas, newdata = testdata)
confusionMatrix(test_pred_lradas, testdata$Outcome)
```

```
## Confusion Matrix and Statistics
##
##           Reference
## Prediction favor unfavor
##    favor     159      22
##    unfavor    49      67
##
##                Accuracy : 0.7609
##                  95% CI : (0.7083, 0.8083)
##     No Information Rate : 0.7003
##     P-Value [Acc > NIR] : 0.012085
##
##                   Kappa : 0.4759
##
##  Mcnemar's Test P-Value : 0.002031
##
##             Sensitivity : 0.7644
##             Specificity : 0.7528
##          Pos Pred Value : 0.8785
##          Neg Pred Value : 0.5776
##              Prevalence : 0.7003
##          Detection Rate : 0.5354
##    Detection Prevalence : 0.6094
##       Balanced Accuracy : 0.7586
##
##        'Positive' Class : favor
##
```

```
test_prob_lradas <- predict(lr_fit_adas, newdata = testdata, type="prob")
mlradas = data.frame(test_prob_lradas, testdata$Outcome)
ylradas<-evalm(mlradas)
```

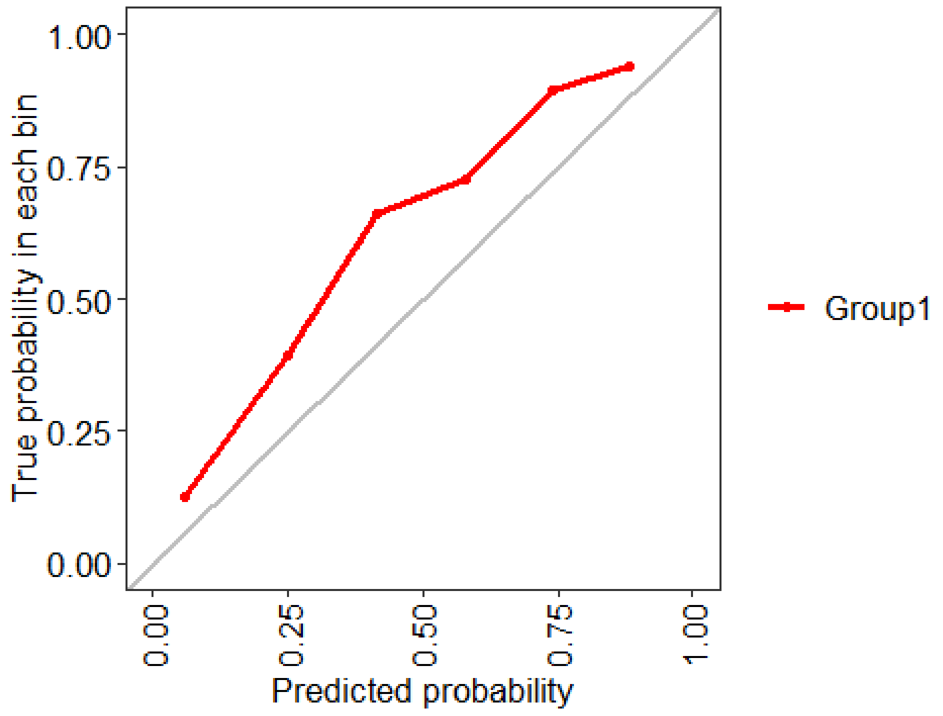

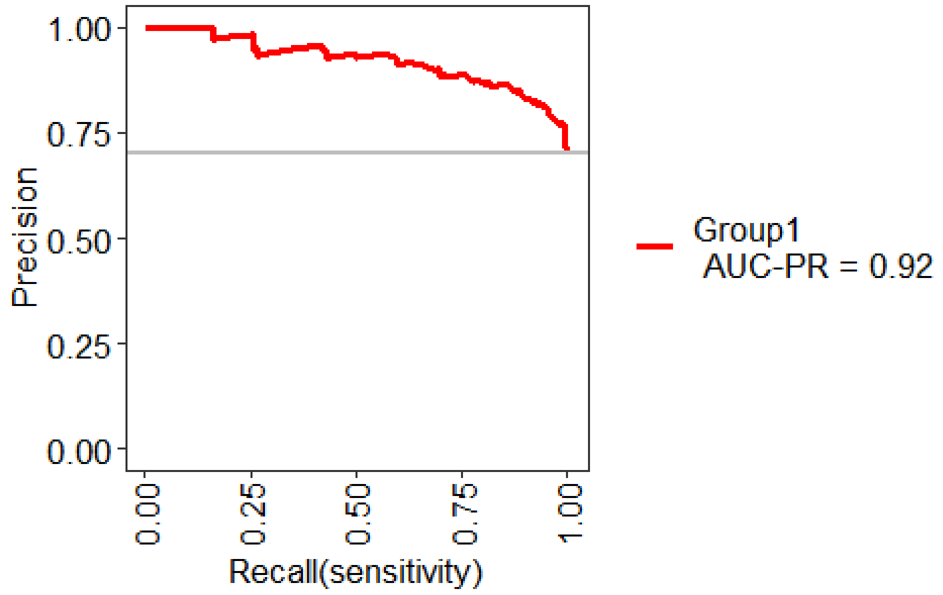

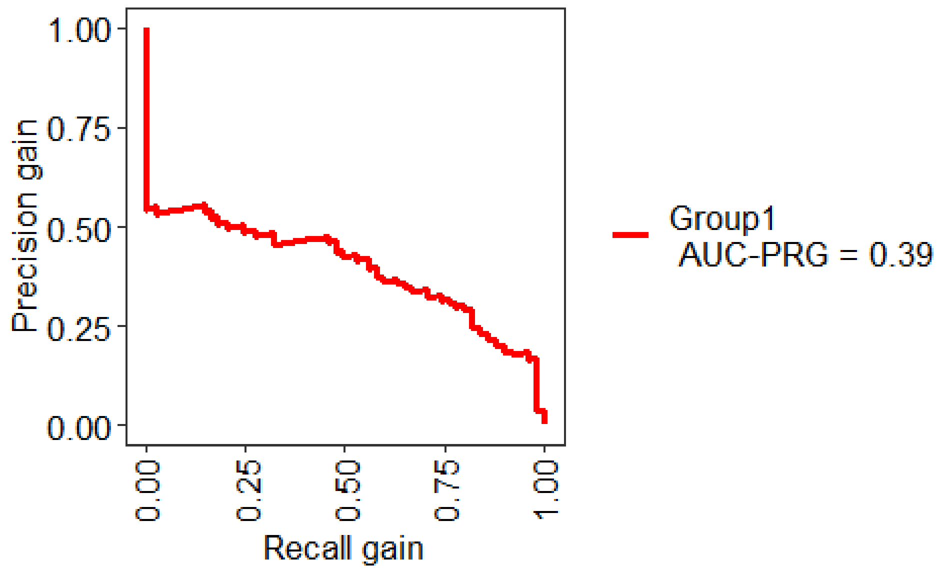

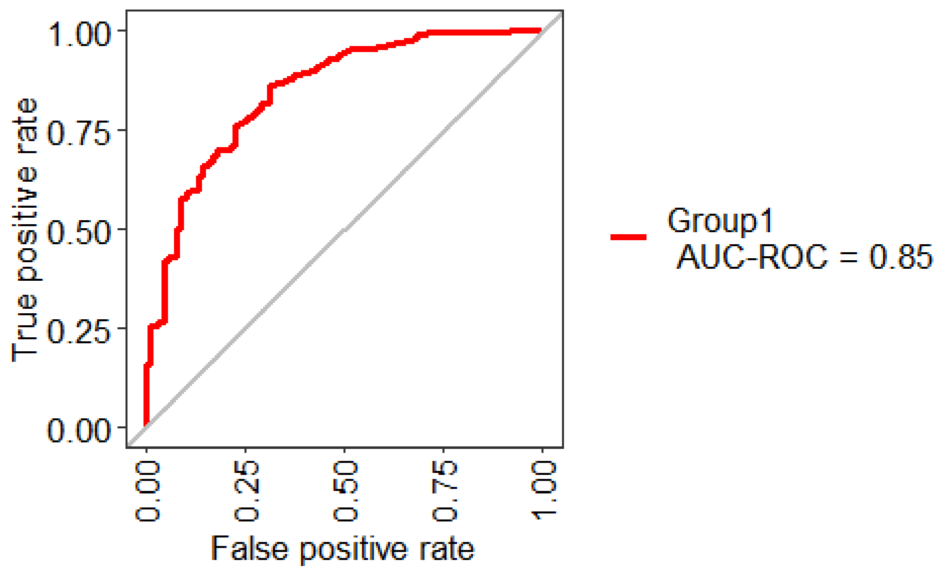


```
ylradas$stdres
```

```
## $Group1
##                  Score        CI
## SENS             0.764  0.7-0.82
## SPEC             0.753 0.65-0.83
## MCC              0.486      <NA>
## Informedness     0.517      <NA>
## PREC             0.878 0.82-0.92
## NPV              0.578 0.49-0.66
## FPR              0.247      <NA>
## F1               0.817      <NA>
## TP             159.000      <NA>
## FP              22.000      <NA>
## TN              67.000      <NA>
## FN              49.000      <NA>
## AUC-ROC          0.850 0.81-0.89
## AUC-PR           0.920      <NA>
## AUC-PRG          0.390      <NA>
```

```
traindata$Outcome<-ifelse(traindata$Outcome=="unfavor", 'X1','X2')
testdata$Outcome<-ifelse(testdata$Outcome=="unfavor", 'X1','X2')
traindata$Outcome<-as.factor(traindata$Outcome)
testdata$Outcome<-as.factor(testdata$Outcome)
table(traindata$Outcome)
```

```
##
##  X1  X2
## 232 537
```

```
table(testdata$Outcome)
```

```
##
##  X1  X2
##  89 208
```

```
set.seed(seed)
rf_fit1 <- train(Outcome~., data = traindata, method = "rf",
                 trControl=ctrl, metric=metric, tuneLength=10,
                 ntree=500, verbose=FALSE)
rf_fit1
```

```
## Random Forest
##
## 769 samples
##  60 predictor
##   2 classes: 'X1', 'X2'
##
## No pre-processing
## Resampling: Cross-Validated (10 fold, repeated 10 times)
## Summary of sample sizes: 691, 693, 693, 693, 692, 692, ...
## Resampling results across tuning parameters:
```

```
##
##   mtry  ROC        Sens        Spec
##    2    0.7559333  0.04213768  0.9953494
##    8    0.7894252  0.35637681  0.9318484
##   14    0.7965962  0.41882246  0.9098672
##   21    0.7976357  0.45202899  0.9007512
##   27    0.7985394  0.46634058  0.8955136
##   34    0.7978595  0.47971014  0.8865828
##   40    0.7975575  0.48320652  0.8834277
##   47    0.7962521  0.48324275  0.8826695
##   53    0.7941233  0.48530797  0.8791509
##   60    0.7944241  0.49092391  0.8780119
##
## ROC was used to select the optimal model using the largest value.
## The final value used for the model was mtry = 27.
```

```
x1 <- evalm(rf_fit1)
```

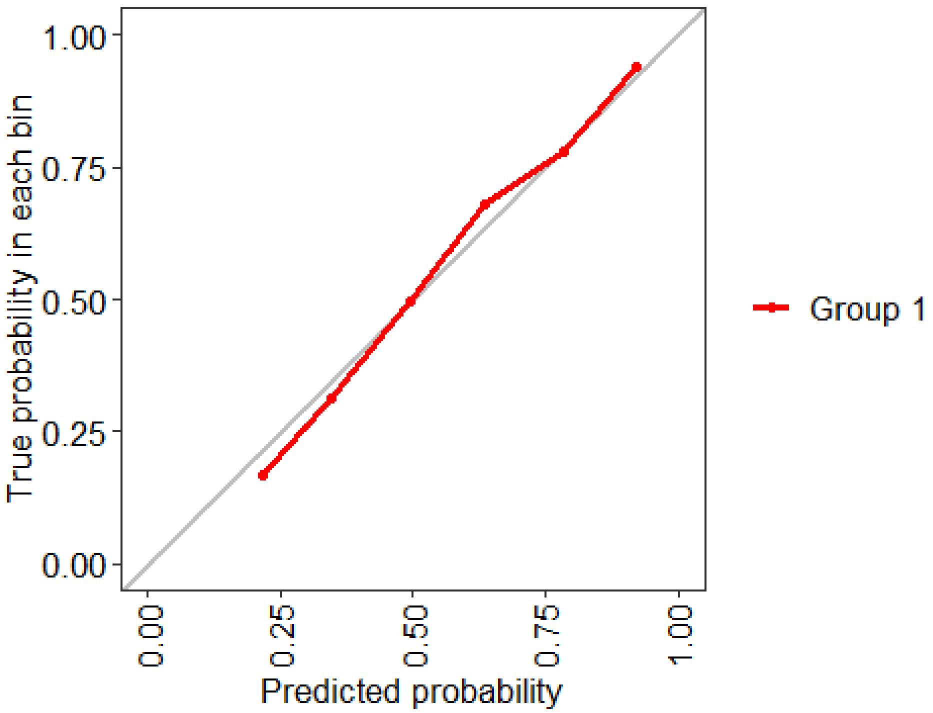
1.00
0.75
0.50
0.25
0.00
True probability in each bin
0.00
0.25
0.50
0.75
1.00
Predicted probability
Group 1

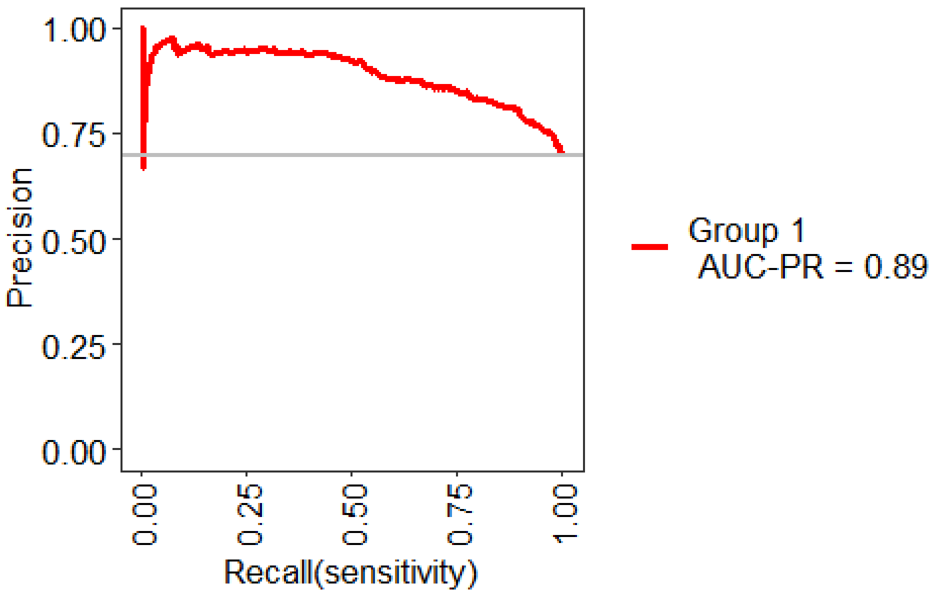
1.00
0.75
0.50
0.25
0.00
Precision
0.00
0.25
0.50
0.75
1.00
Recall(sensitivity)
Group 1
AUC-PR = 0.89

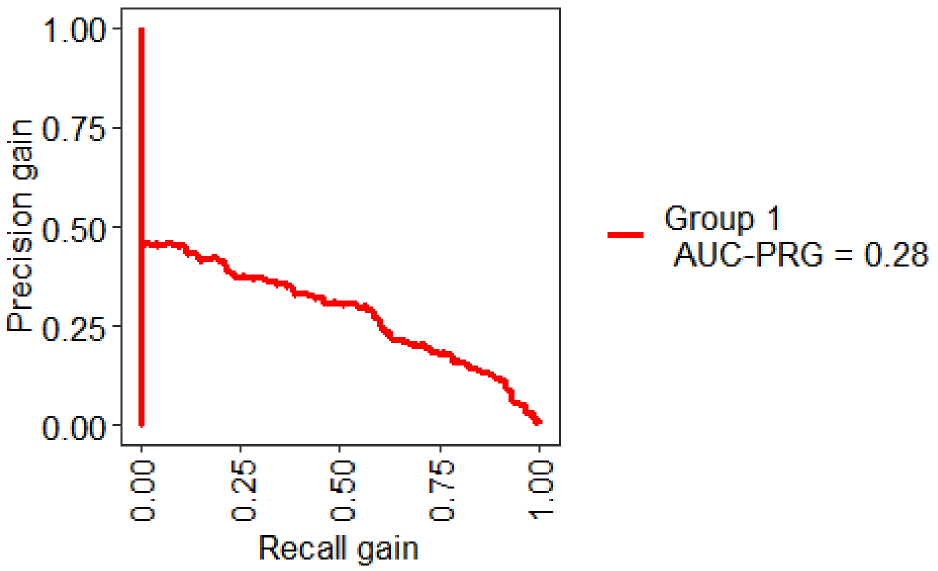
1.00
0.75
0.50
0.25
0.00
Precision gain
0.00
0.25
0.50
0.75
1.00
Recall gain
Group 1
AUC-PRG = 0.28

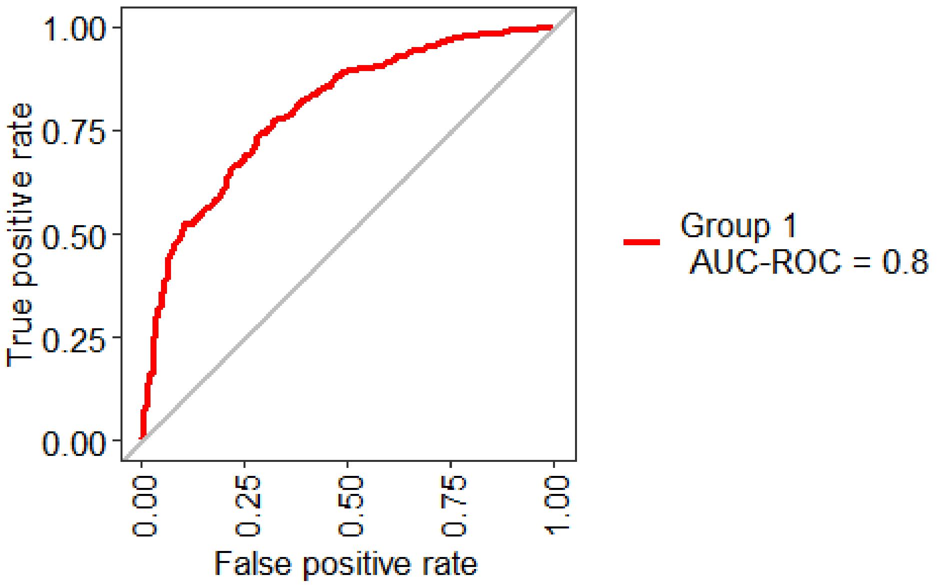
1.00
0.75
0.50
0.25
0.00
True positive rate
0.00
0.25
0.50
0.75
1.00
False positive rate
Group 1
AUC-ROC = 0.8

```
x1$stdres
```

```
## $`Group 1`
##                  Score        CI
## SENS             0.899 0.87-0.92
## SPEC             0.457 0.39-0.52
## MCC              0.403      <NA>
## Informedness     0.356      <NA>
## PREC             0.793 0.76-0.82
## NPV              0.662 0.59-0.73
## FPR              0.543      <NA>
## F1               0.843      <NA>
## TP             483.000      <NA>
## FP             126.000      <NA>
## TN             106.000      <NA>
## FN              54.000      <NA>
## AUC-ROC          0.800 0.77-0.83
## AUC-PR           0.890      <NA>
## AUC-PRG          0.280      <NA>
```

```
test_pred_rf1 <- predict(rf_fit1, newdata = testdata)
confusionMatrix(test_pred_rf1, testdata$Outcome)
```

```
## Confusion Matrix and Statistics
##
##           Reference
## Prediction  X1  X2
##         X1  46  22
##         X2  43 186
##
##                Accuracy : 0.7811
##                  95% CI : (0.7297, 0.8268)
##     No Information Rate : 0.7003
##     P-Value [Acc > NIR] : 0.001122
##
##                   Kappa : 0.4408
##
##  Mcnemar's Test P-Value : 0.013113
##
##             Sensitivity : 0.5169
##             Specificity : 0.8942
##          Pos Pred Value : 0.6765
##          Neg Pred Value : 0.8122
##              Prevalence : 0.2997
##          Detection Rate : 0.1549
##    Detection Prevalence : 0.2290
##       Balanced Accuracy : 0.7055
##
##        'Positive' Class : X1
##
```

```
test_prob_rf1 <- predict(rf_fit1, newdata = testdata, type="prob")
m1 = data.frame(test_prob_rf1, testdata$Outcome)
y1<-evalm(m1)
```

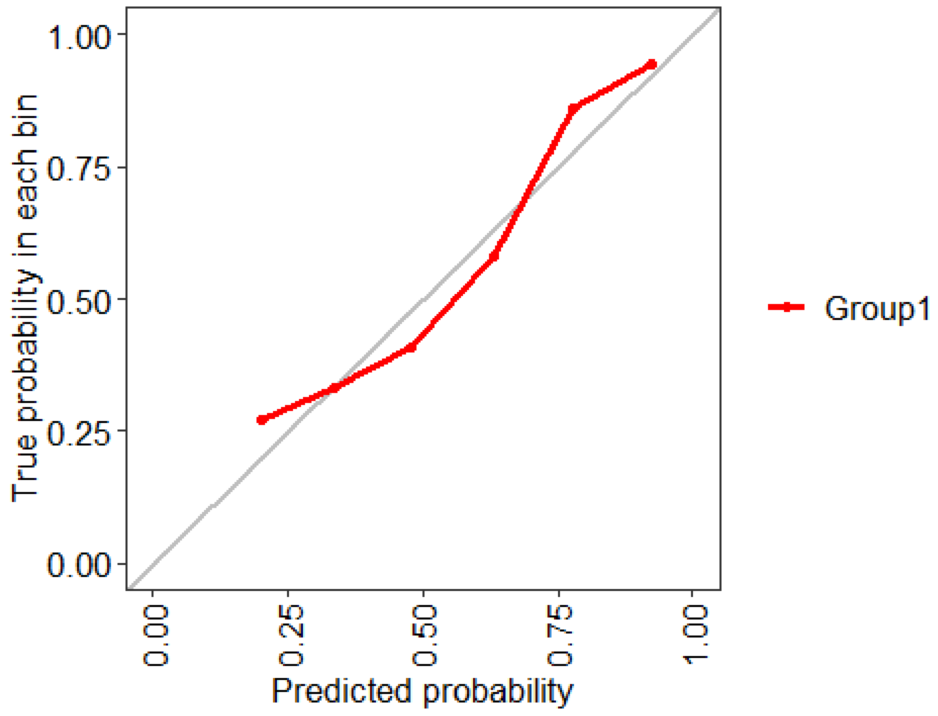

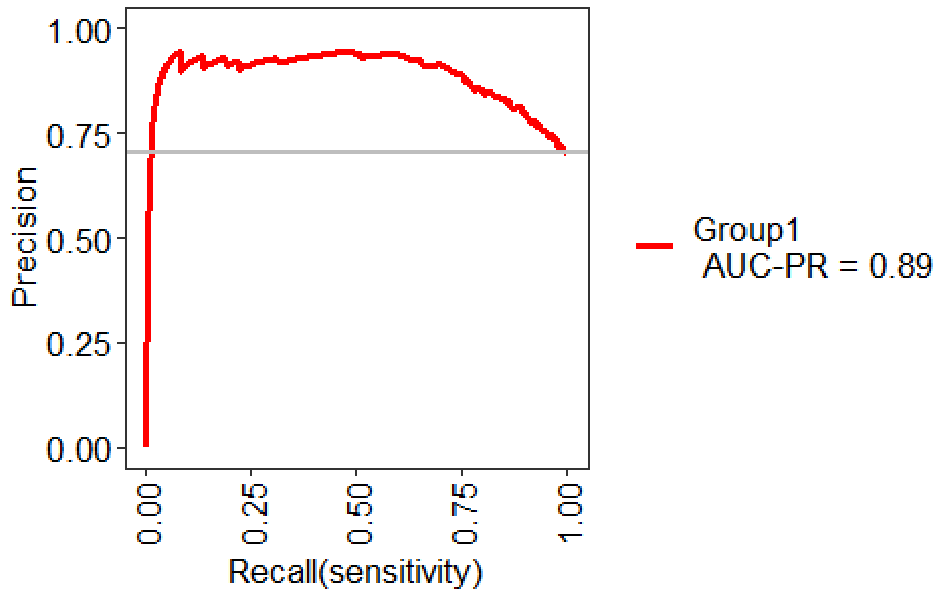

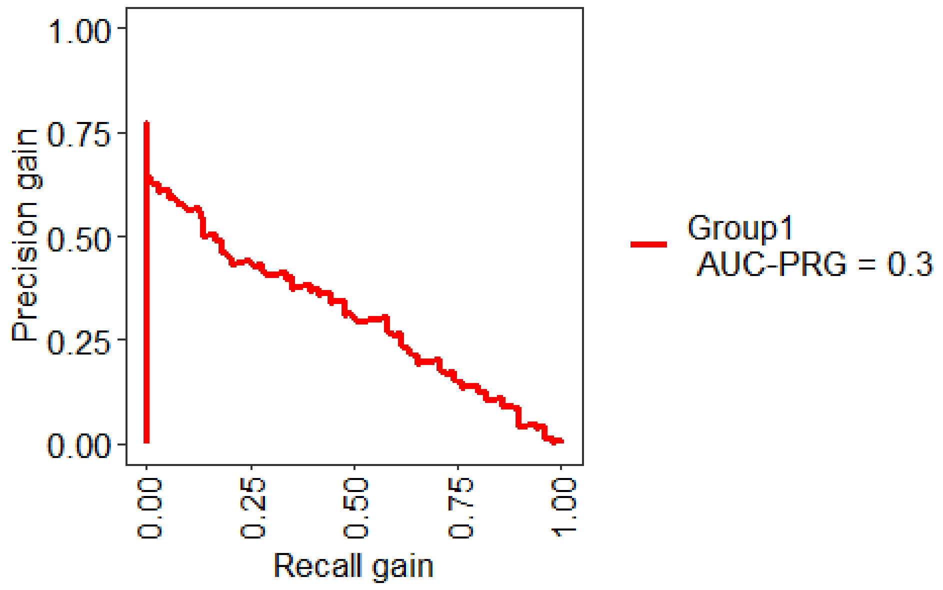
1.00
0.75
0.50
0.25
0.00
Precision gain
0.00
0.25
0.50
0.75
1.00
Recall gain
Group1
AUC-PRG = 0.3

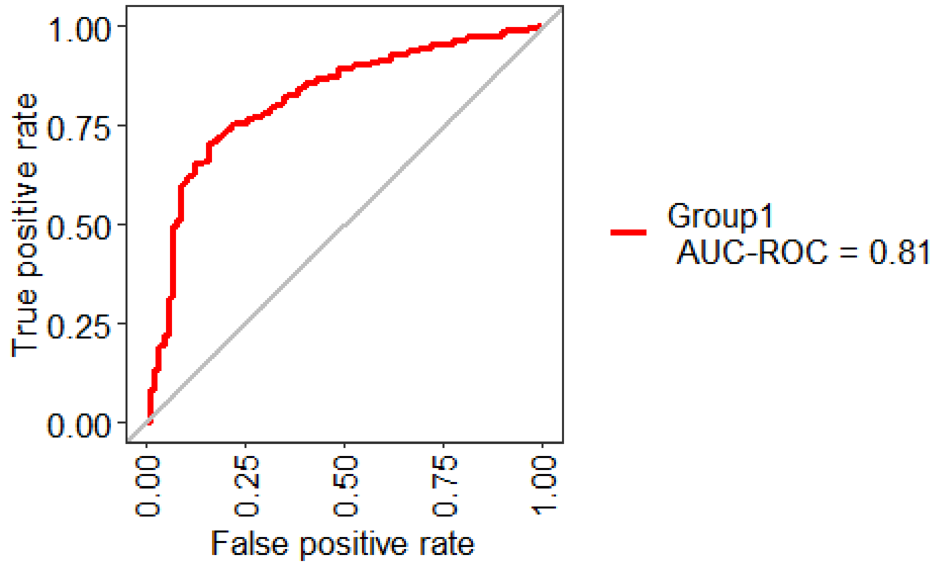
1.00
0.75
0.50
0.25
0.00
True positive rate
0.00
0.25
0.50
0.75
1.00
False positive rate
Group1
AUC-ROC = 0.81

```
y1$stdres
```

```
## $Group1
##                  Score        CI
## SENS             0.894 0.85-0.93
## SPEC             0.494  0.39-0.6
## MCC              0.428      <NA>
## Informedness     0.389      <NA>
## PREC             0.805 0.75-0.85
## NPV              0.667 0.55-0.77
## FPR              0.506      <NA>
## F1               0.847      <NA>
## TP             186.000      <NA>
## FP              45.000      <NA>
## TN              44.000      <NA>
## FN              22.000      <NA>
## AUC-ROC          0.810 0.76-0.86
## AUC-PR           0.890      <NA>
## AUC-PRG          0.300      <NA>
```

```
imp1<-varImp(rf_fit1, scale = FALSE)
imp1
```

```
## rf variable importance
## 
##   only 20 most important variables shown (out of 60)
## 
##                Overall
## NIHSS_adm       56.406
## Age             43.365
## Arrival         16.305
## RanGlu          13.954
## TG              13.773
## Hb              13.589
## PLT             13.480
## LDL             13.078
## BMI             13.046
## BUN             12.446
## HbA1C           12.167
## HDL             11.589
## TC              11.110
## SBP             11.000
## DBP             10.957
## AC               9.621
## Cr               6.940
## PRE_STROKE.2     1.818
## Subtype.CE       1.693
## Subtype.SVO      1.544
```

```
plot(imp1)
```

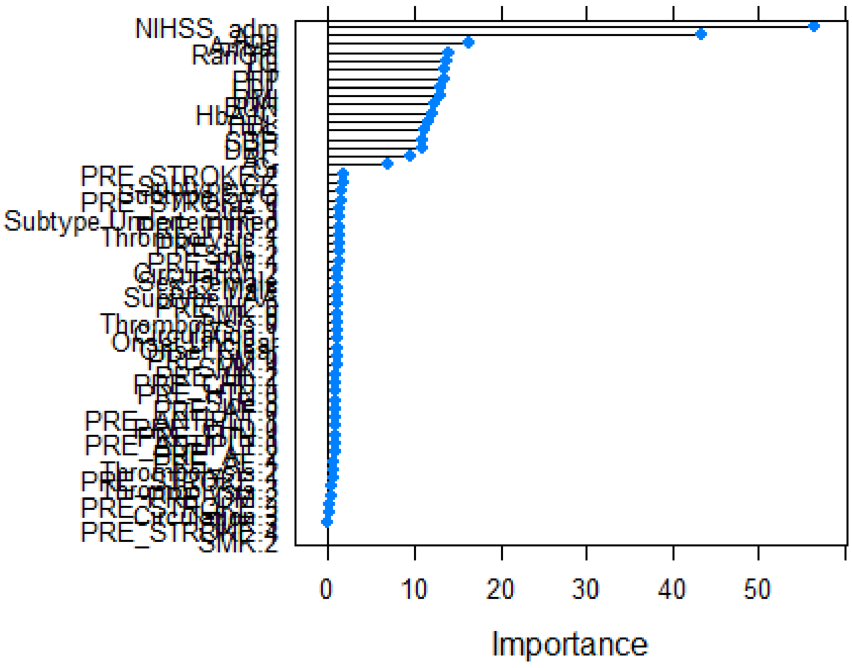


```
set.seed(seed)
rf_fit2 <- train(Outcome~., data = traindata, method = "rf",
                 trControl=ctrl, metric=metric, tuneLength=10,
                 ntree=1000, verbose=FALSE)
rf_fit2

## Random Forest
##
## 769 samples
##  60 predictor
##   2 classes: 'X1', 'X2'
##
## No pre-processing
## Resampling: Cross-Validated (10 fold, repeated 10 times)
## Summary of sample sizes: 691, 693, 693, 693, 692, 692, ...
## Resampling results across tuning parameters:
##
##   mtry  ROC        Sens       Spec
##    2    0.7590144  0.0409058  0.9960867
##    8    0.7909054  0.3559783  0.9314710
##   14    0.7969542  0.4158877  0.9100489
##   21    0.7985477  0.4511413  0.9024179
##   27    0.7989268  0.4697464  0.8955276
##   34    0.7987021  0.4814855  0.8878896
##   40    0.7978965  0.4887500  0.8836094
##   47    0.7965168  0.4819565  0.8813697
```

```
##   53    0.7953973  0.4836051  0.8796890
##   60    0.7949668  0.4891667  0.8795073
##
## ROC was used to select the optimal model using the largest value.
## The final value used for the model was mtry = 27.
```

```
x2 <- evalm(rf_fit2)
```

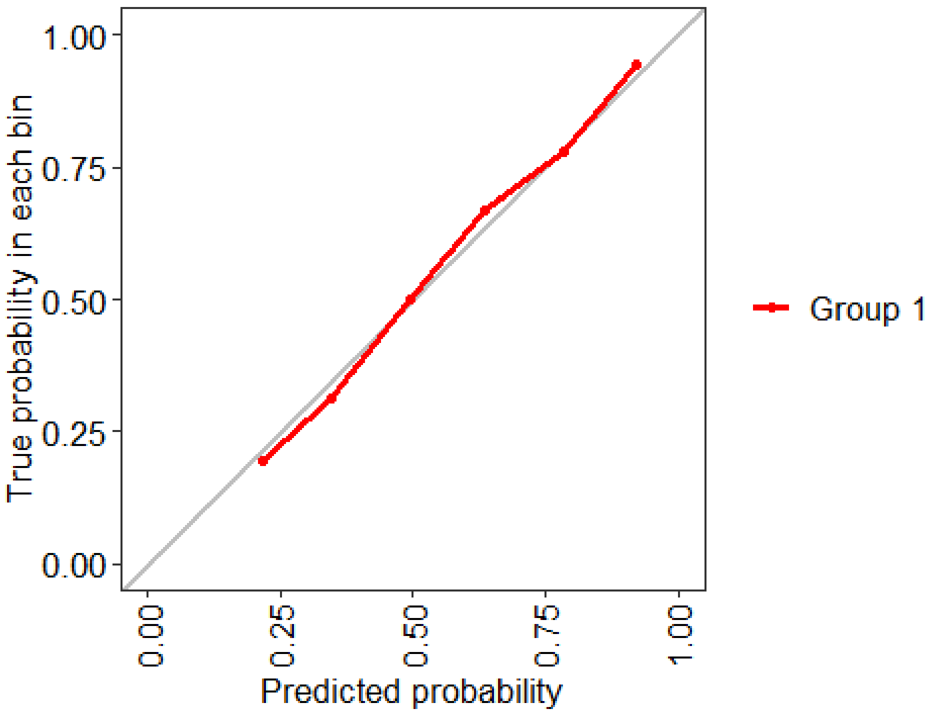

1.00
0.75
0.50
0.25
0.00
True probability in each bin
0.00
0.25
0.50
0.75
1.00
Predicted probability
Group 1

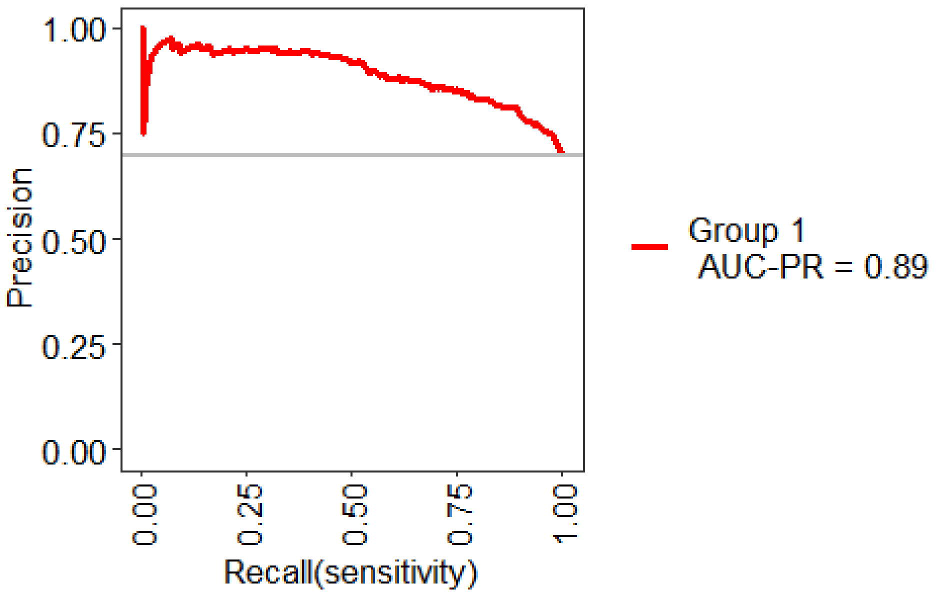

1.00
0.75
0.50
0.25
0.00
Precision
0.00
0.25
0.50
0.75
1.00
Recall(sensitivity)
Group 1
AUC-PR = 0.89

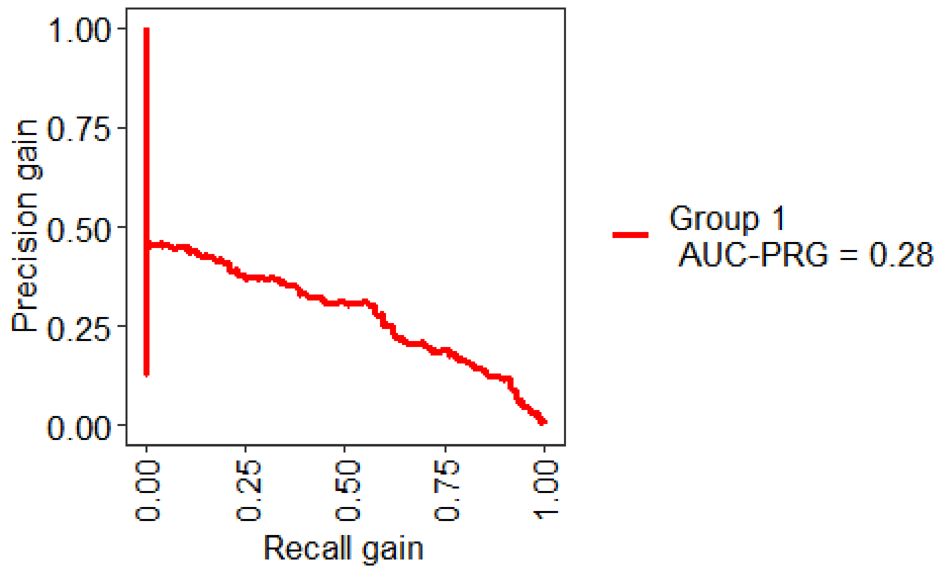
1.00
0.75
0.50
0.25
0.00
Precision gain
0.00
0.25
0.50
0.75
1.00
Recall gain
Group 1
AUC-PRG = 0.28

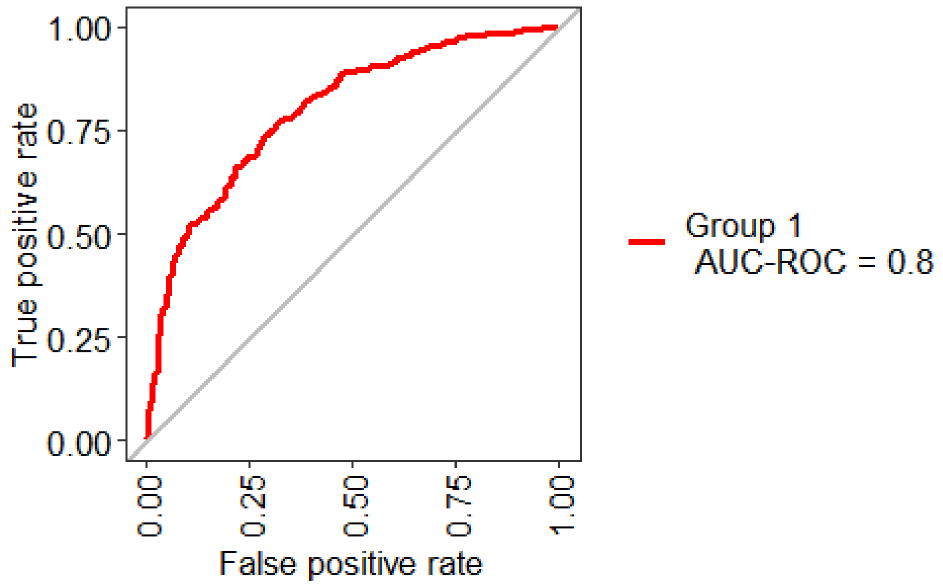
1.00
0.75
0.50
0.25
0.00
True positive rate
0.00
0.25
0.50
0.75
1.00
False positive rate
Group 1
AUC-ROC = 0.8

```
x2$stdres
```

```
## $`Group 1`
##                  Score        CI
## SENS             0.898 0.87-0.92
## SPEC             0.466  0.4-0.53
## MCC              0.408      <NA>
## Informedness     0.363      <NA>
## PREC             0.795 0.76-0.83
## NPV              0.663 0.59-0.73
## FPR              0.534      <NA>
## F1               0.843      <NA>
## TP             482.000      <NA>
## FP             124.000      <NA>
## TN             108.000      <NA>
## FN              55.000      <NA>
## AUC-ROC          0.800 0.77-0.83
## AUC-PR           0.890      <NA>
## AUC-PRG          0.280      <NA>
```

```
test_pred_rf2 <- predict(rf_fit2, newdata = testdata)
confusionMatrix(test_pred_rf2, testdata$Outcome)
```

```
## Confusion Matrix and Statistics
##
##             Reference
## Prediction   X1  X2
##         X1   45  23
##         X2   44 185
##
##                Accuracy : 0.7744
##                  95% CI : (0.7226, 0.8207)
##     No Information Rate : 0.7003
##     P-Value [Acc > NIR] : 0.00266
##
##                   Kappa : 0.4236
##
##  Mcnemar's Test P-Value : 0.01455
##
##             Sensitivity : 0.5056
##             Specificity : 0.8894
##          Pos Pred Value : 0.6618
##          Neg Pred Value : 0.8079
##              Prevalence : 0.2997
##          Detection Rate : 0.1515
##    Detection Prevalence : 0.2290
##       Balanced Accuracy : 0.6975
##
##        'Positive' Class : X1
##
```

```
test_prob_rf2 <- predict(rf_fit2, newdata = testdata, type="prob")
m2 = data.frame(test_prob_rf2, testdata$Outcome)
y2<-evalm(m2)
```

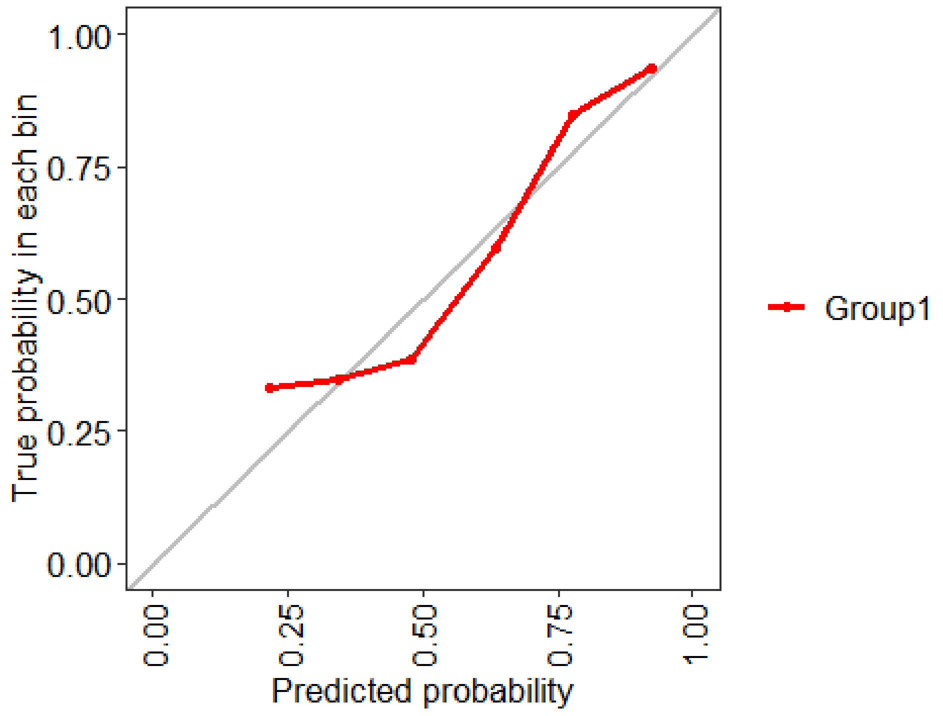

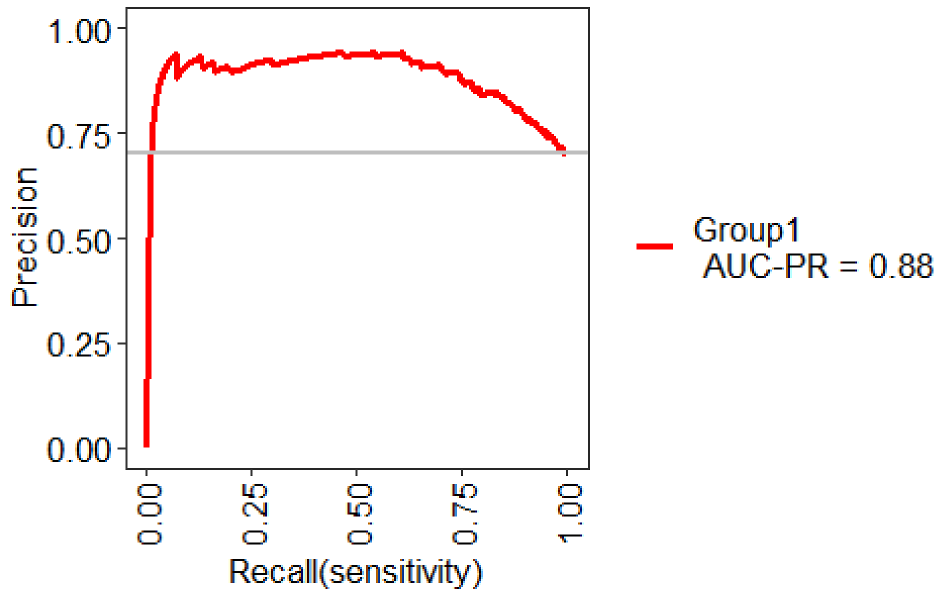

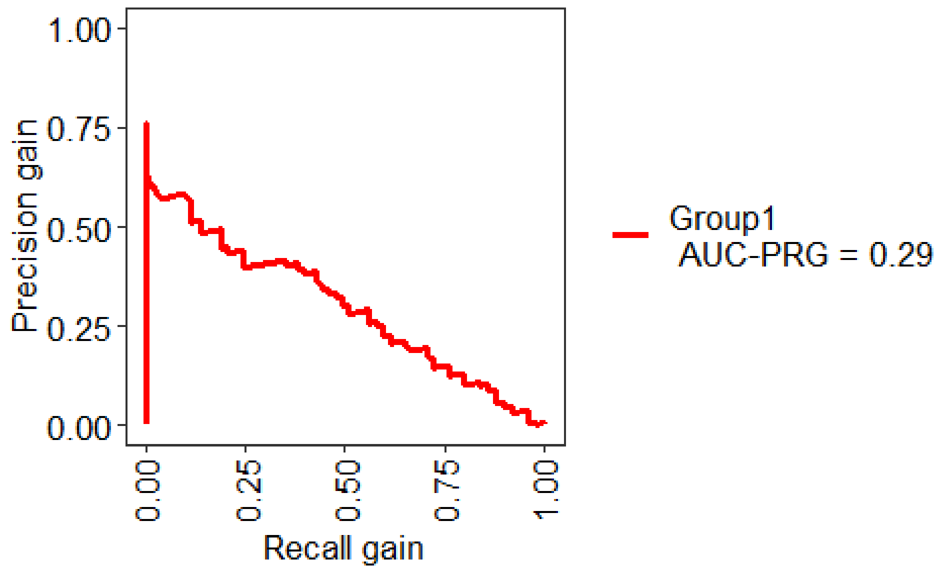
1.00
0.75
0.50
0.25
0.00
Precision gain
0.00
0.25
0.50
0.75
1.00
Recall gain
Group1
AUC-PRG = 0.29

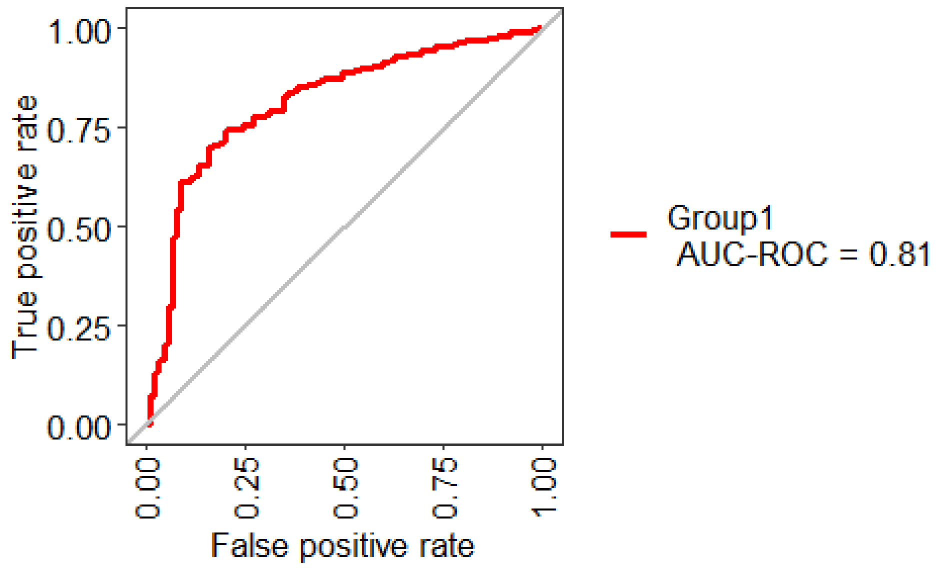
1.00
0.75
0.50
0.25
0.00
True positive rate
0.00
0.25
0.50
0.75
1.00
False positive rate
Group1
AUC-ROC = 0.81

```
y2$stdres
```

```
## $Group1
##                   Score        CI
## SENS              0.889 0.84-0.93
## SPEC              0.506  0.4-0.61
## MCC               0.431      <NA>
## Informedness      0.395      <NA>
## PREC              0.808 0.75-0.85
## NPV               0.662 0.54-0.76
## FPR               0.494      <NA>
## F1                0.847      <NA>
## TP              185.000      <NA>
## FP               44.000      <NA>
## TN               45.000      <NA>
## FN               23.000      <NA>
## AUC-ROC           0.810 0.76-0.86
## AUC-PR            0.880      <NA>
## AUC-PRG           0.290      <NA>
```

```
imp2<-varImp(rf_fit2, scale = FALSE)
imp2
```

```
## rf variable importance
##
##   only 20 most important variables shown (out of 60)
##
##                 Overall
## NIHSS_adm        56.900
## Age              43.162
## Arrival          16.153
## RanGlu           14.061
## PLT              13.754
## TG               13.596
## BMI              13.241
## LDL              13.138
## Hb               13.071
## HbA1C            12.462
## BUN              12.224
## HDL              12.134
## DBP              10.981
## TC               10.873
## SBP              10.814
## AC                9.869
## Cr                6.778
## PRE_STROKE.2      1.896
## Subtype.CE        1.579
## Subtype.SVO       1.554
```

```
plot(imp2)
```

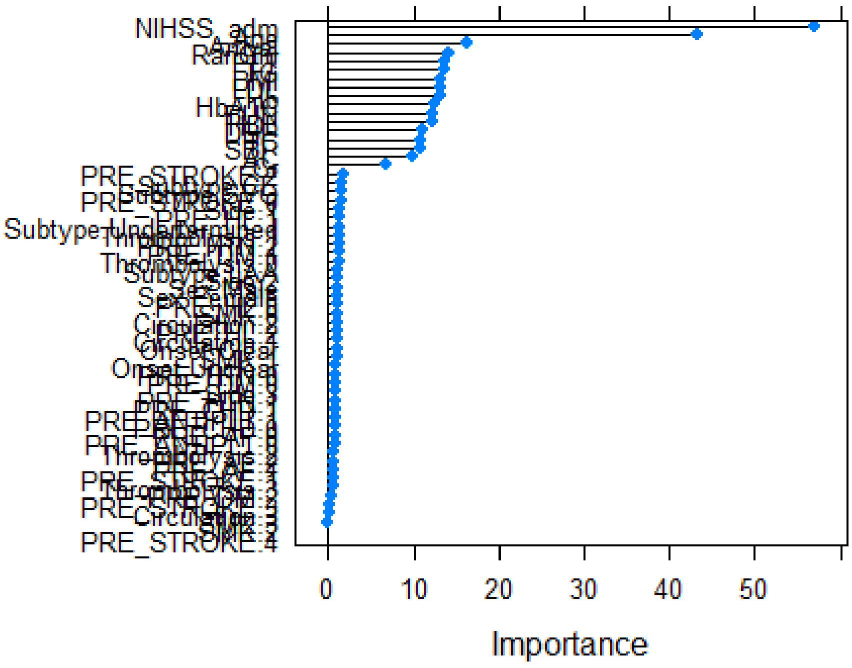


```
set.seed(seed)
rf_fit3 <- train(Outcome~., data = traindata, method = "rf",
                 trControl=ctrl, metric=metric, tuneLength=10,
                 ntree=1500, verbose=FALSE)
rf_fit3
```

```
## Random Forest
##
## 769 samples
##  60 predictor
##   2 classes: 'X1', 'X2'
##
## No pre-processing
## Resampling: Cross-Validated (10 fold, repeated 10 times)
## Summary of sample sizes: 691, 693, 693, 693, 692, 692, ...
## Resampling results across tuning parameters:
##
##   mtry  ROC        Sens        Spec
##    2    0.7598498  0.04047101  0.9962753
##    8    0.7912578  0.35336957  0.9312788
##   14    0.7971415  0.41675725  0.9102446
##   21    0.7987851  0.45119565  0.9005556
##   27    0.7990912  0.46846014  0.8946017
##   34    0.7986602  0.48019928  0.8882635
##   40    0.7980862  0.48659420  0.8847170
##   47    0.7963529  0.48322464  0.8815549
```

```
##   53    0.7958040  0.48489130  0.8796890
##   60    0.7950050  0.48918478  0.8800594
##
## ROC was used to select the optimal model using the largest value.
## The final value used for the model was mtry = 27.
```

```r
x3 <- evalm(rf_fit3)
```

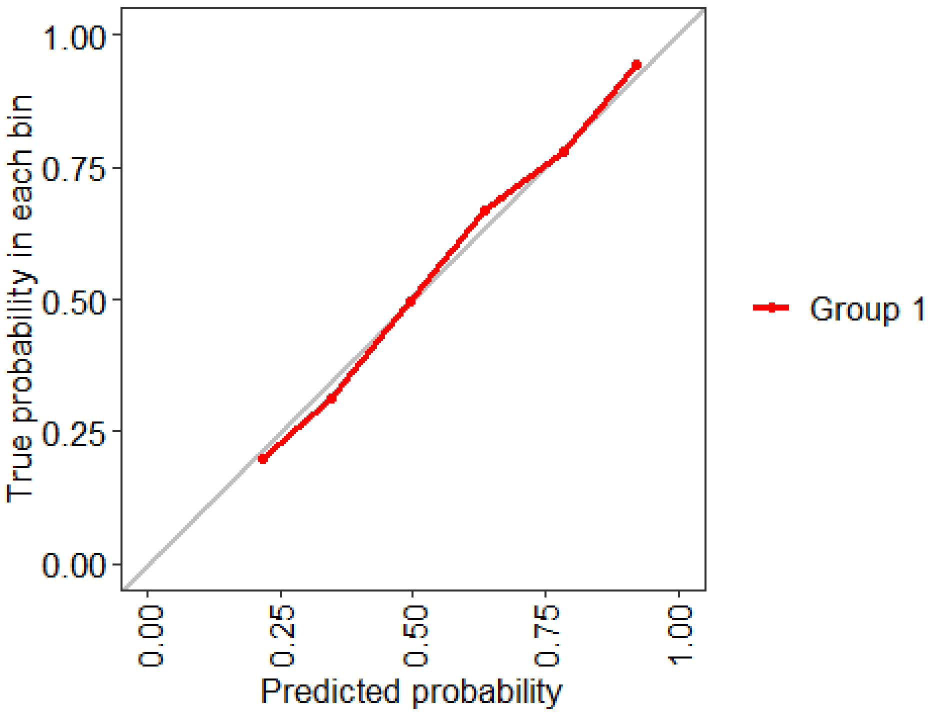
1.00
0.75
0.50
0.25
0.00
True probability in each bin
0.00
0.25
0.50
0.75
1.00
Predicted probability
Group 1

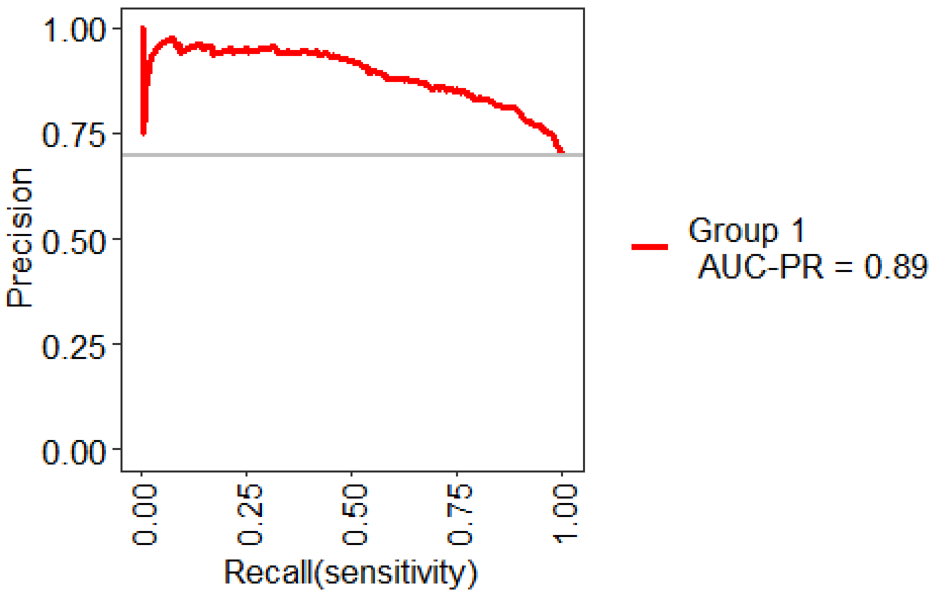
1.00
0.75
0.50
0.25
0.00
Precision
0.00
0.25
0.50
0.75
1.00
Recall(sensitivity)
Group 1
AUC-PR = 0.89

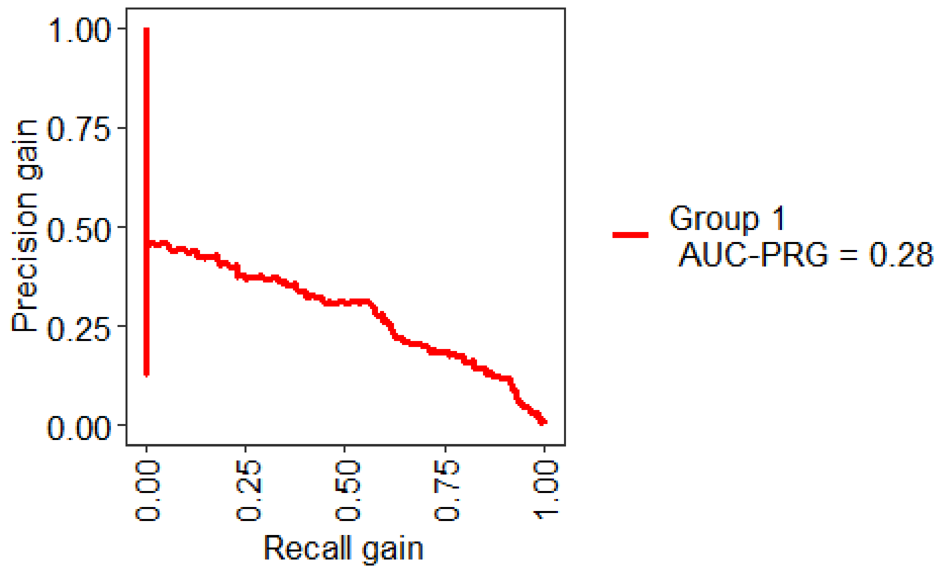
1.00
0.75
0.50
0.25
0.00
Precision gain
0.00
0.25
0.50
0.75
1.00
Recall gain
Group 1
AUC-PRG = 0.28

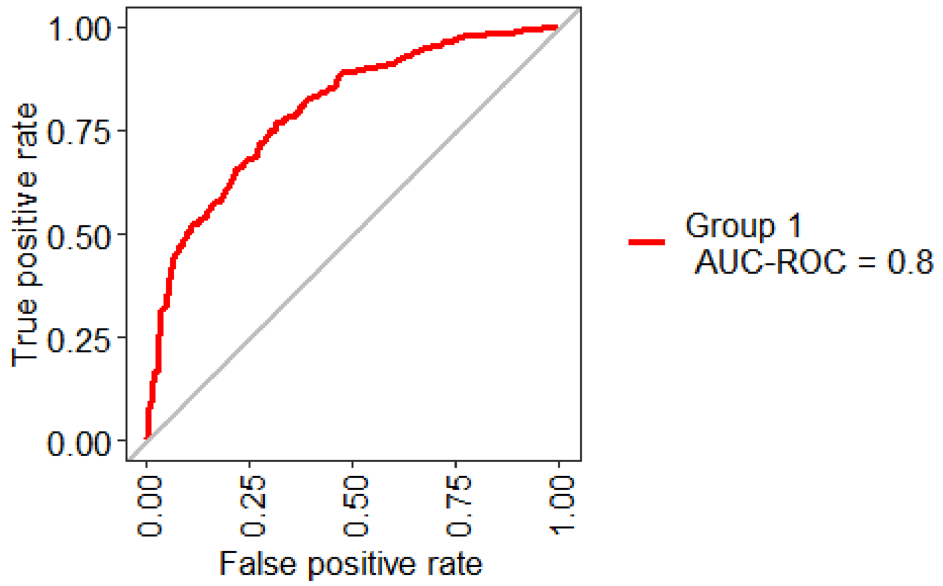
1.00
0.75
0.50
0.25
0.00
True positive rate
0.00
0.25
0.50
0.75
1.00
False positive rate
Group 1
AUC-ROC = 0.8

```
x3$stdres
```

```
## $`Group 1`
##                  Score        CI
## SENS             0.901 0.87-0.92
## SPEC             0.466  0.4-0.53
## MCC              0.414      <NA>
## Informedness     0.367      <NA>
## PREC             0.796 0.76-0.83
## NPV              0.671 0.59-0.74
## FPR              0.534      <NA>
## F1               0.845      <NA>
## TP             484.000      <NA>
## FP             124.000      <NA>
## TN             108.000      <NA>
## FN              53.000      <NA>
## AUC-ROC          0.800 0.77-0.83
## AUC-PR           0.890      <NA>
## AUC-PRG          0.280      <NA>
```

```
test_pred_rf3 <- predict(rf_fit3, newdata = testdata)
confusionMatrix(test_pred_rf3, testdata$Outcome)
```

```
## Confusion Matrix and Statistics
## 
##           Reference
## Prediction  X1  X2
##         X1  43  22
##         X2  46 186
## 
##                Accuracy : 0.771
##                  95% CI : (0.719, 0.8176)
##     No Information Rate : 0.7003
##     P-Value [Acc > NIR] : 0.003986
## 
##                   Kappa : 0.4089
## 
##  Mcnemar's Test P-Value : 0.005285
## 
##             Sensitivity : 0.4831
##             Specificity : 0.8942
##          Pos Pred Value : 0.6615
##          Neg Pred Value : 0.8017
##              Prevalence : 0.2997
##          Detection Rate : 0.1448
##    Detection Prevalence : 0.2189
##       Balanced Accuracy : 0.6887
## 
##        'Positive' Class : X1
## 
```

```
test_prob_rf3 <- predict(rf_fit3, newdata = testdata, type="prob")
m3 = data.frame(test_prob_rf3, testdata$Outcome)
y3<-evalm(m3)
```

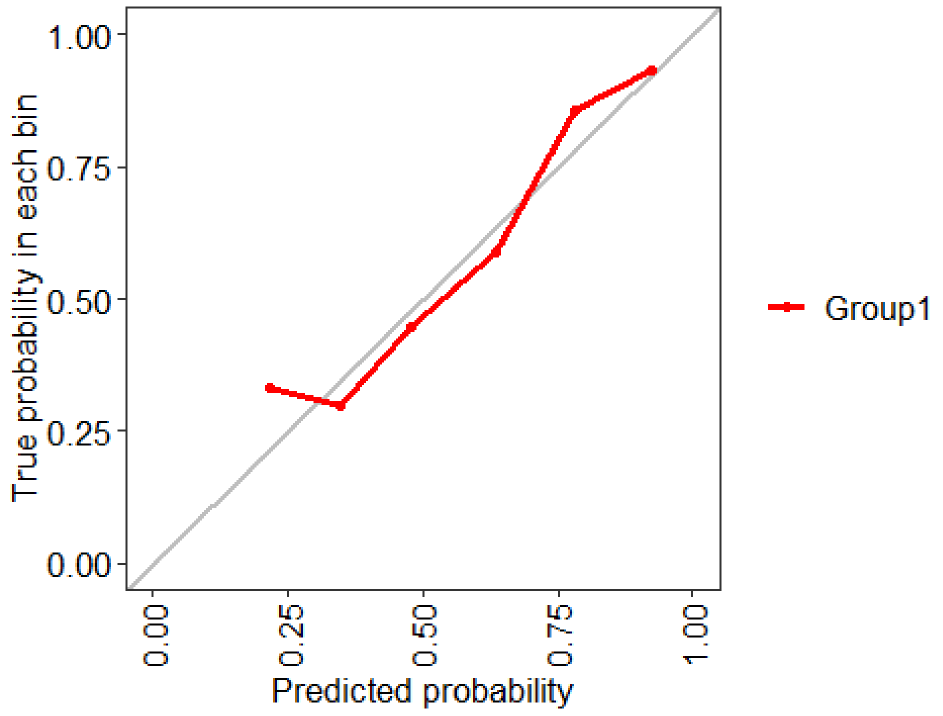

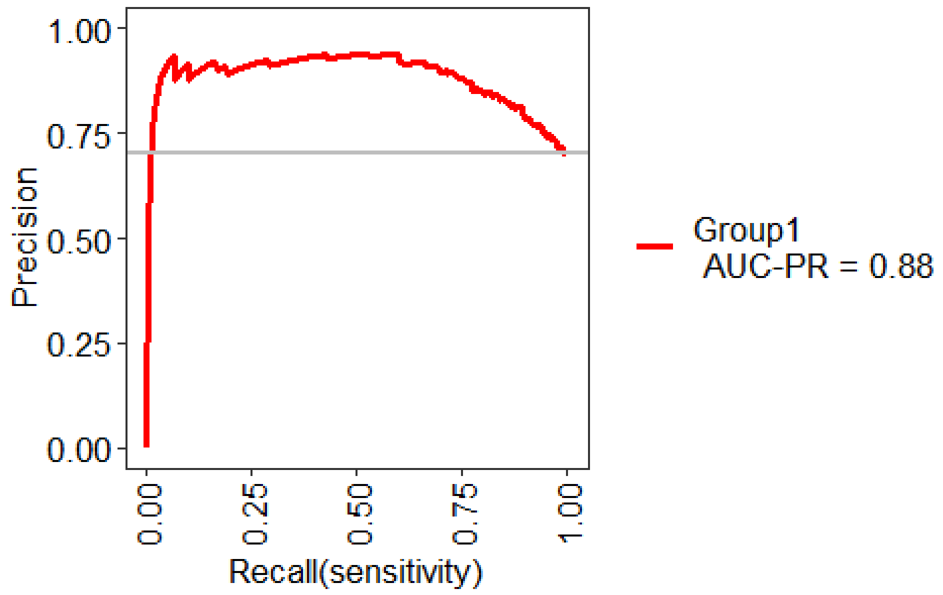

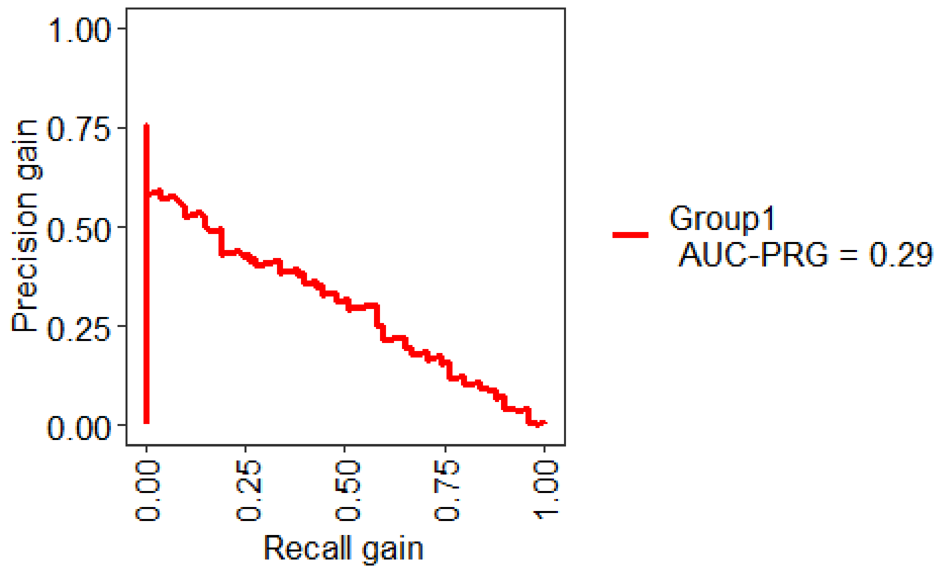
1.00
0.75
0.50
0.25
0.00
Precision gain
0.00
0.25
0.50
0.75
1.00
Recall gain
Group1
AUC-PRG = 0.29

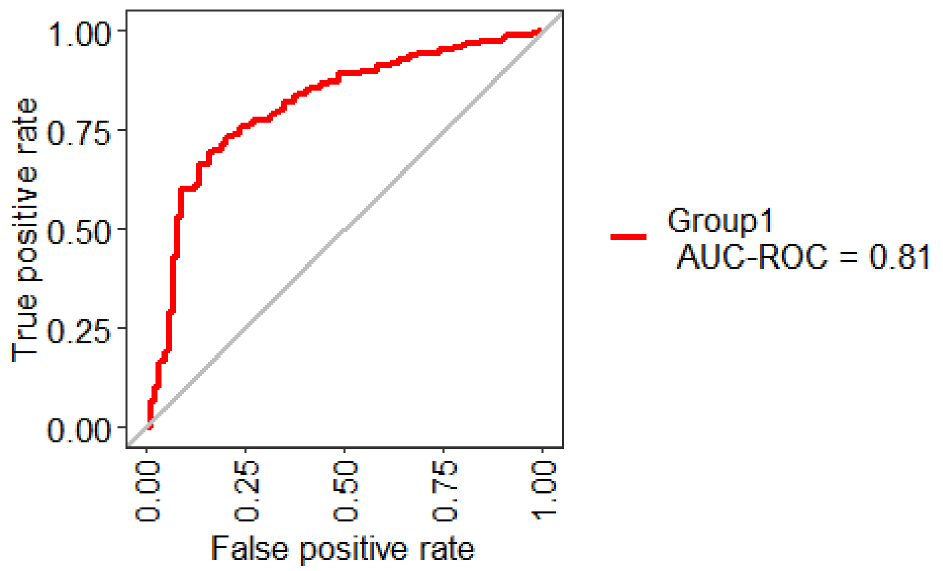
1.00
0.75
0.50
0.25
0.00
True positive rate
0.00
0.25
0.50
0.75
1.00
False positive rate
Group1
AUC-ROC = 0.81

```
y3$stdres
```

```
## $Group1
##                   Score        CI
## SENS              0.894 0.85-0.93
## SPEC              0.483 0.38-0.59
## MCC               0.418      <NA>
## Informedness      0.377      <NA>
## PREC              0.802 0.75-0.85
## NPV               0.662 0.54-0.76
## FPR               0.517      <NA>
## F1                0.845      <NA>
## TP              186.000      <NA>
## FP               46.000      <NA>
## TN               43.000      <NA>
## FN               22.000      <NA>
## AUC-ROC           0.810 0.76-0.86
## AUC-PR            0.880      <NA>
## AUC-PRG           0.290      <NA>
```

```
imp3<-varImp(rf_fit3, scale = FALSE)
imp3
```

```
## rf variable importance
##
##   only 20 most important variables shown (out of 60)
##
##                 Overall
## NIHSS_adm        56.364
## Age              43.275
## Arrival          16.134
## RanGlu           14.056
## PLT              13.818
## TG               13.573
## BMI              13.341
## Hb               13.258
## LDL              13.247
## HbA1C            12.638
## BUN              12.195
## HDL              11.877
## TC               11.074
## DBP              11.001
## SBP              10.805
## AC                9.854
## Cr                6.832
## PRE_STROKE.2      1.928
## PRE_STROKE.0      1.549
## Subtype.CE        1.540
```

```
plot(imp3)
```

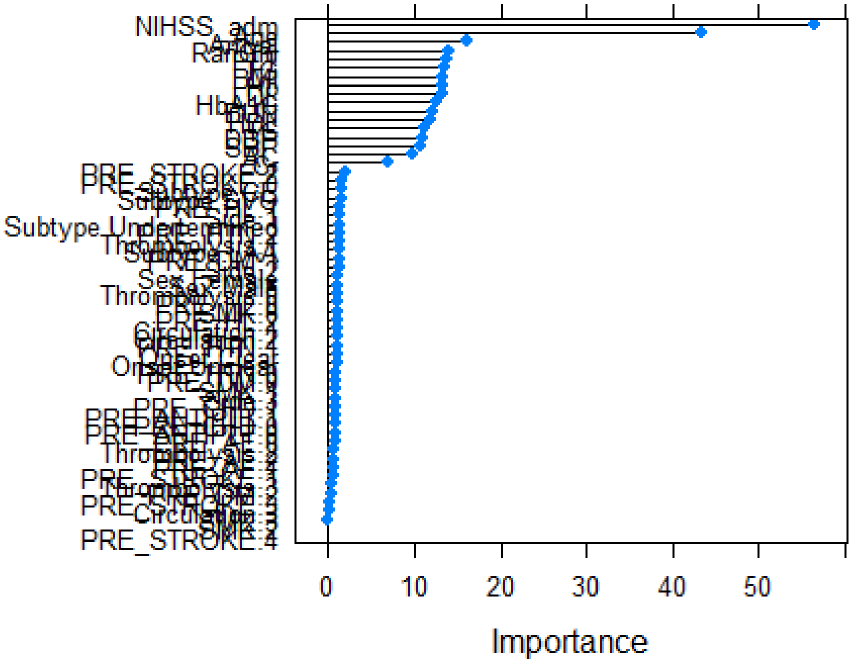


```
set.seed(seed)
xgb_fit <- train(Outcome~., data = traindata, method = "xgbTree",
                 trControl=ctrl, metric=metric, tuneGrid=xgbgrid)
xgb_fit

## eXtreme Gradient Boosting
##
## 769 samples
##  60 predictor
##   2 classes: 'X1', 'X2'
##
## No pre-processing
## Resampling: Cross-Validated (10 fold, repeated 10 times)
## Summary of sample sizes: 691, 693, 693, 693, 692, 692, ...
## Resampling results across tuning parameters:
##
##   eta   max_depth  nrounds  ROC        Sens       Spec
##   0.01  6           500     0.8072146  0.4336051  0.9031481
##   0.01  6          1000     0.8042736  0.4453080  0.8966492
##   0.01  6          1500     0.8025172  0.4475181  0.8908840
##   0.01  8           500     0.8044211  0.4172645  0.9070545
##   0.01  8          1000     0.8035490  0.4358877  0.8981237
##   0.01  8          1500     0.8021440  0.4453986  0.8936618
##   0.10  6           500     0.7880677  0.4604167  0.8742907
##   0.10  6          1000     0.7843400  0.4655435  0.8699930
##   0.10  6          1500     0.7819935  0.4664312  0.8688784
```

```
##   0.10  8           500     0.7907394  0.4634601  0.8770964
##   0.10  8          1000     0.7870869  0.4681703  0.8744724
##   0.10  8          1500     0.7844929  0.4733333  0.8733473
##
## Tuning parameter 'gamma' was held constant at a value of 0
## Tuning
##
## Tuning parameter 'min_child_weight' was held constant at a value of 1
##
## Tuning parameter 'subsample' was held constant at a value of 0.8
## ROC was used to select the optimal model using the largest value.
## The final values used for the model were nrounds = 500, max_depth = 6, eta
##  = 0.01, gamma = 0, colsample_bytree = 0.6, min_child_weight = 1 and
##  subsample = 0.8.

x_xgb <- evalm(xgb_fit)
```

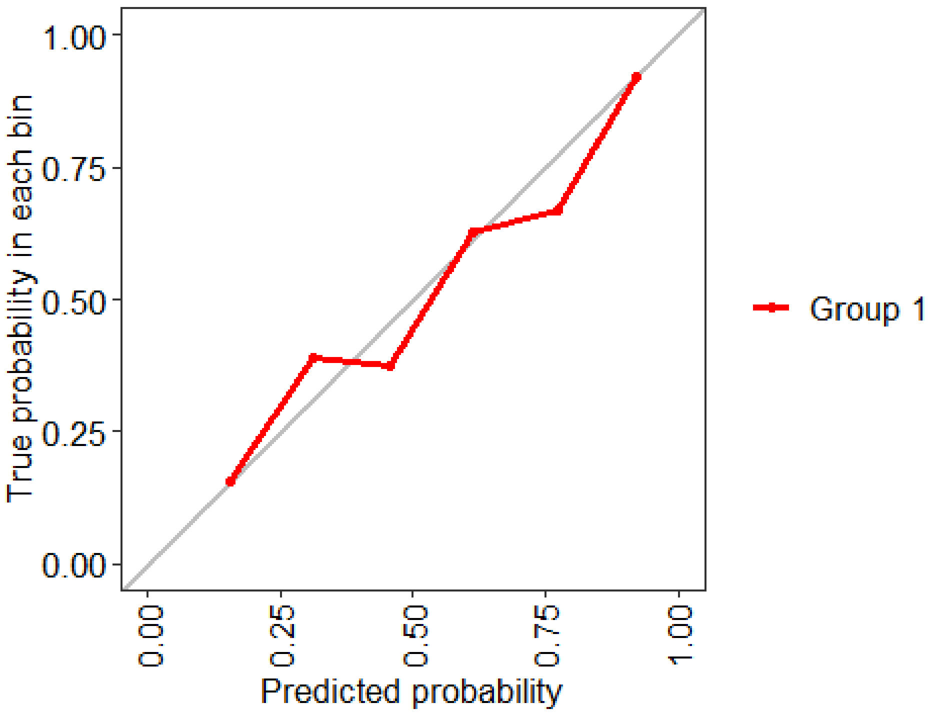

1.00
0.75
0.50
0.25
0.00
True probability in each bin
0.00
0.25
0.50
0.75
1.00
Predicted probability
Group 1

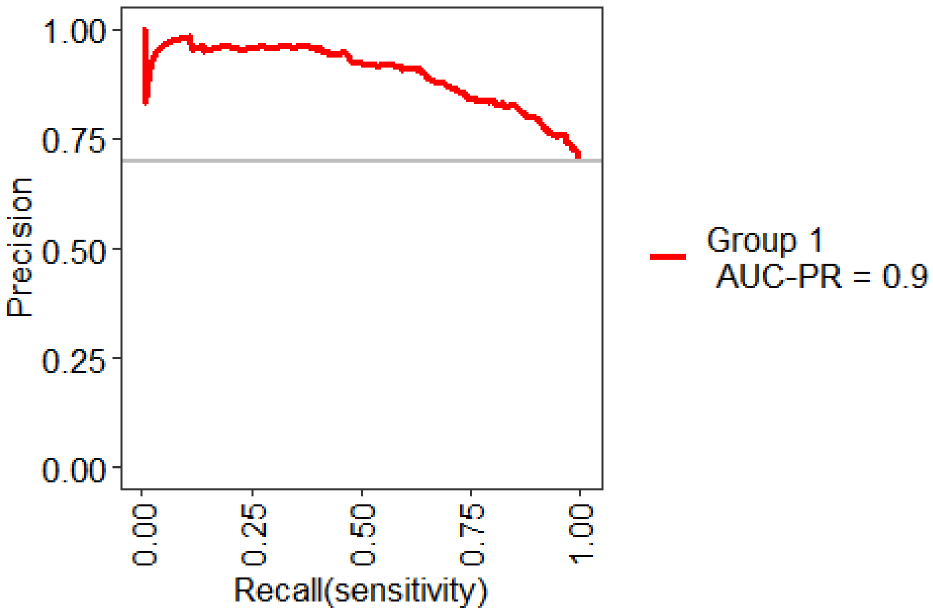

1.00
0.75
0.50
0.25
0.00
Precision
0.00
0.25
0.50
0.75
1.00
Recall(sensitivity)
Group 1
AUC-PR = 0.9

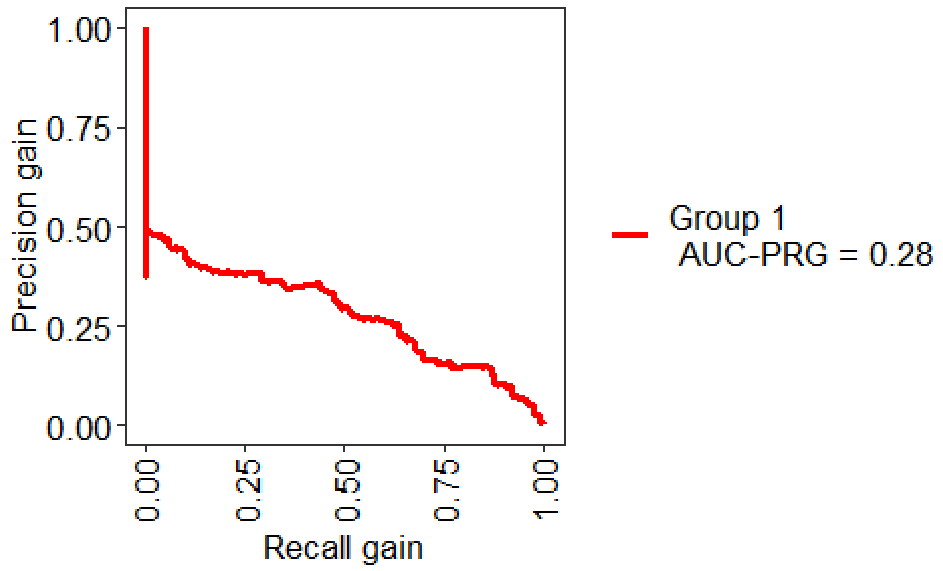

1.00
0.75
0.50
0.25
0.00
Precision gain
0.00
0.25
0.50
0.75
1.00
Recall gain
Group 1
AUC-PRG = 0.28

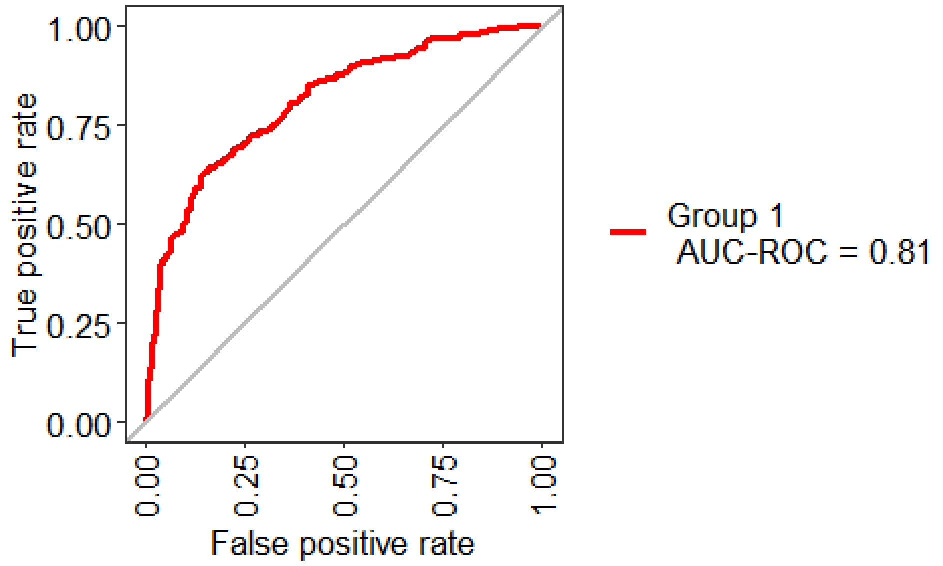

1.00
0.75
0.50
0.25
0.00
True positive rate
0.00
0.25
0.50
0.75
1.00
False positive rate
Group 1
AUC-ROC = 0.81


```
x_xgb$stdres
```

```
## $`Group 1`
##                   Score        CI
## SENS              0.909 0.88-0.93
## SPEC              0.440  0.38-0.5
## MCC               0.403      <NA>
## Informedness      0.348      <NA>
## PREC              0.790 0.76-0.82
## NPV               0.675  0.6-0.75
## FPR               0.560      <NA>
## F1                0.845      <NA>
## TP              488.000      <NA>
## FP              130.000      <NA>
## TN              102.000      <NA>
## FN               49.000      <NA>
## AUC-ROC           0.810 0.78-0.84
## AUC-PR            0.900      <NA>
## AUC-PRG           0.280      <NA>
```

```
test_pred_xgb <- predict(xgb_fit, newdata = testdata)
confusionMatrix(test_pred_xgb, testdata$Outcome)
```

```
## Confusion Matrix and Statistics
## 
##           Reference
## Prediction  X1  X2
##         X1  44  23
##         X2  45 185
## 
##                Accuracy : 0.771
##                  95% CI : (0.719, 0.8176)
##     No Information Rate : 0.7003
##     P-Value [Acc > NIR] : 0.003986
## 
##                   Kappa : 0.413
## 
##  Mcnemar's Test P-Value : 0.010877
## 
##             Sensitivity : 0.4944
##             Specificity : 0.8894
##          Pos Pred Value : 0.6567
##          Neg Pred Value : 0.8043
##              Prevalence : 0.2997
##          Detection Rate : 0.1481
##    Detection Prevalence : 0.2256
##       Balanced Accuracy : 0.6919
## 
##        'Positive' Class : X1
## 
```

```
test_prob_xgb <- predict(xgb_fit, newdata = testdata, type="prob")
mxgb = data.frame(test_prob_xgb, testdata$Outcome)
yxgb<-evalm(mxgb)
```

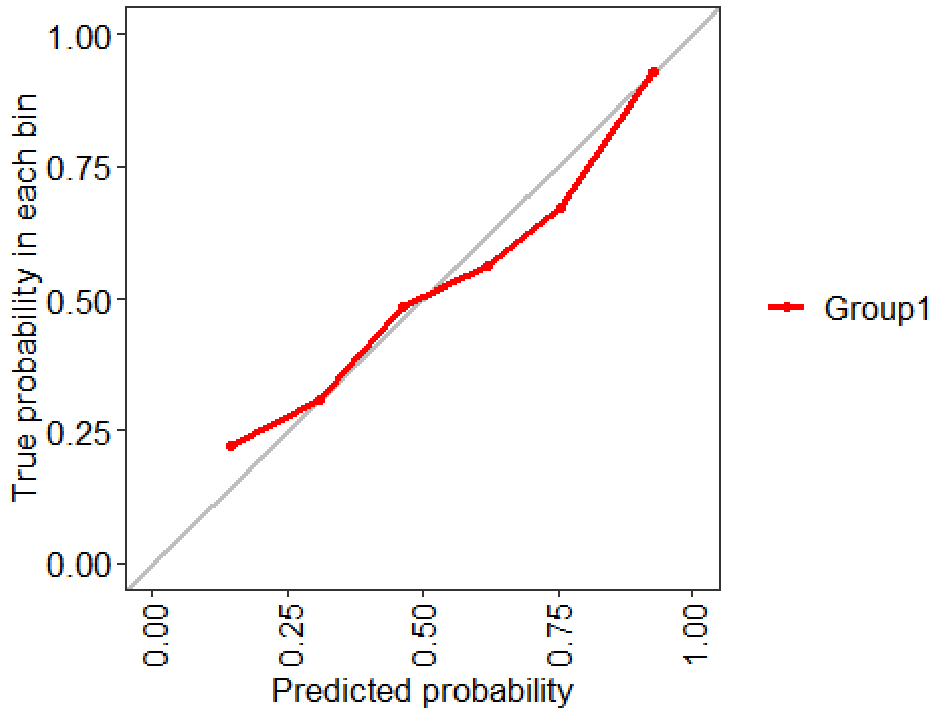

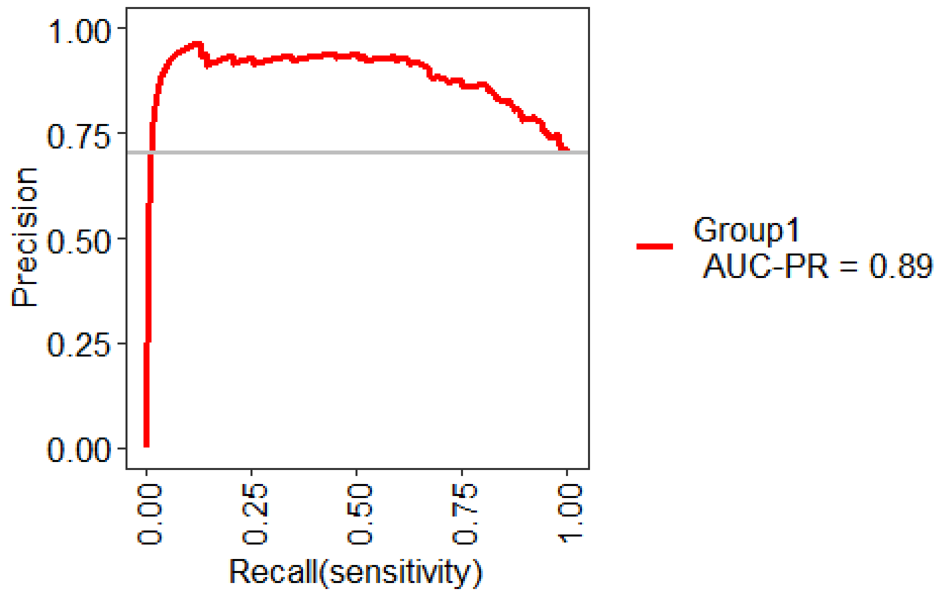

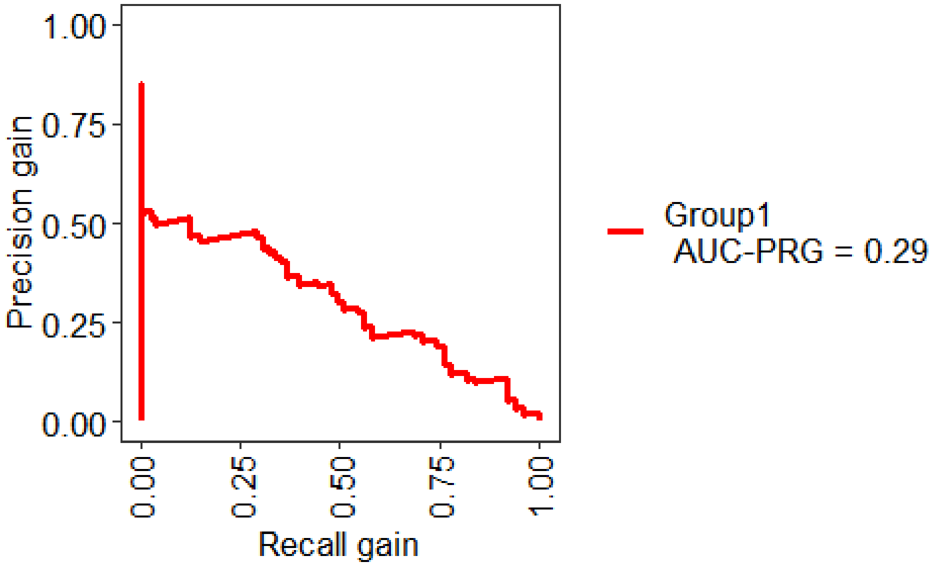
1.00
0.75
0.50
0.25
0.00
Precision gain
0.00
0.25
0.50
0.75
1.00
Recall gain
Group1
AUC-PRG = 0.29

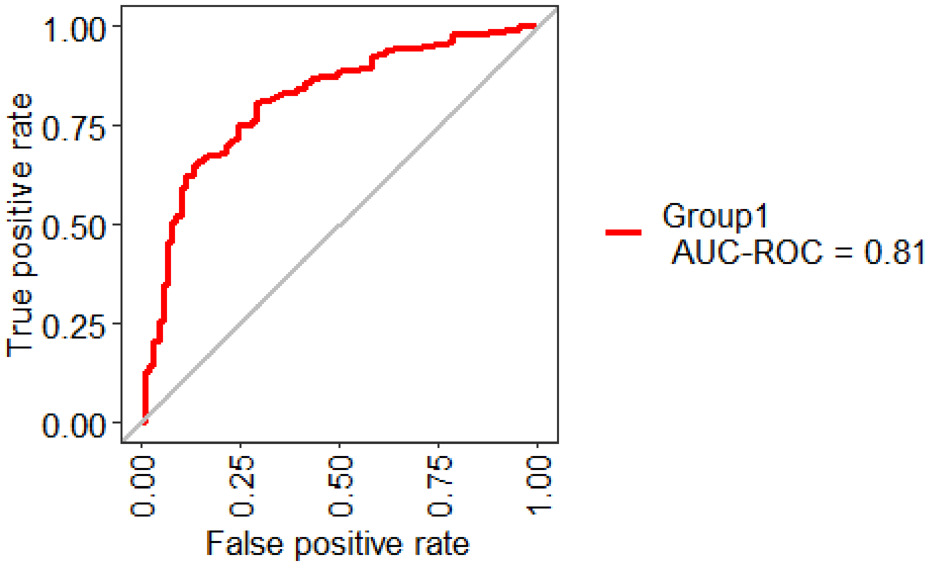
1.00
0.75
0.50
0.25
0.00
True positive rate
0.00
0.25
0.50
0.75
1.00
False positive rate
Group1
AUC-ROC = 0.81

```
yxgb$stdres
```

```
## $Group1
##                   Score         CI
## SENS              0.889 0.84-0.93
## SPEC              0.494  0.39-0.6
## MCC               0.421      <NA>
## Informedness      0.384      <NA>
## PREC              0.804 0.75-0.85
## NPV               0.657 0.54-0.76
## FPR               0.506      <NA>
## F1                0.845      <NA>
## TP              185.000      <NA>
## FP               45.000      <NA>
## TN               44.000      <NA>
## FN               23.000      <NA>
## AUC-ROC           0.810 0.76-0.86
## AUC-PR            0.890      <NA>
## AUC-PRG           0.290      <NA>
```

```
imp<-varImp(xgb_fit, scale = FALSE)
imp
```

```
## xgbTree variable importance
##
##   only 20 most important variables shown (out of 60)
##
##                  Overall
## NIHSS_adm       0.181593
## Age             0.126993
## Arrival         0.052630
## RanGlu          0.050830
## PLT             0.048067
## LDL             0.046479
## SBP             0.044590
## TG              0.041516
## BUN             0.041104
## Hb              0.041096
## BMI             0.039366
## HbA1C           0.039203
## HDL             0.037616
## DBP             0.036614
## TC              0.030849
## AC              0.024639
## Cr              0.018989
## PRE_STROKE.0 0.007174
## PRE_STROKE.2 0.006317
## Side.1          0.005029
```

```
plot(imp)
```

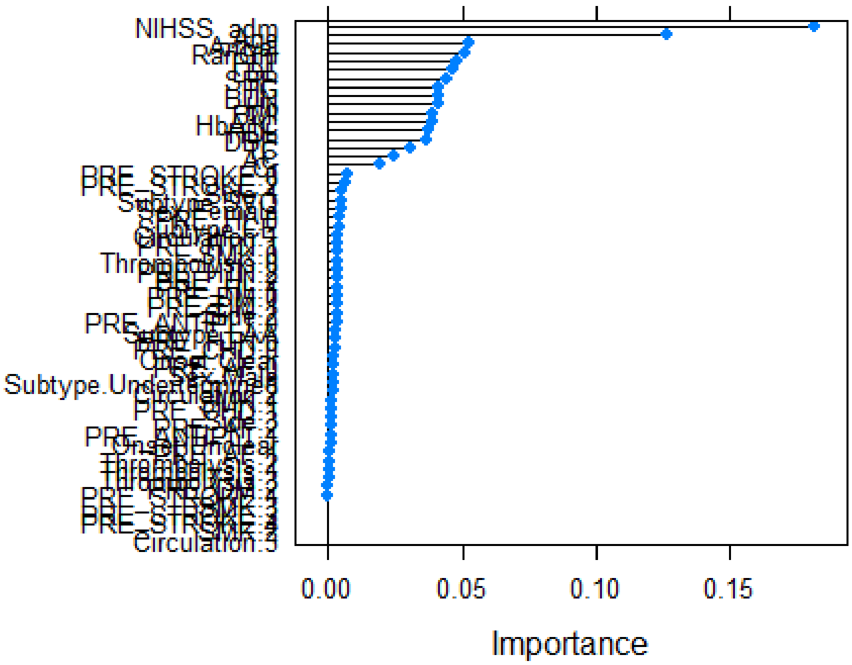


```
set.seed(seed)
svm_fit <- train(Outcome~., data = traindata, method = "svmRadial",
                 trControl=ctrl, metric=metric, tuneLength = 10)
svm_fit

## Support Vector Machines with Radial Basis Function Kernel
##
## 769 samples
##  60 predictor
##   2 classes: 'X1', 'X2'
##
## No pre-processing
## Resampling: Cross-Validated (10 fold, repeated 10 times)
## Summary of sample sizes: 691, 693, 693, 693, 692, 692, ...
## Resampling results across tuning parameters:
##
##   C       ROC        Sens       Spec
##     0.25  0.7808983  0.4439493  0.8882984
##     0.50  0.7792789  0.3826449  0.9091300
##     1.00  0.7750284  0.3722101  0.9080119
##     2.00  0.7699337  0.3684420  0.9145388
##     4.00  0.7562832  0.3404891  0.9200804
##     8.00  0.7360512  0.3188406  0.9193955
##    16.00  0.7211696  0.2944565  0.9197379
##    32.00  0.7169518  0.2866123  0.9227149
##    64.00  0.7136272  0.2917391  0.9184347
```

```
##   128.00  0.7135708  0.2921920  0.9176904
##
## Tuning parameter 'sigma' was held constant at a value of 0.02761147
## ROC was used to select the optimal model using the largest value.
## The final values used for the model were sigma = 0.02761147 and C = 0.25.

x_svm <- evalm(svm_fit)
```

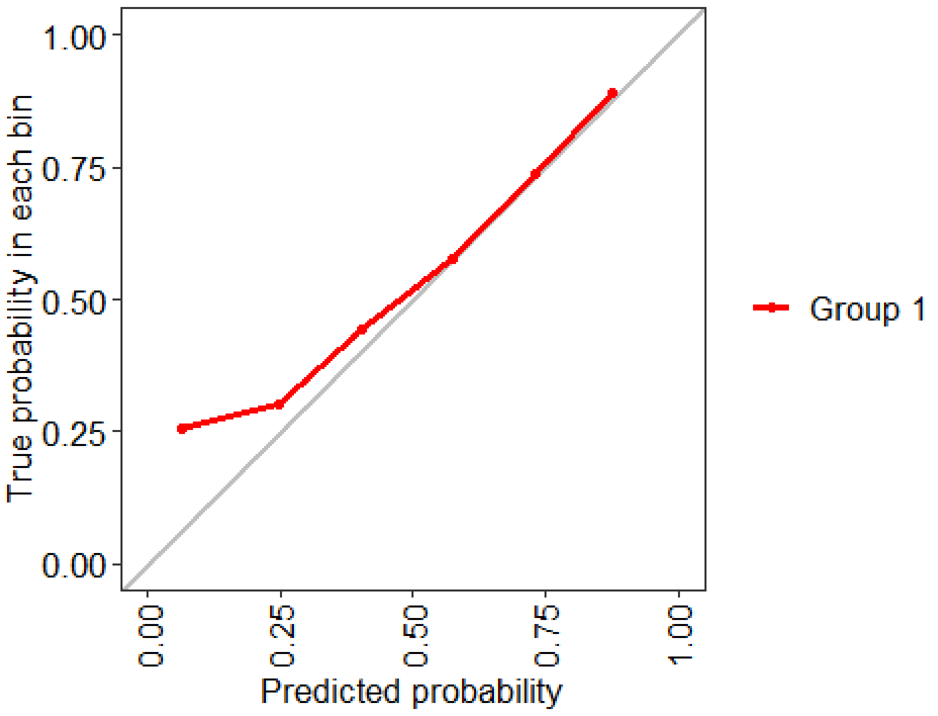

True probability in each bin
1.00
0.75
0.50
0.25
0.00
0.00
0.25
0.50
0.75
1.00
Predicted probability
Group 1

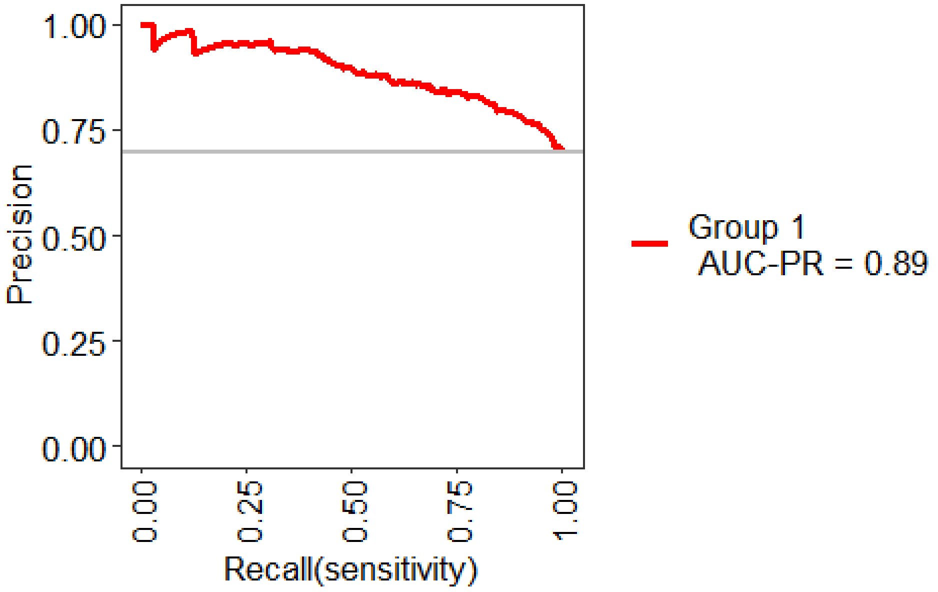

Precision
1.00
0.75
0.50
0.25
0.00
0.00
0.25
0.50
0.75
1.00
Recall(sensitivity)
Group 1
AUC-PR = 0.89

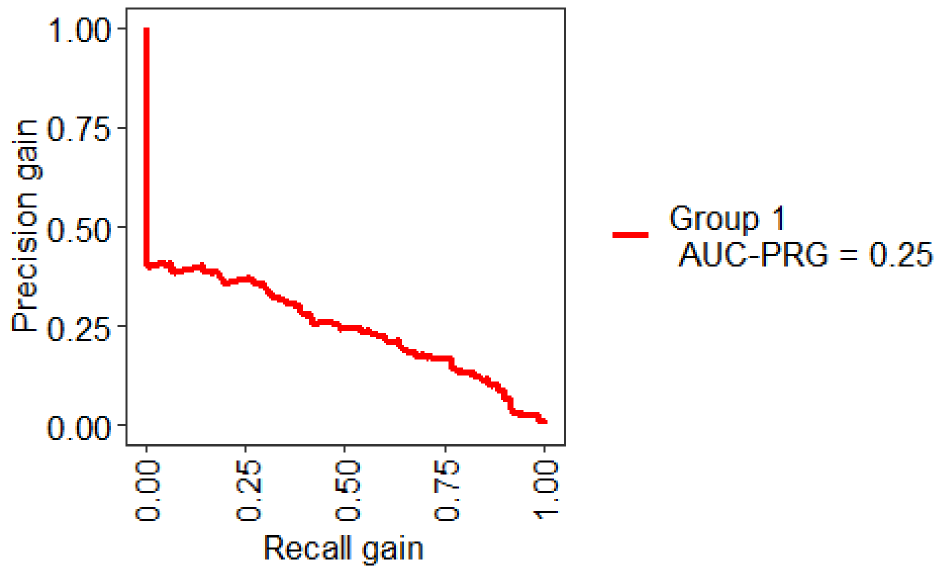
1.00
0.75
0.50
0.25
0.00
Precision gain
0.00
0.25
0.50
0.75
1.00
Recall gain
Group 1
AUC-PRG = 0.25

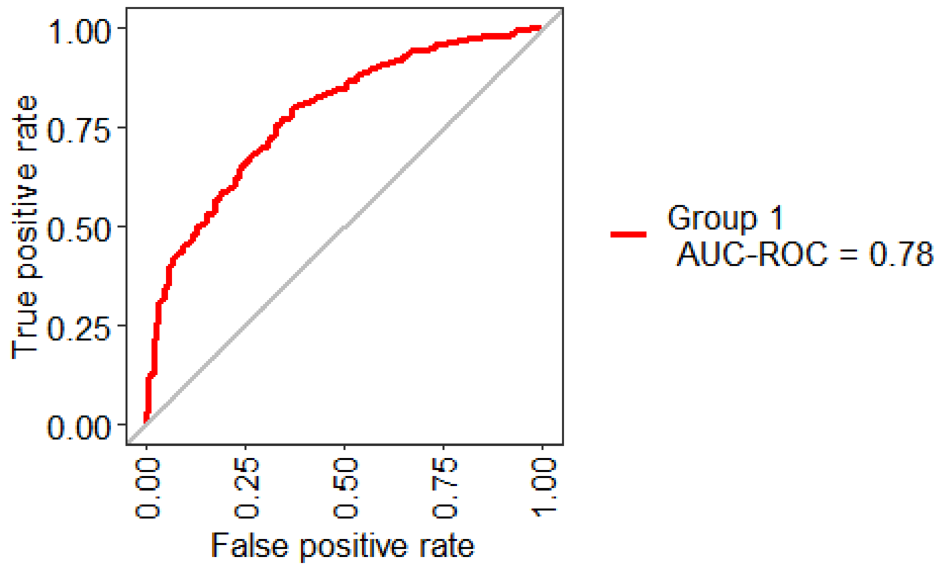
1.00
0.75
0.50
0.25
0.00
True positive rate
0.00
0.25
0.50
0.75
1.00
False positive rate
Group 1
AUC-ROC = 0.78

```
x_svm$stdres
```

```
## $`Group 1`
##                  Score        CI
## SENS             0.888 0.86-0.91
## SPEC             0.448 0.39-0.51
## MCC              0.377      <NA>
## Informedness     0.337      <NA>
## PREC             0.788 0.75-0.82
## NPV              0.634  0.56-0.7
## FPR              0.552      <NA>
## F1               0.835      <NA>
## TP             477.000      <NA>
## FP             128.000      <NA>
## TN             104.000      <NA>
## FN              60.000      <NA>
## AUC-ROC          0.780 0.75-0.81
## AUC-PR           0.890      <NA>
## AUC-PRG          0.250      <NA>
```

```
test_pred_svm <- predict(svm_fit, newdata = testdata)
confusionMatrix (test_pred_svm, testdata$Outcome)
```

```
## Confusion Matrix and Statistics
##
##             Reference
## Prediction  X1  X2
##         X1  50  20
##         X2  39 188
##
##                Accuracy : 0.8013
##                  95% CI : (0.7514, 0.8452)
##     No Information Rate : 0.7003
##     P-Value [Acc > NIR] : 5.361e-05
##
##                   Kappa : 0.4959
##
##  Mcnemar's Test P-Value : 0.01911
##
##             Sensitivity : 0.5618
##             Specificity : 0.9038
##          Pos Pred Value : 0.7143
##          Neg Pred Value : 0.8282
##              Prevalence : 0.2997
##          Detection Rate : 0.1684
##    Detection Prevalence : 0.2357
##       Balanced Accuracy : 0.7328
##
##        'Positive' Class : X1
##
```

```
test_prob_svm <- predict(svm_fit, newdata = testdata, type="prob")
msvm = data.frame(test_prob_svm, testdata$Outcome)
ysvm<-evalm(msvm)
```

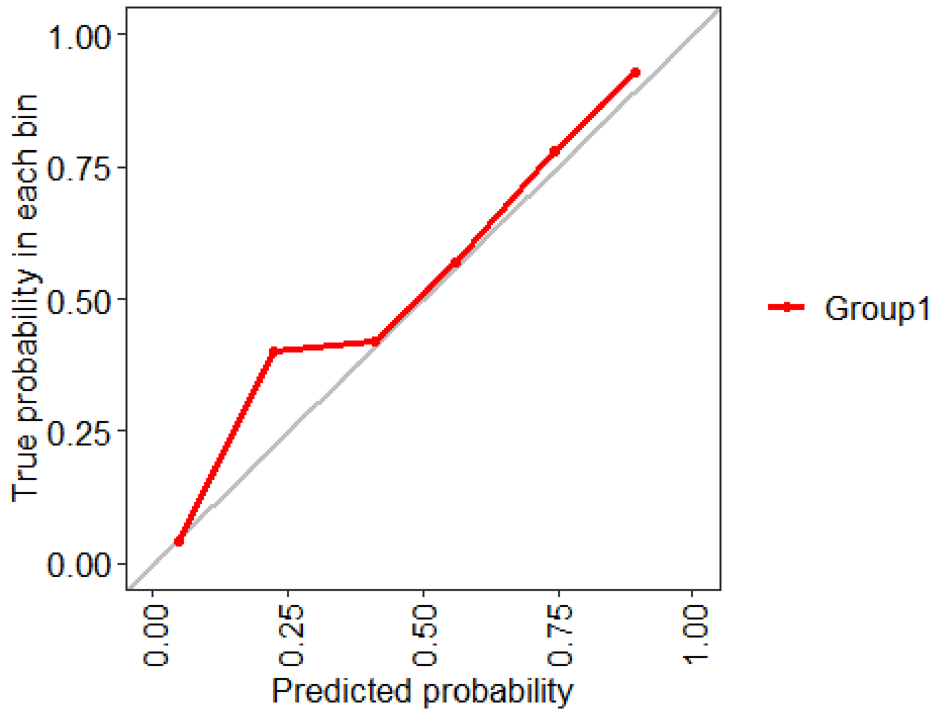

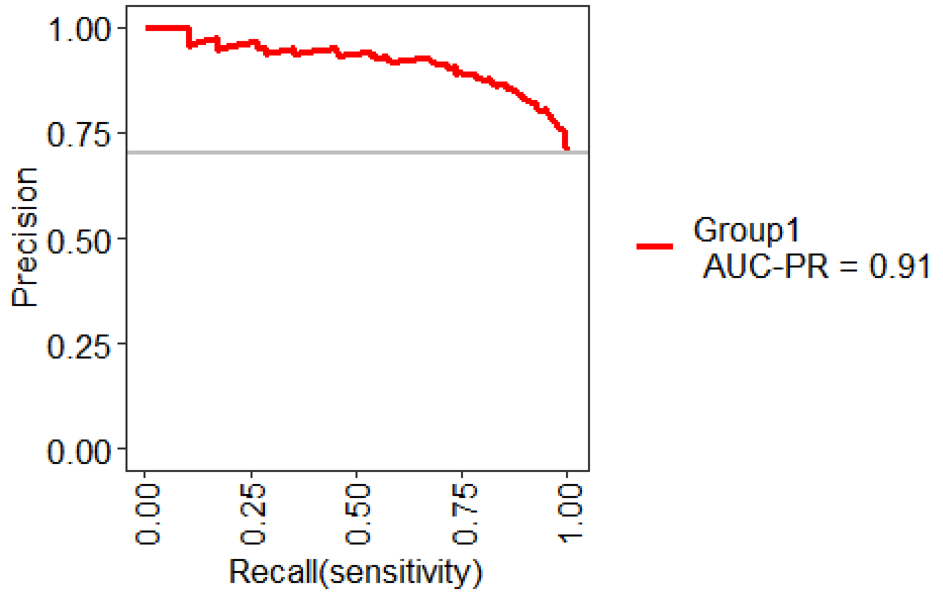

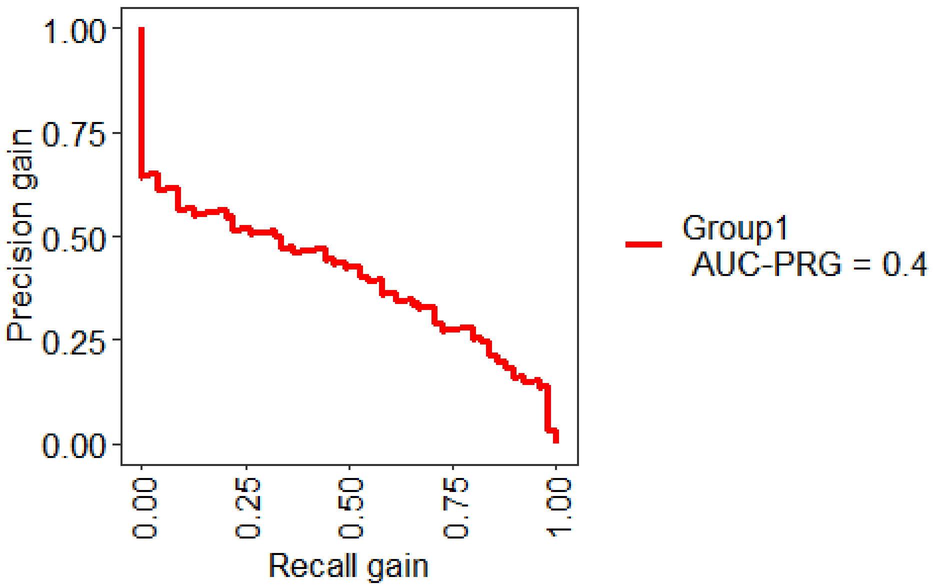

1.00
0.75
0.50
0.25
0.00
Precision gain
0.00
0.25
0.50
0.75
1.00
Recall gain
Group1
AUC-PRG = 0.4

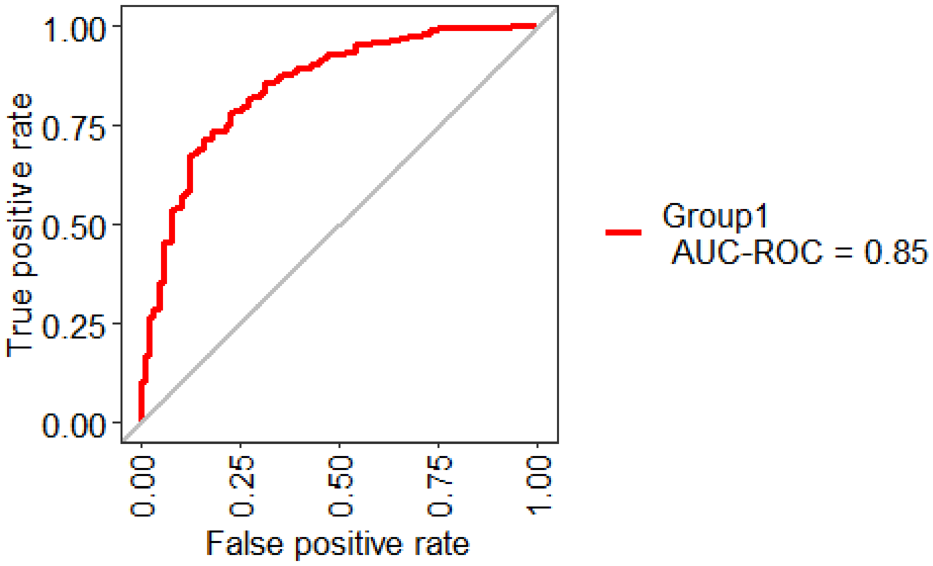

1.00
0.75
0.50
0.25
0.00
True positive rate
0.00
0.25
0.50
0.75
1.00
False positive rate
Group1
AUC-ROC = 0.85


```
ysvm$stdres
```

```
## $Group1
##                   Score        CI
## SENS              0.904 0.86-0.94
## SPEC              0.562 0.46-0.66
## MCC               0.503      <NA>
## Informedness      0.466      <NA>
## PREC              0.828 0.77-0.87
## NPV               0.714  0.6-0.81
## FPR               0.438      <NA>
## F1                0.864      <NA>
## TP              188.000      <NA>
## FP               39.000      <NA>
## TN               50.000      <NA>
## FN               20.000      <NA>
## AUC-ROC           0.850 0.81-0.89
## AUC-PR            0.910      <NA>
## AUC-PRG           0.400      <NA>
```

```
set.seed(seed)
knn_fit <- train(Outcome~., data = traindata, method = "knn",
                 trControl=ctrl, metric=metric, tuneLength = 50)
knn_fit
```

```
## k-Nearest Neighbors
##
## 769 samples
##  60 predictor
##   2 classes: 'X1', 'X2'
##
## No pre-processing
## Resampling: Cross-Validated (10 fold, repeated 10 times)
## Summary of sample sizes: 691, 693, 693, 693, 692, 692, ...
## Resampling results across tuning parameters:
##
##   k   ROC        Sens        Spec
##    5  0.6366825  0.21929348  0.8984906
##    7  0.6452075  0.20943841  0.9301817
##    9  0.6673790  0.19773551  0.9450664
##   11  0.6797653  0.18262681  0.9558910
##   13  0.6881678  0.16121377  0.9689133
##   15  0.6956531  0.15681159  0.9709539
##   17  0.7019509  0.15804348  0.9791405
##   19  0.7044948  0.15163043  0.9821279
##   21  0.7065721  0.14262681  0.9836094
##   23  0.7108257  0.13311594  0.9845353
##   25  0.7177049  0.11764493  0.9850908
##   27  0.7176241  0.11376812  0.9843431
##   29  0.7182129  0.11375000  0.9837945
##   31  0.7155400  0.10994565  0.9837876
```

```
##    33  0.7177519  0.10173913  0.9849092
##    35  0.7225859  0.09916667  0.9850943
##    37  0.7236358  0.09916667  0.9852795
##    39  0.7249821  0.09739130  0.9865828
##    41  0.7250602  0.09396739  0.9873166
##    43  0.7252732  0.09226449  0.9878791
##    45  0.7250579  0.08797101  0.9873201
##    47  0.7242378  0.08534420  0.9862089
##    49  0.7222957  0.08407609  0.9856464
##    51  0.7218365  0.08231884  0.9854647
##    53  0.7213997  0.08059783  0.9856464
##    55  0.7214036  0.07844203  0.9856464
##    57  0.7206454  0.07802536  0.9865793
##    59  0.7197855  0.07760870  0.9878791
##    61  0.7202359  0.07677536  0.9882495
##    63  0.7211058  0.07460145  0.9884347
##    65  0.7215769  0.07201087  0.9888050
##    67  0.7244282  0.07115942  0.9889937
##    69  0.7281901  0.07117754  0.9897379
##    71  0.7304029  0.06775362  0.9902970
##    73  0.7326776  0.06900362  0.9906709
##    75  0.7336795  0.06900362  0.9904857
##    77  0.7333991  0.06599638  0.9908595
##    79  0.7347630  0.06684783  0.9910447
##    81  0.7360292  0.06469203  0.9921698
##    83  0.7381157  0.06340580  0.9934696
##    85  0.7375543  0.05992754  0.9936583
##    87  0.7372151  0.06125000  0.9944060
##    89  0.7354903  0.05909420  0.9947834
##    91  0.7358800  0.05865942  0.9955311
##    93  0.7343451  0.05692029  0.9951572
##    95  0.7331492  0.05393116  0.9960832
##    97  0.7314851  0.05304348  0.9960867
##    99  0.7317255  0.04916667  0.9962753
##   101  0.7324770  0.04742754  0.9970196
##   103  0.7319719  0.04570652  0.9973934
##
## ROC was used to select the optimal model using the largest value.
## The final value used for the model was k = 83.

x_knn <- evalm(knn_fit)
```

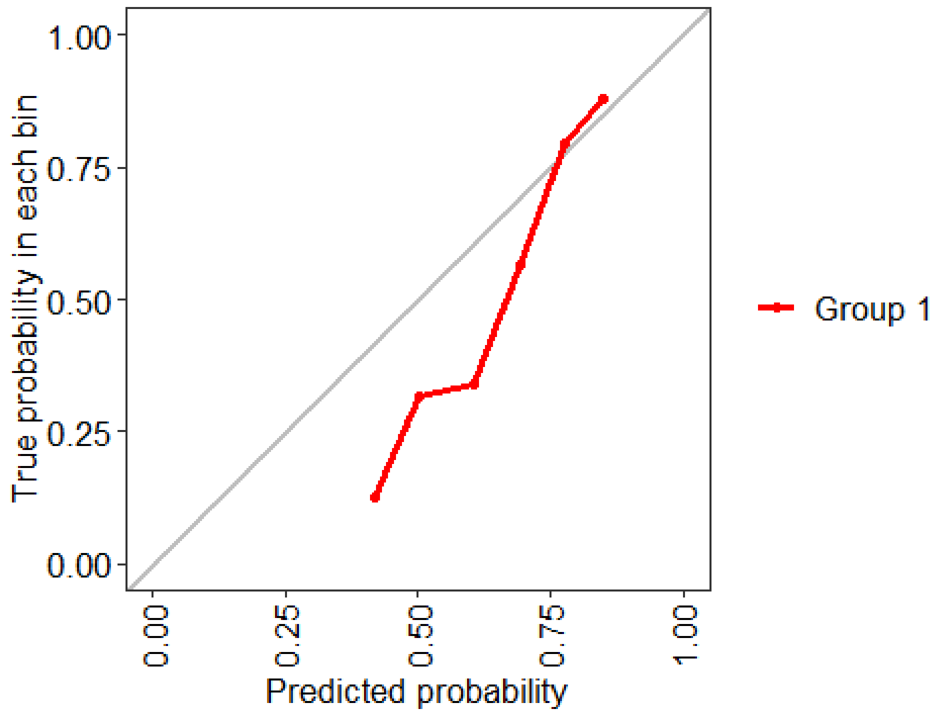
1.00
0.75
0.50
0.25
0.00
True probability in each bin
0.00
0.25
0.50
0.75
1.00
Predicted probability
Group 1

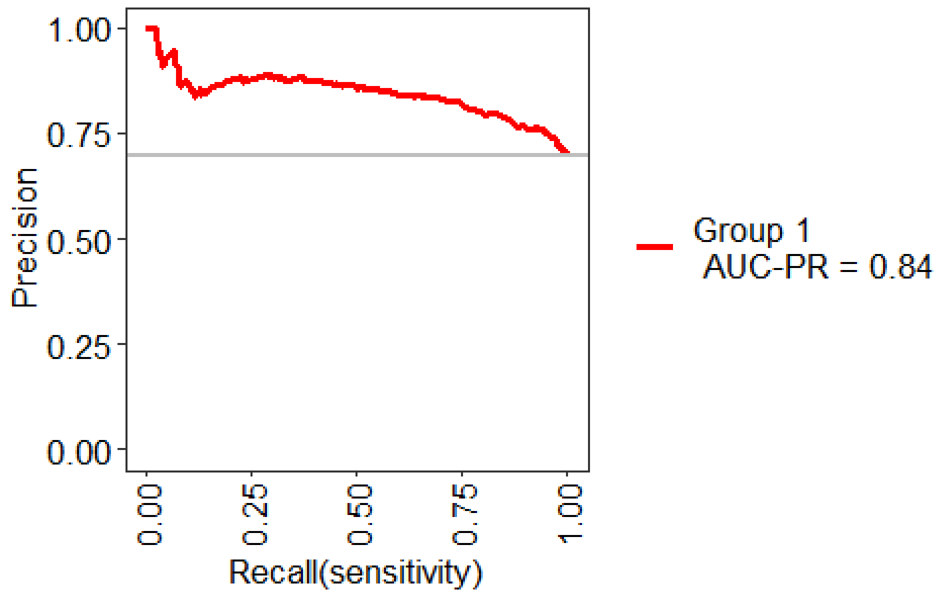
1.00
0.75
0.50
0.25
0.00
Precision
0.00
0.25
0.50
0.75
1.00
Recall(sensitivity)
Group 1
AUC-PR = 0.84

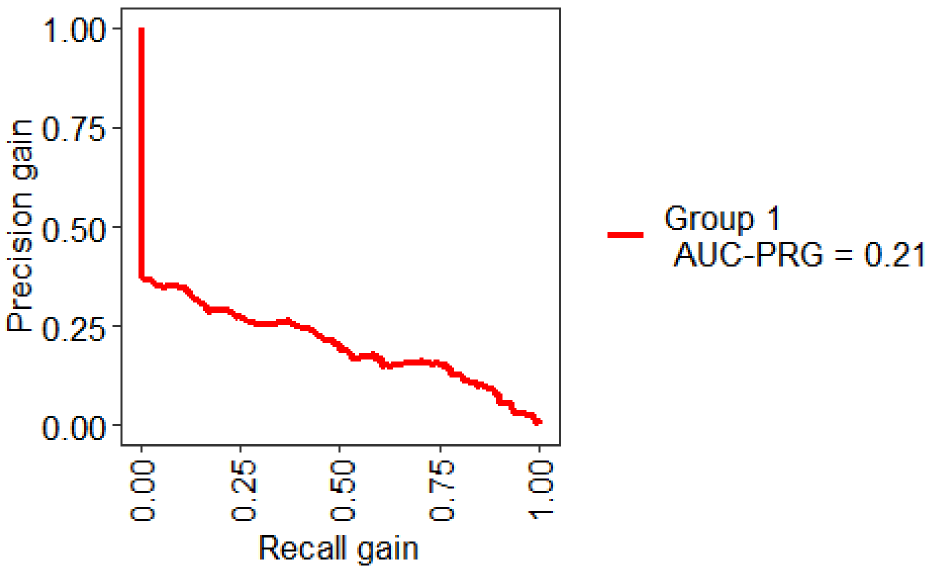
1.00
0.75
0.50
0.25
0.00
Precision gain
0.00
0.25
0.50
0.75
1.00
Recall gain
Group 1
AUC-PRG = 0.21

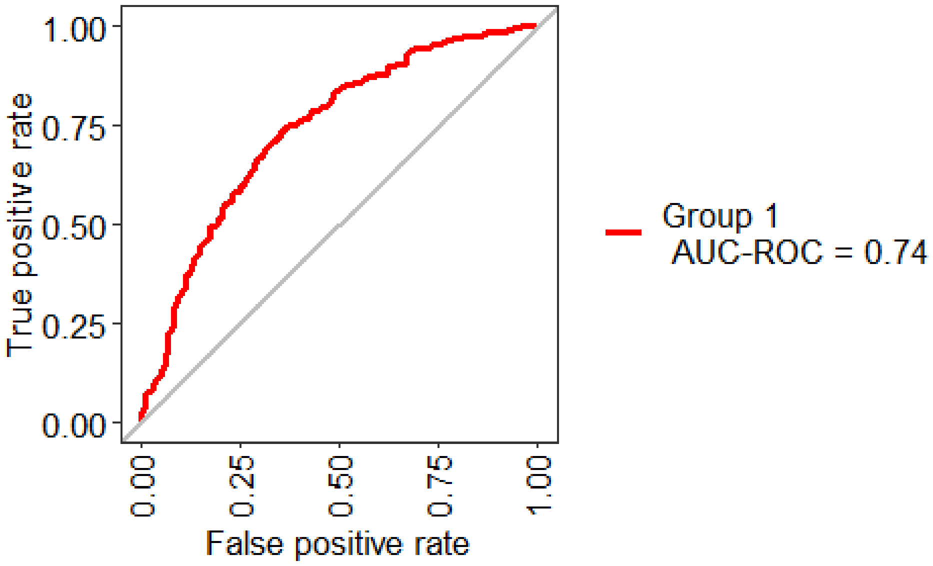
1.00
0.75
0.50
0.25
0.00
True positive rate
0.00
0.25
0.50
0.75
1.00
False positive rate
Group 1
AUC-ROC = 0.74

```
x_knn$stdres
```

```
## $`Group 1`
##                   Score        CI
## SENS              0.993    0.98-1
## SPEC              0.060  0.04-0.1
## MCC               0.161      <NA>
## Informedness      0.053      <NA>
## PREC              0.710 0.68-0.74
## NPV               0.778 0.55-0.91
## FPR               0.940      <NA>
## F1                0.828      <NA>
## TP              533.000      <NA>
## FP              218.000      <NA>
## TN               14.000      <NA>
## FN                4.000      <NA>
## AUC-ROC           0.740  0.7-0.78
## AUC-PR            0.840      <NA>
## AUC-PRG           0.210      <NA>
```

```
test_pred_knn <- predict(knn_fit, newdata = testdata)
confusionMatrix(test_pred_knn, testdata$Outcome)
```

```
## Confusion Matrix and Statistics
##
##           Reference
## Prediction  X1  X2
##         X1   9   1
##         X2  80 207
##
##                Accuracy : 0.7273
##                  95% CI : (0.6728, 0.7771)
##     No Information Rate : 0.7003
##     P-Value [Acc > NIR] : 0.1713
##
##                   Kappa : 0.1291
##
##  Mcnemar's Test P-Value : <2e-16
##
##             Sensitivity : 0.10112
##             Specificity : 0.99519
##          Pos Pred Value : 0.90000
##          Neg Pred Value : 0.72125
##              Prevalence : 0.29966
##          Detection Rate : 0.03030
##    Detection Prevalence : 0.03367
##       Balanced Accuracy : 0.54816
##
##        'Positive' Class : X1
##
```

```
test_prob_knn <- predict(knn_fit, newdata = testdata, type="prob")
mknn = data.frame(test_prob_knn, testdata$Outcome)
yknn<-evalm(mknn)
```

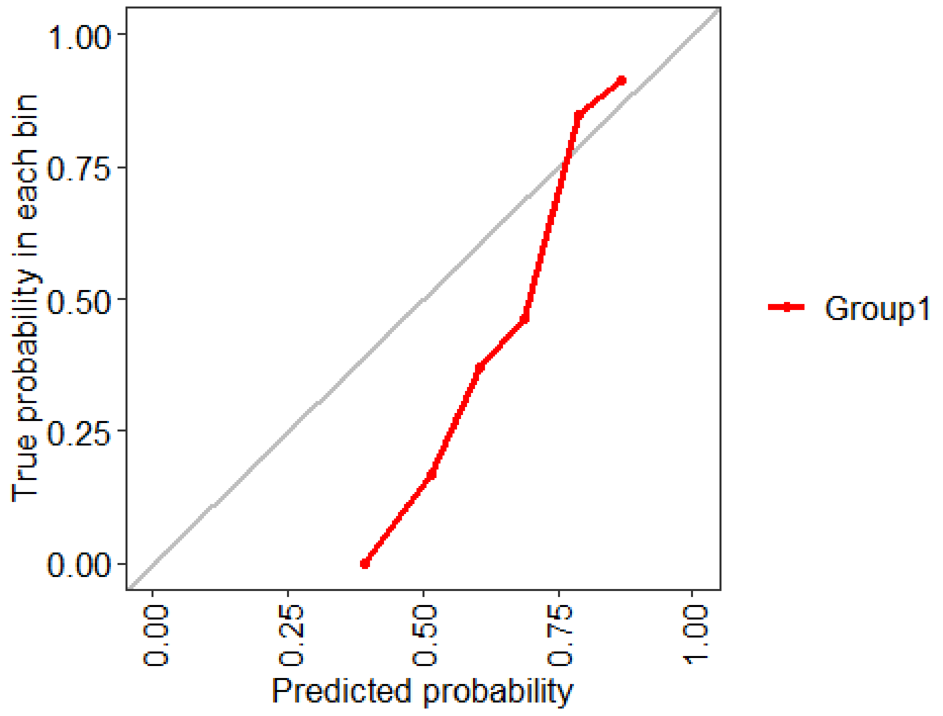

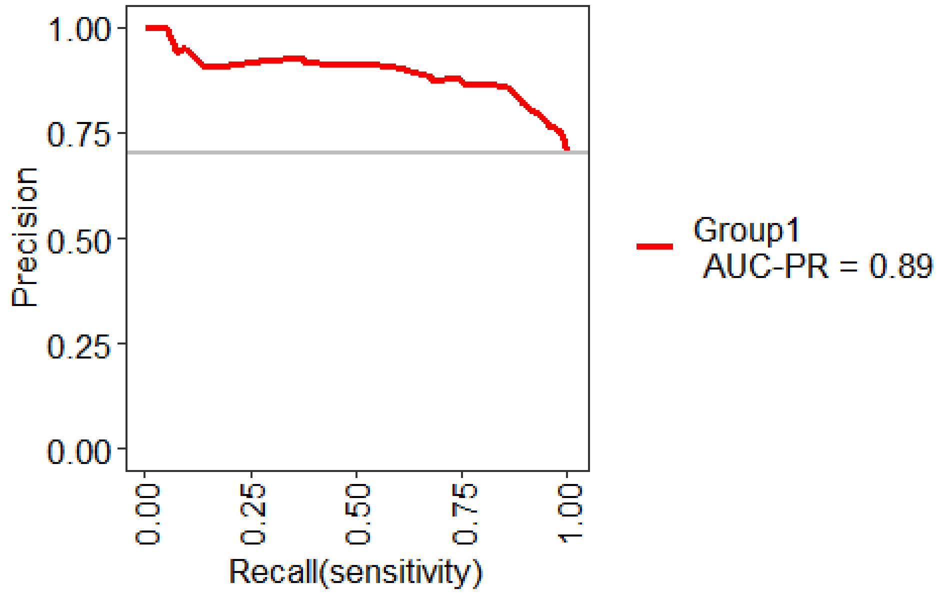

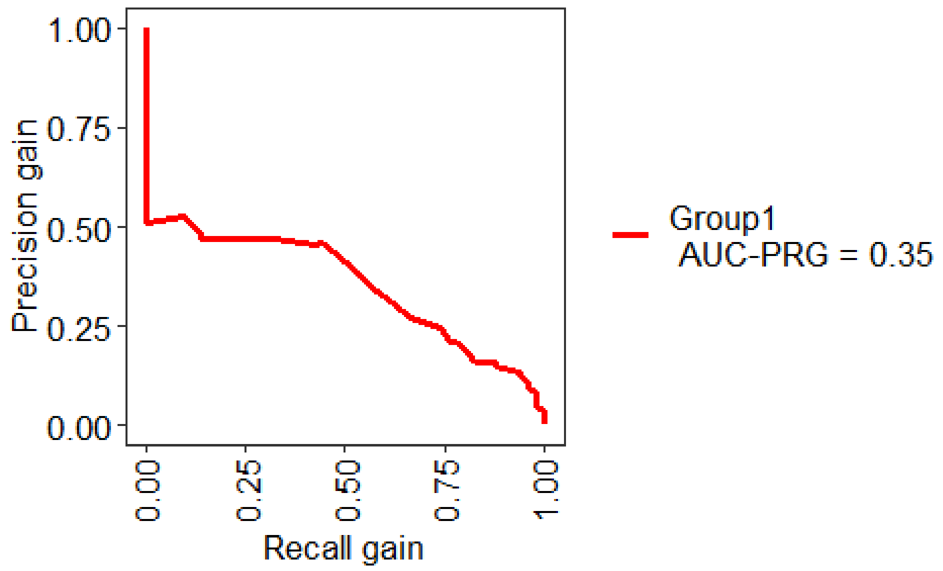
1.00
0.75
0.50
0.25
0.00
Precision gain
0.00
0.25
0.50
0.75
1.00
Recall gain
Group1
AUC-PRG = 0.35

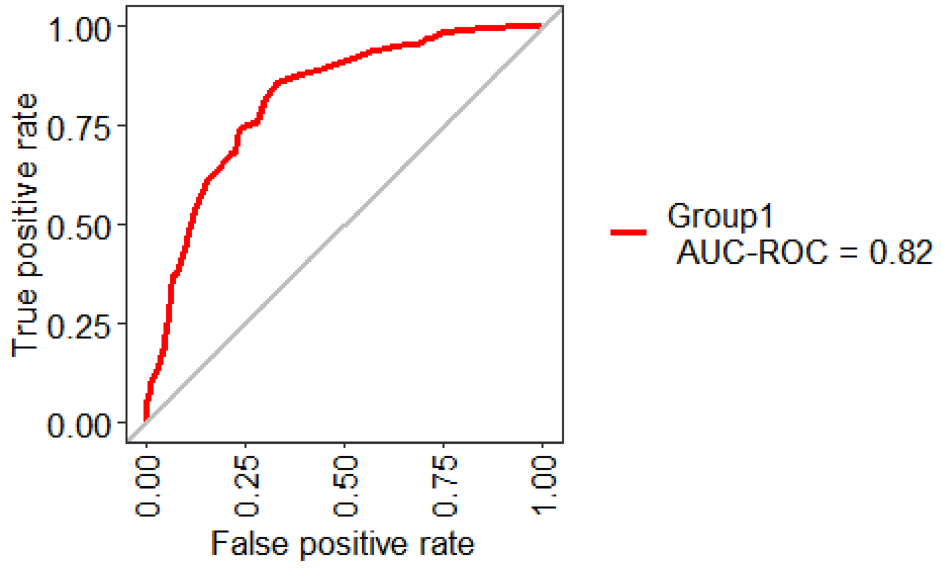
1.00
0.75
0.50
0.25
0.00
True positive rate
0.00
0.25
0.50
0.75
1.00
False positive rate
Group1
AUC-ROC = 0.82

```
yknn$stdres
```

```
## $Group1
##                    Score        CI
## SENS               0.995    0.97-1
## SPEC               0.101 0.05-0.18
## MCC                0.245      <NA>
## Informedness       0.096      <NA>
## PREC               0.721 0.67-0.77
## NPV                0.900  0.6-0.98
## FPR                0.899      <NA>
## F1                 0.836      <NA>
## TP               207.000      <NA>
## FP                80.000      <NA>
## TN                 9.000      <NA>
## FN                 1.000      <NA>
## AUC-ROC            0.820 0.77-0.87
## AUC-PR             0.890      <NA>
## AUC-PRG            0.350      <NA>
```

```
set.seed(seed)
lr_fit<-train(Outcome~., data = traindata, method = "regLogistic",
              trControl=ctrl, metric=metric, tuneLength=5, verbose=FALSE)
lr_fit
```

```
## Regularized Logistic Regression
##
## 769 samples
##  60 predictor
##   2 classes: 'X1', 'X2'
##
## No pre-processing
## Resampling: Cross-Validated (10 fold, repeated 10 times)
## Summary of sample sizes: 691, 693, 693, 693, 692, 692, ...
## Resampling results across tuning parameters:
##
##   cost  loss       epsilon  ROC        Sens       Spec
##   0.25  L1         1e-04    0.7704150  0.3718297  0.9232914
##   0.25  L1         1e-03    0.7708139  0.3705435  0.9232879
##   0.25  L1         1e-02    0.7712565  0.3718297  0.9234696
##   0.25  L1         1e-01    0.7696067  0.3705978  0.9258875
##   0.25  L1         1e+00    0.7463236  0.3392935  0.9244514
##   0.25  L2_dual    1e-04    0.7559086  0.3821558  0.9117470
##   0.25  L2_dual    1e-03    0.7559086  0.3821558  0.9117470
##   0.25  L2_dual    1e-02    0.7559094  0.3821558  0.9117470
##   0.25  L2_dual    1e-01    0.7559179  0.3821558  0.9117470
##   0.25  L2_dual    1e+00    0.7560718  0.3821739  0.9126695
##   0.25  L2_primal  1e-04    0.7559327  0.3821558  0.9117470
##   0.25  L2_primal  1e-03    0.7559813  0.3821558  0.9117470
##   0.25  L2_primal  1e-02    0.7558863  0.3825906  0.9117470
##   0.25  L2_primal  1e-01    0.7557225  0.3804529  0.9128651
```

```
##    0.25  L2_primal  1e+00    0.7604857  0.3232428  0.9343047
##    0.50  L1         1e-04    0.7640213  0.3774094  0.9165723
##    0.50  L1         1e-03    0.7638715  0.3778442  0.9171349
##    0.50  L1         1e-02    0.7640397  0.3795652  0.9173096
##    0.50  L1         1e-01    0.7654522  0.3735688  0.9232949
##    0.50  L1         1e+00    0.7470772  0.3504529  0.9161880
##    0.50  L2_dual    1e-04    0.7534142  0.3878442  0.9080049
##    0.50  L2_dual    1e-03    0.7534142  0.3878442  0.9080049
##    0.50  L2_dual    1e-02    0.7533980  0.3878442  0.9080049
##    0.50  L2_dual    1e-01    0.7534859  0.3878442  0.9081901
##    0.50  L2_dual    1e+00    0.7532163  0.3895833  0.9068938
##    0.50  L2_primal  1e-04    0.7534062  0.3878442  0.9080049
##    0.50  L2_primal  1e-03    0.7534364  0.3874094  0.9080049
##    0.50  L2_primal  1e-02    0.7534502  0.3878261  0.9076345
##    0.50  L2_primal  1e-01    0.7537629  0.3869565  0.9078267
##    0.50  L2_primal  1e+00    0.7604543  0.3262500  0.9333683
##    1.00  L1         1e-04    0.7566451  0.3869565  0.9085744
##    1.00  L1         1e-03    0.7568508  0.3865399  0.9083892
##    1.00  L1         1e-02    0.7581234  0.3891486  0.9098777
##    1.00  L1         1e-01    0.7581455  0.3826812  0.9145353
##    1.00  L1         1e+00    0.7383599  0.3434601  0.9191824
##    1.00  L2_dual    1e-04    0.7511148  0.3917029  0.9061495
##    1.00  L2_dual    1e-03    0.7511148  0.3917029  0.9061495
##    1.00  L2_dual    1e-02    0.7511386  0.3917029  0.9061495
##    1.00  L2_dual    1e-01    0.7511382  0.3921377  0.9059644
##    1.00  L2_dual    1e+00    0.7505362  0.3925543  0.9063277
##    1.00  L2_primal  1e-04    0.7511220  0.3917029  0.9061495
##    1.00  L2_primal  1e-03    0.7511311  0.3917029  0.9059644
##    1.00  L2_primal  1e-02    0.7511814  0.3921377  0.9057757
##    1.00  L2_primal  1e-01    0.7513556  0.3886957  0.9065269
##    1.00  L2_primal  1e+00    0.7603185  0.3271196  0.9326240
##    2.00  L1         1e-04    0.7527723  0.3912681  0.9066981
##    2.00  L1         1e-03    0.7529073  0.3912681  0.9067016
##    2.00  L1         1e-02    0.7528581  0.3908152  0.9065094
##    2.00  L1         1e-01    0.7529134  0.3809783  0.9098777
##    2.00  L1         1e+00    0.7350363  0.3613406  0.9121174
##    2.00  L2_dual    1e-04    0.7490226  0.3942935  0.9048498
##    2.00  L2_dual    1e-03    0.7490071  0.3942935  0.9048498
##    2.00  L2_dual    1e-02    0.7489907  0.3942935  0.9048498
##    2.00  L2_dual    1e-01    0.7490551  0.3942935  0.9052236
##    2.00  L2_dual    1e+00    0.7490489  0.3981522  0.9042942
##    2.00  L2_primal  1e-04    0.7490068  0.3942935  0.9048498
##    2.00  L2_primal  1e-03    0.7491439  0.3942935  0.9048532
##    2.00  L2_primal  1e-02    0.7493408  0.3942935  0.9052201
##    2.00  L2_primal  1e-01    0.7508424  0.3921196  0.9037317
##    2.00  L2_primal  1e+00    0.7603516  0.3275543  0.9324389
##    4.00  L1         1e-04    0.7502136  0.3951449  0.9039133
##    4.00  L1         1e-03    0.7501890  0.3947101  0.9040985
##    4.00  L1         1e-02    0.7506978  0.3951449  0.9024284
```

```
##   4.00  L1         1e-01    0.7501313  0.3808333  0.9052306
##   4.00  L1         1e+00    0.7286558  0.3478804  0.9078267
##   4.00  L2_dual    1e-04    0.7470167  0.3960145  0.9027952
##   4.00  L2_dual    1e-03    0.7470167  0.3960145  0.9027952
##   4.00  L2_dual    1e-02    0.7470328  0.3960145  0.9027952
##   4.00  L2_dual    1e-01    0.7470640  0.3960145  0.9027918
##   4.00  L2_dual    1e+00    0.7470835  0.3981341  0.9015059
##   4.00  L2_primal  1e-04    0.7470330  0.3960145  0.9027952
##   4.00  L2_primal  1e-03    0.7471897  0.3960145  0.9031656
##   4.00  L2_primal  1e-02    0.7476581  0.3951449  0.9035430
##   4.00  L2_primal  1e-01    0.7501897  0.3938587  0.9022432
##   4.00  L2_primal  1e+00    0.7603684  0.3275543  0.9324389
##
## ROC was used to select the optimal model using the largest value.
## The final values used for the model were cost = 0.25, loss = L1 and epsilo
n
##  = 0.01.
```

```
xlr <- evalm(lr_fit)
```

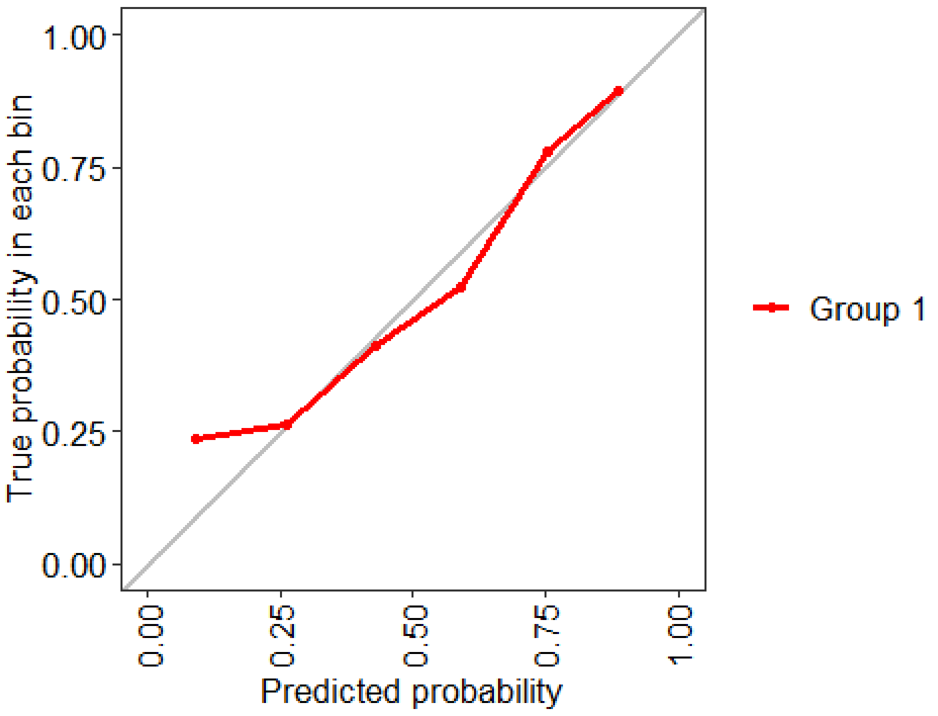
1.00
0.75
0.50
0.25
0.00
True probability in each bin
0.00
0.25
0.50
0.75
1.00
Predicted probability
Group 1

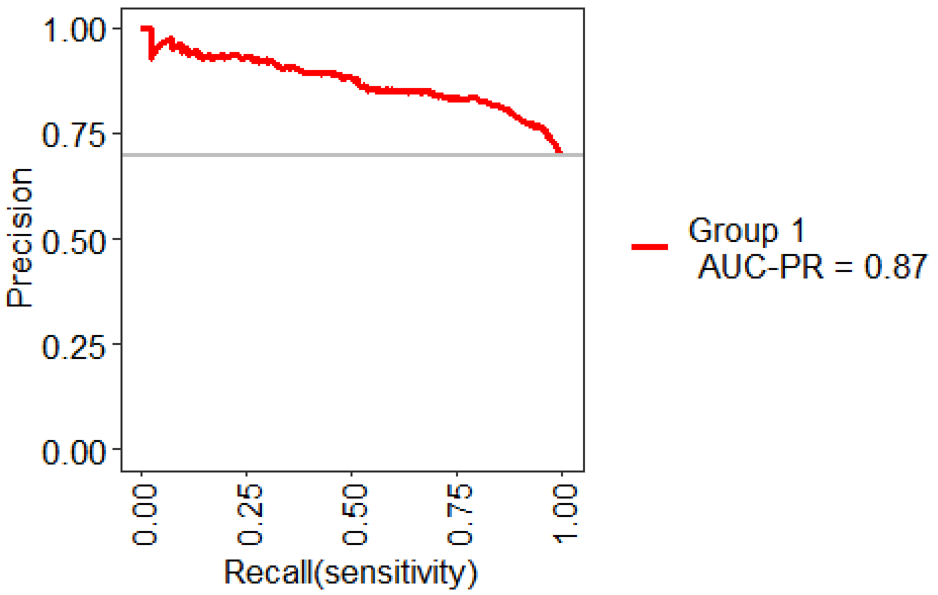
1.00
0.75
0.50
0.25
0.00
Precision
0.00
0.25
0.50
0.75
1.00
Recall(sensitivity)
Group 1
AUC-PR = 0.87

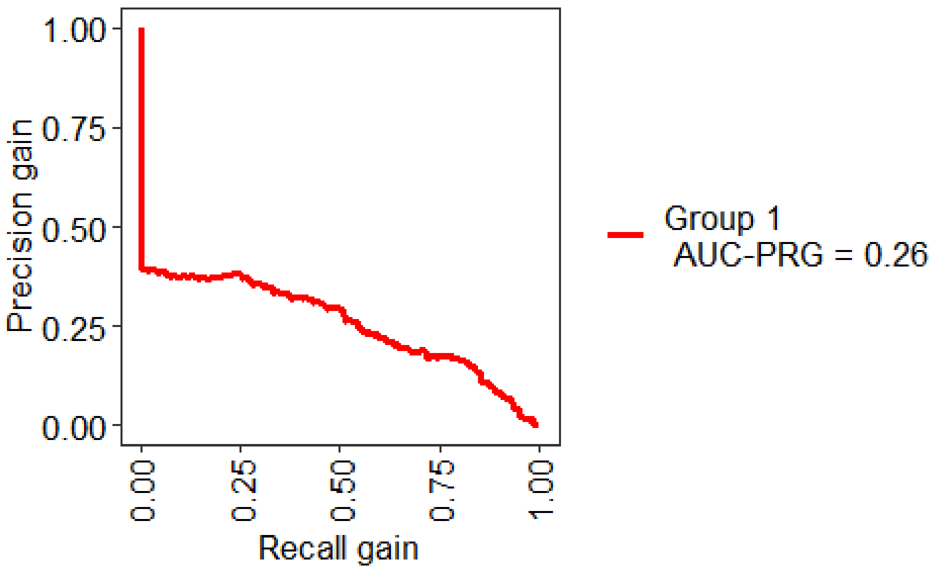
1.00
0.75
0.50
0.25
0.00
Precision gain
0.00
0.25
0.50
0.75
1.00
Recall gain
Group 1
AUC-PRG = 0.26

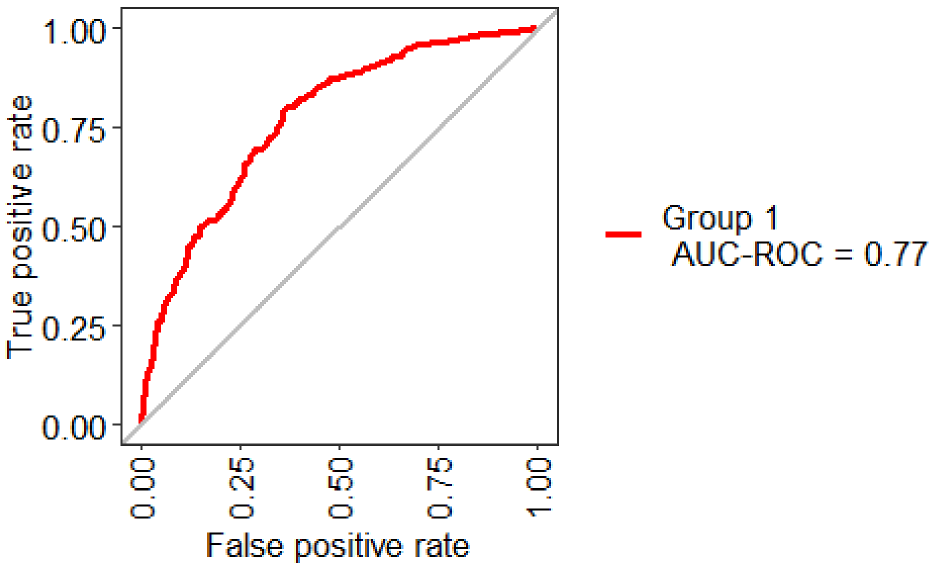
1.00
0.75
0.50
0.25
0.00
True positive rate
0.00
0.25
0.50
0.75
1.00
False positive rate
Group 1
AUC-ROC = 0.77

```
xlr$stdres
```

```
## $`Group 1`
##                  Score        CI
## SENS             0.924  0.9-0.94
## SPEC             0.366 0.31-0.43
## MCC              0.360      <NA>
## Informedness     0.290      <NA>
## PREC             0.771  0.74-0.8
## NPV              0.675 0.59-0.75
## FPR              0.634      <NA>
## F1               0.841      <NA>
## TP             496.000      <NA>
## FP             147.000      <NA>
## TN              85.000      <NA>
## FN              41.000      <NA>
## AUC-ROC          0.770  0.74-0.8
## AUC-PR           0.870      <NA>
## AUC-PRG          0.260      <NA>
```

```
test_pred_lr <- predict(lr_fit, newdata = testdata)
confusionMatrix(test_pred_lr, testdata$Outcome)
```

```
## Confusion Matrix and Statistics
##
##             Reference
## Prediction   X1  X2
##         X1   38  12
##         X2   51 196
##
##                Accuracy : 0.7879
##                  95% CI : (0.7369, 0.833)
##     No Information Rate : 0.7003
##     P-Value [Acc > NIR] : 0.0004396
##
##                   Kappa : 0.4222
##
##  Mcnemar's Test P-Value : 1.688e-06
##
##             Sensitivity : 0.4270
##             Specificity : 0.9423
##          Pos Pred Value : 0.7600
##          Neg Pred Value : 0.7935
##              Prevalence : 0.2997
##          Detection Rate : 0.1279
##    Detection Prevalence : 0.1684
##       Balanced Accuracy : 0.6846
##
##        'Positive' Class : X1
##
```

```
test_prob_lr <- predict(lr_fit, newdata = testdata, type="prob")
mlr = data.frame(test_prob_lr, testdata$Outcome)
ylr<-evalm(mlr)
```

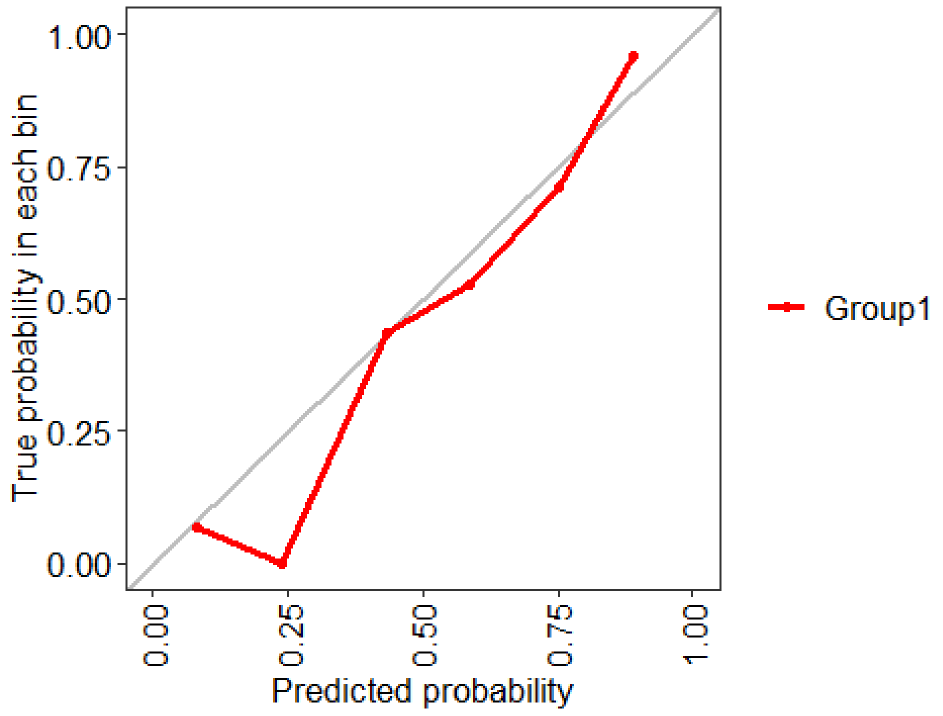

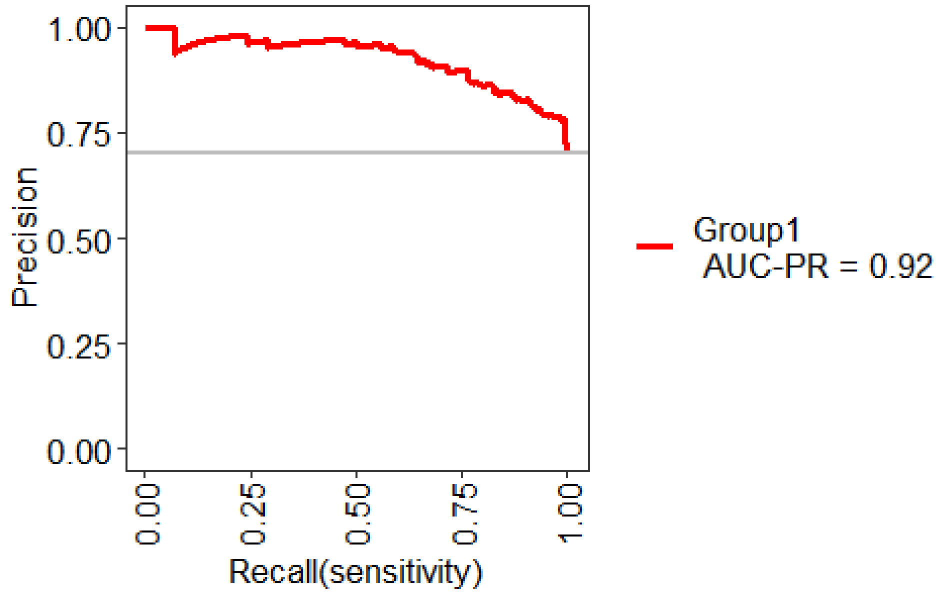

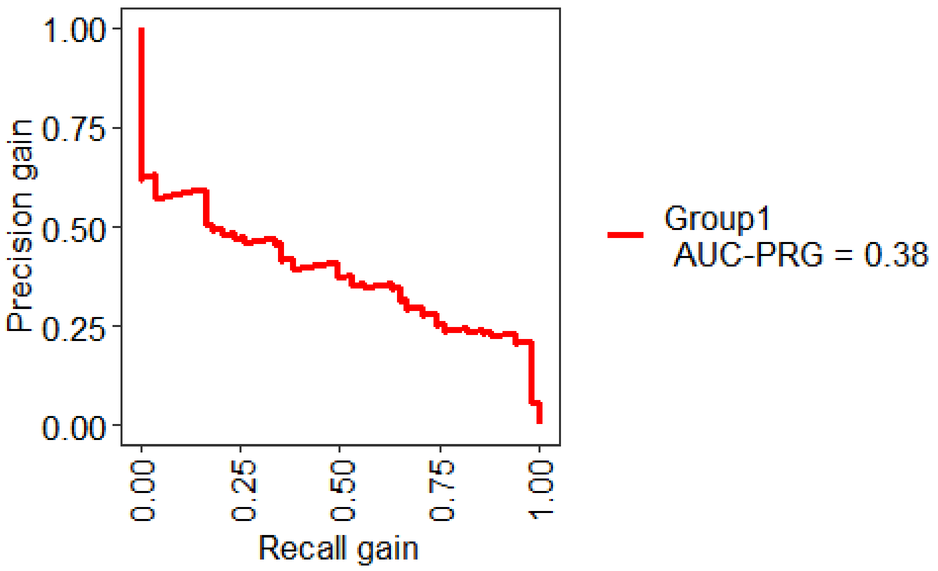
1.00
0.75
0.50
0.25
0.00
Precision gain
0.00
0.25
0.50
0.75
1.00
Recall gain
Group1
AUC-PRG = 0.38

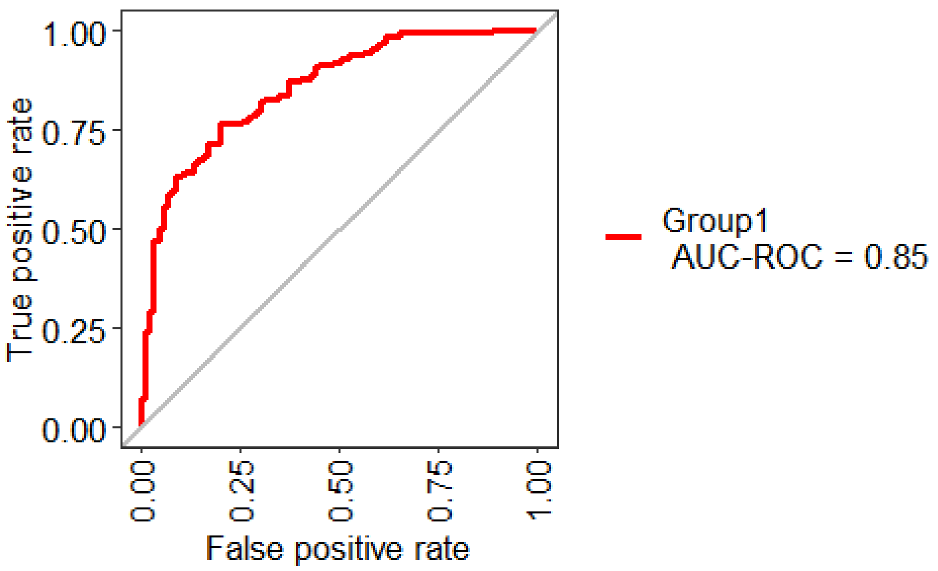
1.00
0.75
0.50
0.25
0.00
True positive rate
0.00
0.25
0.50
0.75
1.00
False positive rate
Group1
AUC-ROC = 0.85

```
ylr$stdres
```

```
## $Group1
##                 Score        CI
## SENS            0.942  0.9-0.97
## SPEC            0.427 0.33-0.53
## MCC             0.452      <NA>
## Informedness    0.369      <NA>
## PREC            0.794 0.74-0.84
## NPV             0.760 0.63-0.86
## FPR             0.573      <NA>
## F1              0.862      <NA>
## TP            196.000      <NA>
## FP             51.000      <NA>
## TN             38.000      <NA>
## FN             12.000      <NA>
## AUC-ROC         0.850 0.81-0.89
## AUC-PR          0.920      <NA>
## AUC-PRG         0.380      <NA>
```
