# Supplementary material for: Machine Learning-Based Three-Month Outcome Prediction in Acute Ischemic Stroke: A Single Cerebrovascular-Specialty Hospital Study in South Korea
Source: Diagnostics (Basel). 2021 Oct 15;11(10):1909. doi: 10.3390/diagnostics11101909 (PMC8534707; doi:10.3390/diagnostics11101909)
Supplement: Supplementary file 1 [file diagnostics-11-01909-s001.zip › Supplementary tables.pdf]

Supplementary Table S1. Optimal training model of each machine learning algorithm.

Algorithm	Balancing	Optimal hyperparameters
RLR	SMOTE	loss = L1, cost = 0.25, epsilon = 0.01
SVM	None	sigma = 0.02761147, C = 0.25
RF	ADASYN	ntree = 1500, mtry = 8
KNN	None	k (neighbors) = 83
XGB	None	nrounds = 500, max_depth = 6, eta = 0.01, gamma = 0, colsample_bytree = 0.6, min_child_weight = 1, subsample = 0.8

RLR regularized logistic regression, *SVM* support vector machines, *RF* random forest, *KNN* k-nearest neighbors, *XGB* extreme gradient boosting, *SMOTE* synthetic minority oversampling technique, *ADASYN* adaptive synthetic

Supplementary Table S2. Confusion matrix.

		Reference	
Algorithm	Prediction	Favorable	Unfavorable
RLR	Favorable	172	28
	Unfavorable	36	61
SVM	Favorable	188	39
	Unfavorable	20	50
RF	Favorable	183	42
	Unfavorable	25	47
KNN	Favorable	207	80
	Unfavorable	1	9
XGB	Favorable	185	45
	Unfavorable	23	44

RLR regularized logistic regression, *SVM* support vector machines, *RF* random forest, *KNN* k-nearest neighbors, *XGB* extreme gradient boosting
